# A new viewpoint on antlers reveals the evolutionary history of deer (Cervidae, Mammalia)

**DOI:** 10.1038/s41598-020-64555-7

**Published:** 2020-06-02

**Authors:** Yuusuke Samejima, Hiroshige Matsuoka

**Affiliations:** 1 Department of Geology and Mineralogy, Division of Earth and Planetary Sciences, Graduate School of Science, Kyoto University, Kitashirakawaoiwake-cho, Sakyo-ku, Kyoto 606-8502 Japan; 2 Present Address: Ministry of Agriculture, Forestry and Fisheries, 1-2-1, Kasumigaseki, Chiyoda-ku, Tokyo 100-8950 Japan

**Keywords:** Zoology, Evolution, Phylogenetics

## Abstract

Recent molecular phylogeny of deer revealed that the characters of antlers previously focused on are homoplasious, and antlers tend to be considered problematic for classification. However, we think antlers are important tools and reconsidered and analysed the characters and structures to use them for classification. This study developed a method to describe the branching structure of antlers by using antler grooves, which are formed on the antlers by growth, and then projecting the position of the branching directions of tines on the burr circumference. By making diagrams, comparing the branching structure interspecifically, homologous elements (tines, beams, and processes) of the antlers of 25 species of 16 genera were determined. Subsequently, ancestral state reconstruction was performed on the fixed molecular phylogenetic tree. It was revealed that Capreolinae and Cervini gained respective three-pointed antlers independently, and their subclades gained synapomorphous tines. We found new homologous and synapomorphous characters, as the antler of Eld’s deer, which has been classified in *Rucervus*, is structurally close to that of *Elaphurus* rather than that of *Rucervus*, consistent with molecular phylogeny. The methods of this study will contribute to the understanding of the branching structure and phylogeny of fossil species and uncover the evolutionary history of Cervidae.

## Introduction

Antlers are a symbolic part of cervids. Species identification and taxonomy of fossil species are mostly based on antlers^1–7^, owing to their frequent production as fossils and morphological diversity. Recent molecular phylogeny^8–12^ revealed that the characters of the antler that have been conventionally focused are considerably homoplasious^8^. There is a view that antlers are problematic as morphological characters for classification^12^, and attempts to combine antlers and phylogeny are currently inactive. In recent phylogenetic analyses using morphological characters^12,13^, the number of antler characters is not large. However, antlers are important characters of Cervidae, and we consider that they should be the main tool for the classification of Cervidae.

We think that the phylogeny of antlers reached its limits because the point of antler characters that have been focused upon are a problem. In particular, the branching structure of the antlers, that is, the homology of tines (as skeletal elements), is considered to be indispensable for classification, but there is still no common view. Widely accepted identification characters of antler homology, or at least those in which the same terminologies are used, are the brow tine and beam of many species^14–29^ and trez tine (or tres tine or tray tine) and bez tine (or bay tine) of *Cervus* and *Dama*^14,19,22,24,28,30^. Especially for the distal tines, there has been little identification of homology and, concomitantly, the terminologies of the tines have been unconsolidated or absent, which has caused confusion.

**Figure 1:**
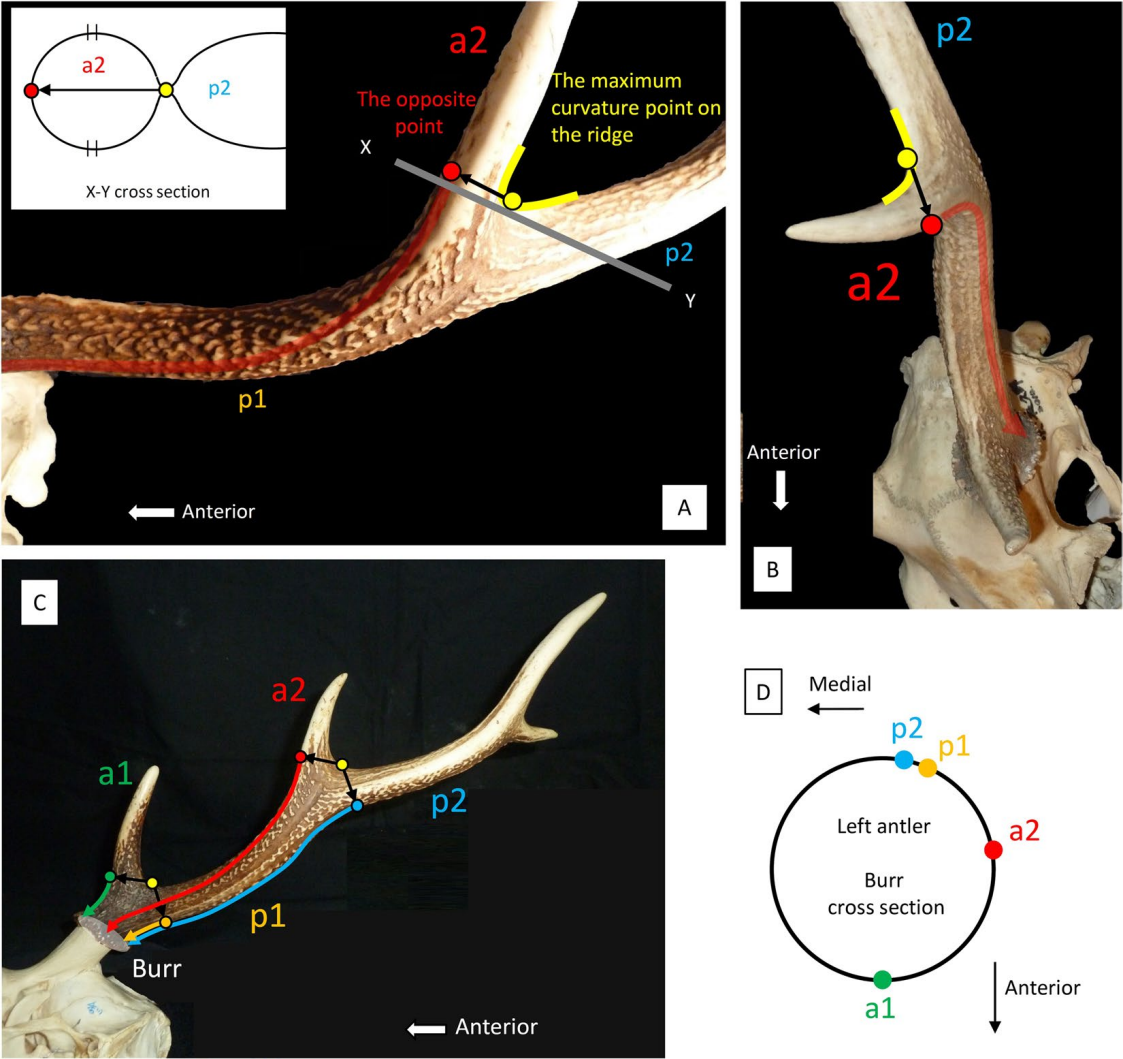
The method of determining the branching direction of tines using antler grooves. (*Cervus nippon*, KUGM-RM018) (**A)** Branching direction of a^2^ in the left lateral view. (**B)** Branching direction of p^2^ in the dorsal view. (**C)** Branching direction of multiple tines in the lateral view. (**D)** Diagram showing the branching direction of the tines on the burr cross section. Codes of a^1^, p^1^, a^2^ and p^2^ are from Pocock^33^.

**Figure 2:**
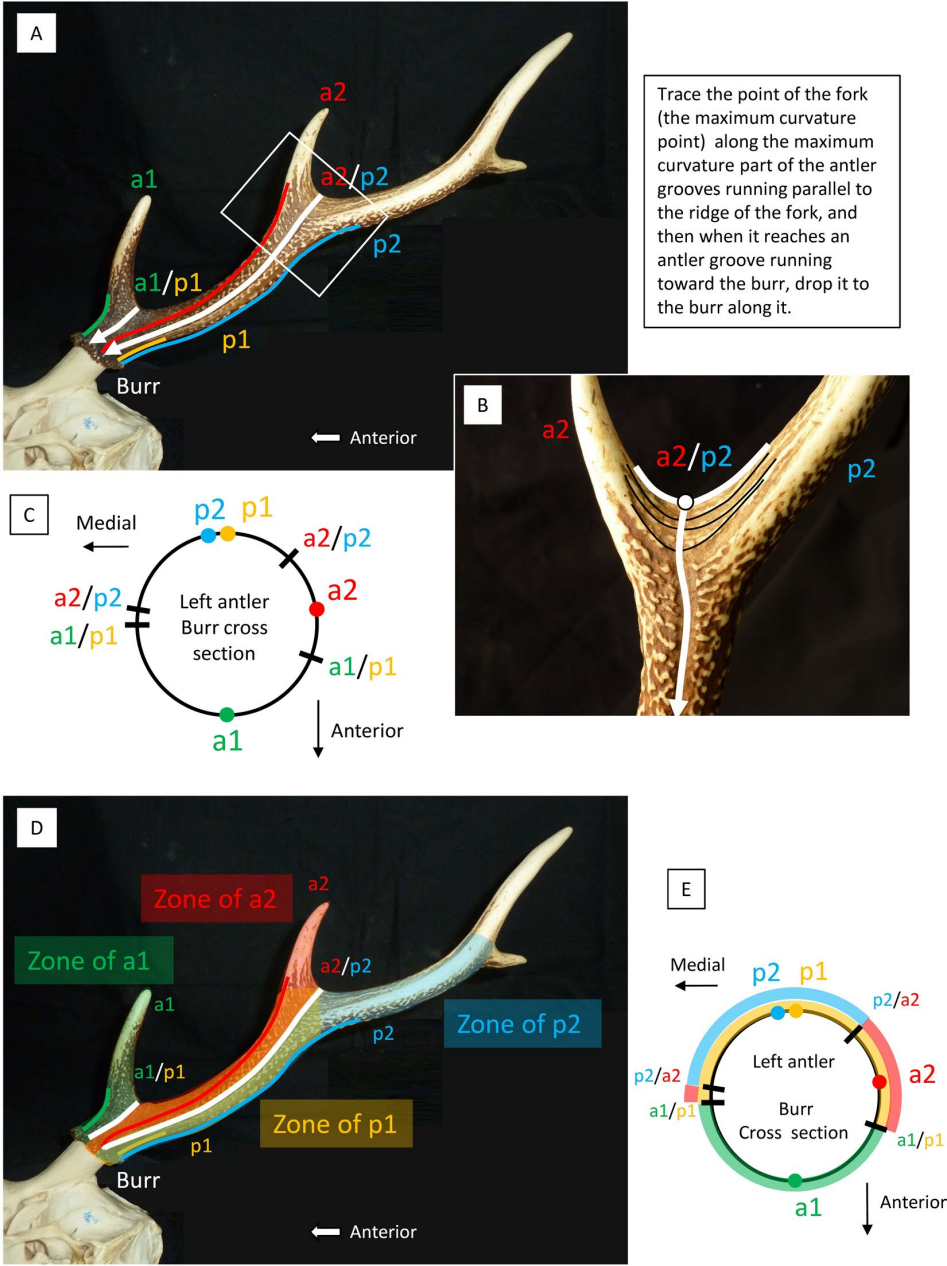
(**A**–**C**) Method of determining the positions of forks. (*Cervus nippon*, KUGM-RM018, left antler) (**A)** The position of fork a^2^/p^2^ and fork a^1^/p^1^ in the lateral view. (**B)** An enlarged view in the square with a white frame. (**C)** Diagram showing the positions of the forks on the burr cross section (the branching direction of the tines is also shown). (**D)** Coloured areas are the zones of the tines. The forks (white lines) are the boundaries of the zones of the tines. (**E)** Diagram showing the zones of the tines. Codes of a^1^, p^1^, a^2^ and p^2^ are from Pocock^33^.

Attempts to identify the homology of tines have been ongoing since before the 1900s^31,32^, but the most exhaustive work in the whole Cervidae family is that of Pocock^33^, who supposed that the antler consisted of anterior and posterior branching structures, and identified homologous tines as a^1^, p^1^, a^2^, p^2^…. However, there are criticisms that this is not true homology because antlers of different forms cannot be compared^34^, and because the tines of antlers evolved independently from more simple ones^35^. Later studies also differed from the view of Pocock between the tines of *Cervus* and *Axis*^13,36^ and between those of *Odocoileus* and *Blastocerus*^37,38^. In addition, Pocock’s method may not be able to certify the twisting of antlers and disappearance of tines (Supplementary Information 1-Figs. 1 and 2). To clarify the loss of a tine and twisting of the antler, the branching structure of the antler must be analysed in detail. The problem so far is that there have been no means to anatomically analyse the branch structure of antlers. Therefore, the recognition of homology was different among researchers, and terminology was either not given or confused. In this study, we devised methods to analyse the branching structure of antlers.

Antlers grow extensionally from the tip^39–41^ like a plant stem. Plant stems may sometimes twist when growing, but it is possible to recognise twisting from streaks on the epidermis, and we can correctly understand the structure of leaves branching from the stem^42^. Similar to plant stems, by paying attention to the streaks on antlers, which are called “antler grooves” (Sup. Inf. 1-Fig. 5), we think that it is possible to recognise this twisting and understand the branching structure of antlers.

**Figure 3:**
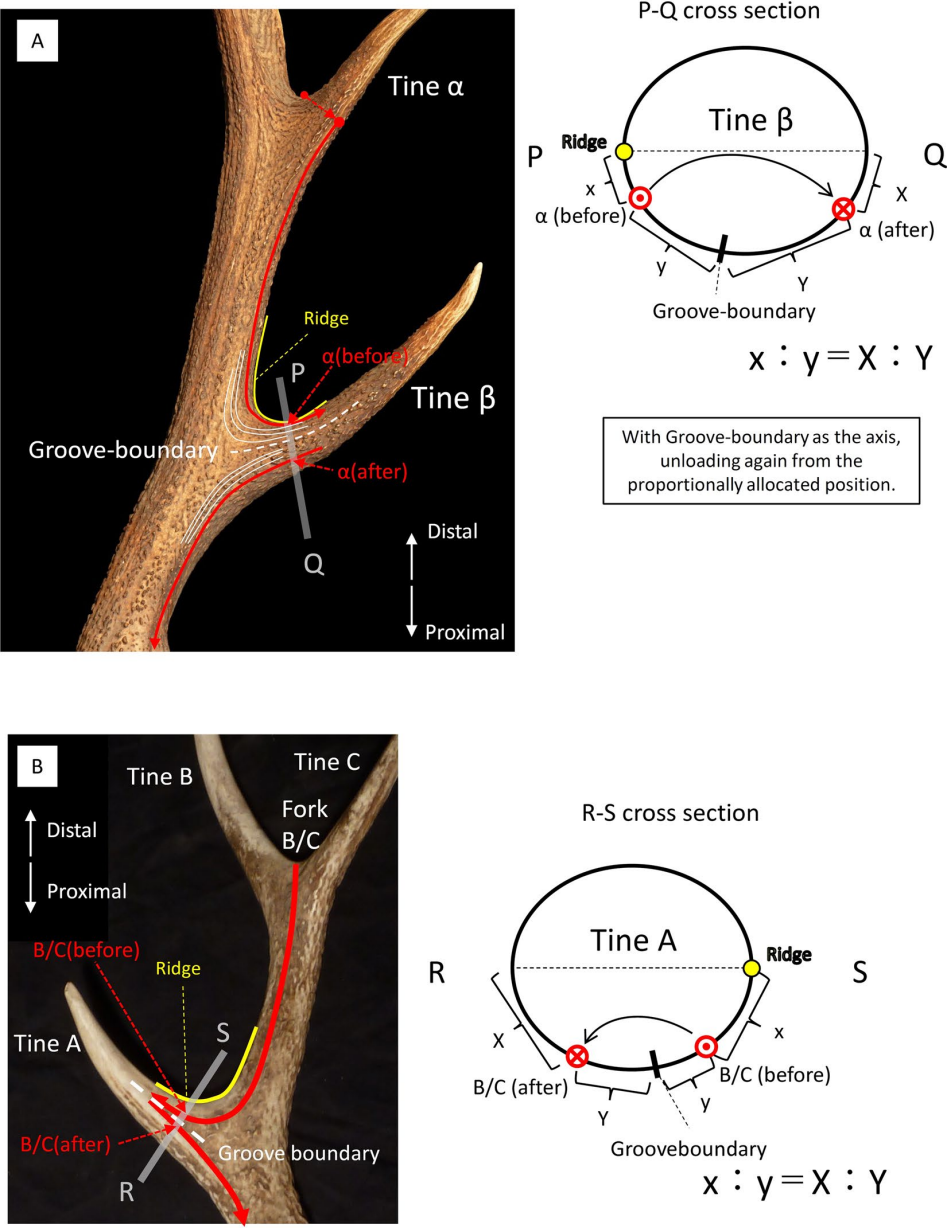
(**A**) A case where the branching direction of a tine overlaps with a proximal tine. (*Cervus elaphus*, KUGM-RM134) (**B)** Where the position of a fork overlaps with a proximal tine (*Cervus nippon*, KUGM-RM005).

**Figure 4:**
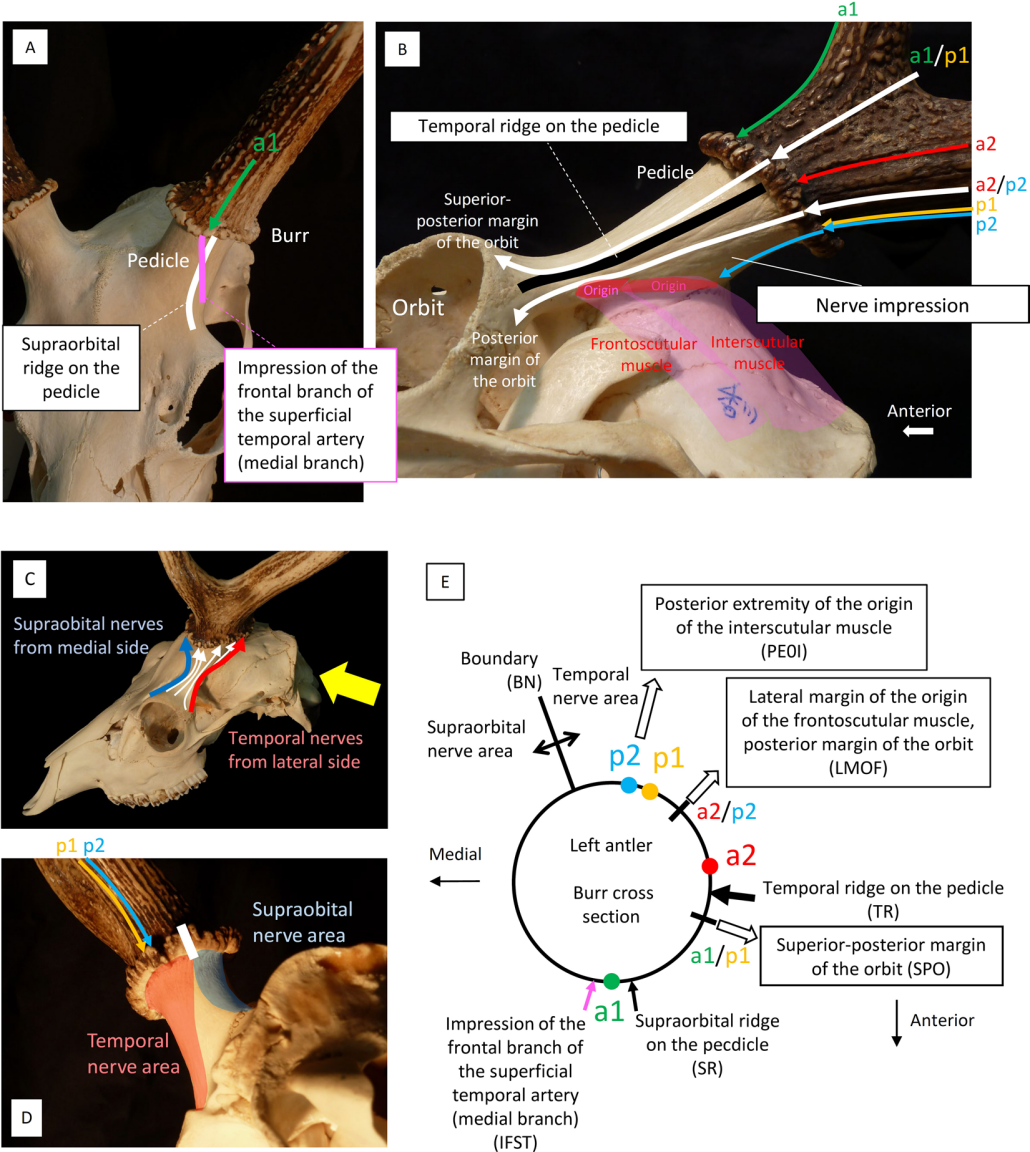
(**A**) Supraorbital ridge on the pedicle (SR) and impression of the frontal branch of the superficial temporal artery (medial branch) (IFST). IFST was not observed in any of the specimens. (*Cervus nippon*, KUGM-RM104, horizontally flipped) (**B)** Indices for determining the positional relation with the burr on the temporal region. (*Cervus nippon*, KUGM-RM018, left lateral view) (**C)** Nerves reaching the burr come from two areas: the supraorbital area and the temporal area. Nerves from the supraorbital area come through the medial side and those from the temporal area come through the lateral side (Wislocki and Singer^49^) (*Cervus nippon*, KUGM-RM001) (**D)** Viewing the individual in Fig. 4C from the direction of the yellow arrow. (**E)** The position of the indices with relation to the skull on the burr cross section. Codes of a^1^, p^1^, a^2^ and p^2^ are from Pocock^33^.

Antler grooves are composed of fine streaks and thick grooves running parallel to them. Fine streaks are thought to be formed by the extension force when the antler grows and can be observed at the tip of a velvet antler (Sup. Inf. 1-Fig. 3). The thick grooves are often observed in the proximal part, which runs almost parallel to the streaks, are traces of arteries, as mentioned in previous studies^43–45^ (Sup. Inf. 1-Fig. 4). The streaks and arterial impressions are often parallel, but sometimes the impressions may be oblique to the streaks, in which case the fine streaks may indicate the true growth direction). In other words, to know the branching structure based on the growth of the antlers, it may be appropriate to follow the antler grooves, that is, the fine streaks formed by the tensile force of growth and the trace of the arteries parallel to them.

From this new point of view, analysing the branch structure of antlers and determining the homology of tines is expected to link the morphology of antlers and molecular phylogeny and enable the classification of fossil species by the antlers.

## Methods

### Descriptive methodology

In this study, we analysed the branching structure of antlers by the following method using the grooves.

#### Branching directions of tines

First, the point of the fork where the target tine branches off the other tine (the point of the maximum curvature on the ridge) is located. Next, the cross section of the target tine taking the opposite point to the fork (the point dividing the circumference into two) is also located; this point indicates the branching direction of the tine. Then, this point is drawn along the antler grooves to the burr, and the branching direction is projected onto the burr. Figure 1A,B shows an example of tine a^2^ of *Cervus nippon*, and Fig. 1C shows the application of multiple tines of the same species. The branching direction of each tine is drawn down to the burr along the antler grooves. It can be projected onto the circle, presenting the cross section of the burr (Fig. 1D).

#### Positions of forks

The positions of forks where the tines bifurcate can be determined using the antler grooves. The point of the fork (at the maximum curvature point) is traced along the maximum curvature points of the antler grooves running parallel to the ridge of the fork. Then, when it reaches the antler grooves toward the burr, it is drawn down to the burr along them (Fig. 2B). Figure 2A shows an example of a^1^/p^1^, the fork where tines a^1^ and p^1^ branch (“*/*” represents a fork), and an example of a^2^/p^2^. For one fork, the line can be placed on the lateral or medial side; therefore, in the cross section of the burr, two positions are determined for one fork (Fig. 2C).

In addition, areas bounded by forks are taken as the “zone” of the tines (Fig. 2D). This is because the tines in which certain antler grooves reach distally from the burr are bound differently by the fork. This zone can be represented as shown in Fig. 2E in the cross section of the burr.

#### When overlapping with a more proximal tine

It is no problem when branching directions of tines or positions of forks come down to the burr, but often, they overlap with more proximal tines and do not reach the burr. In this case, we focused on the antler grooves on the proximal tine that overlaps. There are two flows of antler grooves in the proximal direction on the tine, one running to the burr and the other running to the other tine. In addition, a flow boundary exists. We named this the “groove-boundary” (Fig. 3). When overlapping (example of the branching direction of a tine in Fig. 3A), moving the point α(before) to the new point α(after) is prorated by centring the groove-boundary in the following manner: [length between the ridge and α(before) = x]: [length between α(before) and groove-boundary = y] = [length between the opposite side of the ridge and α(after) = X]: [length between α(after) and the groove-boundary = Y]. Then, α(after) is taken down to the burr. In the case of forks, this was the same (Fig. 3B).

The purpose of centring is to ensure that even when the overlapping proximal tine disappears, the branching direction of the tine or the positions of the fork will come down to the same points on the burr. The reason for performing proportional allotment is because it is presumed that antler grooves leading from the burr to the tine will fold back to more distal tines. Sup. Inf. 1-Fig. 6 is an example of this. In these antlers, the antler grooves leading to tine G and tine H turn back to the more distal tines. Thus, it is presumed that antler grooves leading to a certain tine pass through the tine, turning back and running to more distal tines. Therefore, we think this proportional allotment is appropriate so that the branching direction of the tine or the positions of the fork come down to the same points on the burr.

#### Positional relation with the skull

Antlers grow from the pedicles, protrusions of the posterior lateral area of the frontal bone, which is a part of the skull. Therefore, to determine the true branching structure of the antler, it is necessary to know how it is positioned on the skull; that is, to clarify the relative positional relation between the branching direction of the tines and positions of the forks, and some structural indicators of the skull. Therefore, several structures were used as indices.

In the frontal area (Fig. 4A), the ridge extending from the supraorbital region to the burr on the pedicle can be used as an index. We named it the supraorbital ridge on the pedicle (SR). In addition, the medial branch of the frontal branch of the superficial temporal artery, sending blood to the antler^46,47^, often makes an impression on the pedicle, which can also be used as an index. This is abbreviated as IFST.

In the temporal area (Fig. 4B), the ridge extending from the retro-orbital region to the burr on the pedicle can be used as an index. We named it the temporal ridge on the pedicle (TR). In addition, the frontoscutular muscle and the interscutular muscle adhere at intervals from the temporal ridge on the pedicle, with their lower edges bordering the temporal line^48^. Furthermore, considering the branching directions of the tines and the positions of the forks to extend along the impressions of the nerves^49^, thin streaks run on the pedicle. In the case of a specimen of *Cervus nippon* shown in Fig. 4B, a^1^/p^1^ is extended along the nerve impressions to the superior–posterior margin of the orbit, which is abbreviated as SPO. In the same way, a^2^/p^2^ is extended through the lateral margin of the origin of the frontoscutular muscle, to the posterior margin of the orbit, which is abbreviated as LMOF. p^1^ is extended to the posterior extremity of the origin of the interscutular muscle (PEOI).

In addition, the nerves running to the antler on the surface of the pedicle, which are derived from the trigeminal nerve, come from two areas: the supraorbital area and the temporal area. Nerves from the supraorbital area are through the medial side and those from the temporal area are through the lateral side^49^ (Fig. 4C). The boundary between them (hereafter abbreviated as BN) is on the posterior side of the pedicle (Fig. 4D), and is determined by the impression of nerves.

Figure 4E shows the position of the indices with relation to the skull on the burr cross section.

### Diagram

**Figure 5:**
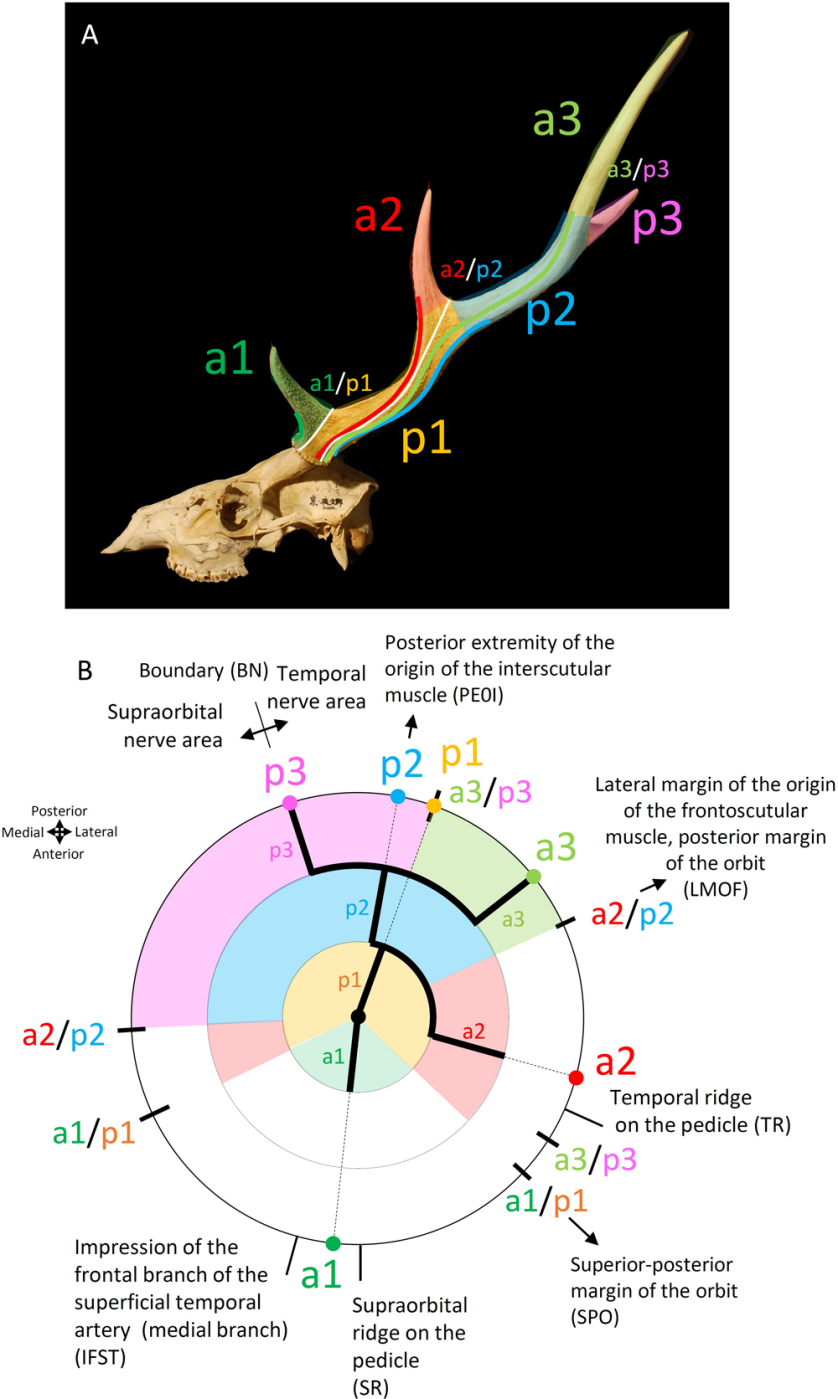
Diagram showing the branching structure of the left antler of *Cervus nippon* (KUGM-RM003). (**A)** Left lateral view. Codes of a^1^, p^1^, a^2^, p^2^, a^3^, and p^3^ obtained from Pocock^33^. (**B)** Diagram of the left antler. The code for each tine corresponds to Fig. 7A. The large circle represents the burr cross section, the dots are the branching direction of the tines, the short lines orthogonal to the circle are the positions of the forks, and the painted areas are the zones of the tines. Inside the circle, the branching hierarchical order among the tines is represented. The central dot is the base of the antler. Outside the circle, the positional relation with the skull is represented; thin lines are the ridges on the pedicle and the thin arrows are the positions extended along the nerve impressions.

We created a diagram to represent the branching structure information of antlers obtained by the above methods in one figure. Figure 5 shows a diagram of the left antler of *Cervus nippon*. This diagram contains all information on the structure and indices focused on in this study. The details of the diagram are explained in the legend of Fig. 5. The method of drawing the diagram is described in detail in Sup. Inf. 2.

### Determining the homologous tine and terminology

In this study, we compared, among the species, the positional order of the branching directions of tines, the positions of forks, the branching hierarchical positions of tines, and indices that determined the positional relation with the skull, on the diagrams. Tines at the same position on the diagram (have the same structure) were determined to be homologous.

We gave terminologies to the homologous tines. Given terminologies were based on previous literature as much as possible; however, new names were made for the tines with no terminologies in the literature. “-tine” and “-beam” were given to those whose length is normally more than twice the basal diameter, and “-process” was used for those normally less than twice the basal diameter. Although “-tine” and “-beam” are named, they are not conceptually distinguished in this study, because a tine inevitably becomes a beam if the top of it bifurcates.

### Reconstructing ancestral character states

After determining the homology of tines, we reconstructed the ancestral states of all tines, treating them as characters. The reconstruction was performed using the fixed topology of the most recent molecular phylogenetic tree, the Bayesian inference analyses of the combined molecular data set by Heckeberg^12^.

We set the character states 2, 1, and 0. When the tine was observed in 80% or more of the observed specimens of the species, the character state was 2. When observed in less than 80%, the character state was 1. When observed in no specimens, the character state was 0.

Between character states 0 and 1, reconstruction was performed by Dollo parsimony^50–52^. This parsimony is the method under the constraint that derived characters are gained only once, respectively, based on Dollo’s law^53^ that complicated derived characters do not evolve twice and it is never gained again once it is lost. In the evolution of antlers in Cervidae, it is presumed that a homologous tine with the same complex structure (having the same branching direction, fork position, and branching hierarchical position and at the same position in the indices determining the positional relation with the skull) is not doubled. This is because it is presumed that the genes expressing the homologous tine may not be acquired twice, but on the other hand, it can be lost forever if the gene is lost in the population.

Between character states 1 and 2, reconstruction was performed using standard parsimony (ACCTRAN). In evolutionary history, a tine that was newly acquired due to a mutation in genes is, if it is adaptive, likely to spread rapidly within the population after acquisition (character state 1 → 2). In contrast, if the tine is no longer adaptive in the offspring population, the rate within the population will decrease (character state 2 → 1). If a new tine is acquired by mutation and is adaptive, it should spread in a short time in the population. Therefore, when 0 → 1 occurs, it is likely that 1 → 2 occurs immediately after. It is unlikely that 1 → 2 will evolve many times in evolutionary history (though this is not without possibility that it will happen several times). In contrast, 2 → 1 is likely to occur independently after the offspring diverges when the tine is no longer adaptive. Therefore, we applied ACCTRAN.

### Materials

The species we observed in this study are listed in Table 1. The ancestral reconstruction was performed using adult specimens, the numbers of which are also shown in Table 1. Sixteen out of the eighteen extant genera^54^ are included. Only *Rucervus schomburgki* is currently extinct. *Hydrophotes intermis*, which is known to have no antlers, was also included in the samples to ensure that it had none. Species names are based on Wilson and Reeder^54^, except for Eld’s deer, which was revealed to belong to the different clade from *Rucervus duvaucelii* and *R. schoburgki* by multiple molecular phylogenic studies^8–12^. Therefore, we used *Panolia*^8,55^ for the genus containing Eld’s deer in this study. All specimens examined belonged to public museum/university collections. The specimen numbers and collection sources are listed in Sup. Inf. 9 (and 6).

**Table 1:** Species analysed in this study. N is the number of adult antlers used for the ancestral character state reconstruction, with left and right antlers counted separately. Specimens for which the species are not known are not included in this table.

*Alces alces*	n = 40
*Axis axis*	n = 20
*Axis porcinus*	n = 2
*Blastocerus dichotomus*	n = 2
*Capreolus capreolus*	n = 24
*Capreolus pygargus*	n = 31
*Cervus canadensis*	n = 34
*Cervus elaphus*	n = 27
*Cervus nippon*	n = 111
*Dama dama*	n = 22
*Elaphodus cephalophus*	n = 2
*Elaphurus davidianus*	n = 18
*Hydropotes inermis*	n = 4
*Mazama americana*	n = 2
*Muntiacus muntjak*	n = 44
*Muntiacus reevesi*	n = 4
*Odocoileus hemionus*	n = 21
*Odocoileus virginianus*	n = 82
*Panolia eldii*	n = 12
*Rangifer tarandus*	n = 49
*Rucervus duvaucelii*	n = 4
*Rucervus schomburgki*	n = 4
*Rusa marianna*	n = 6
*Rusa timorensis*	n = 4
*Rusa unicolor*	n = 62

## Results

### Homologous elements

**Figure 6:**
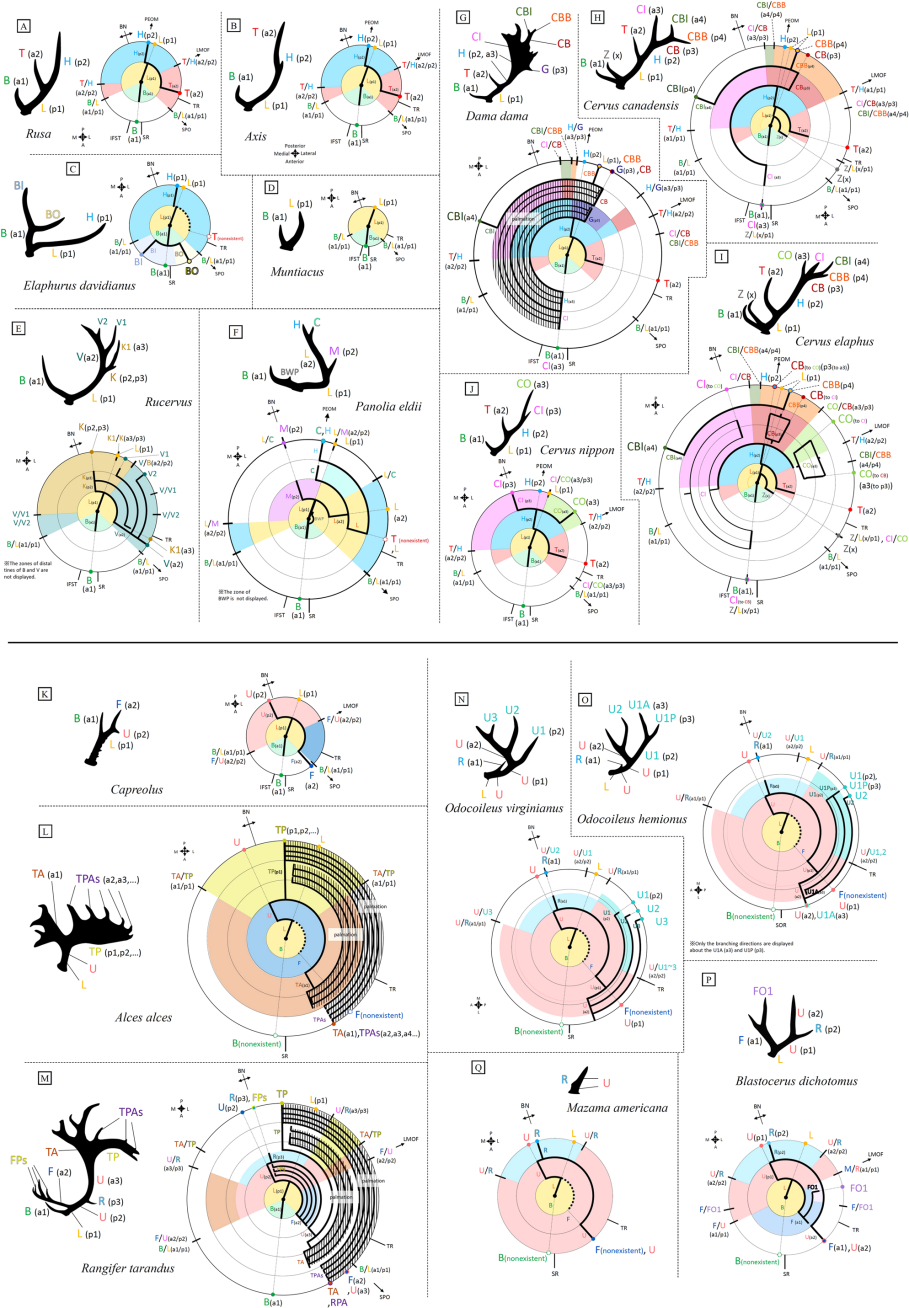
(**A**–**Q**) Typical forms of antlers of the species or genus with more than two-pointed antlers and the diagrams representing the branching structure (see the legend of Fig. 5 and Sup. Inf. 2 for information on how to read the diagram. Codes of a^1^, p^1^, a^2^, p^2^ … written in the brackets are from Pocock^33^). Identification of homologous elements, which are abbreviated as follows (written in coloured letters), from the results of this study. **B**: Brow tine, **BI**: Brow-inner tine, **BO**: Brow-outer tine, **BWP**: Brow process, **C**: Cacuminal tine, **CB**: Back-back tine, **CBI**: Crown-back-inner tine, **CBB**: Crown-back-back tine, **CI**: Crown-inner tine, **CO**: Crown-outer tine, **F**: Frontal tine, **FPs**: Frontal-posterior tines, **G**: Guard tine, **H**: Higher beam, **K**: Back beam, **K1**: Back-first tine, **L**: Lower beam, **M**: Medial tine, **PCs**: Pre-cacuminal tines, **R**: Rear tine, **TA**: Terminal-anterior tine, **T**: Trez tine, **TP**: Terminal-posterior tine, **TPAs**: Terminal-posterior-anterior tines, **U**: Upper beam, **U1**: Upper-1^st^ tine, **U1A**: Upper-1^st^-anterior tine, **U1P**: Upper-1^st^-posterior tine, **U2**: Upper-2^nd^ tine, **U3**: Upper-3^rd^ tine, **V**: Vertical beam, **V1**: Vertical-first tine, **Z**: Bez tine. The abbreviations for the relation with the skull are as follows: **SR**: Supraorbital ridge on the pedicle, **TR**: Temporal ridge on the pedicle, **IFST**: Impression of the frontal branch of the superficial temporal artery (medial branch), **SPO**: Reaching the superior–posterior margin of the orbit, **LMOF**: Reaching lateral margin of the origin of the frontoscutular muscle, to the posterior margin of the orbit. **PEIO**: Reaching the posterior extremity of the origin of the interscutular muscle, **BN**: Boundary of supraorbital nerve area and temporal nerve area.

In this study, we determined more than 40 homologous elements (tines, beams, and processes). The main conclusions are as follows (See Fig. 6).

In *Cervus*, *Rusa*, *Axis*, *Panolia*, *Elaphurus*, *Dama*, *Axis*, *Rucervus*, *Mutiacus*, *Capreolus*, and *Rangifer*, at the first fork, a^1^ is slightly lateral to the IFST and slightly medial to the SR. p^1^ is lateral to the BN. a^1^/p^1^ of the lateral side reaches the SPO though the pedicle. Therefore, tine a^1^ and p^1^ have the same structure among these genera, i.e., are homologous. Tines a^1^ and p^1^ in these genera are termed the brow tine and beam, respectively, in much of the literature^14–29^. Following this, we named a^1^ the brow tine and p^1^ the lower beam. The brow tine was observed in more than 90% of Cervini, *Muntiacus*, *Capreolus*, and *Rangifer*, and the lower beam was seen in 100% of species with more than two-tined antlers.

At the second fork of *Rusa*, *Axis*, *Cervus*, and *Dama*, a^2^ is posterior to the TR and p^2^ reaches the PEIO. a^2^/p^2^ reaches the LMOF through the pedicle. Therefore, a^2^ and p^2^ are homologous among these genera. a^2^ is named the trez tine based on many previous studies^16,19,22,23,25,26,28–30^. p^2^ is called the beam or main beam in previous studies^14,23,26,28–30^, and we named it the lower beam. The trez tine was observed in almost all specimens in *Rusa*, *Axis*, *Cervus*, and *Dama*, and rarely in *Elaphurs*.

At the second fork in *Capreolus* and *Rangifer*, a^2^ is posterior to SR and is slightly anterior to lateral a^1^/p^1^ (=brow tine/lower beam). p^2^ is slightly medial to the BN. The lateral a^2^/p^2^ reaches the LMOF through the pedicle, and the medial a^2^/p^2^ is at the same position as the medial a^1^/p^1^ (=brow tine/lower beam). Therefore, a^2^ and p^2^ are homologous among these two genera. In contrast, a^2^ and p^2^ of these genera have different structures from the above-described a^2^ (=trez tine) and p^2^ (=higher beam) of *Rusa*, *Axis*, *Cervus*, and *Dama*; they have different positional orders of the branching direction and fork positions, as well as differences in the indices determining the positional relations with the skull. (For example, a^2^ of *Rusa*, Axis, *Cevus*, and *Dama* is posterior to lateral a^1^/p^1^ [=brow tine/lower beam], whereas a^2^ of *Capreolus* and *Rangifer* is anterior to lateral a^1^/p^1^ [=brow tine/lower beam]). Therefore, they are not homologous. We gave new names to a^2^ and p^2^ of *Capreolus* and *Rangifer*. a^2^ was named the frontal tine based on previous usage^56^, and p^2^ was named the upper beam, because several examples in the literature^15,28,56^ termed this part the “beam” in *Rangifer*. In addition, in *Blastocerus*, a^1^ is between the SR and TR, p^1^ is slightly medial to the BN, and lateral a^1^/p^1^ reaches LMOF through the pedicle. Therefore, a^1^ and p^1^ of *Blastocerus* are the same as a^2^ and p^2^ of *Caprelus* and *Rangifer*, respectively. That is, a^1^ and p^1^ of *Blastocerus* are termed the frontal tine and upper beam, respectively. The frontal tine and upper beam were observed in almost all specimens in *Rangifer*, *Capreolus*, and *Blastocerus*, and very rarely in *Odocoileus*.

In *Cervus canadensis* and *Cervus elaphus*, x is between the TR and a^1^/p^1^ (=brow tine/lower beam). x/p^1^ (=x/brow tine) is at the same position as x. There are no other tines that have this structure in the typical antlers of the other species. This tine was named the bez tine based on its frequent usage in the literature^14,19,22,28–30^. The bez tine was observed in all specimens of *C. elaphus* and *C. canadensis*, and rarely in *Dama* and *Rusa unicolor*.

In *Panolia eldii*, p^2^ is at the BN and lateral a^2^/p^2^ is at the same position as p^1^ (= lower beam). This structured tine is not seen in the typical antlers of other species. This tine is called the medial tine. The medial tine was observed in 100% of *P. eldii* and rarely in *Elaphurus davidianus*.

In *Rangifer*, p^3^ is at the BN and a^3^ is at the same position as a^2^ (=frontal tine). In *Blastocerus*, p^2^ is at the BN and a^2^ is at the same position as a^1^ (=frontal tine). Therefore, p^3^ of *Rangifer* and p^2^ of *Blastocerus* are homologous; it was named the rear tine, based on previous usage^56^. This tine was observed in Odocoileini.

In this way, the homology of most antler elements (tines, beams and processes) was determined and the terminologies of tines were provided. The various types of antlers observed in this study are shown in Sup. Inf. 3. A detailed explanation of each homologous tine is provided in Sup. Inf. 4. A comparison between the homologous tines identified in the study and those described by Pocock^33^, as well as a comparison between the names given to tines in this study and those used previously in the literature, is shown in Sup. Inf. 5. The matrix of existence or nonexistence of the tines, beams, and processes of all specimens observed in this study is provided in Sup. Inf. 6. The matrix of percentages of the antler elements (tines, beams, and processes) of the species is shown in Sup. Inf. 7.

### Ancestral states and evolution

**Figure 7:**
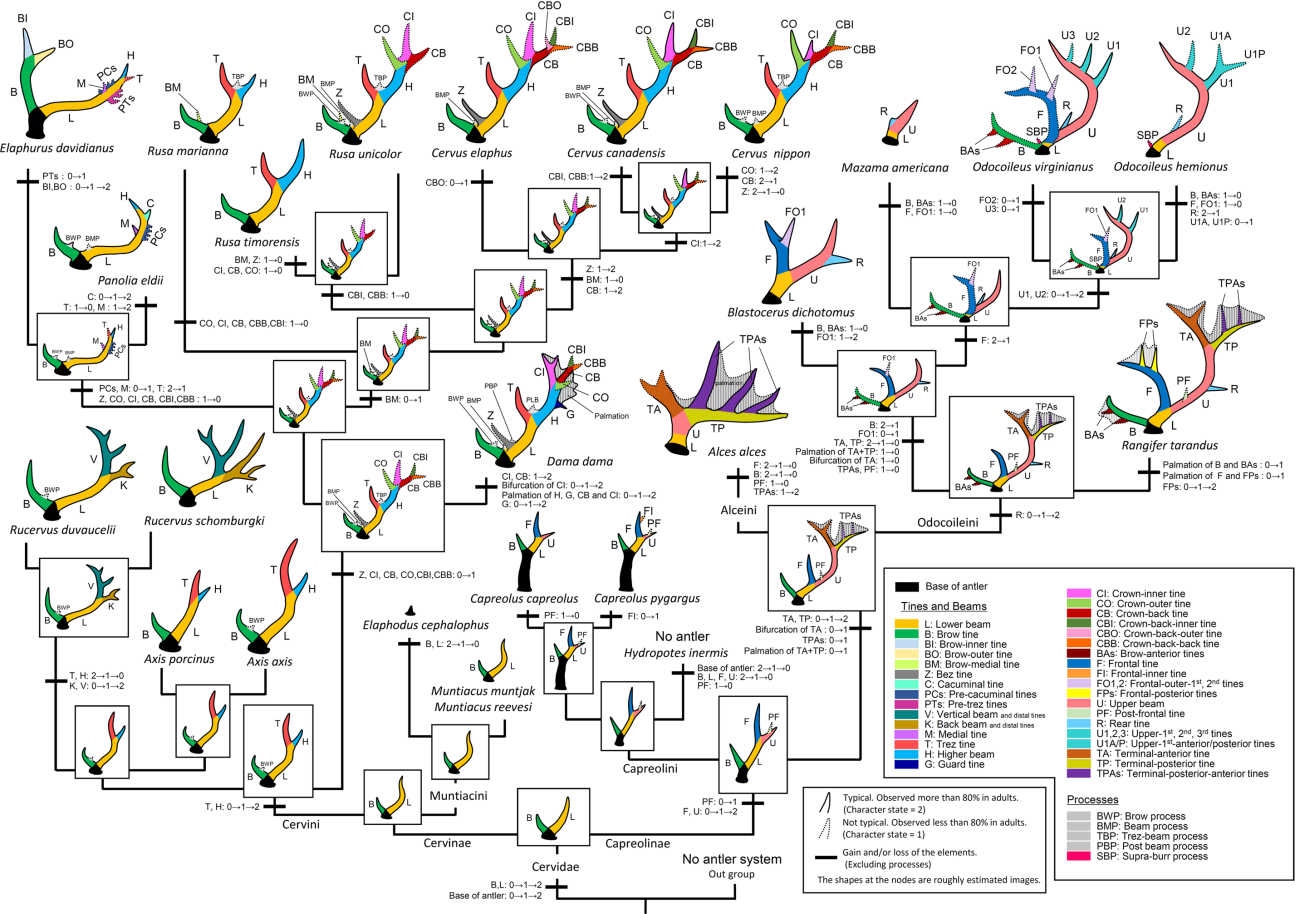
Evolution of the antlers of Cervidae. The silhouettes are the lateral view of the left antlers. The fixed topology of the molecular phylogenetic tree used for reconstruction is from Heckeberg^12^. The silhouettes depicted in the squares were reconstructed at the nodes. *Muntiacus reevesi* and *Muntiacus muntjak*, which are sister taxa, were represented as one because all antlers of the two species have the same character states.

Sup. Inf. 8 shows the results of the reconstruction of ancestral character states, from the fixed topology of Heckeberg^12^, of the respective elements (tines, beams, and processes) based on the homology determined in this study. Figure 7 shows the reconstructed ancestral states (depicted as silhouettes) at the nodes and the evolutionary history of the antlers.

The most recent common ancestor (MRCA) of the extant species had two-pointed antlers with the brow tine and the lower beam. In *Mutiacus*, the primitive two-pointed antlers were retained.

The common ancestor of Cervini possessed a brow tine, lower beam, trez tine, higher beam, and brow process. *Axis* retained primitive three-point antlers. *Rucervus* lost its trez tine and higher beam and gained vertical and back beams and distal tines. The common ancestor of *Dama*, *Elaphurus*, *Panolia*, *Rusa*, and *Cervus* gained the potential ability to express a crown-inner tine, crown-outer tine, and crown-back tine, which form a trifurcation (“crown”) at the end of the higher beam. *Dama* gained a guard tine and palmation of the distal portion of the higher beam. The clade of *Elaphurus davidianus* and *Panolia eldii* gained a medial tine and pre-cacuminal tines. *Panolia eldii* gained a cacuminal tine and *Elaphurus* gained pre-trez tines and the bifurcation of the brow tine. *Cervus* makes different forms for each species depending on the manner of existence of the distal tines on the higher beam. *C. nippon* lost its bez tine.

In Capreolinae, the common ancestor was typically three-pointed with the brow tine, lower beam, frontal tine, and upper beam, and *Capreolus* almost retained this form. *Hydropotes* lost their antlers completely. The clade of Alceini and Odocoileini gained the ability to express terminal-anterior/posterior tines. *Alces* lost its brow tine and frontal tine. Odocoileini gained a rear tine. In *Odocoileus*, the brow and frontal tines were not normally expressed, and upper-1^st^, 2^nd^, and 3^rd^ tines were obtained.

## Discussion

### Arrangement of the terminology of homologous elements

As a result of this study, the correspondence between homologous elements and previous terminology was clarified. For example, the “posteromedial tine”^27^ or “back-inner tine”^21^ and “anterolateral tine”^27^ or “front-outer tine”^21^ in *Rusa and Axis* are homologous to the “trez tine”^16,19,22,23,25,26,28–30^ and posterior portion of the “beam”^14,23,26,28–30^ in *Cervus elaphus*, respectively. We named the former the trez tine and the latter the higher beam. Further, the “bez tine”^28^ of *Rangifer tarandus* was not homologous to the “bez tine”^14,19,22,28–30^ of *Cervus*. We named the former the bez tine and the latter the frontal tine. In this study, we also gave terminologies to the elements that have not been given them before. It can be said that a measurable arrangement was performed for the identification of these antler elements in the future. (See Sup. Inf. 5 for the correspondence between terminologies in this study and those previously used).

### Different three-pointed structures between Cervini and Capreolinae

This study revealed that three-pointed structures are different between Cervini and Capreolinae. Compared to the basic three-pointed forms of Cervini and Capreolinae, the brow tine and lower beam are homologous, but the tines bifurcating at the end of the lower beam were the trez tine and higher beam in Cervini, and the frontal tine and upper beam in Capreolinae. The view of identifying the homology of the tines at the second fork between Cervini and Capreolinae^32,33,57^ needs to be dismissed completely. At the same time, the MRCA of the extant species was revealed to have been two-pointed. Although it is known that primitive fossil cervids, such as *Procervulus* and *Dicrocerus*, are two-pointed, it was revealed that the antler of the MRCA of extant species was still two-pointed at that stage. The presumption that the MRCA was three-pointed^9^ was contradicted in this study.

### *Panolia eldii* has a different structured antler to *Rucervus*

Eld’s deer belongs to *Rucervus*^35,54^, but all recent molecular phylogenetic topology studies^8–12^ show that the position of this species is not in the same clade as *Rucervus duvaucelii* and *R. schomburgki*, but in a clade with *Elaphurus davidianus*; therefore, Pitra *et al*.^8^ used the genus *Panolia*, and this study follows it. Analysis of the antler in this study revealed that the branching structure of the antler of *Panolia eldii* was completely different from that of *Rucervus*, and was closer to that *Elaphurus*, reflecting the molecular phylogeny.

The p^2^ branching direction (in Pocock^33^) of *Panolia eldii*, named the medial tine in this study, is at the BN, and the branching direction of its counterpart a^2^, the lower beam, is posterior to the TR. The medial tine/lower beam (the position of the fork) (lateral side) is at the same as the branching direction of the lower beam (to the brow tine). In contrast, in *Rucervus*, the branching direction of p^2^ (in Pocock^33^), the back beam (in this study), is lateral to the BN, and its counterpart a^2^, the vertical beam, is anterior to the TR. The vertical beam (a^2^)/back beam (p^2^) (the position of the fork) is not the same as the branching direction of the lower beam. Examination of the other tines does not reveal any homologous elements except for the brow tine and lower beam. The branching structure of the antler of *Panolia eldii*, which has been found in *Rucervus* owing to its unique curved antler form, was quite different, which is consistent with the molecular phylogeny.

On the other hand, it was revealed that there was plural synapomorphy between *Panolia eldii* and *Elaphurus davidianus*, which are sister taxa by molecular phylogeny.

The medial tine (100% in *P. eldii* and 11% in *E. davidianus*), pre-cacuminal tines (40% in *P. eldii* and 33% in *E. davidianus*), and loss of the trez tine (100% in *P. eldii* and 94% in *E. davidianus*) (Sup. Inf. 3-Figs. 27–31) are synapomorphies in the two genera.

### Crown tines of *Cervus* and *Dama*

The study also provided a new view into the distal tines of *Cervus* and *Dama*. The distal tines of *Cervus elaphus* are often termed “crown (tines)”^25,28,30^. This terminology is also used in *Cervus canadensis*^29^. However, the distinction between the tines in the crown has not yet been clarified, and an inter-specific comparison of the inside tines of the crown has not yet been performed. The most complex antler in *Cervus* is that of *C. elaphus*, whose typical antler trifurcates at the distal end of the higher beam. We named these three tines the crown-inner tine, crown-outer tine, and crown-back tine. The crown-back tine sometimes trifurcates at the distal end, and we named these three tines the crown-back-inner tine, crown-back-outer tine, and crown-back-back tine (Sup. Inf. 3-Figs. 10–13). As a result of the comparison between *C. elaphus* and *C. canadensis*, it was revealed that the distal tines at the end of the higher beam in the typical antler of *C. canadensis* were the crown-inner tine and crown-back tine, and the crown-back-inner tine and crown-back-back tine distally branching from the crown-back tine (Sup. Inf. 3-Fig. 14). Furthermore, in the typical antler of *Cervus nippon*, it was revealed that the distal tines at the end of the higher beam were the crown-inner tine and crown-outer tine (Sup. Inf. 3-Fig. 15). As evidence, trifurcation containing the crown-back tine is sometimes seen in *C. nippon* (Sup. Inf. 3-Figs. 16 and 17). In *Cervus*, the recognition of homology of the brow tine, bez tine, trez tine (or tres tine), beam and crown (or cornet, or royal) tines has been well established^14,19,22,23,29^, but the structure and homology of the inside tines of the crown have not been studied at all. To the best of our knowledge, this study solved the complex inside structure of the crown of *Cervus* and the homology of the tines inside it for the first time.

In addition, this study revealed the distal structure of the antler of *Dama dama*. The distal portion of the adult antler of *Dama dama* is palmate, whereas that of the juvenile antler is not palmate. Analysis of the juvenile antler identified the crown-inner tine and crown-back tine (Sup. Inf. 3-Fig. 20). In addition, analysis of adults revealed that the palmate parts are mainly the crown-inner tine and crown-back tine (Sup. Inf. 3-Figs. 21–23). As evidence, some mutant antlers have a crown-outer tine (Sup. Inf. 3-Fig. 24). (In addition, *Dama dama* has an autapomorphy, termed the guard tine, at the bottom of the palmation. The terminology “guard tine” is used by the Australian Deer Association^58^). For the antler of *Dama dama*, like in *Cervus*, wide recognition of homology of the proximal portion of the antler exists with *Cervus* species for the brow tine, bez tine, trez tine, and beam^16,20,24,26^, but regarding the distal portion, palmation needs to be focused upon, and homologous elements with other species are not been known. The new viewpoint of this study has revealed that the branching structure of the distal portion of *Dama dama* is homologous to that of *Cervus*.

### Curious antlers in Capreolinae: especially those in *Alces* and *Odocoileus*

Comparisons of the antlers of *Alces* and *Odocoileus* with those of *Capreolus*, *Rangifer*, and *Blastocerus* have been seldom made^33,37,38^ but there is a poor unified view of homology and terminologies. In this study, we revealed the remarkable homology of Capreolinae antlers.

First, a^1^ of *Blastocerus* is the frontal tine, which is not homologous to a^1^ = brow tine of *Rangifer* and *Capreolus* but was homologous to a^2^. Therefore, it was revealed that the brow tine was lost in *Blastocerus*.

For *Alces alces*, it was revealed that the palmate portion of its antler is homologous to the distal portion of that in *Rangifer tarandus*. Photographs of the specimens provided in Sup. Inf. 3-Fig. 41 show the similarity between the two (the distal portion of the antler of *Rangifer* is often palmate like *Alces*). We named this portion the terminal-anterior/posterior tines based on the previously used terminology, “terminal tines”^15^. Therefore, in *Alces*, the brow tine and frontal tine were completely lost, and only the terminal-anterior/posterior tines were enlarged. This study has identified the great peculiarity of the antler of *Alces*.

This study also revealed that *Odocoileus* has a very specific structured antler. It was difficult to recognise the homologous correspondence to the other genera from the antler of *Odocoileus* when we observed only the typical antler. The clue came from the NSMT-M32362 specimen of *O. virginianus* in the National Science Museum (Sup. Inf. 3-Figs. 45–47). This specimen has a very abnormal left antler. The typical antler part corresponds to the posterior portion of this abnormal one. By comparing to the antler of *Rangifer tarandus*, it was revealed that this abnormal antler has both a brow tine and frontal tine. It almost trifurcates (strictly bifurcates twice) at the proximal part, the anterior branch is the brow tine, the middle is the frontal tine, and the posterior is the upper beam (lower beam is very short). It was revealed that the first anterior tine of a typical antler of *Odocoileus*, which is often called the “sub-basal snag^59,60^”, is homologous to the posterior branching tail-like tine in *Rangifer* (rear tine). Therefore, in *Odocoileus*, the brow tine and frontal tine are completely lost normally, the whole antler is significantly twisted, and the rear tine, which branches off posteriorly in *Rangifer* and *Blastocerus*, branches anteromedially at the relatively proximal part of the antler. It became clear that the lower beam was very short and the upper beam, from which autapomorphic tines (upper-1st, 2nd, and 3rd tines) branch off, extended greatly.

Thus, it was uncovered that *Odocoileus* has a very specific and surprisingly peculiar antler that loses proximal tines and is entirely twisted.

### Application to the phylogeny of fossil species

In this study, we clarified the structural differences between the three-pointed antlers of Cervini and Capreolinae. Comparing the antler of a fossil species with the basic branching structure of antlers of both clades will help to determine which clade the fossil species belongs to.

Moreover, as in the case of *Panolia eldii* (which was traditionally classified in *Rucervus* based on the external form of the antler but was reconfigured into the same clade as *Elaphurus* by molecular phylogeny), in this study, the antler was revealed to be different in structure from *Rucervus* and have some synapomorphies with *Elaphurus*, in accordance with the molecular phylogeny. There is a great possibility that, from a new point of view using the antler grooves of this study, the systematic position of the fossil species that had been previously classified only by the external form of their antlers could be reconfigured.

As it was uncovered that the palmate portion of the antler of *Alces* was in fact homologous to the distal portion of that of *Rangifer*, by re-examining, from the viewpoint of this study, the structure of the distal portion of the fossil antlers that had not been noticed so far, the phylogenetic classification could be revisited. Naturally, the viewpoint of this study will also contribute to the taxonomy and phylogeny of newly generated fossil species. The new point of view in this study has the potential to clarify the evolutionary history of the whole Cervidae family, including fossil species.

## Supplementary information

Supplementary information 1 - Antler grooves.

Supplementary information 2 - The way of drawing the diagram showing branching structure.

Supplementary information 3 - Various types of antlers observed in this study.

Supplementary information 4 - Explanation of homologous elements (tines, beams and processes).

Supplementary information 5 - Comparison of the elements with Pocock (1933) and other previous works.

Supplementary information 6 - Matrix of existence of the elements of all the specimens observed in this study.

Supplementary information 7 - Percentages of the elements by species.

Supplementary information 8 - Ancestral state reconstruction of the homologous elements.

Supplemenatary information 9 - List of the specimens observed in this study.
