# Publisher Correction: A new viewpoint on antlers reveals the evolutionary history of deer (Cervidae, Mammalia)

**DOI:** 10.1038/s41598-020-69877-0

**Published:** 2020-07-31

**Authors:** Yuusuke Samejima, Hiroshige Matsuoka

**Affiliations:** 1 Department of Geology and Mineralogy, Division of Earth and Planetary Sciences, Graduate School of Science, Kyoto University, Kitashirakawaoiwake-cho, Sakyo-ku, Kyoto, 606-8502 Japan; 2 Present Address: Ministry of Agriculture, Forestry and Fisheries, 1-2-1, Kasumigaseki, Chiyoda-ku, Tokyo, 100-8950 Japan

Correction to: *Scientific Reports*https://doi.org/10.1038/s41598-020-64555-7, published online 02 June 2020

In the original version of this Article, the supplementary information files were incorrectly ordered, resulting in multiple mislabelled files.

In addition, Table 1 of Supplementary Information File 7, which had been initially provided for peer review, was omitted from the Supplementary Material originally published with this Article.

These errors have been corrected in the HTML version of the Article; the PDF version was correct at time of publication.

