# Supplementary material for: A new viewpoint on antlers reveals the evolutionary history of deer (Cervidae, Mammalia)
Source: Sci Rep. 2020 Jun 2;10:8910. doi: 10.1038/s41598-020-64555-7 (PMC7265483; doi:10.1038/s41598-020-64555-7)
Supplement: Supplementary file 1 — Supplementary information 1 - Antler grooves. [file 41598_2020_64555_MOESM1_ESM.pdf]

A new viewpoint on antlers reveals the evolutionary history of deer (Cervidae, Mammalia)

Yuusuke Samejima & Hiroshige Matsuoka

Supplementary Information 1

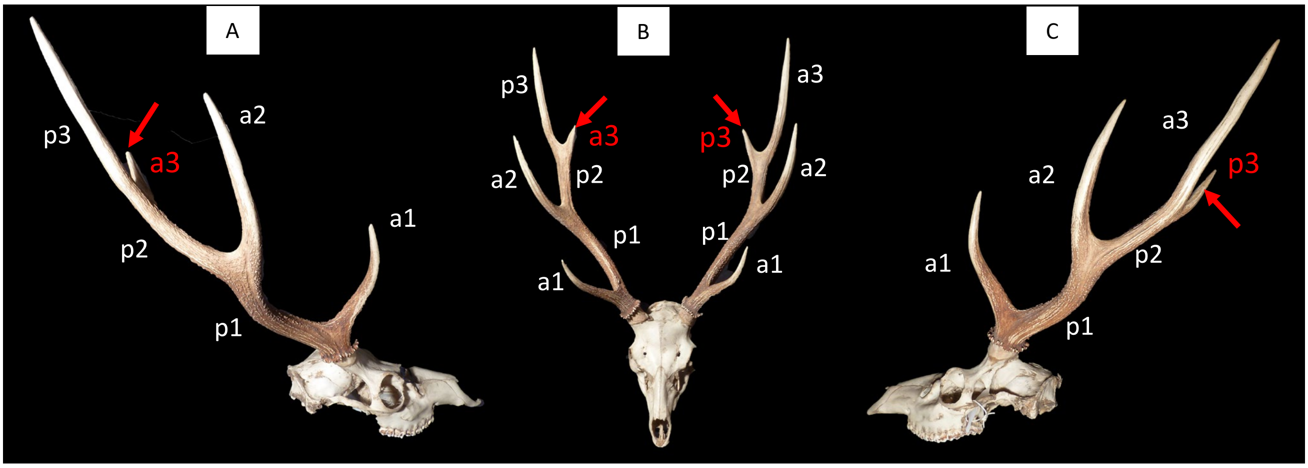

Figure 1. *Cervus nippon* (KUGM-RM005) A) Right antler in the lateral view, B) Dorsal view, C) Left antler in the lateral view. Giving codes to tines by the method of Pocock (1933). The tines indicated by the red arrows are given different codes on the left and right because anterior-posterior relation are converse by difference of degree of twisting.

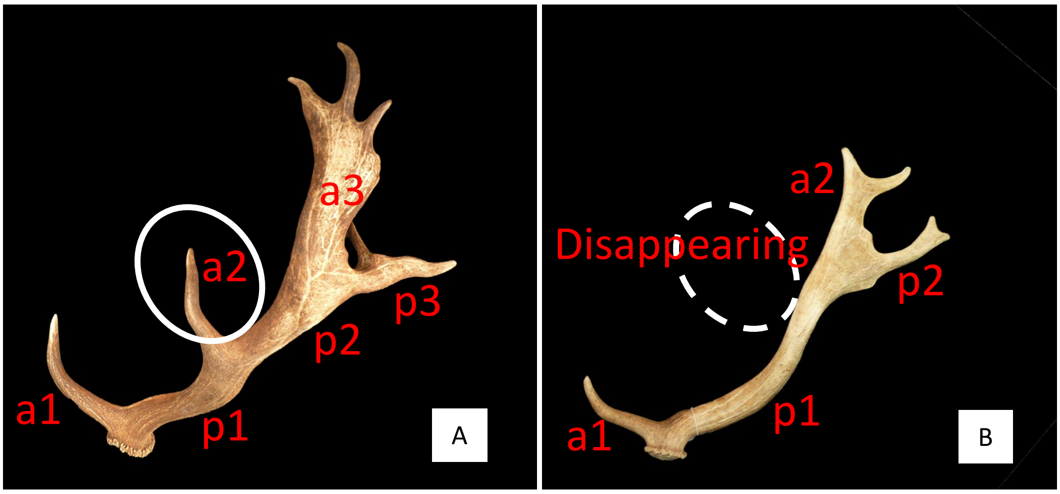

Figure 2 *Dama dama* A) KUGM-RM107 , horizontally flipped, B) KUGM-RM143, horizontally flipped. Giving codes to tines by the method of Pocock (1933). The tine that is presumed to be disappearing is skipped.

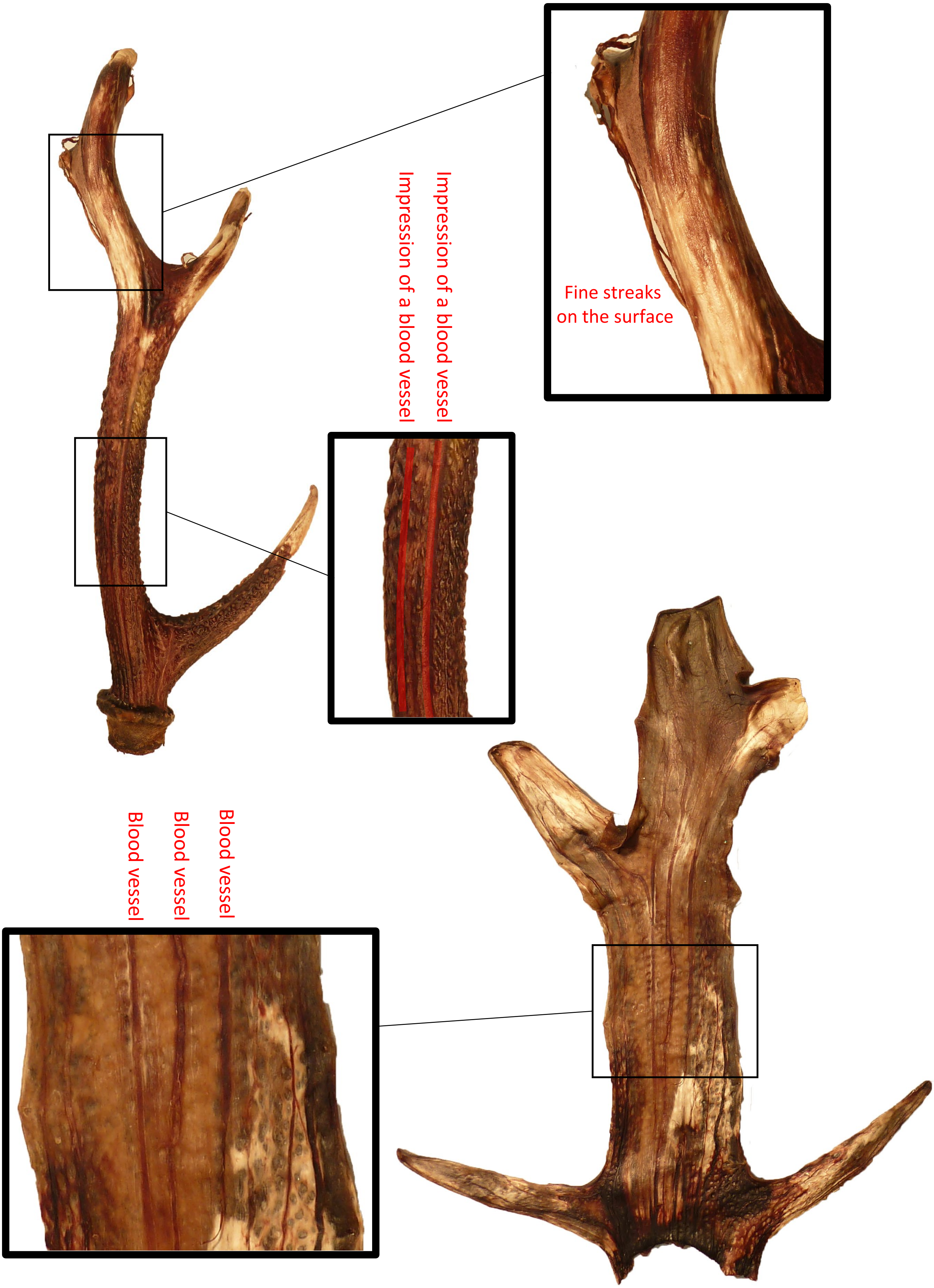

Figure 3. A dried specimen of a velvet antler of *Cervus nippon* (KUGM-RM206) .
1. Near the top, thin streaks are formed by tension of extending. 2. In the proximal area, blood vessels flow in the dermis along the thin streaks.

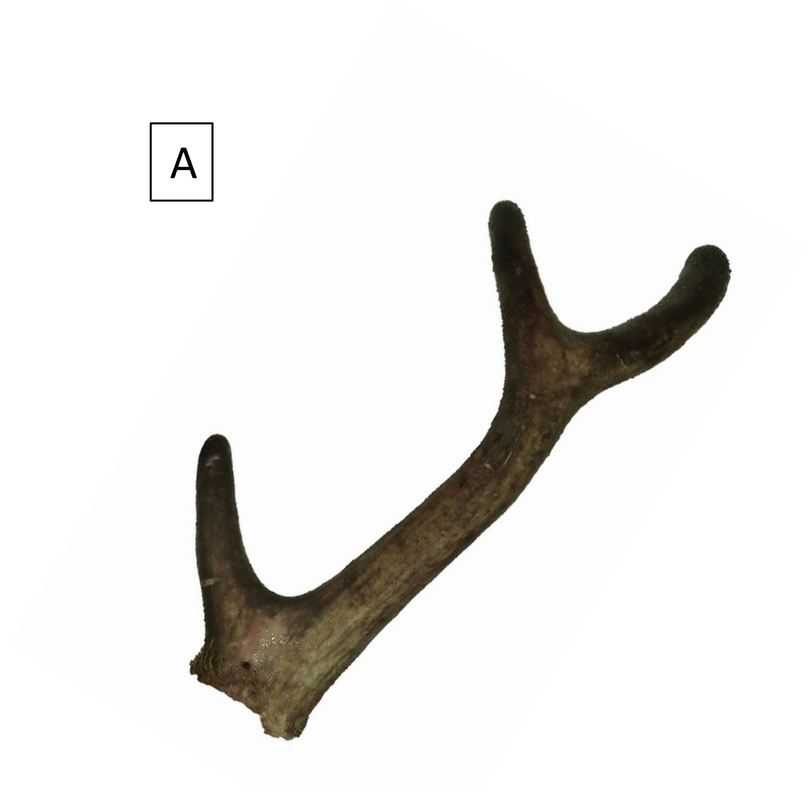

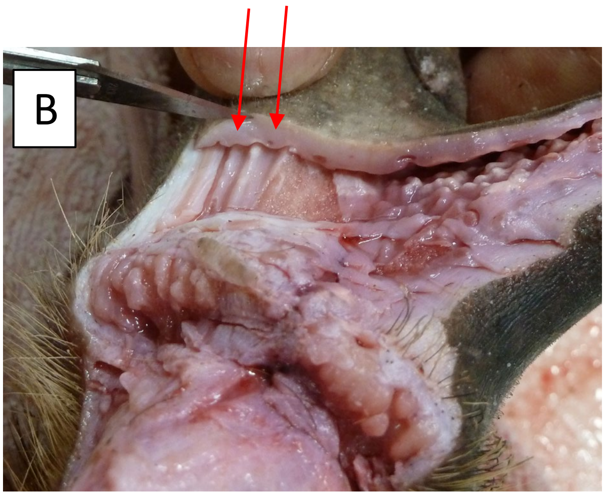

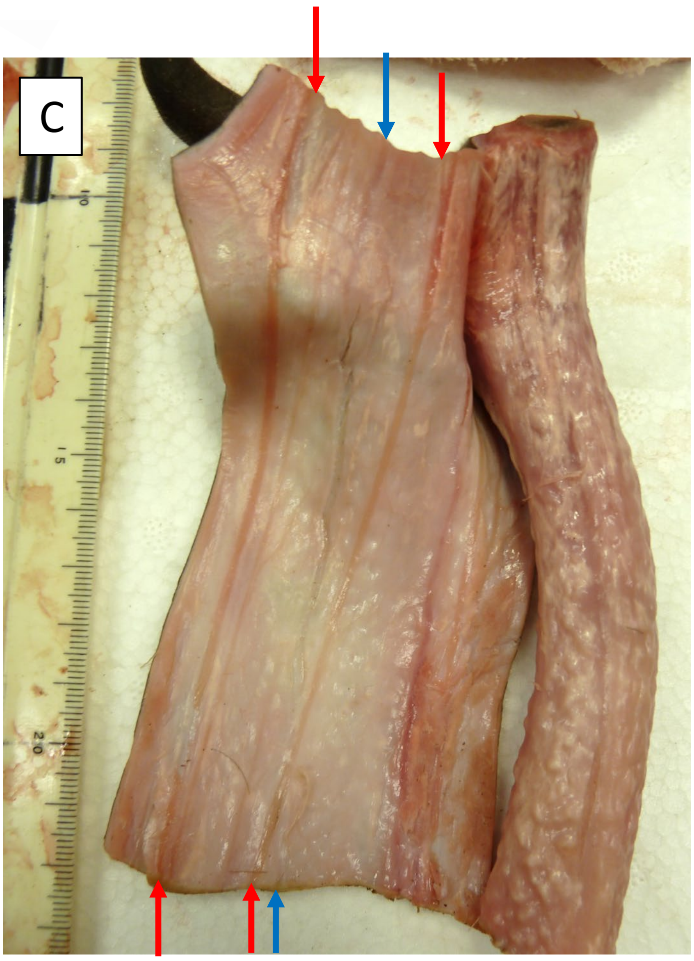

Figure 4.
A wet specimen of a velvet antler of *Cervus nippon* (KUGM-RM221). A) The natural outside appearance. B) The velvet was peeled off around the burr. Vessels were recognized corresponding to antler grooves. (Shown by red arrows.) C) The velvet in the center of the antler was peeled off. The red arrows show arteries corresponding to antler grooves, and the blue arrows show veins not corresponding to them.

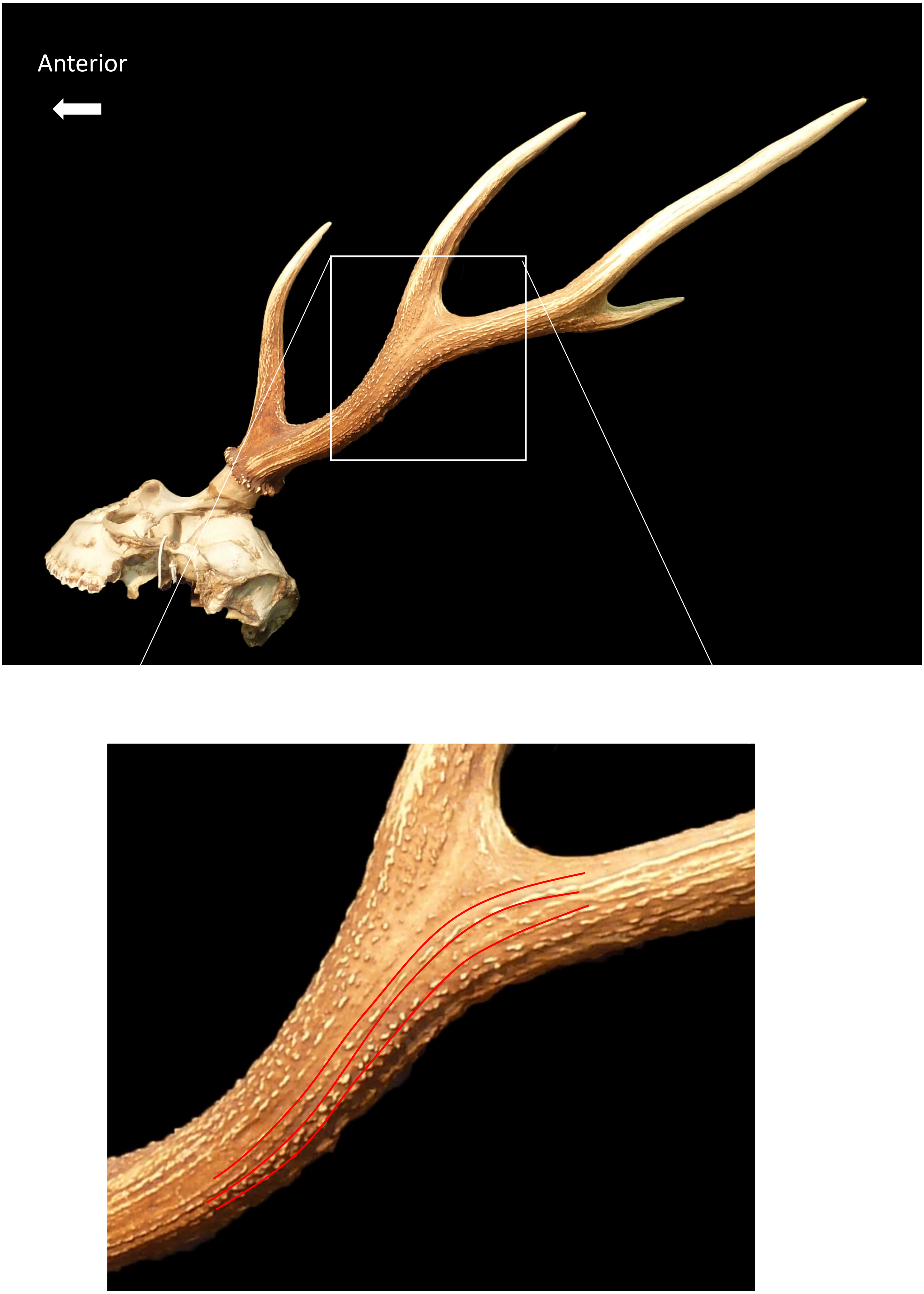

Figure 5. *Cervus nippon* (KUGM-RM005) in the lateral view of the left antler. Antler grooves are recognized, shown in the red lines in the lower picture.

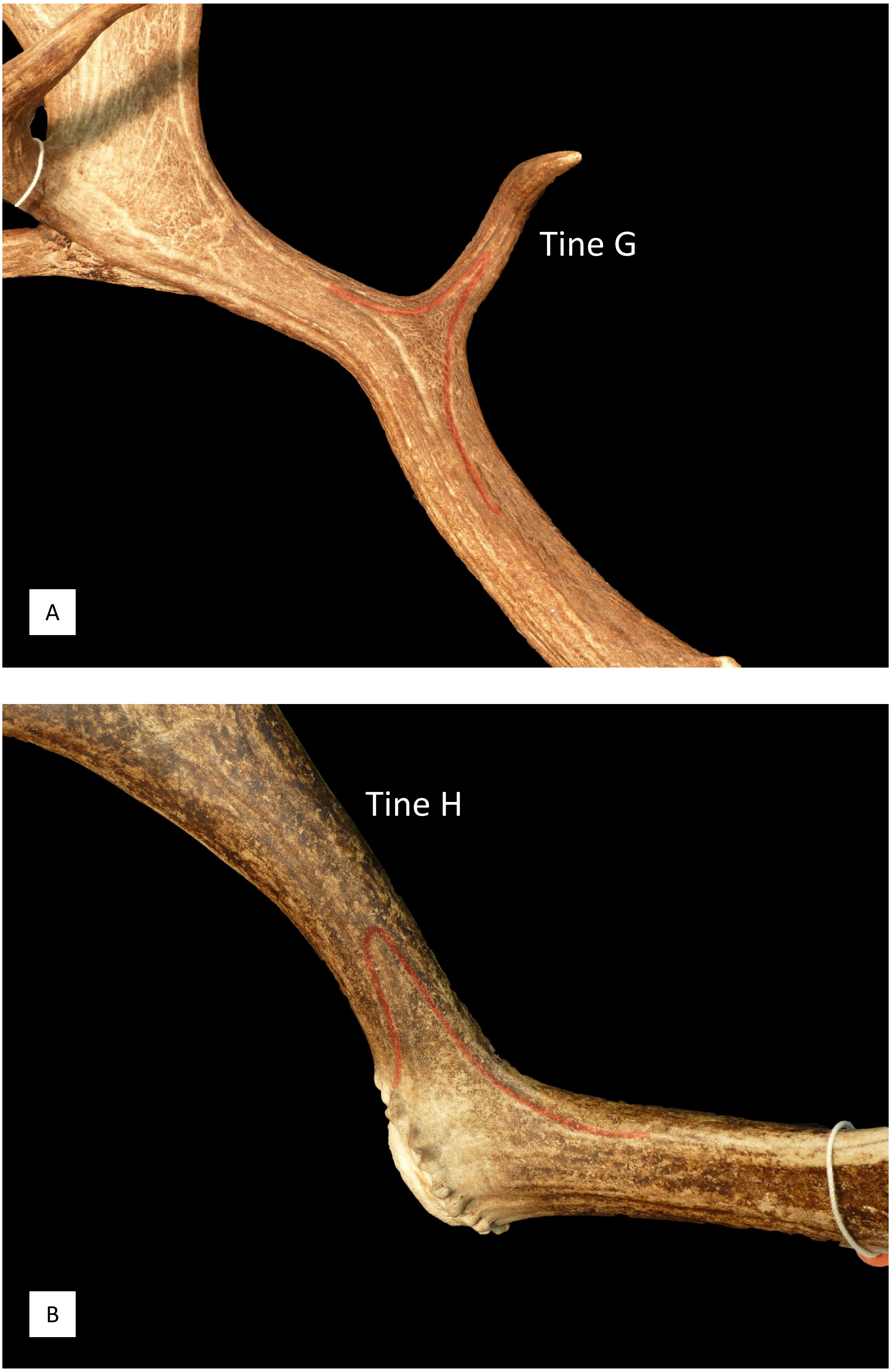

Figure 6 Example of antler grooves turning back through a tine (shown by red lines) . A) *Dama dama,* KUGM-RM107, left antler. B) *Rangifer tarandus,* KUGM-RM52, left antler.
