# Supplementary material for: A new viewpoint on antlers reveals the evolutionary history of deer (Cervidae, Mammalia)
Source: Sci Rep. 2020 Jun 2;10:8910. doi: 10.1038/s41598-020-64555-7 (PMC7265483; doi:10.1038/s41598-020-64555-7)

A new viewpoint on antlers reveals the evolutionary history of deer (Cervidae, Mammalia)

Yuusuke Samejima & Hiroshige Matsuoka

Supplementary Information 2

Note about the Diagrams

・The large circle represents the burr cross section.
・The dots are the branching direction of the tines.
・The short lines orthogonal to the circle are the positions of the forks.
・The painted areas are the zones of the tines.
・Inside the circle, the branching hierarchical order among the tines is represented.
・The central dot is the base of antler.

The way of drawing the diagram showing branching structure

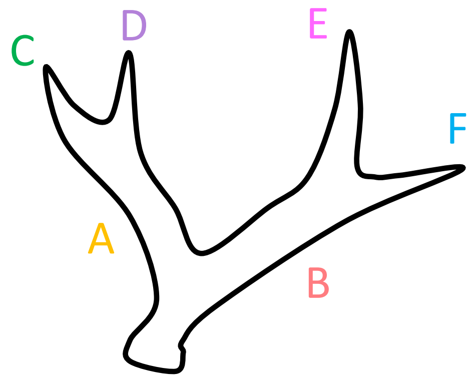

The following four diagrams all represent the same branching structure.

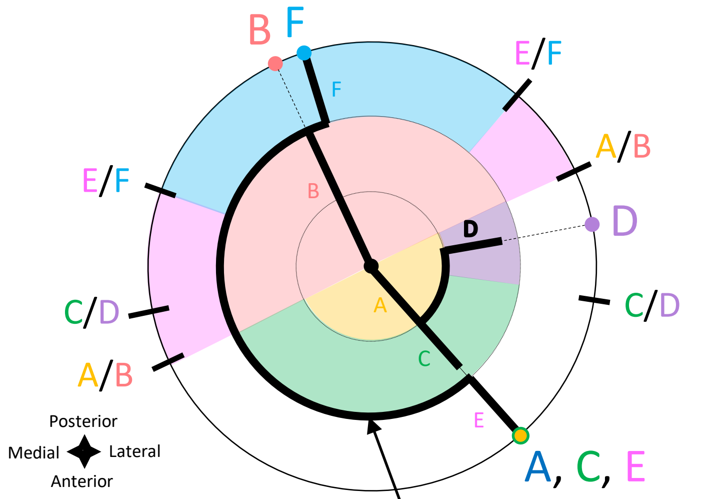

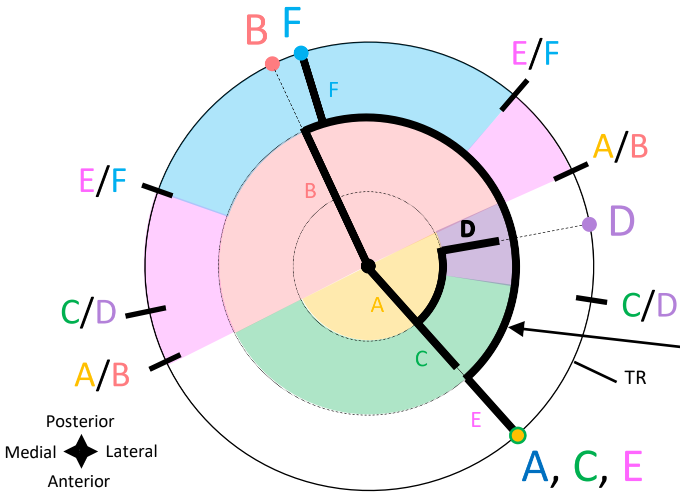

A concentric partial circle connecting branching tines can be rounded from either side.

If the distal portions of the two tines overlap, either tine can be written inside (or outside).

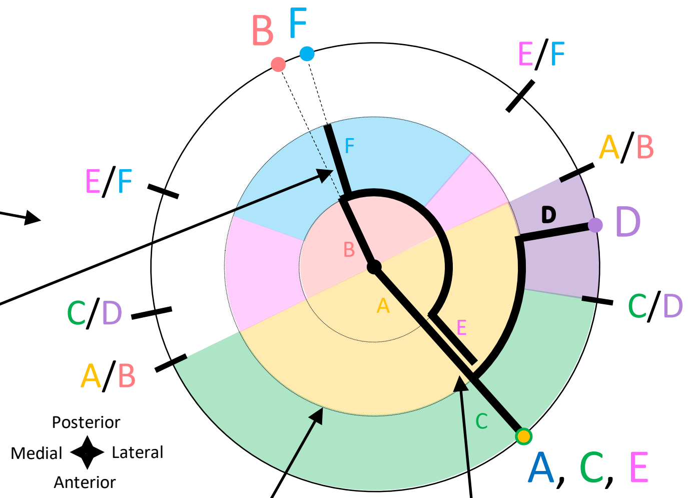

The length of a branch has no meaning. However, the color is painted for the length.

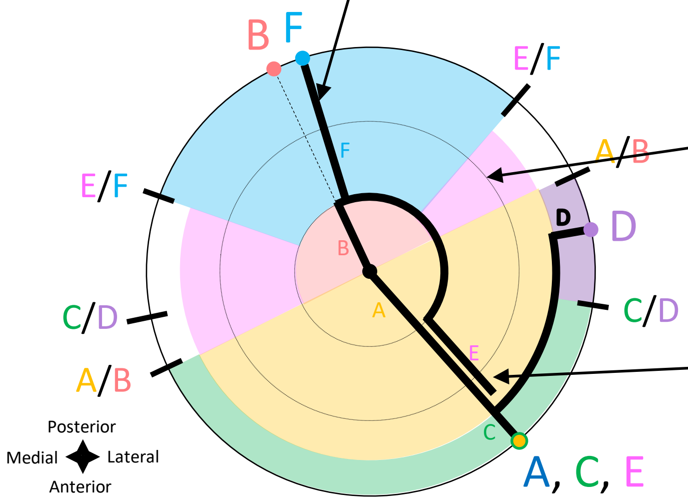

Concentric circles of dotted lines are for making the diagram easier to see, and have no meaning.

If the directions of a tine is at the same as that of another tine, write it with a slight shift in parallel.

<u>The way of drawing the diagram showing branching structure</u>

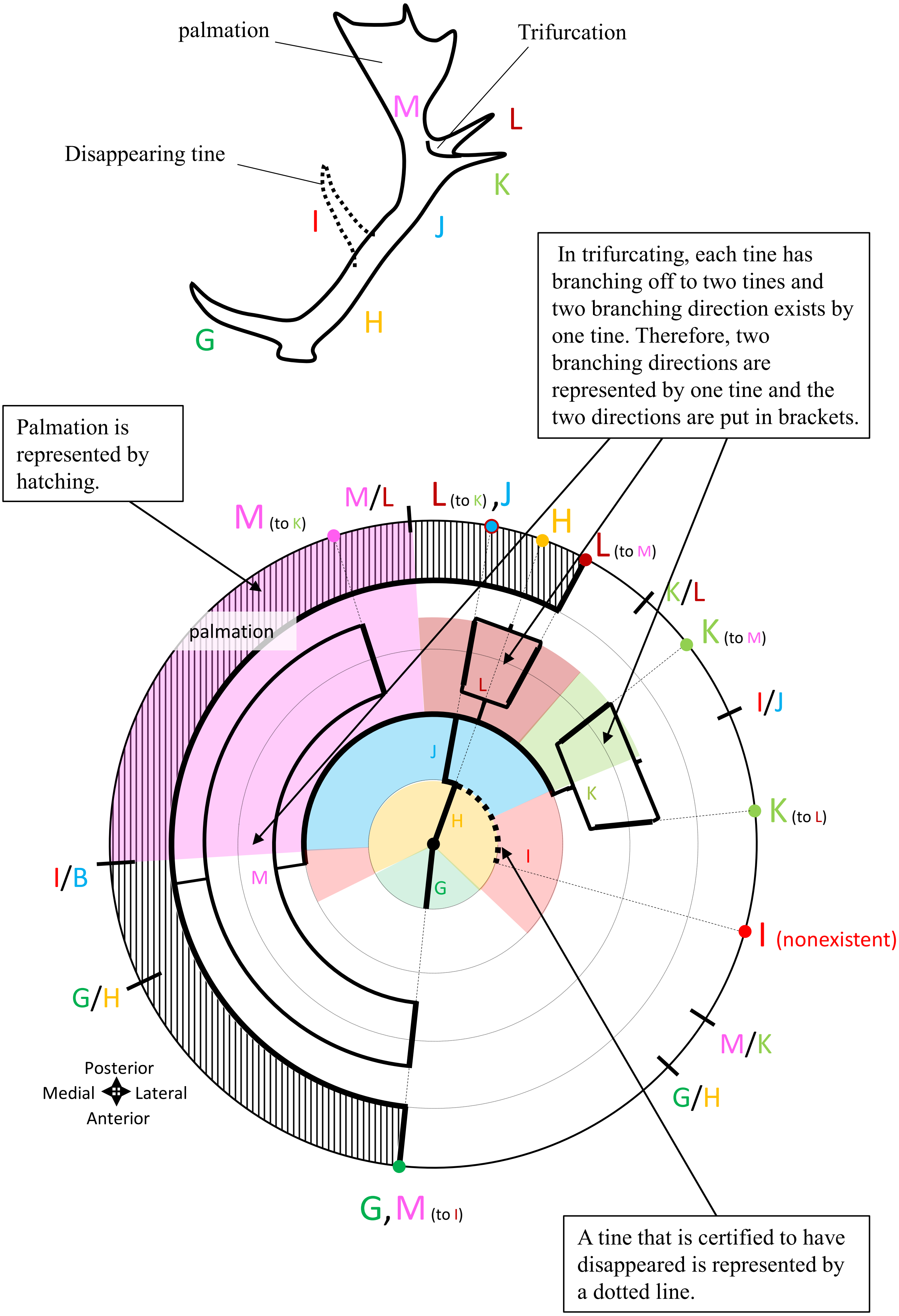

Supplement: Supplementary file 2 — Supplementary information 2 - The way of drawing the diagram showing branching structure. [file 41598_2020_64555_MOESM2_ESM.pdf]
