# Supplementary material for: A new viewpoint on antlers reveals the evolutionary history of deer (Cervidae, Mammalia)
Source: Sci Rep. 2020 Jun 2;10:8910. doi: 10.1038/s41598-020-64555-7 (PMC7265483; doi:10.1038/s41598-020-64555-7)
Supplement: Supplementary file 3 — Supplementary information 3 - Various types of antlers observed in this study. [file 41598_2020_64555_MOESM3_ESM.pdf]

A new point of view on antlers reveals the evolutionary history of deer (Cervidae, Mammalia)

Yuusuke Samejima & Hiroshige Matsuoka

Supplementary Information 4

Explanation of homologous elements

Brow tine (B): The anterior branching tine at the first fork in many species. The counterpart is the lower beam. Branching direction is slightly medial to the supraorbital ridge on the pedicle and slightly lateral to impression of the frontal branch of superficial temporal artery (medial branch). *Odocoileus Virginianus, Odocoileus hemionus, Mazama americana, Blastocerus dichotomus* and *Alces alces* usually don't have Brow tine.

Brow-inner tine (BI) and Brow-outer tine (BO): Distal tines of the brow tine. The BI branches medially and the BO branches laterally. The branching direction of the BO is lateral to the supraorbital ridge on the pedicle and anterior to the temporal ridge on pedicle. Neither BI nor BO overlap with Lower beam. Observed always in *Elaphurus davidianus.*

Brow process (BWP): A process coming out dorsally on the brow tine. Sometimes observed in Cervini species.

Brow-medial tine (BM): Branching medially from the brow tine above the fork of the brow tine and Lower beam. Sometimes observed in *Rusa unicolor* and *Rusa mariaana*.

Brow-anterior tines (BAs): Multiple tines branching forward from the brow tine. The branching direction is slightly lateral to the brow tine. Observed often in *Rangifer tarandus*, and rarely in *Odocoileus virginianus*. In *Rangifer tarandus*, often palmate with the brow tine.

Lower beam (L): The posterior branch at the first fork in most species. The counterpart is the brow tine. The fork with the brow tine (B/L) on the lateral side is anterior to the temporal ridge on pedicle and extended on the pedicle to the superior-posterior margin of the orbit. Observed in most species.

Bez tine (Z): Branching a little laterally on the lower beam. Often being nearly trifurcated with the brow tine and lower beam. Usually observed in *Cervus canadensis* and *Cervus elaphus*, and sometimes observed in *Dama dama* and *Rusa unicolor*.

Beam process (BMP): A process coming out dorsally on the lower beam nearly at the fork of the brow tine and the lower beam. Observed sometimes in *Dama dama*, *Panolia eldii*, *Rusa* and *Cervus*.

Post beam process (PBP): A process branching dorsally on the lower beam posterior to the BMP. A little medial to the BMP. Observed in *Dama.*

Sub-medial tine (SM): Branching medially from the lower beam. The branching direction is at the boundary of the supraorbital and temporal nerve areas. The fork with the lower beam (L/SM) is quite medial to the branching direction of the higher beam. Observed rarely in *Rusa*.

Medial tine (M): Branching medially from the the lower beam. The branching direction on the burr is at the boundary of the supraorbital and temporal nerve areas. The fork with the lower beam (L/M) is just at the branching direction of the lower beam. Observed always in *Rucervus eldii* and sometimes in *Elaphurus davidianus*.

Pre-trez tines (PTs): Multiple tiny tines branching in the same direction of the trez tine on the lower beam. Observed often in *Elaphurus davidianus*.

Pre-cacuminal tines (PCs): Multiple tiny tines branching in the same direction of the higher beam on the lower beam. Observed often in *Elaphurus davidianus* and *Rucervus eldii.*

Cacuminal tine (C): Branching in the same direction of the higher beam on the lower beam. The fork with the lower beam (L/C) is slightly medial to the medial tine. Observed often in *Rucervus eldii.*

Trez-beam process (TBP): A process between the trez tine and the higher beam at the fork. Observed often in *Rusa* and sometimes in *Dama* and *Cervus nippon.*

Trez tine (T): Branching anterolaterally at the end of the lower beam. The counterpart is the higher beam. The branching direction is posterior to the temporal ridge on the pedicle. The fork with the higher beam (T/H) on the lateral side, going down through the burr on the pedicle along the burr, reaches the lateral margin of the origin of the frontoscutular muscle and the posterior margin of the orbit. Observed widely in Cervini except *Rucervus*, *Panolia eldii* and normal *Elaphurus davidianus*.

Higher beam (H): Branching posteromedially at the end of the lower beam. The counterpart is The trez tine. The branching direction, going down through the burr on the pedicle along the burr, reaches the posterior extremity of the origin of the interscutular muscle. Observed widely in Cervini except *Rucervus duvaucelii* and *Rucervus schomburgki*.

Guard tine (G): Branching downward from the higher beam. Observed usually in adult *Dama dama*.

Crown-inner tine (CI): Branching medially at the end of the higher beam. Forming trifurcation (called "crown") with the crown-outer tine and the crown-tail tine. Observed always in *Dama dama, Cervus nippon* and *Cervus canadensis,* often in *Cervus elaphus* and rarely in *Rusa unicolor*.

Crown-outer tine (CO): Branching laterally at the end of the higher beam. Forming trifurcation with the crown-inner tine and the crown-tail tine. Observed usually in *Cervus nippon and Cervus elaphus,* sometimes in *Cervus canadensis* and rarely in *Rusa unicolor* and *Dama dama*.

Crown-back tine (CB): Branching backward at the end of the higher beam. Forming trifurcation with the crown-inner tine and the crown-outer tine. Observed always in *Cervus canadensis, in Cervus elaphus and in Dama dama,* and sometimes in *Cervus nippon* and *Rusa unicolor*. In *Dama dama*, palmate with the crown-inner tine and the guard tine.

Crown-back-inner tine (CBI): Branching medially at the end of the back-tail tine. Forming trifurcation with the crown-back-outer tine and the crown-back-back tine. Observed often in *Cervus elaphus* and *Dama dama,* always in *Cervus canadensis* and rarely in *Cervus nippon.*

Crown-back-outer tine (CBO): Branching laterally at the end of the back-tail tine. Forming

trifurcation with the crown-back-back tine and the crown-back-back tine. Observed often in *Cervus elaphus*.

Crown-back-back tine (CBB): Branching medially at the end of the back-tail tine. Forming a trifurcation with the crown-back-back tine and the crown-back-outer tine. Observed always in *Cervus canadensis,* often in *Cervus elaphus* and *Dama dama,* and rarely in *Cervus nippon*.

Vertical beam (V), Back beam (B), and distal tines: The vertical beam branches anterolaterally and The back beam branches posteromedially at the second fork in *Rucervus schomburgki and Rucervus duvaucelii*. The branching structure of the second fork of these species is clearly different from the trez tine and higher beam bifurcation. Therefore, the vertical beam and back beam are not homologous to the trez tine and higher beam. Either of the V or B could be included in the lower beam, but more analysis is necessary.

Frontal tine (F): Branching forward slightly laterally at the end of the lower beam. The counterpart of the upper beam. The branching direction is anterior to the temporal ridge on the pedicle and slightly anterior to the fork of the brow tine and lower beam (B/L) on the lateral side. The fork with the upper beam (F/U) on the lateral side, going down through the burr on the pedicle along the burr, reaches the lateral margin of the origin of the frontoscutular muscle and the posterior margin of the orbit. F/U on the medial side is at the same position of the fork of the brow tine and lower beam (B/L) on the medial side. Observed in Capreolinae, usually in *Rangifer tarandus* and *Capreolus*.

Frontal-inner tine (FI): Branching medially from the frontal tine. Observed sometimes in *Capreolus pygargus*.

Frontal-posterior tines (FPs): Generic name of multiple tines branching upward in the same direction from the frontal tine. Observed only in *Rangifer tarandus*. The determination of homology of each one is a future task.

Frontal-outer tine group

Frontal-outer-1st tine (FO1): Branching upward laterally on the frontal tine. Observed in *Blastocerus dichotomus* and mutant *Odocoileus virginianus.*

Frontal-outer-2nd tine (FO2): Branching upward laterally on the frontal tine after the FO1. Observed in mutant *Odocoileus virginianus.*

Upper beam (U): Branching at the end of the lower beam. The counterpart of the frontal tine. The branching direction is slightly medial to the boundary of the supraorbital and temporal nerve areas. Observed widely in Capreolinae.

Post-frontal tine (PF): Branching upward from the upper beam. Observed sometimes in *Capreolus pygargus* and rarely in *Rangifer tarandus*.

Rear tine (R): Branching backward from the upper beam. The branching direction is on the burr is at

the boundary of the supraorbital and temporal nerve areas. Observed in Odocoileini. Often called the “subbasal snag” in *Odocoileus.*

Terminal-anterior tine (TA): Branching anteriorly at the end of the upper beam. The counterpart of the terminal-posterior tine. The branching direction is lateral to the supraorbital ridge on the pedicle. The fork with the terminal-posterior tine (TA/TP) on the lateral side is at the same position of M/R on the lateral side. Observed in *Rangifer tarandus* and *Alece alces*.

Terminal-posterior tine (TP): Branching posteriorly at the end of the upper beam. The counterpart of the terminal-posterior tine. Observed in *Alces alces* and *Rangifer taranus*. In *Alces alces*, palmate in many cases.

Terminal-posterior-anterior tines (TPAs): The generic name of multiple tines branching upward in the same direction on the terminal-posterior tine. Observed in *Rangifer tarandus*. Determination of homology of each one is a future task.

Upper tine group: Observed only in *Odocoileus.*

Upper-1st tine (U1): Branching upward first on the upper beam in *Odocoileus virginianus* and *Odocoileus hemionus*.

Upper-1st-anterior tine (U1A): Branching anteriorly at the end of the U1 in *Odocoileus hemionus*.

Upper-1st-posterior tine (U1P): Branching posteriorly at the end of the U1 in *Odocoileus hemionus*.

Upper-2nd tine (U2): Branching upward second on the upper beam in *Odocoileus virginianus* and *Odocoileus hemionus*.

Upper-3rd tine (U3): Branching upward third on the upper beam in *Odocoileus virginianus*.

Supra-burr process (SBP): A process coming out right above the burr. Sometimes observed in *Odocoileus*.
