# Supplementary material for: A new viewpoint on antlers reveals the evolutionary history of deer (Cervidae, Mammalia)
Source: Sci Rep. 2020 Jun 2;10:8910. doi: 10.1038/s41598-020-64555-7 (PMC7265483; doi:10.1038/s41598-020-64555-7)
Supplement: Supplementary file 4 — Supplementary information 4 - Explanation of homologous elements (tines, beams and processes). [file 41598_2020_64555_MOESM4_ESM.pdf]

Yuusuke Samejima & Hiroshige Matsuoka

Supplementary Information 5

Table 1 Comparison with a1, p1, a2, p2… of Pocock (1933).

	Base of antler	Brow tine	Lower beam	Bez tine	Medial tine	Vertical beam	Back beam and its distal tines	Trez tine	Higher beam	Guard tine	Crown-outer tine	Crown-inner tine	Crown-back tine	Crown-back-outer tine	Crown-back-inner tine	Crown-back-back tine	Frontal tine	Upper beam	rear tine	Terminal-anterior tine	Terminal-posterior tine	Upper-1st tine	Upper-2nd tine	Upper-3rd tine
Cervus nippon	b	a1	p1					a2	p2		p3	a3												
Cervus canadensis	b	a1	p1	x				a2	p2			a3	p3		p4	a4								
Cervus elaphus	b	a1	p1	x				a2	p2		a3/p3	a3	p3/a3		p4	a4								
Rusa unicolor	b	a1	p1					a2	p2															
Rusa timoensis	b	a1	p1					a2	p2															
Rusa mariaana	b	a1	p1					a2	p2															
Panolia eldii	b	a1	p1,a2		p2																			
Elaphurus davidianus	b	a1	p1						p1															
Dama dama	b	a1	p1					a2	p2,a3	p3														
Axis axis	b	a1	p1					a2	p2															
Axis porcinus	b	a1	p1					a2	p2															
Rucervus schomburgki	b	a1	p1			a2	p3																	
Rucervus duvaucelii	b	a1	p1			a2	p3																	
Muntiacus muntjak	b	a1	p1																					
Muntiac reevisi	b	a1	p1																					
Elaphodus cephalophus	b																							
Hydropotes intermis																								
Capreolus capreolus	b	a1	p1														a2	p2						
Capreolus pygargus	b	a1	p1														a2	p2						
Alces alces	b																			a1	p1			
Rangifer tarandus	b	a1	p1														a2	p2,a3	p3					
Odocoileus virgirianus	b																	p1,a2	a1			p2		
Odocoileus hemionus	b																	p1,a2	a1			p2		
Mazama americana	b																							
Blastocerus dichotomus	b																a1	p1,a2	p2					

Table 2 Comparison with previous works about homology and terminology of the elements.

		B<br>**Brow tine**	L<br>**Lower beam**	Z<br>**Bez tine**	T<br>**Trez tine**	H<br>**Higher beam**	G<br>**Guard tine**	CI<br>**Crown-inner tine**	CO<br>**Crown-outer tine**	CB<br>**Crown-back tine**	F<br>**Frontal tine**	U<br>**Upper Beam**	R<br>**Rear tine**	TA/TP<br>**Terminal-anterior/posteior tines**
Cervus elaphus	Mooney (1952)	Brow tine	Beam	Bez tine	Tres tine	Beam	—		Cornet		—	—	—	—
	Muir *et al.* (1987)	Brow tine	Main shaft	Bez tine	Trez tine	Main shaft	—		Royal tines		—	—	—	—
	Fennessy *et al.* (1992)	Brow tine	Main Beam	Bez tine	Trez tine	Main Beam	—		Royal tines		—	—	—	—
	Mateos *et al.* (2008)	Brow tine			Trez tine		—		Crown tines		—	—	—	—
	Salmerón (2014)	Eye tine	Main Beam	Bez tine	Trez tine	Main beam	—		Crown		—	—	—	—
	Lefebvre *et.al.* (2016)	Brow tine	BeamA2	Bez tine	Trez tine	BeamB	—		Crown		—	—	—	—
Cervus canadensis	Croitor & Obada (2018)	Brow tine	Beam	Bez tine	Trez tine	Beam	—		Crown		—	—	—	—
Cervus nippon	Mooney (1952)	Brow tine	Beam	—	Tres tine	Beam	—		Cornet		—	—	—	—
	Feldharmer (1980)	Brow tine		—	Tray tine		—				—	—	—	—
	Hayden *et al.* (1994)	Brow tine	Main Beam	—	Trez tine	Main beam	—				—	—	—	—
Rusa	Grubb (1990)	Brow tine	Beam		Front-outer tine	Back inner tine	—				—	—	—	—
Rusa unicolor	Leslie (2011)	Brow tine			Anterolateral tine	Posteromedial tine	—				—	—	—	—
Dama dama	Chapman (1975)	Brow tine	Beam		Trez tine	Beam		Spellers		Spellers	—	—	—	—
	Feldharmer *et al.* (1988)	Brow tine		(Bay tine)	Tray tine						—	—	—	—
	Lister *et al.* (2005)	Brow tine	Beam		Middle tine	Beam	Back tine				—	—	—	—
	Ciuti *et al.* (2011)	Brow tine	Beam		Trez tine	Beam	Last speller	Spellers		Spellers	—	—	—	—
	Australian Deer Society	Brow tine	Beam	Bez tine	Trez tine	Beam	Guard tine	Palmation			—	—	—	—
Elaphurus davidianus	Walton & Hosey (1984)	Brow tine	Beam tine	—		—	—	—	—	—	—	—	—	—
Axis	Grubb (1990)	Brow tine	Beam	—	Front-outer tine	Back inner tine	—	—	—	—	—	—	—	—
Rangifer tarandus	Banfield (1961)	Brow tine	Beam	—	—	—	—	—	—	—	Bez tine	Beam	Posterior tine	Terminal tines
	Markusson & Folstad (1997)	Brow tine	Main Beam	—	—	—	—	—	—	—	Frontal tine	Main beam	Rear tine	
	Lefebvre *et.al* (2016)	Brow tine	Beam A	—	—	—	—	—	—	—	Bez tine	Beam A2、BeamC		
Odocoileus	Allen & Wallom (1984)		Beam	—	—	—	—	—	—	—		Beam	Subbasal snag	—
	Gustafson (1985)		Beam	—	—	—	—	—	—	—		Beam	Basal tine	—
