# Supplementary material for: A new viewpoint on antlers reveals the evolutionary history of deer (Cervidae, Mammalia)
Source: Sci Rep. 2020 Jun 2;10:8910. doi: 10.1038/s41598-020-64555-7 (PMC7265483; doi:10.1038/s41598-020-64555-7)
Supplement: Supplementary file 5 — Supplementary information 5 - Comparison of the elements with Pocock (1933) and other previous works. [file 41598_2020_64555_MOESM5_ESM.pdf]

A new viewpoint on antlers reveals the evolutionary history of deer (Cervidae, Mammaila)

Yuusuke Samejima & Hiroshige Matsuoka

Supplementary Information 6

Matrix of existence of the elements of all the specimens observed

Annotation

All specimens (except some of *Rangifer tarandus*) are males.

Abbreviations of the idices with the relation to the skull

SR	Supraorbital ridge on the pedicle
IFST	Impression of the frontal branch of the superficial temporal artery (medial branch)
TR	Temporal ridge on the pedicle
BN	Boundery of supraorbital and temporal nerve area

Symbols and numbers

○	Applicable or Existent
×	Nonexistent
(blank)	Unconfermed or Nothing to describe in particular
―	Noncoverd
1	Existent
0	Nonexistent
?	Unknown or Unsolved

Abbreviations of the museums and institutes

CBM	Natural History Museum and Institute, Chiba
HUBG	Botanic Garden, Hokkaido University
KPM	Kanagawa Prefecture Museum of Natural History
KUGM	Department of Geology and Mineralogy, Facility of Science, Kyoto University
KUZ	The Kyoto University Museum
LBM	Lake Biwa Museum
NSMT	National Museum of Nature and Science
OMNH	Osaka Museum of Natural History
SMZ	Sapporo Maruyama Zoo
TG	Taga Town Museum
TUM	The Tohoku University Museum

Cervus nippon (Mature antlers)

Museum or Institute	Number	Floor reposited	Subspecies	Locality/Note	Specimen type	Right	Left	SR	IFST	TR	BN	Base of antler	Brow tine	Brow process	Lower beam	Beam process	Trez tine	Trez-beam process	Higher beam	Crown-outer tine	Crown-inner tine	Crown-back tine	Crown-Crown-inner tine	Crown-back-back tine
KUGM	RM135			Cut antler	Antler	○		—	—	—	—	1	1	0	1	1	1	0	1	1	1	1	0	0
KUGM	RM124				Skull、Antler	○		○	×	○	○	1	1	0	1	1	1	0	1	1	1	0	0	0
							○	○	×	○	○	1	1	0	1	0	1	0	1	1	0	1	0	0
KUGM	RM127				Antler (tip)	○		—	—	—	—	1	?	?	1	?	?	?	1	1	1	1	1	1
KUGM	RM125			Shed antler	Antler		○	—	—	—	—	1	1	0	1	1	0	0	1	1	1	1	1	1
KUGM	RM126		*C.n.yesoensis*	Mikasa, Hokkaido	Part of skull、Antler	○		○	○	○	○	1	1	0	1	0	1	0	1	1	1	1	0	0
KUGM	RM001				Skull、Antler	○		○	×	○	○	1	1	0	1	0	1	0	1	1	1	0	0	0
							○	○	×	○	○	1	1	0	1	0	1	0	1	1	1	0	0	0
KUGM	RM005		*C.n.aplodontus*	Tamba	Skull、Antler	○		○	×	○	○	1	1	0	1	0	1	0	1	1	1	0	0	0
							○	○	×	○	○	1	1	0	1	0	1	0	1	1	1	0	0	0
KUGM	RM003		*C.n.aplodontus*	Yakuno, Kyoto	Skull、Antler	○		○	×	○	○	1	1	0	1	0	1	0	1	1	1	0	0	0
							○	○	○	○	○	1	1	0	1	0	1	0	1	1	1	0	0	0
KUGM	RM007		*C.n.aplodontus*	Kiyosumi, Chiba	Skull、Antler	○		○	×	○	○	1	1	0	1	0	1	0	1	1	1	0	0	0
KUGM	RM091				Skull、Antler	○		?	?	?	?	1	1	0	1	0	1	0	1	1	1	0	0	0
							○	?	?	?	?	1	1	0	1	0	1	0	1	0	0	0	0	0
KUGM	RM004		*C.n.yesoensis*	Hidaka, Hokkaido	Skull、Antler	○		○	○	○	○	1	1	0	1	1	1	0	1	1	1	0	0	0
							○	○	○	○	○	1	1	0	1	1	1	0	1	1	1	0	0	0
KUGM	RM017		*C.n.yesoensis*	Nemuro, Hokkaido	Skull、Antler	○		○	○	○	○	1	1	0	1	0	1	0	1	1	1	1	0	0
							○	○	○	○	○	1	1	0	1	0	1	0	1	1	1	1	0	0
KUGM	RM104				Skull、Antler	○		○	○	○	○	1	1	0	1	0	1	0	1	1	1	0	0	0
							○	○	○	○	○	1	1	0	1	0	1	0	1	1	1	0	0	0
KUGM	RM018		*C.n.nippon*	Kagawa	Skull、Antler	○		○	×	○	○	1	1	0	1	0	1	0	1	1	1	0	0	0
							○	○	×	○	○	1	1	0	1	1	1	0	1	1	1	0	0	0
KUGM	RM016		*C.n.yesoensis*	Nemuro, Hokkaido	Skull、Antler	○		○	○	○	○	1	1	0	1	1	1	0	1	1	1	1	0	0
							○	○	○	○	○	1	1	0	1	1	1	0	1	1	1	1	0	0
KUGM	RM145				Antler	○						1	1	0	1	1	0	0	1	1	1	0	0	0
							○					1	1	0	1	1	1	0	1	1	1	0	0	0
HUBG	10278		*C.n.taiouanus*	Taiwan	Antler	○						1	1	0	1	0	1	0	1	1	1	1	0	0
							○					1	1	0	1	0	1	0	1	1	1	1	0	0
HUBG	13037		*C.n.taiouanus*	Taiwan	Antler	○						1	1	0	1	0	1	0	1	1	1	1	0	0
							○					1	1	0	1	0	1	0	1	?	?	?	?	?
HUBG	10279			China	Antler	○						1	1	0	1	0	1	0	1	1	1	0	0	0
							○					1	1	0	1	0	1	0	1	1	1	0	0	0
NSMT	M43312	1F	*C.n.hortulorum*	Ussuri	Antler	○						1	1	0	1	0	1	0	1	1	1	0	0	0
							○					1	1	0	1	0	1	0	1	1	1	0	0	0
NSMT	M43307	1F	*C.n.yesoensis*	Hokkaido	Antler	○						1	1	0	1	1	1	0	1	1	1	1	0	0
							○					1	1	0	1	1	1	0	1	1	1	1	0	0
NSMT	M43313	1F	*C.n.taiouanus*	Taiwan	Antler	○						1	1	0	1	0	1	0	1	1	1	0	0	0
							○					1	1	0	1	0	1	0	1	1	1	0	0	0
NSMT	M21192	1F	*C.n.taiouanus*	Taiwan	Antler・Skull		○					1	0	0	1	0	0	0	1	0	1	1	1	1
NSMT	M01158	1F	*C.n.taiouanus*	Taiwan	Antler	○						1	1	0	1	0	1	0	1	1	1	0	0	0
							○					1	1	0	1	0	1	0	1	1	1	0	0	0
NSMT	M01156	1F	*C.n.mantchuricus*	Mauchu	Antler	○						1	1	0	1	0	1	0	1	1	1	0	0	0
							○					1	1	0	1	0	1	0	1	1	1	0	0	0
NSMT	M43306	1F	*C.n.aplodontus*	Shimotsuke (Tochigi)	Antler	○						1	1	0	1	1	1	0	1	1	1	0	0	0
							○					1	1	0	1	1	1	0	1	1	1	0	0	0
NSMT	M43317	1F	*C.n.aplodontus*	Iwami (Shimane)	Antler	○						1	1	0	1	0	1	0	1	1	1	0	0	0
							○					1	1	0	1	0	1	0	1	1	1	0	0	0
NSMT	M43321	1F	*C.n.nippon*	Iyo (Ehime)	Antler	○						1	1	0	1	0	1	0	1	1	1	0	0	0
							○					1	1	0	1	0	1	0	1	1	1	0	0	0

Colored tines (two out of Crown-inner tine, Crown-outer tine, and Crown-back tine) join together more distally than the other of the three.

Cervus nippon (mature antlers)(continued)

Museum or Institute	Number	Floor reposited	Subspecies	Locality/Note	Specimen type	Right	Left	SR	FBSTAMI	TR	BN	Base of antler	Brow tine	Brow process	Lower beam	Beam process	Trez tine	Trez-beam process	Higher beam	Crown-outer tine	Crown-inner tine	Crown-back tine	Crown-Crown-inner tine	Crown-back-back tine
NSMT	M43322	1F	*C.n.aplodontus*	Izu (Shizuoka)	Antler	○						1	1	0	1	0	1	0	1	1	1	0	0	0
							○					1	1	0	1	0	1	0	1	1	1	0	0	0
NSMT	M43321	1F	*C.n.aplodontus*	Yamashiro (Kyoto)	Antler	○						1	1	0	1	0	1	0	1	1	1	0	0	0
							○					1	1	0	1	0	1	0	1	1	1	0	0	0
NSMT	M43316	1F	*C.n.aplodontus*	Shimotsuke (Tochigi)	Antler	○						1	1	0	1	0	1	0	1	1	1	0	0	0
							○					1	1	0	1	0	1	0	1	**1**	1	**1**	0	0
NSMT	M43314	1F	*C.n.nippon*	Kagoshima	Antler	○						1	1	0	1	0	1	0	1	1	1	0	0	0
							○					1	1	0	1	0	1	0	1	1	1	0	0	0
NSMT	M47189	1F	*C.nippon.yakushimae*	Yakushima	Skull・Antler	○						1	1	0	1	0	1	0	1	0	0	0	0	0
							○					1	1	0	1	0	1	0	1	0	0	0	0	0
NSMT	M14306	1F	*C.n.pulchellus*	Tsushima	Skull・Antler	○						1	1	0	1	0	1	0	1	1	1	0	0	0
							○					1	1	0	1	0	1	0	1	1	1	0	0	0
NSMT	M13693	1F	*C.n.pulchellus*	Tsushima	Skull・Antler	○						1	1	0	1	0	1	0	1	1	1	0	0	0
							○					1	1	0	1	0	1	0	1	1	1	0	0	0
NSMT	No number	7F			Stuffing	○		—	—	—	—	1	1	0	1	0	1	0	1	1	1	0	0	0
							○	—	—	—	—	1	1	1	1	0	1	0	1	1	1	0	0	0
NSMT	No number	7F		Tsushima?	Stuffing	○		—	—	—	—	1	1	0	1	0	1	0	1	1	1	0	0	0
							○	—	—	—	—	1	1	0	1	0	1	0	1	1	1	0	0	0
NSMT	M42803	7F	*C.n.yesoensis*	Yubari, Hokkaido	Stuffing	○		—	—	—	—	1	1	1	1	0	1	0	1	1	1	0	0	0
							○	—	—	—	—	1	1	1	1	0	1	0	1	1	1	0	0	0
NSMT	M37524	7F	*C.n.yesoensis*	Hidaka, Hokkaido	Stuffing	○		—	—	—	—	1	1	0	1	0	1	0	1	1	1	0	0	0
							○	—	—	—	—	1	1	0	1	0	1	0	1	1	1	0	0	0
NSMT	M37523	7F	*C.n.yesoensis*	Hidaka, Hokkaido	Stuffing	○		—	—	—	—	1	1	0	1	0	1	0	1	**1**	1	**1**	0	0
							○	—	—	—	—	1	1	0	1	0	1	0	1	**1**	**1**	1	0	0
NSMT	M50096	7F		Nikko, Tochigi	Stuffing	○		—	—	—	—	1	1	0	1	0	1	0	1	1	1	0	0	0
							○	—	—	—	—	1	1	0	1	0	1	0	1	1	1	0	0	0
NSMT	M49973	7F			Stuffing	○		—	—	—	—	1	1	0	1	0	1	0	1	0	0	0	0	0
							○	—	—	—	—	1	1	0	1	0	1	0	1	1	1	0	0	0
CBM	ZZ0001202		*C.n.nippon*	Kimitsu, Chiba	Antler・Skull	○						1	1	0	1	0	1	0	1	0	0	0	0	0
							○					1	1	0	1	0	1	0	1	0	0	0	0	0
CBM	ZZ0001182		*C.n.nippon*	Amatsukominato, Chiba	Antler・Skull	○						1	1	0	1	0	1	0	1	1	1	0	0	0
							○					1	1	0	1	0	1	0	1	1	1	0	0	0
CBM	No number	Displayed			Stuffing	○		—	—	—	—	1	1	0	1	0	1	0	1	**1**	1	**1**	0	0
							○	—	—	—	—	1	1	0	1	0	1	0	1	1	1	1	1	1
CBM	ZZ0000828				Complete skeleton	○						1	1	0	1	0	1	0	1	1	1	0	0	0
							○					1	1	0	1	1	1	0	1	1	1	0	0	0
CBM	ZZ0000618		*C.n.nippon*	Amatsukominato, Chiba	Antler・Skull	○						1	1	0	1	0	1	0	1	1	1	0	0	0
							○					1	1	0	1	0	1	0	1	1	1	0	0	0
CBM	ZZ0000238		*C.n.nippon*	Amatsukominato, Chiba	Antler・Skull	○						1	1	0	1	0	1	0	1	1	1	0	0	0
							○					1	1	0	1	0	1	0	1	1	1	0	0	0
CBM	ZZ0001116		*C.n.nippon*	Amatsukominato, Chiba	Stuffing	○		—	—	—	—	1	1	0	1	0	1	0	1	1	1	0	0	0
							○	—	—	—	—	1	1	0	1	0	1	0	1	1	1	0	0	0
CBM	ZZ0005971		*C.n.yesoensis*		Stuffing	○		—	—	—	—	1	1	0	1	0	1	0	1	0	0	0	0	0
							○	—	—	—	—	1	1	0	1	0	1	0	1	0	0	0	0	0
TZP	Displayed in *C.nippon* area		*C.n.yesoensis*	Yakushima	Antler	○		—	—	—	—	1	1	0	1	0	1	0	1	0	0	0	0	0
							○	—	—	—	—	1	1	0	1	0	1	0	1	0	0	0	0	0
TZP	Displayed in *C.nippon* area		*C.n.pulchellus*	Honshu	Antler	○		—	—	—	—	1	1	0	1	0	1	0	1	1	1	0	0	0
							○	—	—	—	—	1	1	0	1	0	1	0	1	1	1	0	0	0

Colored tines (two out of Crown-inner tine, Crown-outer tine, and Crown-back tine) join together more distally than the other of the three.

Cervus nippon (mature antlers)(continued)

Museum or Institute	Number	Floor reposited	Subspecies	Locality/Note	Specimen type	Right	Left	SR	FBSTAMI	TR	BN	Base of antler	Brow tine	Brow process	Lower beam	Beam process	Trez tine	Trez-beam process	Higher beam	Crown-outer tine	Crown-inner tine	Crown-back tine	Crown-Crown-inner tine	Crown-back-back tine
TZP	Displayed in *C.nippon* area		*C.n.yesoensis*	Hokkaido	Antler	○		—	—	—	—	1	1	0	1	0	1	0	1	1	1	0	0	0
							○	—	—	—	—	1	1	0	1	0	1	0	1	1	1	1	0	0
SMZ	4th from the right	1F Displayed			Skull+Antler	○		○		○		1	1	0	1	0	1	0	1	1	1	0	0	0
							○	○		○		1	1	0	1	0	1	0	1	1	1	0	0	0
SMZ	5th from the right	1F Displayed			Skull+Antler	○		○		○		1	1	0	1	0	1	0	1	1	1	1	0	0
							○	○		○		1	1	0	1	0	1	0	1	1	1	0	0	0
SMZ		2F Displayed	*C.n.yesoensis*	Hokkaido	Velvet antler	○		—	—	—	—	1	1	0	1	0	1	1	1	Before completion				
SMZ		2F Displayed	*C.n.aplodontus*	Honshu	Antler	○		—	—	—	—	1	1	0	1	1	1	0	1	1	1	0	0	0
SMZ		2F Displayed	*C.n.yesoensis*	Hokkaido	Antler+Skull	○						1	1	0	1	0	1	0	1	1	1	0	0	0
							○					1	1	0	1	0	1	0	1	1	1	0	0	0
SMZ		2F Displayed	*C.n.yesoensis*		Antler		○	—	—	—	—	1	1	0	1	0	1	0	1	1	1	0	0	0
								—	—	—	—	1	1	0	1	1	1	0	1	1	1	0	0	0
KUGM	RM213				Antler		○	—	—	—	—	1	1	0	1	0	1	0	1	1	1	0	0	0
											Sum	111	109	3	111	17	107	1	111	98	98	22	4	4
											Percentage	100%	98%	3%	100%	15%	96%	1%	100%	90%	90%	20%	4%	4%

Cervus nippon (Juvenile antlers)

Museum or Institute	Number	Floor reposited	Subspecies	Locality	Note	Specimen type	Right	Left	SR	IFST	TR	BN	Base of antler	Brow tine	Brow process	Lower beam	Beam process	Trez tine	Trez-beam process	Higher beam	Crown-outer tine	Crown-inner tine	Crown-back tine
KUGM	RM008		*C.n.yesoensis*		Juvenile (estimated 2-3 years old)	Skull、Antler	○		○	×	○	○	1	1	0	1	0	1	0	1	0	0	0
								○	○	×	○	○	1	1	0	1	0	1	0	1	0	0	0
KUGM	RM009		*C.n.yesoensis*		Juvenile (estimated 1-2 years old)	Skull、Antler	○		○	○	○	○	1	1	0	1	0	0	0	0	0	0	0
								○	○	×	○	○	1	1	0	1	0	0	0	0	0	0	0
KUGM	RM006		*C.n.yesoensis*		Juvenile (estimated 1-2 years old)	Skull、Antler	○		○	×	○	○	1	0	0	0	0	0	0	0	0	0	0
								○	○	×	○	○	1	0	0	0	0	0	0	0	0	0	0
NSMT	M23151				Juvenile	Stuffing	○						1	1	0	1	0	1	0	1	0	0	0
								○					1	1	0	1	0	1	0	1	0	0	0
NSMT	No number				Juvenile	Head stuffing	○						1	1	0	1	0	1	0	1	0	0	0
								○					1	1	0	1	0	1	0	1	0	0	0
CBM	ZZ0002037		*C.n.aplodontus*	Kimitsu, Chiba	Juvenile (2 years old)	Skull、Antler	○						1	1	0	1	0	1	0	1	1	1	0
								○					1	1	0	1	0	1	0	1	1	1	0
CBM	ZZ0001967		*C.n.aplodontus*	Kamogawa, Chiba	Juvenile (2 years old)	Skull、Antler	○						1	1	0	1	0	1	0	1	1	1	0
								○					1	1	0	1	0	1	0	1	0	0	0
CBM	ZZ0001776		*C.n.aplodontus*	Kamogawa, Chiba	Juvenile (2 years old)	Skull、Antler	○						1	1	0	1	0	1	0	1	0	0	0
								○					1	1	0	1	0	1	0	1	0	0	0
CBM	ZZ0001570		*C.n.aplodontus*	Kamogawa, Chiba	Juvenile (2 years old)	Skull、Antler	○						1	1	0	1	0	1	0	1	0	0	0
								○					1	1	0	1	0	1	0	1	0	0	0
CBM	ZZ0001775		*C.n.aplodontus*	Kamogawa, Chiba	Juvenile (2 years old)	Skull、Antler	○						1	0	0	1	0	0	0	1	0	0	0
								○					1	0	0	1	0	0	0	1	0	0	0
CBM	ZZ0001679		*C.n.aplodontus*	Kamogawa, Chiba	Juvenile (1 years old)	Skull、Antler	○						1	0	0	1?	0	0	0	0	0	0	0
								○					1	0	0	1?	0	0	0	0	0	0	0
CBM	ZZ0001516		*C.n.aplodontus*	Kamogawa, Chiba	Juvenile (2 years old)	Skull、Antler	○						1	0	0	1	0	0	0	1	0	0	0
								○					1	0	0	1	0	1	0	1	0	0	0
CBM	ZZ0001553		*C.n.aplodontus*	Kamogawa, Chiba	Juvenile (1 years old)	Skull、Antler	○						1	0	0	1?	0	0	0	0	0	0	0
								○					1	0	0	1?	0	0	0	0	0	0	0
CBM	ZZ0001313		*C.n.aplodontus*	Kamogawa, Chiba	Juvenile (2 years old)	Skull、Antler	○						1	0	0	1?	0	0	0	0	0	0	0
								○					1	0	0	1?	0	0	0	0	0	0	0
CBM	ZZ0001774		*C.n.aplodontus*	Kominato, Chiba	Juvenile (3 years old)	Skull、Antler	○						1	1	0	1	0	1	0	1	1	1	0
								○					1	1	0	1	0	1	0	1	1	1	0
SMZ	1st from the right	1F displayed	*C.n.yesoensis*		Juvenile (1-2 years old)	Skull、Antler	○		○	○	○		1	0	0	0	0	0	0	0	0	0	0
								○	○	○	○		1	0	0	0	0	0	0	0	0	0	0
SMZ	2nd from the right	1F displayed	*C.n.yesoensis*		Juvenile (2-3 years old)	Skull、Antler	○		○	○	○		1	0	0	1	0	1	0	1	1	1	0
								○	○	○	○		1	1	0	1	0	0	0	0	0	0	0
SMZ	3rd from the right	1F displayed	*C.n.yesoensis*		Juvenile (3-4 years old)	Skull、Antler	○		○	○	○		1	1	0	1	0	1	0	1	1	1	0
								○	○	○	○		1	1	0	1	1	0	0	1	1	1	0

Cervus canadensis

Museum or Institute	Number	Reposited room	Note	Specimen type	Right	Left	SR	IFST	TR	BN	Base of antler	Brow tine	Brow process	Lower beam	Beam process	Bez tine	Trez tine	Trez-beam process	Higher beam	Crown-outer tine	Crown-inner tine	Crown-back tine	Crown-back-inner tine	Crown-back-back tine
KUGM	RM109			Antler	○		—	—	—	—	1	1	0	1	0	1	1	0	1	0	1	1	1	1
						○	—	—	—	—	1	1	0	1	0	1	1	0	1	0	?	1	?	?
NSMT	M43324	1F		Antler,Skull	○		○	○	○	○	1	1	0	1	0	1	1	0	1	0	1	1	1	1
						○	○	○	○	○	1	1	0	1	0	1	1	0	1	0	1	1	1	1
NSMT	M03810	1F		Antler	○		—	—	—	—	1	1	0	1	0	1	1	0	1	0	1	1	1	1
						○	—	—	—	—	1	1	0	1	0	1	1	0	1	0	1	1	1	1
NSMT	M43309	1F	Juvenile?	Antler	○		—	—	—	—	1	1	0	1	1	1	1	0	1	0	1	1	0	0
						○	—	—	—	—	1	1	0	1	0	1	1	0	1	0	1	1	0	0
NSMT	M07538	1F		Antler	○		—	—	—	—	1	1	0	1	0	1	1	0	1	0	1	1	1	0
						○	—	—	—	—	1	1	0	1	0	1	1	0	1	0	1	1	1	0
NSMT	M01164	1F		Antler	○		—	—	—	—	1	1	0	1	0	1	1	0	1	0	1	1	1	0
						○	—	—	—	—	1	1	0	1	0	1	1	0	1	0	1	1	1	0
NSMT	M01167	1F		Antler	○		—	—	—	—	1	1	1	1	0	1	1	0	1	0	1	1	1	0
						○	—	—	—	—	1	1	1	1	0	1	1	0	1	0	1	1	1	0
NSMT	M01165	1F		Antler	○		—	—	—	—	1	1	1	1	0	1	1	0	1	0	1	1	1	0
						○	—	—	—	—	1	1	0	1	0	1	1	0	1	0	1	1	1	0
NSMT	M42710	7F		Stuffing	○		—	—	—	—	1	1	0	1	0	1	1	0	1	0	1	1	1	1
						○	—	—	—	—	1	1	0	1	0	1	1	0	1	0	1	1	1	1
NSMT	M32034	7F		Stuffing	○		—	—	—	—	1	1	0	1	0	1	1	0	1	1	1	1	1	1
						○	—	—	—	—	1	1	0	1	0	1	1	0	1	0	1	1	1	1
NSMT	M01094	7F	Velvet antlers	Stuffing head	○		—	—	—	—	1	1	0	1	0	1	1	0	1	1	1	1	1	1
						○	—	—	—	—	1	1	0	1	0	1	1	0	1	1	1	1	1	1
NSMT	M39878	7F		Stuffing	○		—	—	—	—	1	1	0	1	1	1	1	0	1	0	1	1	1	1
						○	—	—	—	—	1	1	0	1	1	1	1	0	1	0	1	1	1	1
NSMT	M39877	7F		Stuffing	○		—	—	—	—	1	1	0	1	0	1	1	0	1	0	1	1	1	1
						○	—	—	—	—	1	1	0	1	0	1	1	0	1	0	1	1	1	1
NSMT	M39876	7F		Stuffing	○		—	—	—	—	1	1	0	1	0	1	1	0	1	0	1	1	1	1
						○	—	—	—	—	1	1	0	1	0	1	1	0	1	0	1	1	1	1
NSMT	M32353	7F		Stuffing	○		—	—	—	—	1	1	0	1	0	1	1	0	1	0	1	1	1	1
						○	—	—	—	—	1	1	0	1	0	1	1	0	1	0	1	1	1	1
NSMT	M56402	7F		Stuffing	○		—	—	—	—	1	1	0	1	0	1	1	0	1	0	1	1	1	1
						○	—	—	—	—	1	1	0	1	0	1	1	0	1	0	1	1	1	1
NSMT	M56403	7F		Stuffing	○		—	—	—	—	1	1	0	1	1	1	1	0	1	0	1	1	1	1
						○	—	—	—	—	1	1	0	1	1	1	1	0	1	0	1	1	1	1
TUM	No number		from Kashimir	Antler	○		○				1	1	1	1	1	1	1	0	1	0	1	1	1	1
						○	○				1	1	0	1	1	1	1	0	1	0	1	1	1	1
Sum (excluding "Juvenile?")											34	34	4	34	6	34	34	0	34	3	33	34	33	25
Percentage (excluding "Juvenile?")											100%	100%	12%	100%	18%	100%	100%	0%	100%	9%	97%	100%	97%	74%

Colored tines (two out of Crown-inner tine, Crown-outer tine, and Crown-back tine) join together more distally than the other of the three.

Cervus elaphus

Museum or Institute	Number	Reposited room	Note	Specimen type	R	L	SR	FBSTAMI	TR	BN	Base of antler	Brow tine	Brow process	Beam process	Lower beam	Bez tine	Trez tine	Trez-beam process	Higher beam	Crown-outer tine	Crown-inner tine	Crown-back tine	Crown-back-inner tine	Crown-back-outer tine	Crown-back-back tine
KUGM	019		Juvenile	Antler・Skull	○		○	×	○	○	1	1	0	0	1	0	1	0	1	1	1	1	0	0	0
						○	○	×	○	○	1	1	0	0	1	0	1	0	1	1	1	1	0	0	0
KUGM	048			Antler		○	—	—	—	—	1	1	0	0	1	1	1	0	1	0	1	1	1	1	0
KUGM	132			Antler	○		○	×	○	○	1	1	0	0	1	1	1	0	1	?	?	?	?	?	?
						○	○	×	○	○	1	1	0	0	1	1	1	0	1	?	?	?	?	?	?
NSMT	43318	1F	Juvenile?		○		?	?	?	?	1	1	0	0	1	1	1	0	1	0	0	0	0	0	0
						○	?	?	?	?	1	1	0	0	1	1	1	0	1	1	1	0	0	0	0
NSMT	1162	1F	Scottish red deer	Antler	○		—	—	—	—	1	1	0	0	1	1	1	0	1	0	0	1	1	0	1
						○	—	—	—	—	1	1	0	0	1	1	1	0	1	1	0	1	0	1	1
NSMT	43325	1F	Only "Cervus" is written.	Stuffing	○		—	—	—	—	1	1	0	0	1	1	1	0	1	1	1	1	0	0	0
			Estimated to be *C,elaphus*			○	—	—	—	—	1	1	0	0	1	1	1	0	1	1	1	1	1	1	0
NSMT	32195	7F		Stuffing	○		—	—	—	—	1	1	0	1	1	1	1	0	1	1	1	1	1	0	1
						○	—	—	—	—	1	1	0	1	1	1	1	0	1	1	1	1	0	0	0
NSMT	56408	7F	Distal branch is covered with cushioning material	Stuffing	○		—	—	—	—	1	1	0	1	1	1	1	0	1	?	?	?	?	?	?
						○	—	—	—	—	1	1	0	1	1	1	1	0	1	1	1	1	?	?	?
NSMT	56401	7F		Stuffing	○		—	—	—	—	1	1	0	0	1	1	1	0	1	1	0	1	1	1	1
						○	—	—	—	—	1	1	0	0	1	1	1	0	1	1	0	1	1	0	1
NSMT	32006	7F		Stuffing	○		—	—	—	—	1	1	0	?	1	1	1	0	1	1	1	1	0	0	0
						○	—	—	—	—	1	1	0	?	1	1	1	0	1	1	1	0	0	0	0
NSMT	No number	7F	The face is in ragged	Stuffing	○		—	—	—	—	1	1	0	1	1	1	1	0	1	1	1	1	1	1	0
						○	—	—	—	—	1	1	0	1	1	1	1	0	1	1	1	1	0	0	0
NSMT	1163	7F	German red deer	Antler	○		—	—	—	—	1	1	0	0	1	1	1	0	1	0	1	1	1	0	1
						○	—	—	—	—	1	1	0	1	1	1	1	0	1	0	1	1	1	0	1
NSMT	32194	7F		Stuffing	○		—	—	—	—	1	1	0	0	1	1	1	0	1	1	1	1	0	0	0
						○	—	—	—	—	1	1	0	0	1	1	1	0	1	1	0	1	1	0	1
LBM	1900000213			Antler	○		—	—	—	—	1	1	0	0	1	1	1	0	1	1	0	1	0	1	0
						○	—	—	—	—	1	1	0	0	1	1	1	0	1	1	0	1	0	0	0
TUM	No number	2F	From South Pacific Mandate	Antler	○		○				1	1	0	0	1	1	1	0	1	0	1	1	0	0	0
						○	○				1	1	0	0	1	1	1	0	1	1	0	1	0	0	0
TUM	No number	1F	Displayed	Antler	○		○				1	1	0	0	1	1	1	0	1	1	1	1	1	1	0
						○	○				1	1	0	0	1	1	1	0	1	1	1	1	1	1	0
Sum (excluding "Juvenile" and "Juvenile?")											27	27	0	7	27	27	27	0	27	19	16	23	12	8	8
Percentage (excluding "Juvenile" and "Juvenile?")											100%	100%	0%	26%	100%	100%	100%	0%	100%	79%	67%	96%	52%	35%	35%

Colored tines (two out of Crown-inner tine, Crown-outer tine, and Crown-back tine) join together more distally than the other of the three.

Rusa unicolor

Museum or Institute	Number	Reposited floor	Note	Specimen type	Right	Left	SR	IFST	TR	BN	Base of antler	Brow tine	Brow process	Front-medial tine	Bez tine	Lower beam	Beam process	Trez tine	Trez-beam process	Higher beam	Crown-outer tine	Crown-inner tine	Crown-back tine	Crown-back-inner tine	Crown-back-back tine
KUGM	RM020			Antler, Skull	○		○	○	○	○	1	1	0	0	0	1	0	1	0	1	0	0	0	0	0
						○	○	○	○	○	1	1	0	0	0	1	0	1	0	1	0	0	0	0	0
KUGM	RM039			Antler, Skull	○		○	○	○	○	1	1	0	0	0	1	0	1	0	1	0	0	0	0	0
						○	○	○	○	○	1	1	0	0	0	1	0	1	0	1	0	0	0	0	0
KUGM	RM137			Antler	○		—	—	—	—	1	1	0	0	0	1	0	1	0	1	0	0	0	0	0
						○	—	—	—	—	1	1	0	0	0	1	0	1	0	1	0	0	0	0	0
KUGM	RM139			Antler	○		—	—	—	—	1	1	0	0	0	1	0	1	0	1	0	0	0	0	0
						○	—	—	—	—	1	1	0	0	0	1	0	1	0	1	0	0	0	0	0
KUGM	RM140			Antler	○		—	—	—	—	1	1	0	0	0	1	0	1	0	1	0	0	0	0	0
						○	—	—	—	—	1	1	0	0	0	1	0	1	0	1	0	0	0	0	0
KUGM	RM138			Antler, Skull (half)	○		○	?	○	?	1	1	0	0	0	1	0	1	0	1	0	0	0	0	0
						○	○	?	○	?	1	1	0	0	0	1	0	1	0	1	0	0	0	0	0
NSMT	M01146	1F		Antler, Skull	○						1	1	1	1	0	1	1	1	0	1	0	0	0	0	0
						○					1	1	1	0	0	1	1	1	0	1	0	0	0	0	0
NSMT	M01147	1F		Antler	○						1	1	0	0	0	1	0	1	0	1	0	0	0	0	0
						○					1	1	0	0	0	1	0	1	0	1	0	0	0	0	0
NSMT	M08939	1F		Antler, Skull	○						1	1	0	0	0	1	0	1	0	1	0	0	0	0	0
						○					1	1	0	0	0	1	0	1	0	1	0	0	0	0	0
NSMT	M08932	1F		Antler, Skull	○						1	1	0	0	0	1	0	1	0	1	0	0	0	0	0
						○					1	1	0	0	0	1	0	1	0	1	1	1	1	0	0
NSMT	M08950	1F		Antler, Skull	○						1	1	0	0	0	1	0	1	0	1	0	0	0	0	0
						○					1	1	0	0	0	1	0	1	0	1	0	0	0	0	0
NSMT	M34316	1F		Antler, Skull	○						1	1	0	0	0	1	0	1	0	1	1	1	0	0	0
						○					1	1	0	0	0	1	0	1	1	1	0	0	0	0	0
NSMT	M09624	1F		Antler, Skull	○						1	1	0	0	0	1	0	1	0	1	0	0	0	0	0
						○					1	1	1	0	0	1	0	1	0	1	0	0	0	0	0
NSMT	M36067	1F	Painted blue	Antler, Skull	○						1	1	1	0	0	1	0	1	0	1	0	0	0	0	0
						○					1	1	1	0	0	1	1	1	0	1	0	0	0	0	0
NSMT	M01849	1F		Antler	○		—	—	—	—	1	1	1	0	0	1	0	1	0	1	0	0	0	0	0
						○	—	—	—	—	1	1	0	0	0	1	0	1	0	1	0	0	0	0	0
NSMT	M08955	1F		Antler	○		—	—	—	—	1	1	0	0	0	1	0	1	0	1	0	0	0	0	0
						○	—	—	—	—	1	1	0	0	0	1	0	1	0	1	0	0	0	0	0
NSMT	M08949	1F		Antler	○		—	—	—	—	1	1	0	0	0	1	0	1	0	1	0	0	0	0	0
						○	—	—	—	—	1	1	0	0	0	1	0	1	0	1	0	0	0	0	0
NSMT	M43318	1F	From Borneo	Antler	○		—	—	—	—	1	1	0	0	0	1	0	1	1	1	0	0	0	0	0
						○	—	—	—	—	1	1	0	1	0	1	0	1	1	1	0	0	0	0	0
NSMT	M43308	1F		Antler	○		—	—	—	—	1	1	0	0	0	1	0	1	0	1	0	0	0	0	0
						○	—	—	—	—	1	1	0	0	0	1	0	1	0	1	0	0	0	0	0
NSMT	M32196	7F		Stuffing	○		—	—	—	—	1	1	1	0	0	1	0	1	1	1	0	0	0	0	0
						○	—	—	—	—	1	1	0	1	0	1	0	1	1	1	0	0	0	0	0
NSMT	No number	7F		Stuffing	○		—	—	—	—	1	1	0	0	0	1	0	1	0	1	0	0	0	0	0
						○	—	—	—	—	1	1	0	0	0	1	0	1	0	1	0	0	0	0	0
NSMT	M32138	7F		Stuffing	○		—	—	—	—	1	1	0	0	0	1	0	1	0	1	0	0	0	0	0
						○	—	—	—	—	1	1	0	0	0	1	0	1	0	1	0	0	0	0	0
NSMT	M05986	7F		Stuffing	○		—	—	—	—	1	1	0	0	0	1	0	1	0	1	0	0	0	0	0
						○	—	—	—	—	1	1	0	0	0	1	0	1	0	1	0	0	0	0	0
OMNH	M0435			Antler, Skull	○		○				1	1	0	0	0	1	0	1	0	1	0	0	0	0	0
						○	○				1	1	0	0	0	1	0	1	0	1	0	0	0	0	0
HUBG	10290			Antler, Skull	○		○				1	1	0	0	0	1	0	1	0	1	0	0	0	0	0
						○	○				1	1	0	0	0	1	0	1	0	1	0	0	0	0	0

Rusa unicolor (continued)

Museum or Institute	Number	Reposited floor	Note	Specimen type	Right	Left	SR	IFST	TR	BN	Base of antler	Brow tine	Brow process	Front-medial tine	Bez tine	Lower beam	Beam process	Trez tine	Trez-beam process	Higher beam	Crown-outer tine	Crown-inner tine	Crown-back tine	Crown-back-inner tine	Crown-back-back tine
HUBG	10294			Antler, Skull	○		○				1	1	0	0	0	1	0	1	0	1	0	0	0	0	0
						○	○				1	1	0	0	0	1	0	1	0	1	0	0	0	0	0
HUBG	32836			Antler, Skull	○		○				1	1	0	0	0	1	0	1	0	1	0	0	0	0	0
						○	○				1	1	0	0	0	1	0	1	0	1	0	0	0	0	0
HUBG	10276			Antler, Skull	○		○				1	1	0	0	0	1	0	1	0	1	0	0	0	0	0
						○	○				1	1	0	0	0	1	0	1	0	1	0	0	0	0	0
LBM	19900000008			Antler, Skull	○		○				1	1	0	0	0	1	0	1	1	1	0	0	0	0	0
						○	○				1	1	0	0	0	1	0	1	0	1	0	0	0	0	0
LBM	19900000003			Antler, Skull	○		○				1	1	0	0	1	1	0	1	0	1	0	0	0	0	0
						○	○				1	1	0	1	1	1	0	1	0	1	0	0	0	0	0
										Sum	62	62	7	5	2	62	3	62	6	62	4	2	3	0	0
										Percentage	100%	100%	11%	8%	3%	100%	5%	100%	10%	100%	6%	3%	5%	0%	0%

Rusa timorensis

Museum or Institute	Number	Reposited floor	Specimen type	Right	Left	SR	IFST	TR	BN	Base of antler	Brow tine	Brow process	Front-medial tine	Lower beam	Bez tine	Trez tine	Trez-beam process	Higher beam
KUGM	RM021		Antler, Skull	○		○	○	○	○	1	1	0	0	1	0	1	0	1
					○	○	○	○	○	1	1	0	0	1	0	1	0	1
TG	TK-0152		Antler, Skull	○		○				1	1	0	0	1	0	1	0	1
					○	○				1	1	0	0	1	0	1	0	1
									Sum	4	4	0	0	4	0	4	0	4
									Percentage	100%	100%	0%	0%	100%	0%	100%	0%	100%

Rusa mariaana

Museum or Institute	Number	Reposited floor	Specimen type	Right	Left	SR	IFST	TR	BN	Base of antler	Brow tine	Brow process	Front-medial tine	Lower beam	Bez tine	Trez tine	Trez-beam process	Higher beam
NSMT	M26698	1F	Antler, Skull	○		○	○	○	○	1	1	0	0	1	0	1	0	1
					○	○	○	○	○	1	1	0	0	1	0	1	1	1
HUBG	10291		Antler	○		○		○		1	1	0	0	1	0	1	0	1
					○	○		○		1	1	0	0	1	0	1	0	1
NSMT	M08423	1F	Antler	○		○	○	○	○	1	1	0	0	1	0	1	0	1
					○	○	○	○	○	1	1	0	1	1	0	1	0	1
									Sum	6	6	0	1	6	0	6	1	6
									Percentage	100%	100%	0%	17%	100%	0%	100%	17%	100%

Rusa sp.

Museum or Institute	Number	Note	Specimen type	Right	Left	SR	IFST	TR	BN	Base of antler	Brow tine	Brow process	Front-medial tine	Bez tine	Lower beam	Beam process	Sub-medial tine	Trez tine	Trez-beam process	Higher beam	Crown-outer tine	Crown-inner tine	Crown-back tine
KUGM	RM210	With mutilpe processes	Antler, Skull	○		○	○	○	○	1	1	0	1	0	1	0	1	1	1	1	0	0	0
					○	○	○	○	○	1	1	0	1	0	1	0	1	1	1	1	0	0	0
KUGM	RM211		Antler, Skull	○		?	?	?	?	1	1	0	0	0	0	0	1	1	0	1	0	0	0
					○	?	?	?	?	1	1	0	0	0	0	0	1	1	1	1	0	0	0
KUGM	RM212		Antler, Skull	○		○	○	○	○	1	1	0	0	0	0	0	0	1	0	1	0	0	0
					○	○	○	○	○	1	1	0	0	0	0	0	1	1	0	1	0	0	0

Elaphurus davidianus

Museum or Institute	Number	Reposited floor or place	Note	Specimen type	Right	Left	SR	IFST	TR	BN	Base of antler	Brow tine	Brow-outer tine	Front-inner tine	Brow process	Lower beam	Bez tine	Beam process	Medial tine	Trez tine	Higher beam	Pre-cauminal tines	Pre-trez tines
GMNS	RM022			Skull, Antler	○		○	×	○	○	1	1	1	1	0	1	0	0	0	0	1	0	0
						○	○	×	○	○	1	1	1	1	0	1	0	0	0	0	1	0	0
GMNS	RM041			Antler		○	—	—	—	—	1	1	1	1	0	1	0	0	0	0	1	0	0
GMNS	RM042			Antler		○	—	—	—	—	1	1	1	1	0	1	0	0	0	0	1	0	1
GMNS	RM043			Antler	○		—	—	—	—	1	1	1	1	0	1	0	0	0	0	1	0	1
GMNS	RM044			Antler	○		—	—	—	—	1	1	1	1	0	1	0	0	0	0	1	1	1
						○	—	—	—	—	1	1	1	1	0	1	0	0	0	0	1	1	1
GMNS	RM153			Antler		○	—	—	—	—	1	1	1	1	0	1	0	0	0	0	1	0	1
GMNS	RM154			Antler		○	—	—	—	—	1	1	1	1	0	1	0	0	0	0	1	0	1
GMNS	RM155			Antler		○	—	—	—	—	1	1	1	1	0	1	0	0	0	0	1	0	0
NSMT	M00464		from Qing dynasty	Complete skeleton	○		○	×	○	○	1	1	1	1	0	1	0	0	1	0	?	0	0
						○	○	×	○	○	1	1	1	1	0	1	0	0	1	1	1	0	0
NSMT	M00425	1F	from Qing dynasty	Antler	○		—	—	—	—	1	1	1	1	0	1	0	0	0	0	1	1	0
						○	—	—	—	—	1	1	1	1	0	1	0	0	1?	0	1	0	0
NSMT	M01168	1F		Antler, Skull	○		○	×	○	○	1	1	1	1	0	1	0	0	0	0	1	1	1
						○	○	×	○	○	1	1	1	1	0	1	0	0	0	0	1	0	1
NSMT	M38630	1F		Antler, Skull	○		○	×	○	○	1	1	1	1	0	1	0	0	0	0	1	1	0
						○	○	×	○	○	1	1	1	1	0	1	0	0	0	0	1	1	0
										Sum	18	18	18	18	0	18	0	0	2	1	17	6	8
										Percentage	100%	100%	100%	100%	0%	100%	0%	0%	11%	6%	100%	33%	44%

Panolia eldii

Museum or Institute	Number	Reposited floor	Note	Specimen type	Right	Left	SR	IFST	TR	BN	Base of antler	Brow tine	Brow process	Lower beam	Beam process	Medial tine	Trez tine	Cacuminal tine	Pre-cacuminal tines	Higher beam
LBM	1990000009=1990000010		Replicas of the same individual	Skull, Antler	○		—	—	—	—	1	1	1	1	1	1	0	1	0	1
						○	—	—	—	—	1	1	1	1	1	1	0	1	1	1
OMNH	M2034			Skull, Antler	○		○	○	○	○	1	1	1	1	0	1	0	1	0	1
						○	○	○	○	○	1	1	1	1	0	1	0	1	0	1
HUBG	10286			Antler	○		○		○	○	1	1	1	1	1	1	0	1	1	1
						○	○		○	○	1	1	1	1	1	1	0	1	1	1
NSMT	M01153	1F		Antler	○		○	○	○	○	1	1	1	1	1	1	0	1	1	1
						○	○	○	○	○	1	1	1	1	1	1	0	1	1	1
NSMT	M01155	1F		Antler	○		○		○	○	1	1	2	1	0	1	0	1	1	1
						○	○		○	○	1	1	1	1	0	1	0	1	1	1
NSMT	M01154	7F		Stuffing	○		—	—	—	—	1	1	0	1	0	1	0	1	1	1
						○	—	—	—	—	1	1	1	1	0	1	0	1	0	1
										Sum	12	12	12	12	6	12	0	12	8	12
										Percentage	100%	100%	100%	100%	50%	100%	0%	100%	67%	100%

Dama dama (Mature antlers)

Museum or Institute	Number	Reposited floor	Note	Specimen type	Right	Left	SR	IFST	TR	BN	Base of antler	Brow tine	Brow process	Lower beam	Beam process	Post beam process	Bez tine	Trez tine	Trez-beam process	Higher beam	Guard tine	Crown-outer tine	Crown-inner tine	Crown-inner tine bifurcation	Crown-back tine	Back-tail-inner tine	Crown-back-back tine	Back tine and its distal tines palmation
KUGM	RM011			Skull、Antler	○		○	×	×	○	1	1	0	1	0	0	0	1	0	1	0	0	1	1	1	0	0	1
						○	○	○	×	○	1	1	0	1	0	0	0	1	0	1	1	0	1	1	1	0	0	1
KUGM	RM010			Skull、Antler	○		○	×	×	○	1	1	0	1	0	0	0	1	0	1	0	0	1	1	1	0	0	1
						○	○	×	×	○	1	1	0	1	0	0	1	1	0	1	1	0	1	1	1	1	0	1
KUGM	RM045			Antler	○		—	—	—	—	1	1	0	1	0	0	0	1	0	1	1	0	1	1	1	0	0	1
KUGM	RM133			Antler	○		—	—	—	—	1	1	0	1	0	0	0	1	0	1	1	0	1	1	1	1	1	1
KUGM	RM143			Antler	○		—	—	—	—	1	1	0	1	1	0	1	0	0	1	1	0	1	1	1	1	1	1
KUGM	RM144			Antler	○		—	—	—	—	1	1	0	1	0	1	0	1	0	1	1	0	1	1	1	1	1	1
KUGM	RM107			Antler	○		—	—	—	—	1	1	0	1	0	0	0	1	0	1	0	0	1	1	1	1	0	1
						○	—	—	—	—	1	1	0	1	1	1	0	1	0	1	0	1	1	1	1	1	0	1
LBM	1900000787			Skull, Antler	○		○	○	×	○	1	1	0	1	0	1	0	1	0	1	1	0	1	1	1	0	0	1
						○	○	○	×	○	1	1	0	1	0	0	0	1	0	1	1	0	1	1	1	0	0	1
NSMT	M32070	7F		Stuffing	○		—	—	—	—	1	1	0	1	0	0	0	1	0	1	1	0	1	1	1	1	1	1
						○	—	—	—	—	1	1	0	1	0	0	0	1	0	1	1	0	1	1	1	0	0	1
NSMT	M32192	7F		Stuffing	○		—	—	—	—	1	1	0	1	0	0	0	1	0	1	1	0	1	1	1	0	0	1
						○	—	—	—	—	1	1	0	1	0	0	0	1	0	1	1	0	1	1	1	0	0	1
NSMT	M56407	7F		Head Stuffing	○		—	—	—	—	1	1	0	1	0	0	0	1	0	1	1	0	1	1	1	1	1	1
						○	—	—	—	—	1	1	0	1	0	0	0	1	1	1	1	0	1	1	1	1	0	1
NSMT	No number	7F	Lamp (Upper)	Antler	○		—	—	—	—	1	1	0	1	0	0	0	1	0	1	1	0	1	1	0	0	1	1
						○	—	—	—	—	1	1	0	1	0	0	0	1	0	1	1	0	1	1	0	0	1	1
NSMT	No number	7F	Lamp (Lower)	Antler	○		—	—	—	—	1	1	0	1	0	0	0	1	0	1	1	0	1	1	0	0	1	1
						○	—	—	—	—	1	1	0	1	0	0	0	1	0	1	1	0	1	1	1	1	1	1
										Sum	22	22	0	22	2	3	2	21	1	22	18	1	22	22	19	10	9	22
										Percentage	100%	100%	0%	100%	9%	14%	9%	95%	5%	100%	82%	5%	100%	100%	86%	45%	41%	100%

Dama dama (Juvenile antlers)

Museum or Institute	Number	Note	Specimen type	Right	Left	SR	IFST	TR	BN	Base of antler	Brow tine	Brow process	Lower beam	Beam process	Post beam process	Bez tine	Trez tine	Trez-beam process	Higher beam	Guard tine	Crown-outer tine	Crown-inner tine	Crown-inner tine bifurcation	Crown-back tine	Back-tail-inner tine	Crown-back-back tine	Back tine and its distal tines palmation
KUGM	RM056	Juvenile	Antler		○	—	—	—	—	1	1	0	0	0	0	0	1	0	1	0	0	1	0	1	1	1	0
KUGM	RM200	Juvenile	Antler		○	—	—	—	—	1	1	0	0	0	0	0	1	0	1	0	0	1	1	1	0	0	0
KUGM	RM201	Juvenile	Antler	○		—	—	—	—	1	1	0	0	0	0	0	1	0	1	0	0	1	0	1	0	0	0
KUGM	RM202	Juvenile	Antler	○		—	—	—	—	1	1	0	0	0	0	0	1	0	1	0	0	1	0	1	1	1	0
KUGM	RM203	Juvenile	Antler	○		—	—	—	—	1	1	0	0	0	0	0	1	0	1	0	0	1	1	1	0	0	0
KUGM	RM204	Juvenile	Antler	○		—	—	—	—	1	1	0	0	0	0	0	1	0	1	0	0	1	0	1	0	0	0
KUGM	RM205	Juvenile	Antler	○		—	—	—	—	1	1	0	0	0	0	0	1	0	1	0	0	1	?	1	1	1	0

Axis axis

Museum or Institute	Number	Reposited floor	Specimen type	Right	Left	SR	IFST	TR	BN	Base of antler	Brow tine	Brow process	Lower beam	Beam process	Trez tine	Higher beam
KUGM	RM014		Skull, Antler	○		○	○	○	○	1	1	0	1	0	1	1
					○	○	○	○	○	1	1	0	1	0	1	1
KUGM	RM047		Antler	○		—	—	—	—	1	1	0	1	0	1	1
KUGM	RM046		Antler		○	—	—	—	—	1	1	1	1	0	1	1
NSMT	M01145	1F	Antler	○		○		○	○	1	1	0	1	0	1	1
					○	○		○	○	1	1	0	1	0	1	1
NSMT	M32346	7F	Stuffing	○		—	—	—	—	1	1	0	1	0	1	1
					○	—	—	—	—	1	1	0	1	0	1	1
NSMT	M32342	7F	Stuffing	○		—	—	—	—	1	1	0	1	0	1	1
					○	—	—	—	—	1	1	0	1	0	1	1
NSMT	M32131	7F	Antler	○		—	—	—	—	1	1	0	1	0	1	1
					○	—	—	—	—	1	1	0	1	0	1	1
NSMT	M32345	7F	Stuffing	○		—	—	—	—	1	1	0	1	0	1	1
					○	—	—	—	—	1	1	0	1	0	1	1
NSMT	M32132	7F	Stuffing	○		—	—	—	—	1	1	0	1	0	1	1
					○	—	—	—	—	1	1	0	1	0	1	1
NSMT	M00131	7F	Antler	○		○		○	○	1	1	0	1	0	1	1
					○	○		○	○	1	1	0	1	0	1	1
NSMT	M42835	7F	Stuffing	○		—	—	—	—	1	1	0	1	0	1	1
					○	—	—	—	—	1	1	0	1	0	1	1
									Sum	20	20	1	20	0	20	20
									Percentag	100%	100%	5%	100%	0%	100%	100%

Elaphodus cephalophus

Museum or Institute	Number	Specimen type	Right	Left	SR	IFST	TR	BN	Base of antler
KPM	No number	Antler, Skull	○		○	○	○	○	1
				○	○	○	○	○	1
								Sum	2
								Percentage	100%

Axis porcinus

Museum or Institute	Number	Reposited floor	Specimen type	Right	Left	SR	IFST	TR	BN	Base of antler	Brow tine	Brow process	Lower beam	Beam process	Trez tine	Higher beam
NSMT	M19849	1F	Skull, Antler	○		○	○	○	○	1	1	0	1	0	1	1
					○	○	○	○	○	1	1	0	1	0	1	1
									Sum	2	2	0	2	0	2	2
									Percentage	100%	100%	0%	100%	0%	100%	100%

Rucervus duvaucelii

Museum or Institute	Number	Reposited floor	Specimen type	Right	Left	SR	IFST	TR	BN	Base of antler	Brow tine	Brow process	Lower beam	Beam process	Vertical beam	Verticl-1st tine	Verticl-2nd tine	Back beam	Back-1st tine	Back-2nd tine
NSMT	M01152	1F	Skull, Antler	○		○	○	○	○	1	1	0	1	0	1	1	1	1	1	0
					○	○	○	○	○	1	1	0	1	0	1	1	1	1	1	0
TUM	No number		Head Stuffing	○		—	—	—	—	1	1	1	1	0	1	1	1	1	1	0
					○	—	—	—	—	1	1	1	1	0	1	1	1	1	1	0
									Sum	4	4	2	4	0	4	4	4	4	4	0
									Percentage	100%	100%	50%	100%	0%	100%	100%	100%	100%	100%	0%

Rucervus schomburgki

Institute	Number	floor	Specimen type	Right	Left	SR	FBSTAMI	TR	BN	Base of antler	Brow tine	Brow process	Lower beam	Beam process	Vertical beam	Verticl-1st tine	Verticl-2nd tine	Back beam	Back-1st tine	Back-2nd tine
NSMT	M43311	1F	Skull, Antler	○		○	○	○	○	1	1	0	1	0	1	1	1	1	1	1
					○	○	○	○	○	1	1	0	1	0	1	1	1	1	1	1
NSMT	M01151	1F	Antler	○		○	○	○	○	1	1	0	1	0	1	1	1	1	1	1
					○	○	○	○	○	1	1	0	1	0	1	1	1	1	1	1
									Sum	4	4	0	4	0	4	4	4	4	4	4
									Percentag	100%	100%	0%	100%	0%	100%	100%	100%	100%	100%	100%

Muntiacus reevesi<br>Museum or Institute	Number	Reposited floor/Note	Specimen type	Right	Left	SR	IFST	TR	BN	Base of antler	Brow tine	Lower beam
KUGM	RM024		Antler・skull	○		○	○	○	○	1	1	1
					○	○	○	○	○	1	1	1
KUGM	RM026		Antler・skull	○		○	○	○	○	1	1	1
					○	○	○	○	○	1	1	1
NSMT	M43315	1F	Antler	○		—	—	—	—	1	1	1
	(Upper)				○	—	—	—	—	1	1	1
NSMT	M43315	1F	Antler	○		—	—	—	—	1	1	1
	(Lower)				○	—	—	—	—	1	1	1
CBM	ZZ0004650	1F	Antler・skull	○		○				1	1	1
					○	○				1	1	1
CBM	ZZ0004982	1F	Antler・skull	○		○				1	1	1
					○	○				1	1	1
CBM	ZZ0005697	1F	Antler・skull	○		○				1	1	1
					○	○				1	1	1
CBM	ZZ0003058	1F	Antler・skull	○		○				1	1	1
					○	○				1	1	1
CBM	ZZ0005820		Antler・skull	○		○				1	1	1
					○	○				1	1	1
CBM	ZZ0005700		Antler・skull	○		○				1	0	1
					○	○				1	1	1
CBM	ZZ0005689		Antler・skull	○		○				1	0	1
					○	○				1	1	1
CBM	ZZ0004663		Antler・skull	○		○				1	1	1
					○	○				1	1	1
CBM	ZZ0004638		Antler・skull	○		○				1	1	1
					○	○				1	1	1
CBM	ZZ0004636		Antler・skull	○		○				1	1	1
					○	○				1	1	1
CBM	ZZ0004679		Antler・skull	○		○				1	1	1
					○	○				1	1	1
CBM	ZZ0004669		Antler・skull	○		○				1	1	1
					○	○				1	1	1
CBM	ZZ0003059		Antler・skull	○		○				1	1	1
					○	○				1	1	1
CBM	ZZ0006103		Antler・skull	○		○				1	1	1
					○	○				1	1	1
CBM	ZZ0006096		Antler・skull	○		○				1	1	1
					○	○				1	1	1
CBM	IM9	Isumi, Chiba	Antler・skull	○		○				1	1	1
					○	○				1	1	1
CBM	ZZ0006097		Antler・skull	○		○				1	1	1
					○	○				1	1	1
CBM	ZZ0006130		Antler・skull	○		○				1	1	1
					○	○				1	1	1
CBM	ZZ0004648		Antler・skull	○		○				1	1	1
					○	○				1	1	1
CBM	ZZ0004606		Antler・skull	○		○				1	1	1
					○	○				1	1	1
									Sum	44	42	44
									Percentage	100%	95%	100%

Muntiacus muntjak<br>Museum or Institute	Number	Reposited floor	Specimen type	Right	Left	SR	IFST	TR	BN	Base of antler	Brow tine	Lower beam
NSMT	M01144	1F	Antler・skull	○		○	○	○	○	1	1	1
					○	○	○	○	○	1	1	1
KUGM	RM023		Antler・skull	○		○		○	○	1	1	1
					○	○		○	○	1	1	1
									Sum	4	4	4
									Percentage	100%	100%	100%

Capreolus pygargus

Museum or Institute	Number	Reposited floor	Specimen type	Right	Left	SR	IFST	TR	BN	Base of antler	Brow tine	Lower beam	Frontal tine	Frontal-inner tine	Upper beam	Post-frontal tine
KUGM	RM131		Antler	○		○	○	○	○	1	1	1	1	1	1	1
					○	○	○	○	○	1	1	1	1	0	1	0
KUGM	RM080		Antler	○						1	1	1	1	0	1	0
					○					1	1	1	1	0	1	0
KUGM	RM081		Antler	○		○	○	○	○	1	1	1	1	0	1	0
					○	○	○	○	○	1	1	1	1	0	1	0
KUGM	RM082		Antler	○						1	1	1	1	0	1	0
					○					1	1	1	1	0	1	0
KUGM	RM083		Antler	○						1	1	1	1	0	1	0
					○					1	1	1	1	0	1	0
KUGM	RM084		Antler	○						1	1	1	1	0	1	0
					○					1	1	1	1	0	1	1
KUGM	RM085		Antler	○						1	1	1	1	0	1	0
					○					1	1	1	1	0	1	0
KUGM	RM086		Antler	○						1	1	1	1	0	1	0
					○					1	1	1	1	0	1	0
KUGM	RM087		Antler	○						1	1	1	1	0	1	0
					○					1	1	1	1	0	1	0
KUGM	RM130		Antler	○						1	0	1	1	0	1	0
NSMT	M23806	1F	Antler	○						1	1	1	1	0	1	0
					○					1	1	1	1	0	1	0
NSMT	M01169	1F	Antler	○						1	1	1	1	0	1	0
	Upper				○					1	1	1	1	0	1	0
NSMT	M01169	1F	Antler	○						1	1	1	1	0	1	0
	Middle				○					1	1	1	1	0	1	0
NSMT	M01169	1F	Antler	○						1	1	1	1	0	1	0
	Lower				○					1	1	1	1	0	1	0
NSMT	M01170	1F	Antler	○						1	1	1	1	0	1	1
					○					1	1	1	1	0	1	1
NSMT	M56419	7F	Skull	○						1	1	1	1	0	1	0
					○					1	1	1	1	0	1	0
									Sum	31	30	31	31	1	31	4
									Percentage	100%	97%	100%	100%	3%	100%	13%

Capreolus caperolus

Museum or Institute	Number	Reposited floor/Note	Specimen type	Right	Left	SR	IFST	TR	BN	Base of antler	Brow tine	Lower beam	Frontal tine	Upper beam
KUGM	RM027		Skull、Antler	○		○	○	○	○	1	1	1	1	1
					○	○	○	○	○	1	1	1	1	1
KUGM	RM028		Complete skeleton	○		○	×	○	○	1	1	1	1	1
					○	○	×	○	○	1	1	1	1	1
KUGM	RM102	Juvenile？	Skull、Antler	○		○	×	○	○	1	1	1	0	0
					○	○	○	○	○	1	1	1	1	1
KUGM	RM101	Juvenile？	Skull、Antler	○		○	×	○	○	1	1	1	1	1
					○	○	×	○	○	1	1	1	1	1
KUGM	RM097	Juvenile？	Skull、Antler	○		○	○	○	○	1	1	1	1	1
					○	○	×	○	○	1	1	1	1	0
KUGM	RM100	Juvenile？	Skull、Antler	○		○	×	○	○	1	1	1	1	1
					○	○	×	○	○	1	1	1	1	1
NSMT	M43323	1F	Antler	○		—	—	—	—	1	1	1	1	1
					○	—	—	—	—	1	1	1	1	1
NSMT	M911	1F	Complete skeleton	○		○	○	○	○	1	1	1	1	1
					○	○	○	○	○	1	1	1	1	1
NSMT	M32377	7F	Stuffing	○		—	—	—	—	1	1	1	1	1
					○	—	—	—	—	1	1	1	1	1
NSMT	M32247	7F	Stuffing	○		—	—	—	—	1	1	1	1	1
					○	—	—	—	—	1	1	1	1	1
NSMT	M56410	7F	Skull	○		—	—	—	—	1	1	1	1	1
					○	—	—	—	—	1	1	1	1	1
NSMT	M32183	7F	Skull	○		—	—	—	—	1	1	1	1	1
					○	—	—	—	—	1	0	1	1	1
									Sum	24	23	24	23	22
									Percentage	100%	96%	100%	96%	92%

Hydropotes intermis

Museum or Institute	Number	Specimen type	
KUGM	RM031	Skull	No antlers
KUGM	RM215	Skull	No antlers

Alces alces

Museum or Institute	Number	Reposited floor	Note	Specimen type	Right	Left	SR	IFST	TR	BN	Base of antler	Brow tine	Middke tine	Lower beam	Upper beam	Terminal-anterior tine	Terminal-anterior tine biffurcation	Terminal-anterior tine palmation	Terminal-posterior tine	Terminal-posterior tine palmation	Terminal-posterior-anterior tines
KUGM	RM029			Skull、Antler	○		○	×	○	○	1	0	0	1	1	1	0	0	1	1	1
						○	○	×	○	○	1	0	0	1	1	1	0	0	1	1	1
KUGM	RM049			Antler	○		—	—	—	—	1	0	0	1	1	1	1	0	1	1	1
						○	—	—	—	—	1	0	0	1	1	1	1	1	1	1	1
KUGM	RM152			Antler	○		—	—	—	—	1	0	0	1	1	1	0	0	1	0	1
KUGM	RM151			Antler	○		—	—	—	—	1	0	0	1	1	1	0	0	1	1	1
KUGM	RM150			Antler	○		—	—	—	—	1	0	0	1	1	1	0	0	1	0	1
KUGM	RM148			Antler	○		—	—	—	—	1	0	0	1	1	1	0	0	1	1	1
KUGM	RM146			Antler		○	—	—	—	—	1	0	0	1	1	1	1	0	1	1	1
KUGM	RM147			Antler		○	—	—	—	—	1	0	0	1	1	1	1	0	1	1	1
NSMT	M43310	1F		Antler	○		○	×	○	○	1	0	0	1	1	1	1	0	1	0	1
						○	○	×	○	○	1	0	0	1	1	1	1	0	1	0	1
NSMT	M56471	1F		Antler	○		—	—	—	—	1	0	0	1	1	1	1	0	1	0	1
						○	—	—	—	—	1	0	0	1	1	1	1	0	1	0	1
NSMT	M01172	1F		Antler	○		—	—	—	—	1	0	0	1	1	1	1	1	1	1	1
						○	—	—	—	—	1	0	0	1	1	1	1	1	1	1	1
NSMT	M01095	1F		Antler+Skull	○		○	×	○	○	1	0	0	1	1	1	1	1	1	1	1
						○	○	×	○	○	1	0	0	1	1	1	1	1	1	1	1
NSMT	M32304	7F		Stuffing	○		—	—	—	—	1	0	0	1	1	1	1	1	1	1	1
						○	—	—	—	—	1	0	0	1	1	1	1	1	1	1	1
NSMT	M32040	7F		Stuffing	○		—	—	—	—	1	0	0	1	1	1	1	1	1	1	1
						○	—	—	—	—	1	0	0	1	1	1	1	1	1	1	1
NSMT	M32374	7F		Stuffing	○		—	—	—	—	1	0	0	1	1	1	1	0	1	1	1
						○	—	—	—	—	1	0	0	1	1	1	1	0	1	1	1
NSMT	M39880	7F		Stuffing	○		—	—	—	—	1	0	0	1	1	1	1	1	1	1	1
						○	—	—	—	—	1	0	0	1	1	1	1	1	1	1	1
NSMT	M39881	7F		Stuffing	○		—	—	—	—	1	0	0	1	1	1	1	1	1	1	1
						○	—	—	—	—	1	0	0	1	1	1	1	1	1	1	1
NSMT	M34879	7F		Stuffing	○		—	—	—	—	1	0	0	1	1	1	1	1	1	1	1
						○	—	—	—	—	1	0	0	1	1	1	1	1	1	1	1
NSMT	M56391	7F		Stuffing	○		—	—	—	—	1	0	0	1	1	1	1	1	1	1	1
						○	—	—	—	—	1	0	0	1	1	1	1	1	1	1	1
NSMT	M56393	7F		Stuffing	○		—	—	—	—	1	0	0	1	1	1	0	0	1	0	1
						○	—	—	—	—	1	0	0	1	1	1	0	0	1	0	1
NSMT	M32305	7F		Antler	○		—	—	—	—	1	0	0	1	1	1	1	1	1	1	1
						○	—	—	—	—	1	0	0	1	1	1	1	1	1	1	1
NSMT	M01052	7F	Siberian elk	Stuffing	○		—	—	—	—	1	0	0	1	1	1	0	0	1	0	0
						○	—	—	—	—	1	0	0	1	1	1	0	0	1	0	0
NSMT	M32305	7F		Antler	○		—	—	—	—	1	0	0	1	1	1	1	1	1	1	1
						○	—	—	—	—	1	0	0	1	1	1	1	1	1	1	1
										Sum	40	0	0	40	40	40	30	21	40	30	38
										Percentage	100%	0%	0%	100%	100%	100%	75%	53%	100%	75%	95%

Rangifer tarandus

Museum or Institute	Number	Reposited floor	Note	Specimen type	Right	Left	SR	IFST	TR	BN	Base of antler	Brow tine	Brow-anterior tines	Brow tine and Brow-anterior tines palmation	Lower beam	Frontal tine	Frontal-posterior tines	Frontal tine and Frontal-posterior tines palmation	Upper beam	Rear tine	Post-frontal tine	Terminal-anterior tine	Terminal-anterior tine bifurcation	Terminal-anterior tine palmation	Terminal-posterior tine	Terminal-posterior-anterior tines	Terminal-posterior tine palmation
KUGM	RM052		Trifurcation of F, M and R	Antler	○		—	—	—	—	1	1	1	1	1	1	1	0	1	1	0	1	1	1	1	1	0
						○	—	—	—	—	1	1	1	1	1	1	1	0	1	0	0	1	1	0	0	0	0
KUGM	RM051			Antler	○		—	—	—	—	1	1	0	0	1	1	0	0	1	1	0	1	1	0	1	0	0
						○	—	—	—	—	1	1	0	0	1	1	0	0	1	1	0	1	1	0	1	0	0
KUGM	RM050			Antler (Stuffing)	○		—	—	—	—	1	1	1	0	1	1	1	0	1	1	0	1	0	0	0	0	0
						○	—	—	—	—	1	1	1	0	1	1	1	0	1	1	0	1	0	0	1	0	0
NSMT	M56438	1F	On board	Antler	○		×	×	○	○	1	1	0	0	1	1	1	0	1	1	0	1	1	0	1	0	0
						○	×	×	○	○	1	1	1	1	1	1	1	0	1	1	0	1	1	0	1	0	0
NSMT	M31343	1F		Antler	○		×	×	○	○	1	1	1	0	1	1	1	0	1	0	0	1	0	0	1	1	0
						○	×	×	○	○	1	1	0	0	1	1	1	0	1	1	0	1	1	0	1	1	0
NSMT	M01248	1F		Antler	○		—	—	—	—	1	1	1	0	1	1	1	0	1	0	0	1	1	0	1	1	0
						○	—	—	—	—	1	1	0	0	1	1	1	0	1	1	0	1	1	0	1	1	0
NSMT	M01096	1F		Antler	○		—	—	—	—	1	1	0	0	1	1	1	0	1	1	0	1	1	0	1	1	0
						○	—	—	—	—	1	1	1	1	1	1	1	1	1	1	0	1	1	0	1	1	0
NSMT	M01173	1F		Antler	○		—	—	—	—	1	1	0	0	1	1	1	0	1	1	0	1	1	0	1	1	0
						○	—	—	—	—	1	1	1	0	1	1	1	0	1	1	0	1	1	0	1	0	0
NSMT	M01174	1F		Antler	○		—	—	—	—	1	1	1	1	1	1	1	0	1	1	0	1	1	1	1	1	0
						○	—	—	—	—	1	1	0	0	1	1	1	0	1	1	0	1	1	1	1	1	0
NSMT	M54712	1F	The tip is missing	Antler	○		—	—	—	—	1	1	1	1	1	1	1	0	1	0	0	1	1	0	1	0	0
						○	—	—	—	—	1	1	1	1	1	1	1	0	1	0	0	1	0	0	1	0	0
NSMT	M01023	1F		Antler・Skull	○		×	×	○	○	1	1	1	0	1	1	1	1	1	1	0	1	1	0	1	0	0
						○	×	×	○	○	1	1	0	0	1	1	1	0	1	1	0	1	1	0	1	0	0
NSMT	M10205	7F		Antler・Skull	○		×	×	○		1	1	0	0	1	1	0	0	1	0	0	1	0	0	1	0	0
						○	×	×	○		1	1	1	0	1	1	0	0	1	0	0	1	0	0	1	0	0
NSMT	M32042	7F		Stuffing	○		—	—	—	—	1	1	1	1	1	1	1	1	1	1	0	1	0	1	1	0	0
						○	—	—	—	—	1	1	1	1	1	1	1	1	1	1	0	1	0	1	1	0	0
NSMT	M32032	7F		Stuffing	○		—	—	—	—	1	1	1	1	1	1	1	1	1	1	0	1	1	0	1	1	0
						○	—	—	—	—	1	1	1	0	1	1	1	0	1	1	0	1	0	0	1	0	0
NSMT	M32044	7F		Stuffing	○		—	—	—	—	1	1	1	1	1	1	1	1	1	1	0	1	1	1	1	1	0
						○	—	—	—	—	1	1	1	1	1	1	1	1	1	1	0	1	1	1	1	0	0
NSMT	M32318	7F		Stuffing	○		—	—	—	—	1	1	1	0	1	1	1	0	1	1	0	1	1	0	1	0	0
						○	—	—	—	—	1	1	1	1	1	1	1	0	1	1	0	1	1	0	1	0	0
NSMT	M32307	7F		Stuffing	○		—	—	—	—	1	1	1	1	1	1	1	0	1	1	0	1	1	1	1	0	0
						○	—	—	—	—	1	1	1	1	1	1	1	0	1	1	0	1	1	1	1	0	0
NSMT	M32317	7F		Stuffing	○		—	—	—	—	1	1	1	1	1	1	1	0	1	1	0	1	1	0	1	1	0
						○	—	—	—	—	1	1	1	0	1	1	1	0	1	1	0	1	1	0	1	0	0
NSMT	M32043	7F		Stuffing	○		—	—	—	—	1	1	1	1	1	1	1	1	1	1	0	1	1	1	1	1	1
						○	—	—	—	—	1	1	1	1	1	1	1	1	1	1	0	1	1	1	1	1	1
NSMT	M42840	7F		Stuffing	○		—	—	—	—	1	1	0	0	1	1	1	0	1	1	0	1	0	0	1	1	0
						○	—	—	—	—	1	1	1	1	1	1	0	0	1	1	0	1	0	0	1	0	0
NSMT	M39883	7F		Stuffing	○		—	—	—	—	1	1	1	1	1	1	1	1	1	1	0	1	1	1	1	1	0
						○	—	—	—	—	1	1	0	0	1	1	1	1	1	1	0	1	1	1	1	1	0
NSMT	M39882	7F		Stuffing	○		—	—	—	—	1	1	1	1	1	1	1	1	1	1	0	1	1	1	1	1	0
						○	—	—	—	—	1	1	0	0	1	1	1	1	1	1	0	1	1	1	1	1	0
NSMT	M39884	7F		Stuffing	○		—	—	—	—	1	1	1	1	1	1	1	1	1	1	0	1	0	1	1	1	0
						○	—	—	—	—	1	1	0	0	1	1	1	1	1	1	0	1	0	1	1	1	0
NSMT	M56387	7F		Stuffing	○		—	—	—	—	1	1	1	0	1	1	1	1	1	1	0	1	1	0	1	1	0
						○	—	—	—	—	1	1	1	1	1	1	1	0	1	1	0	1	1	0	1	1	0
NSMT	M43364	7F	Antler is detached	Stuffing	○		—	—	—	—	1	1	0	0	1	1	1	0	1	1	0	1	1	1	1	1	1
						○	—	—	—	—	1	1	1	1	1	1	1	1	1	1	0	1	1	1	1	1	1
KUZ	M00205			Antler		○	—	—	—	—	1	1	0	0	1	1	1	0	1	1	1	1	1	0	0	0	0
KUZ	No number			Antler	○		—	—	—	—	1	1	0	0	1	1	1	0	1	1	0	1	1	0	1	0	0
						○	—	—	—	—	1	1	0	0	1	1	1	0	1	1	0	1	1	0	1	0	0
										Sum	49	49	33	22	49	49	46	17	49	43	1	49	36	18	46	25	4
										Percentage	100%	100%	67%	45%	100%	100%	94%	35%	100%	88%	2%	100%	73%	37%	94%	51%	8%

Odocoileus hemionus

Museum or Institute	Number	Reposited floor	Specimen type	Right	Left	SR	IFST	TR	BN	Base of antler	Brow tine	Spra-burr process	Upper beam	Rear tine	Upper-1st tine	Upper-2nd tine	Upper-first-anterior/posterior tine	
KUGM	RM054		Antler		○	—	—	—	—	1	0	0	1	1	1	1	1	
KUGM	RM053		Antler	○		—	—	—	—	1	0	1	1	1	1	1	1	
KUGM	RM055		Antler		○	—	—	—	—	1	0	0	1	1	1	1	1	
KUGM	RM032		Antler, Skull	○		○	×	○	○	1	0	0	1	1	1	1	1	
					○	○	×	○	○	1	0	0	1	1	1	1	1	
KUGM	RM076		Antler, Part of skull	○		—	—	—	—	1	0	0	1	1	1	0	0	
					○	—	—	—	—	1	0	0	1	1	1	0	0	
NSMT	M32364	7F	Stuffed	○		—	—	—	—	1	0	0	1	1	1	1	0	
					○	—	—	—	—	1	0	0	1	1	1	1	0	
NSMT	M32409	7F	Antler, Skull	○		—	—	—	—	1	0	0	1	0	1	1	0	*
					○	—	—	—	—	1	0	0	1	0	1	1	0	*
NSMT	M32355	7F	Stuffed	○		—	—	—	—	1	0	0	1	0	1	1	0	*
					○	—	—	—	—	1	0	0	1	0	1	1	0	*
NSMT	M56412	7F	Antler	○		—	—	—	—	1	0	0	1	0	1	1	0	*
					○	—	—	—	—	1	0	0	1	0	1	1	0	*
NSMT	M42837	7F	Stuffed	○		—	—	—	—	1	0	0	1	1	1	1	1	
					○	—	—	—	—	1	0	1	1	1	1	1	1	
NSMT	M56386	7F	Stuffed	○		—	—	—	—	1	0	0	1	0	1	1	0	*
					○	—	—	—	—	1	0	0	1	0	1	1	0	*
CBM	ZZ0006163		Stuffed	○		—	—	—	—	1	0	0	1	0	1	0	1	*
					○	—	—	—	—	1	0	0	1	0	1	1	0	*
									Sum	21	0	2	21	11	21	18	8	
									Percentage	100%	0%	10%	100%	52%	100%	86%	38%	

*Assuming that the proximal branch is RO1.

Blastocerus dichotomus

Museum or Institute	Number	Specimen type	Right	Left	SR	IFST	TR	BN	Base of antler	Brow tine	Lower beam	Frontal tine	Frontal-outer-first tine	Upper beam	Rear tine
KUGM	RM134	Antler, Part of Skull	○		○	×	○	○	1	0	1	1	1	1	1
				○	○	×	○	○	1	0	1	1	1	1	1
								Sum	2	0	2	2	2	2	2
								Percentage	100%	0%	100%	100%	100%	100%	100%

Mazama americama

Museum or Institute	Number	Specimen type	R	L	SR	IFST	TR	BN	Base of antler	Brow tine	Lower beam	Upper beam	Rear tine
KUGM	RM035	Complete skeleton	○		○	×	○	○	1	0	0	1	1
				○	○	×	○	○	1	0	0	1	1
								Sum	2	0	0	2	2
								Percentage	100%	0%	0%	100%	100%

Odocoileus virgirianus

Museum or Institute	Number	Reposited floor	Note	Specimen type	Right	Left	SR	FBSTAMI	TR	BN	Base of antler	Spraburral-front process	Brow tine	Front anterior tines	Frontal tine	Frontal-outer-1st tine	Frontal-outer-2nd tine	Upper beam	Rear tine	Upper-1st tine	Upper-2nd tine	Upper-third tine
NSMT	M00282	1F		Compete skeleton	○		○	×	○	○	1	0	0	0	0	0	0	1	1	1	1	0
						○	○	×	○	○	1	0	0	0	0	0	0	1	1	1	1	0
NSMT	M32322	7F	Some tines are impossible to be determined due to strings	Stuffing	○		—	—	—	—	1	0	0	0	0	0	0	1	1	?	?	?
						○	—	—	—	—	1	0	0	0	0	0	0	1	1	?	?	?
NSMT	M39872	7F		Stuffing	○		—	—	—	—	1	0	0	0	0	0	0	1	1	1	1	0
						○	—	—	—	—	1	0	0	0	0	0	0	1	1	1	1	0
NSMT	M00189	1F		Antler	○		○		○		1	1	0	0	0	0	0	1	1	1	1	?
						○	○		○		1	1	0	0	0	0	0	1	1	1	1	?
NSMT	M39866	7F		Stuffing	○		—	—	—	—	1	0	0	0	0	0	0	1	1	1	1	1
						○	—	—	—	—	1	0	0	0	0	0	0	1	1	1	1	1
NSMT	M32362	7F		Stuffing	○		—	—	—	—	1	0	0	0	1	0	0	1	1	1	1	0
						○	—	—	—	—	1	0	1	1	1	1	1	1	1	1	1	0
NSMT	M39836	7F	Injured	Antler	○		—	—	—	—	1	1	0	0	0	0	0	1	1	1	1	1
						○	—	—	—	—	1	1	0	0	0	0	0	1	1	1	?	?
NSMT	M39839	7F		Antler	○		—	—	—	—	1	1	0	0	0	0	0	1	1	1	1	1
						○	—	—	—	—	1	1	0	0	0	0	0	1	1	1	1	1
NSMT	M39838	7F		Antler	○		—	—	—	—	1	1	0	0	0	0	0	1	1	1	1	1
						○	—	—	—	—	1	1	0	0	0	0	0	1	1	1	1	1
NSMT	M39837	7F		Antler	○		—	—	—	—	1	0	0	0	0	0	0	1	1	1	1	1
						○	—	—	—	—	1	0	0	0	0	0	0	1	1	1	1	1
NSMT	M39844	7F		Antler	○		—	—	—	—	1	0	0	0	0	0	0	1	1	1	1	1
						○	—	—	—	—	1	0	0	0	0	0	0	1	1	1	1	1
KUGM	RM033			Antler+Skull	○		○	×	○	○	1	0	0	0	0	0	0	1	1	1	1	1
						○	○	×	○	○	1	0	0	0	0	0	0	1	1	1	1	1
KUGM	RM208			Antler+Part of skull	○						1	1	0	0	0	0	0	1	1	1	1	1
						○					1	1	0	0	0	0	0	1	1	1	1	1
KUGM	RM074			Antler+Part of skull	○						1	0	0	0	0	0	0	1	1	1	1	0
						○					1	0	0	0	0	0	0	1	1	1	1	0
KUGM	RM077			Antler+Part of skull	○						1	0	0	0	0	0	0	1	1	1	1	0
						○					Broken											
KUGM	RM078			Antler+Part of skull	○						1	0	0	0	0	0	0	1	1	0	1	1
						○					1	0	0	0	0	0	0	1	1	0	1	0
KUGM	RM117			Antler		○					1	0	0	0	0	0	0	1	1	1	1	0
KUGM	RM118			Antler+Part of skull	○						1	0	0	0	0	0	0	1	1	1	1	0
						○					1	0	0	0	0	0	0	1	1	1	1	0
KUGM	RM116			Antler+Part of skull	○						1	0	0	0	0	0	0	1	1	1	0	0
						○					1	0	0	0	0	0	0	1	1	1	0	0
KUGM	RM112			Antler+Part of skull	○						1	0	0	0	0	0	0	1	1	1	1	0
KUGM	RM115			Antler+Part of skull	○						1	0	0	0	0	0	0	1	1	1	1	0
						○					1	0	0	0	0	0	0	1	1	1	1	0
KUGM	RM'058			Antler+Part of skull	○						1	0	1	0	0	0	0	1	1	1	0	0
						○					1	0	1	0	0	0	0	1	1	1	0	0
KUGM	RM054			Antler+Part of skull	○						1	0	0	0	0	0	0	1	1	1	1	0
						○					1	0	0	0	0	0	0	1	1	1	1	0
KUGM	RM071			Antler+Part of skull	○						1	0	0	0	0	0	0	1	1	1	1	1
						○					1	0	0	0	0	0	0	1	1	1	1	1

Odocoileus vigirianus (continuted)

Museum or Institute	Number	Reposited floor	Note	Specimen type	Right	Left	SR	FBSTAMI	TR	BN	Base of antler	Spraburral-front process	Brow tine	Front anterior tines	Frontal tine	Frontal-outer-1st tine	Frontal-outer-2nd tine	Upper beam	Rear tine	Upper-1st tine	Upper-2nd tine	Upper-third tine
KUGM	RM088			Antler+Part of skull	○						1	0	0	0	0	0	0	1	1	0	0	0
						○					1	0	0	0	0	0	0	1	1	0	1	1
KUGM	RM072			Antler+Part of skull	○						1	0	0	0	0	0	0	1	1	1	1	0
						○					1	0	0	0	0	0	0	1	1	1	1	0
KUGM	RM090			Antler		○					1	0	0	0	0	0	0	1	1	1	1	1
KUGM	RM068			Antler+Part of skull	○						1	0	0	0	0	0	0	1	1	1	1	0
						○					1	0	0	0	0	0	0	1	1	1	1	0
KUGM	RM089			Antler		○					1	0	0	0	0	0	0	1	1	0	1	1
KUGM	RM075			Antler+Part of skull	○						1	0	0	0	0	0	0	1	1	0	1	0
						○					1	0	0	0	0	0	0	1	1	0	1	0
KUGM	RM'073			Antler		○					1	1	0	0	0	0	0	1	1	1	1	0
KUGM	RM057			Antler+Part of skull	○						1	0	0	0	0	0	0	1	1	0	1	1
						○					1	0	0	0	0	0	0	1	1	0	1	1
KUGM	RM064			Antler+Part of skull	○						1	0	0	0	0	0	0	1	0	?	?	?
						○					1	0	0	0	0	0	0	1	0	?	?	?
KUGM	RM059			Antler+Part of skull	○						1	0	0	0	0	0	0	1	1	0	1	1
						○					1	0	0	0	0	0	0	1	1	0	1	1
KUGM	RM066			Antler+Part of skull	○						1	0	0	0	0	0	0	1	1	1	0	0
						○					1	0	0	0	0	0	0	1	1	1	1	0
KUGM	RM067			Antler+Part of skull	○						1	0	0	0	0	0	0	1	1	1	1	1
						○					1	0	0	0	0	0	0	1	1	1	1	1
KUGM	RM062			Antler+Part of skull	○						1	0	0	0	0	0	0	1	1	1	1	0
						○					1	0	0	0	0	0	0	1	1	1	1	0
KUGM	RM141			Antler+Skull	○						1	0	0	0	0	0	0	1	1	1	1	0
						○					1	0	0	0	0	0	0	1	1	1	0	0
KUGM	RM142			Antler+Skull	○						1	0	0	0	0	0	0	1	1	1	1	0
						○					1	0	0	0	0	0	0	1	1	1	1	0
KUGM	RM034			Antler+Skull	○						1	0	0	0	0	0	0	1	1	1	1	0
						○					1	0	0	0	0	0	0	1	1	1	1	0
KUGM	RM092			Antler+Skull	○						1	0	0	0	0	0	0	1	1	1	1	1
						○					1	0	0	0	0	0	0	1	1	1	1	1
KUGM	RM136			Antler+Skull	○						1	0	0	0	0	0	0	1	1	1	1	1
						○					1	0	0	0	0	0	0	1	1	1	1	1
KUGM	RM079			Antler+Skull	○						1	0	0	0	0	0	0	1	1	1	0	0
						○					1	0	0	0	0	0	0	1	1	1	0	0
KUGM	RM069		Juvenile?	Antler+Skull	○						1	0	0	0	0	0	0	1	1	1	0	0
						○					1	0	0	0	0	0	0	1	1	1	0	0
KUGM	RM113		Juvenile?	Antler+Part of skull	○						1	0	0	0	0	0	0	1	1	0	0	0
						○					1	0	0	0	0	0	0	1	1	0	0	0
KUGM	RM'061		Juvenile?	Antler+Part of skull	○						1	0	0	0	0	0	0	1	1	1	0	0
						○					1	0	0	0	0	0	0	1	1	1	0	0
KUGM	RM060		Juvenile?	Antler+Part of skull	○						1	0	0	0	0	0	0	1	1	1	0	0
						○					1	1	1	0	0	0	0	1	1	1	0	0
KUGM	RM070		Juvenile?	Antler+Part of skull	○						1	0	0	0	0	0	0	1	1	1	0	0
						○					1	0	0	0	0	0	0	1	1	1	0	0
KUGM	RM065		Juvenile?	Antler+Part of skull	○						1	0	0	0	0	0	0	1	1	1	0	0
						○					1	0	0	0	0	0	0	1	1	1	0	0
KUGM	RM144		Juvenile?	Antler+Part of skull	○						1	0	0	0	0	0	0	1	1	1	0	0
						○					1	0	0	0	0	0	0	1	1	1	?	?

Odocoileus vigirianus (continuted)

Museum or Institute	Number	Reposited floor	Note	Specimen type	Right	Left	SR	IFST	TR	BN	Base of antler	Spraburral-front process	Brow tine	Front anterior tines	Frontal tine	Frontal-outer-1st tine	Frontal-outer-2nd tine	Upper beam	Rear tine	Upper-1st tine	Upper-2nd tine	Upper-third tine
KUGM	RM119		Juvenile?	Antler+Part of skull	○						1	0	0	0	0	0	0	1	1	0	0	0
						○					1	0	0	0	0	0	0	1	1	1	0	0
KUGM	RM063		Juvenile?	Antler+Part of skull	○						1	0	0	0	0	0	0	1	1	1	0	0
						○					1	0	0	0	0	0	0	1	1	1	0	0
KUGM	RM111		Juvenile?	Antler+Part of skull	○						1	0	0	0	0	0	0	0	1	0	0	0
						○					1	0	0	0	0	0	0	1	1	1	0	0
KUGM	RM123		Juvenile?	Antler+Part of skull		○					1	0	0	0	0	0	0	1	1	0	1	0
KUGM	RM114		Juvenile	Antler+Part of skull	○						1	0	0	0	0	0	0	1	1	0	0	0
						○					1	0	0	0	0	0	0	1	1	0	0	0
KUGM	RM120		Juvenile	Antler+Part of skull	○						1	0	0	0	0	0	0	1	0	0	0	0
						○					1	0	0	0	0	0	0	1	0	0	0	0
KUGM	RM121		Juvenile	Antler+Part of skull	○						1	0	0	0	0	0	0	0	0	0	0	0
						○					1	0	0	0	0	0	0	0	0	0	0	0
KUGM	RM122		Juvenile	Antler+Part of skull	○						1	0	0	0	0	0	0	1	1	1	0	0
						○					1	0	0	0	0	0	0	1	1	0	0	0
KUGM	RM110		Juvenile	Antler+Part of skull	○						1	1	0	0	0	0	0	1	0	0	0	0
						○					1	1	0	0	0	0	0	1	0	0	0	0
Sum (excluding ”Juvenile” and "Juvenile?")											82	12	3	1	2	1	1	82	80	67	68	33
Percentage (excluding ”Juvenile” and "Juvenile?")											100%	15%	4%	1%	2%	1%	1%	100%	98%	86%	88%	44%
