# Supplementary material for: A new viewpoint on antlers reveals the evolutionary history of deer (Cervidae, Mammalia)
Source: Sci Rep. 2020 Jun 2;10:8910. doi: 10.1038/s41598-020-64555-7 (PMC7265483; doi:10.1038/s41598-020-64555-7)
Supplement: Supplementary file 6 — Supplementary information 6 - Matrix of existence of the elements of all the specimens observed in this study. [file 41598_2020_64555_MOESM6_ESM.pdf]

A new point of view on antlers reveals the evolutionary history of deer (Cervidae, Mammalia)

Yuusuke Samejima & Hiroshige Matsuoka

Supplemantary Information 7

Ancestral reconstruction of each homologous element on the fixed topology

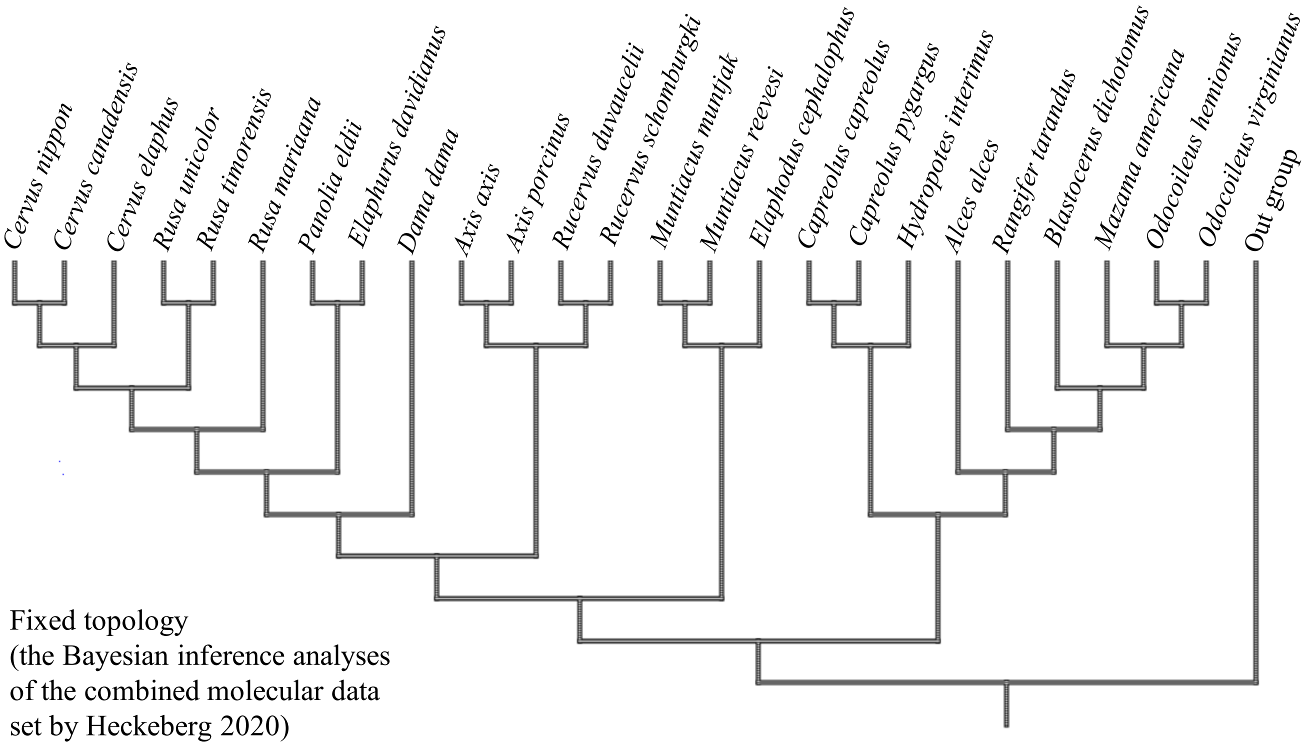

Fixed topology
(the Bayesian inference analyses
of the combined molecular data
set by Heckeberg 2020)

Character state reconstruction of the elements in adults on the fixed topology are shown in the following pages.

The characters states of the terminal nodes are by observed percentages in this study, and those of the inner nodes are by reconstruction.

○ 0：0% (None)

● 1：Less than 80% (Not typical)

● 2：More than 80% (Typical)

Percentage of each element is shown in Table 2 of the article.

The reconstructed is by Dollo parsimony between character state 0 and 1, and by standard parsimony (ACCTRAN) between character state 1 and 2. The fixed topology is from the Bayesian inference analyses of combined molecular data set by Heckeberg (2020).

The figures are made by Mesquite ver.3.61 and partly modified by hand.

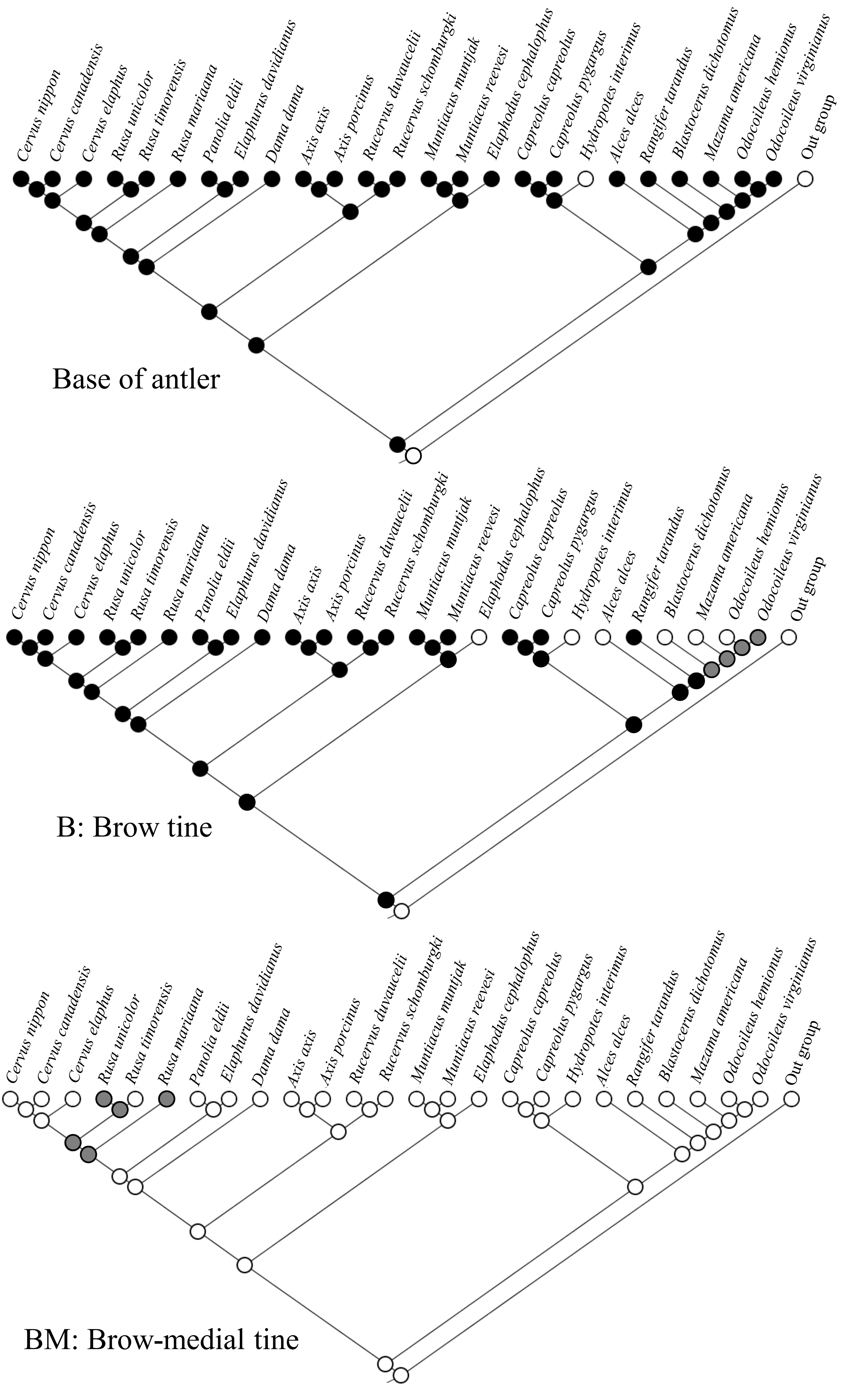
Cervus nippon
Cervus canadensis
Cervus elaphus
Rusa unicolor
Rusa timorensis
Rusa mariaana
Panolia eldii
Elaphurus davidianus
Dama dama
Axis axis
Axis porcinus
Rucervus duvaucelii
Rucervus schomburgki
Muntiacus muntjak
Muntiacus reevesi
Elaphodus cephalophus
Capreolus capreolus
Capreolus pygargus
Hydropotes interimus
Alces alces
Rangifer tarandus
Blastocerus dichotomus
Mazama americana
Odocoileus hemionus
Odocoileus virginianus
Out group
Base of antler
Cervus nippon
Cervus canadensis
Cervus elaphus
Rusa unicolor
Rusa timorensis
Rusa mariaana
Panolia eldii
Elaphurus davidianus
Dama dama
Axis axis
Axis porcinus
Rucervus duvaucelii
Rucervus schomburgki
Muntiacus muntjak
Muntiacus reevesi
Elaphodus cephalophus
Capreolus capreolus
Capreolus pygargus
Hydropotes interimus
Alces alces
Rangifer tarandus
Blastocerus dichotomus
Mazama americana
Odocoileus hemionus
Odocoileus virginianus
Out group
B: Brow tine
Cervus nippon
Cervus canadensis
Cervus elaphus
Rusa unicolor
Rusa timorensis
Rusa mariaana
Panolia eldii
Elaphurus davidianus
Dama dama
Axis axis
Axis porcinus
Rucervus duvaucelii
Rucervus schomburgki
Muntiacus muntjak
Muntiacus reevesi
Elaphodus cephalophus
Capreolus capreolus
Capreolus pygargus
Hydropotes interimus
Alces alces
Rangifer tarandus
Blastocerus dichotomus
Mazama americana
Odocoileus hemionus
Odocoileus virginianus
Out group
BM: Brow-medial tine

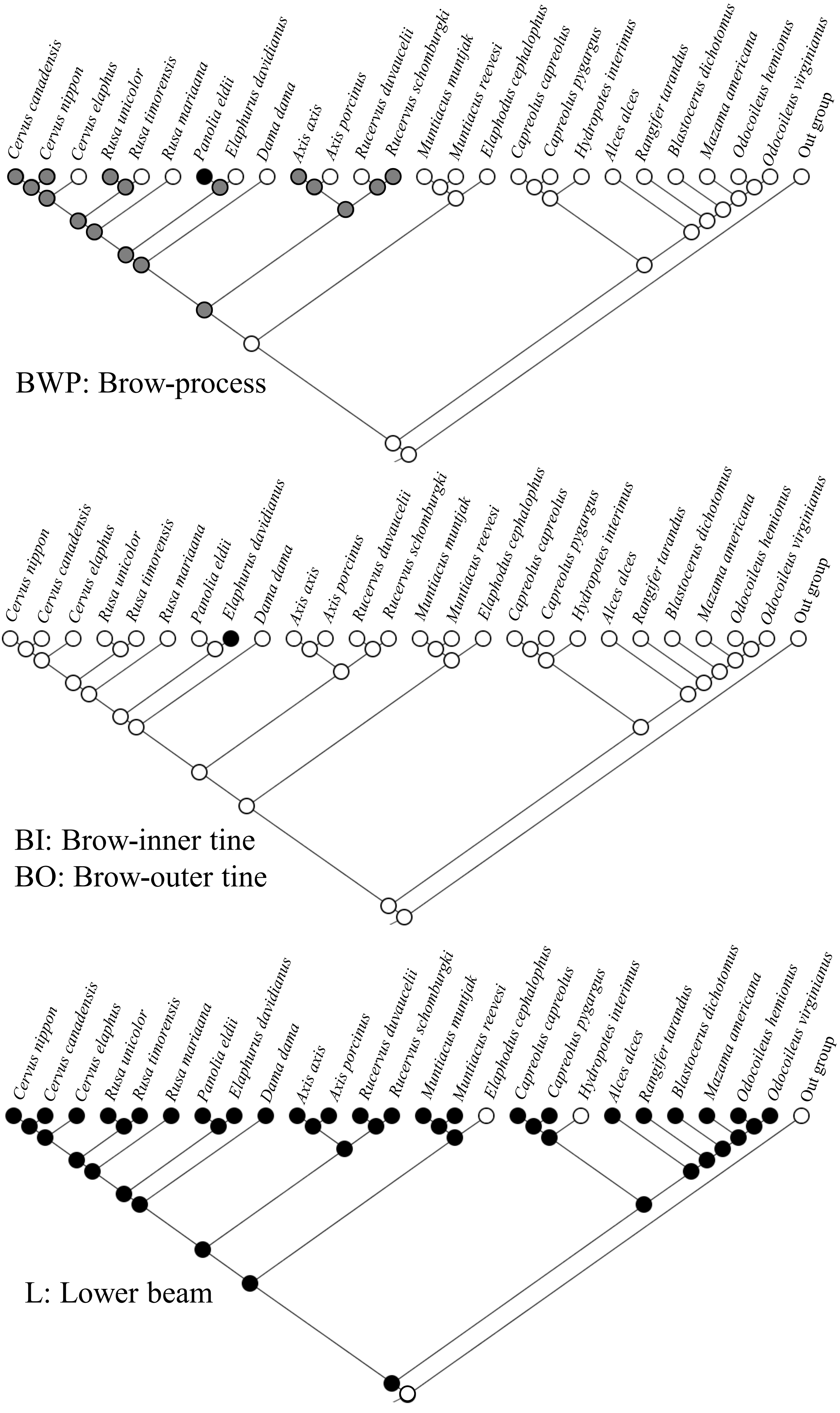
Cervus canadensis
Cervus nippon
Cervus elaphus
Rusa unicolor
Rusa timorensis
Rusa mariaana
Panolia eldii
Elaphurus davidianus
Dama dama
Axis axis
Axis porcinus
Rucervus duvaucelii
Rucervus schomburgki
Muntiacus muntjak
Muntiacus reevesi
Elaphodus cephalophus
Capreolus capreolus
Capreolus pygargus
Hydropotes interimus
Alces alces
Rangifer tarandus
Blastocerus dichotomus
Mazama americana
Odocoileus hemionus
Odocoileus virginianus
Out group
BWP: Brow-process
Cervus nippon
Cervus canadensis
Cervus elaphus
Rusa unicolor
Rusa timorensis
Rusa mariaana
Panolia eldii
Elaphurus davidianus
Dama dama
Axis axis
Axis porcinus
Rucervus duvaucelii
Rucervus schomburgki
Muntiacus muntjak
Muntiacus reevesi
Elaphodus cephalophus
Capreolus capreolus
Capreolus pygargus
Hydropotes interimus
Alces alces
Rangifer tarandus
Blastocerus dichotomus
Mazama americana
Odocoileus hemionus
Odocoileus virginianus
Out group
BI: Brow-inner tine
BO: Brow-outer tine
Cervus nippon
Cervus canadensis
Cervus elaphus
Rusa unicolor
Rusa timorensis
Rusa mariaana
Panolia eldii
Elaphurus davidianus
Dama dama
Axis axis
Axis porcinus
Rucervus duvaucelii
Rucervus schomburgki
Muntiacus muntjak
Muntiacus reevesi
Elaphodus cephalophus
Capreolus capreolus
Capreolus pygargus
Hydropotes interimus
Alces alces
Rangifer tarandus
Blastocerus dichotomus
Mazama americana
Odocoileus hemionus
Odocoileus virginianus
Out group
L: Lower beam

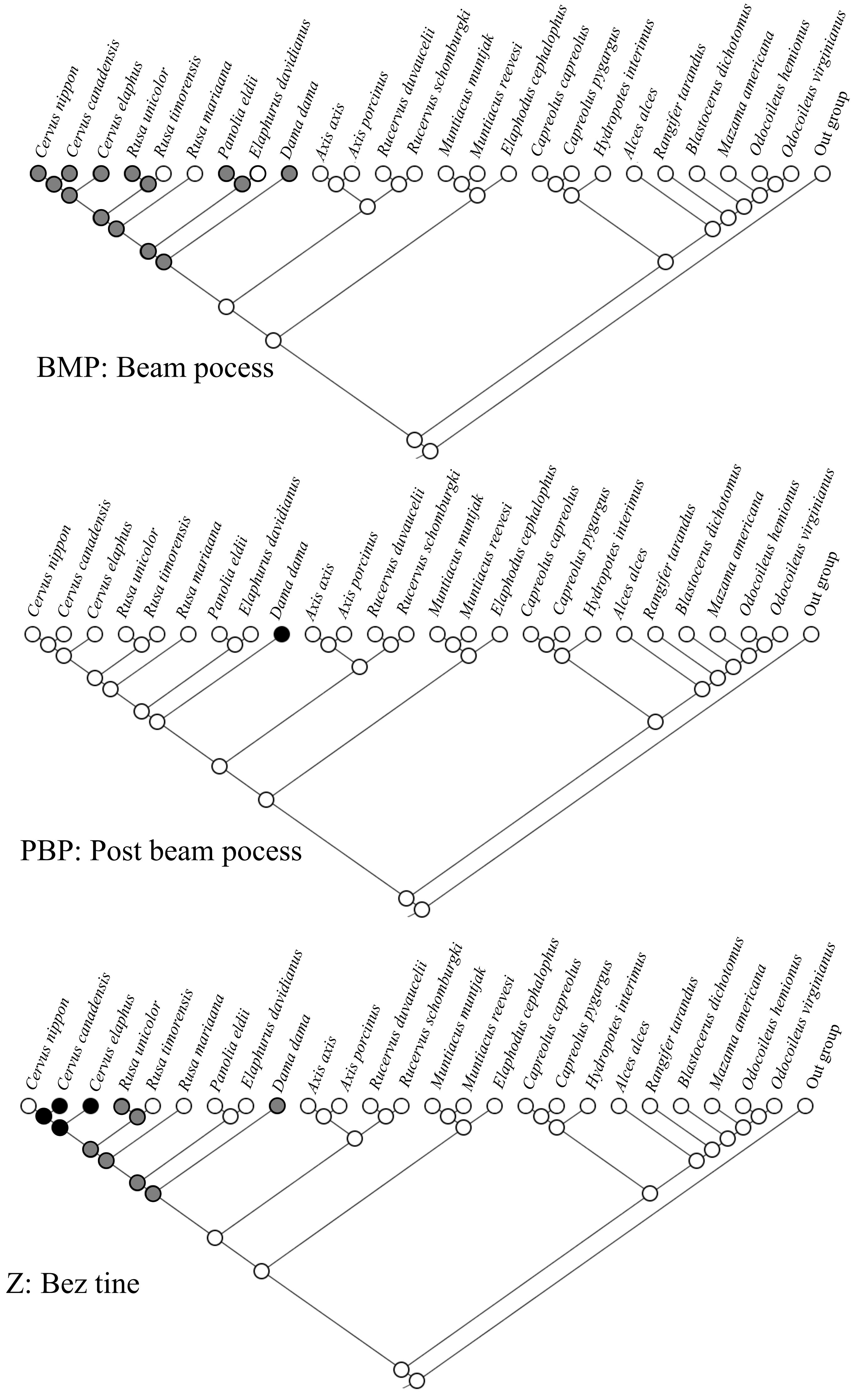
Cervus nippon
Cervus canadensis
Cervus elaphus
Rusa unicolor
Rusa timorensis
Rusa mariaana
Panolia eldii
Elaphurus davidianus
Dama dama
Axis axis
Axis porcinus
Rucervus duvaucelii
Rucervus schomburgki
Muntiacus muntjak
Muntiacus reevesi
Elaphodus cephalophus
Capreolus capreolus
Capreolus pygargus
Hydropotes interimus
Alces alces
Rangifer tarandus
Blastocerus dichotomus
Mazama americana
Odocoileus hemionus
Odocoileus virginianus
Out group
BMP: Beam pocess
Cervus nippon
Cervus canadensis
Cervus elaphus
Rusa unicolor
Rusa timorensis
Rusa mariaana
Panolia eldii
Elaphurus davidianus
Dama dama
Axis axis
Axis porcinus
Rucervus duvaucelii
Rucervus schomburgki
Muntiacus muntjak
Muntiacus reevesi
Elaphodus cephalophus
Capreolus capreolus
Capreolus pygargus
Hydropotes interimus
Alces alces
Rangifer tarandus
Blastocerus dichotomus
Mazama americana
Odocoileus hemionus
Odocoileus virginianus
Out group
PBP: Post beam pocess
Cervus nippon
Cervus canadensis
Cervus elaphus
Rusa unicolor
Rusa timorensis
Rusa mariaana
Panolia eldii
Elaphurus davidianus
Dama dama
Axis axis
Axis porcinus
Rucervus duvaucelii
Rucervus schomburgki
Muntiacus muntjak
Muntiacus reevesi
Elaphodus cephalophus
Capreolus capreolus
Capreolus pygargus
Hydropotes interimus
Alces alces
Rangifer tarandus
Blastocerus dichotomus
Mazama americana
Odocoileus hemionus
Odocoileus virginianus
Out group
Z: Bez tine

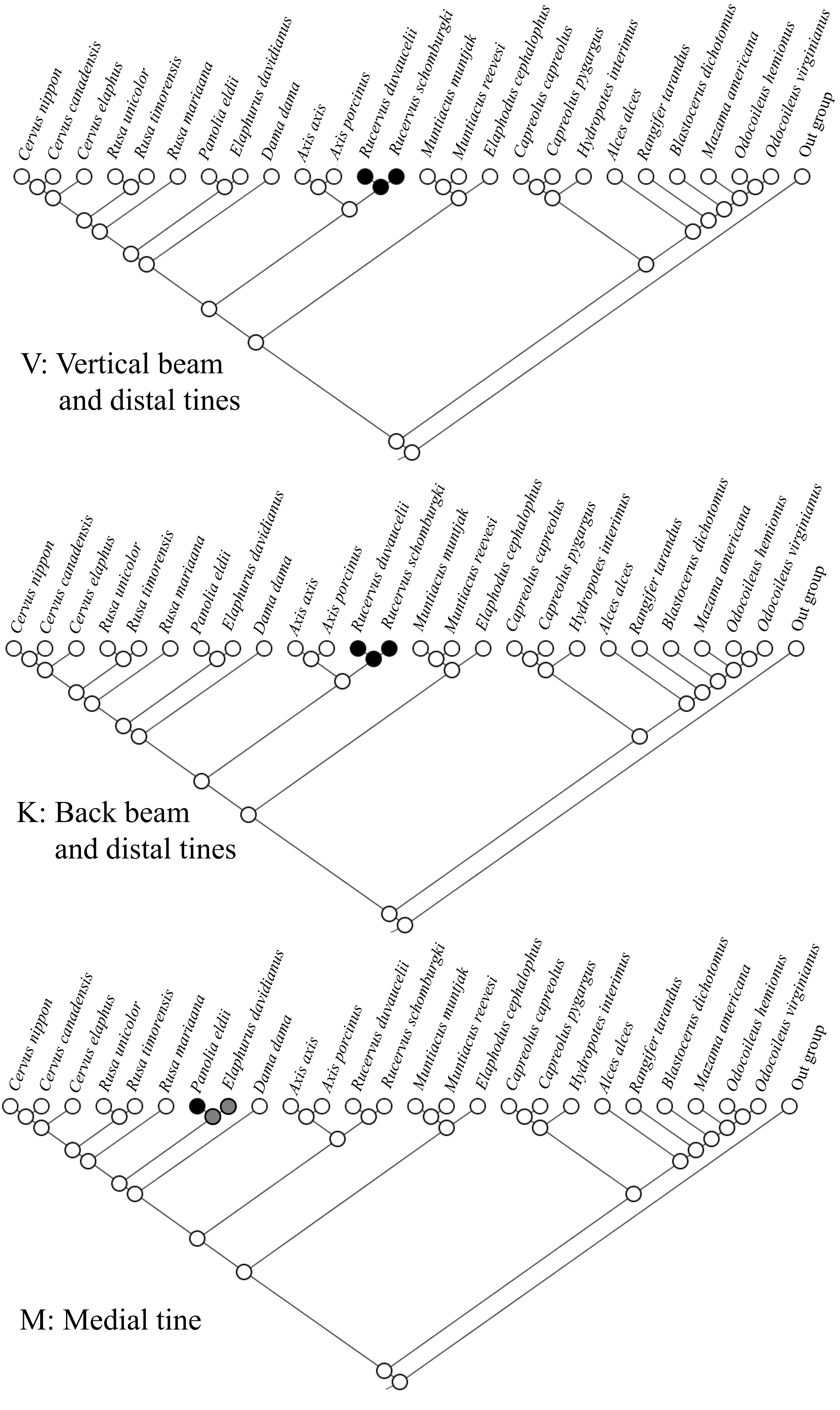
Cervus nippon
Cervus canadensis
Cervus elaphus
Rusa unicolor
Rusa timorensis
Rusa mariaana
Panolia eldii
Elaphurus davidianus
Dama dama
Axis axis
Axis porcinus
Rucervus duvaucelii
Rucervus schomburgki
Muntiacus muntjak
Muntiacus reevesi
Elaphodus cephalophus
Capreolus capreolus
Capreolus pygargus
Hydropotes interimus
Alces alces
Rangifer tarandus
Blastocerus dichotomus
Mazama americana
Odocoileus hemionus
Odocoileus virginianus
Out group
V: Vertical beam
and distal tines
Cervus nippon
Cervus canadensis
Cervus elaphus
Rusa unicolor
Rusa timorensis
Rusa mariaana
Panolia eldii
Elaphurus davidianus
Dama dama
Axis axis
Axis porcinus
Rucervus duvaucelii
Rucervus schomburgki
Muntiacus muntjak
Muntiacus reevesi
Elaphodus cephalophus
Capreolus capreolus
Capreolus pygargus
Hydropotes interimus
Alces alces
Rangifer tarandus
Blastocerus dichotomus
Mazama americana
Odocoileus hemionus
Odocoileus virginianus
Out group
K: Back beam
and distal tines
Cervus nippon
Cervus canadensis
Cervus elaphus
Rusa unicolor
Rusa timorensis
Rusa mariaana
Panolia eldii
Elaphurus davidianus
Dama dama
Axis axis
Axis porcinus
Rucervus duvaucelii
Rucervus schomburgki
Muntiacus muntjak
Muntiacus reevesi
Elaphodus cephalophus
Capreolus capreolus
Capreolus pygargus
Hydropotes interimus
Alces alces
Rangifer tarandus
Blastocerus dichotomus
Mazama americana
Odocoileus hemionus
Odocoileus virginianus
Out group
M: Medial tine

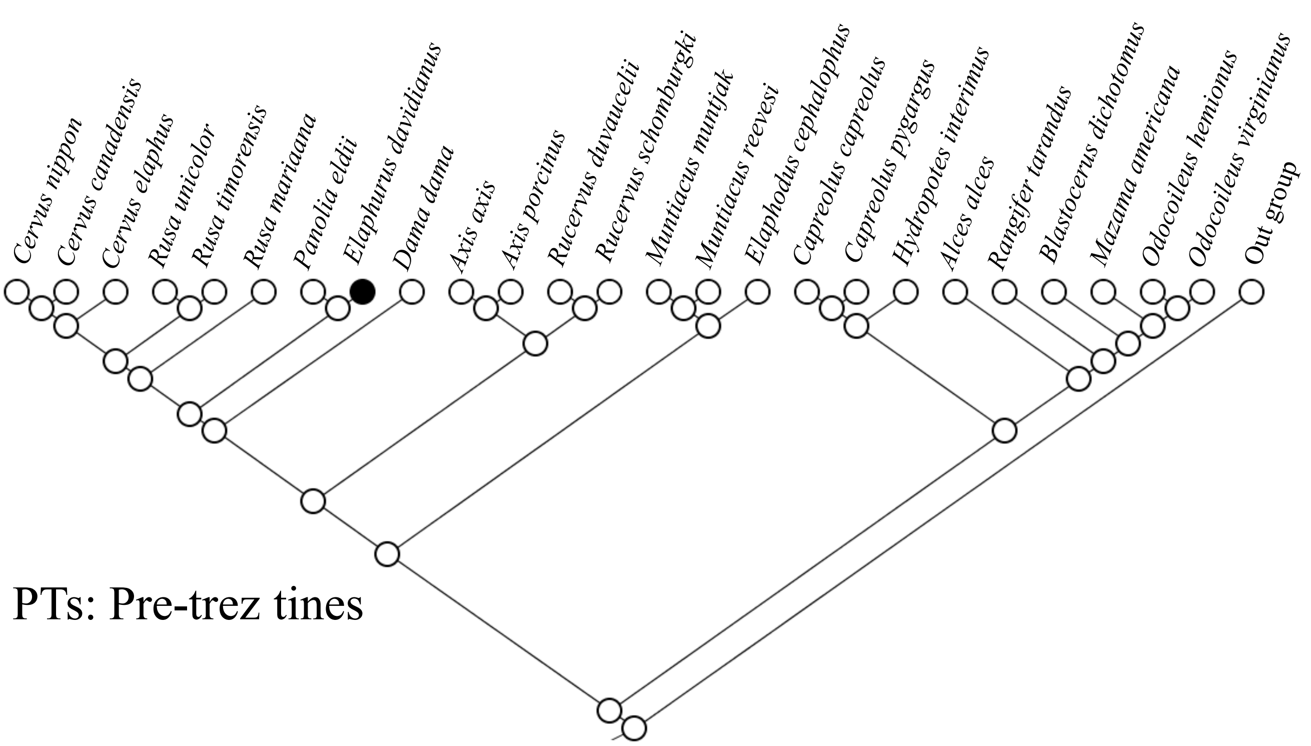

PTs: Pre-trez tines

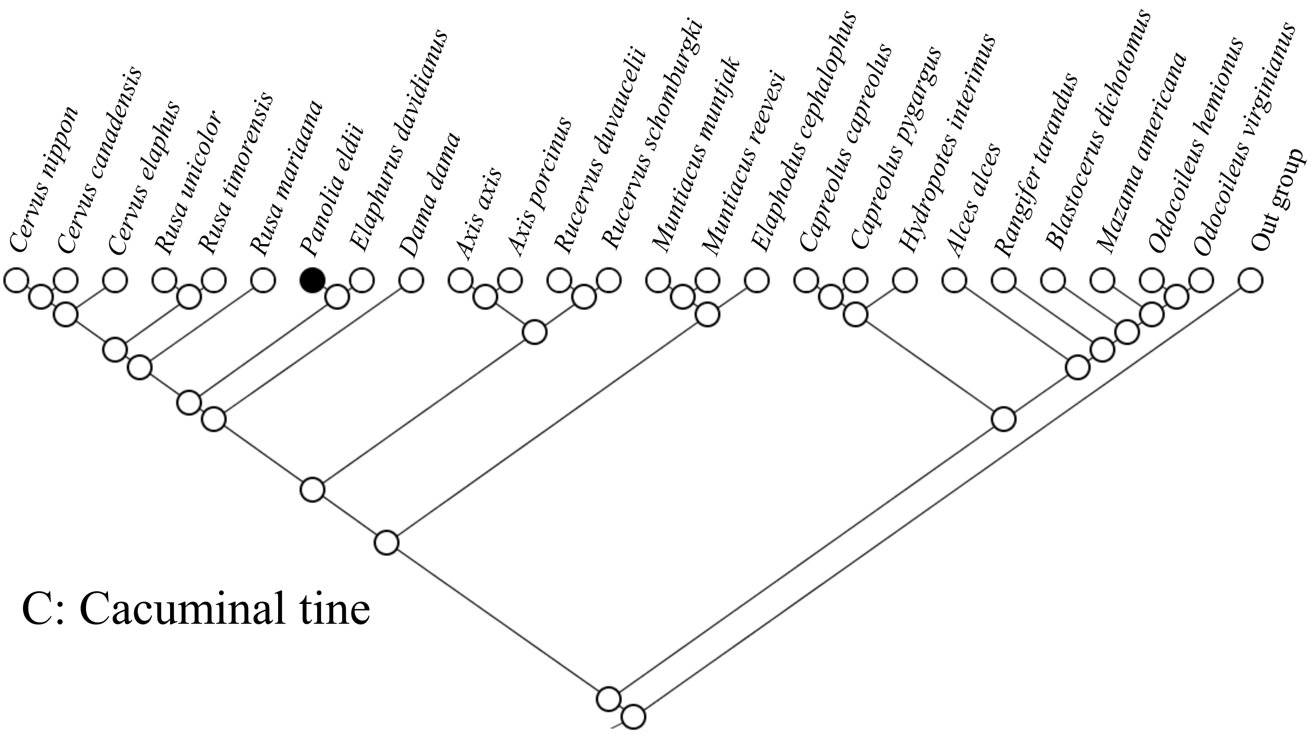

C: Cacuminal tine

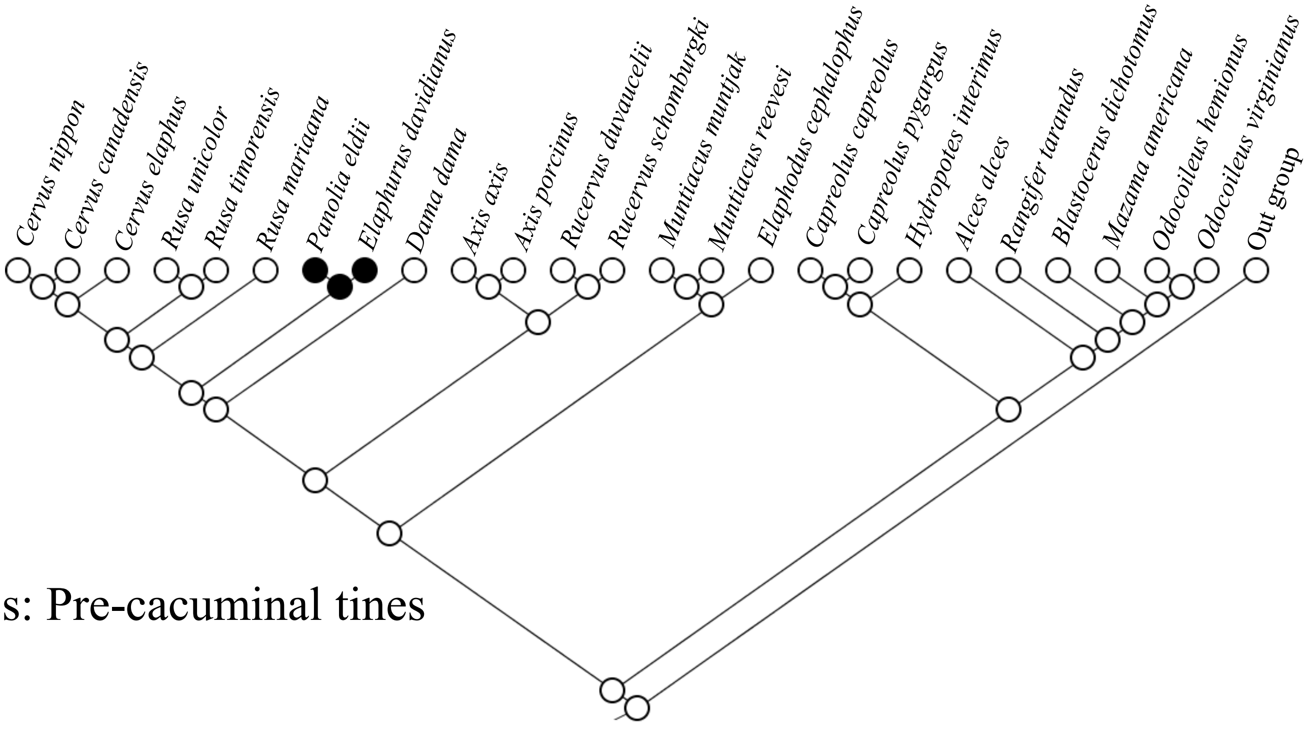

PCs: Pre-cacuminal tines

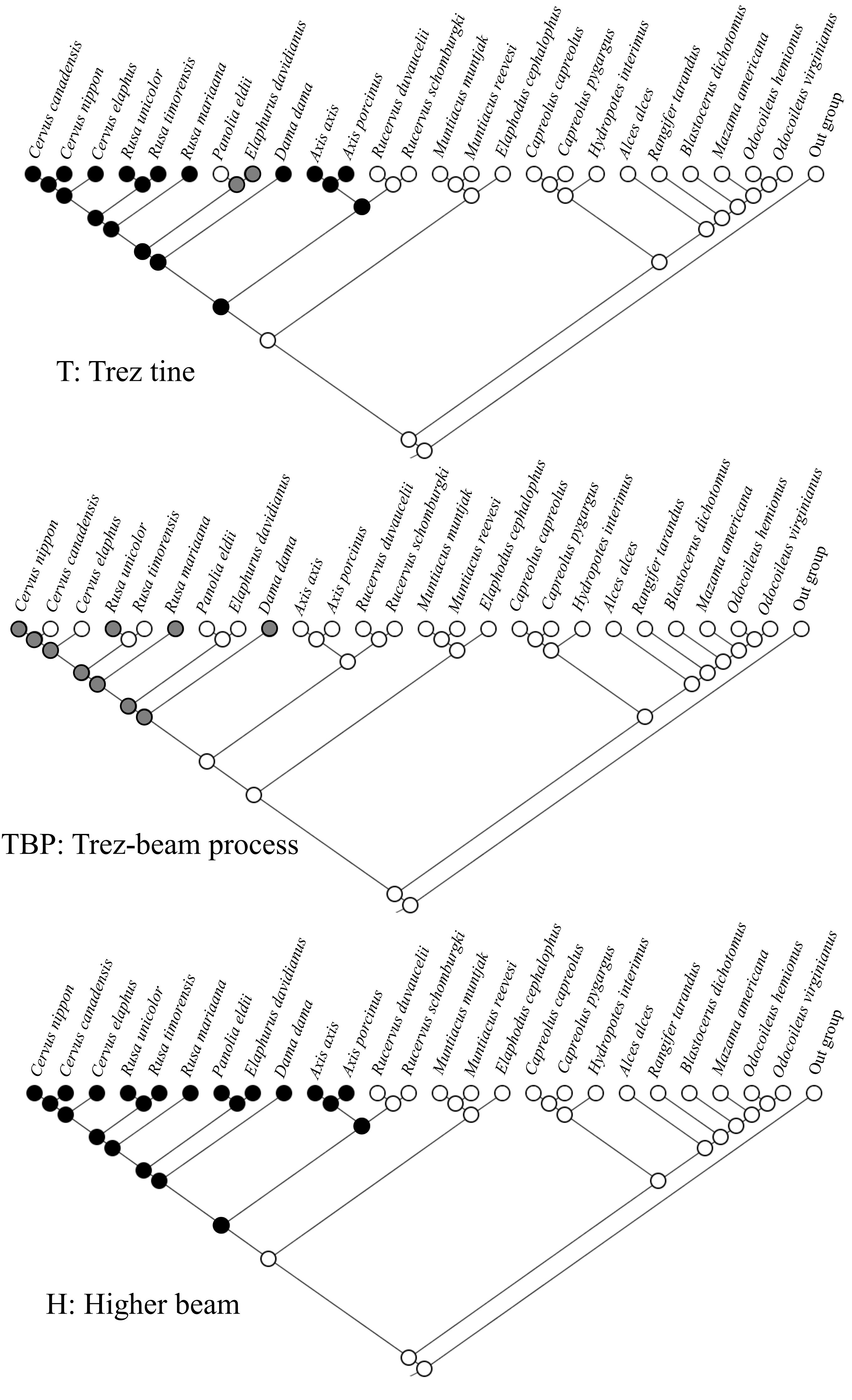
Cervus canadensis
Cervus nippon
Cervus elaphus
Rusa unicolor
Rusa timorensis
Rusa mariaana
Panolia eldii
Elaphurus davidianus
Dama dama
Axis axis
Axis porcinus
Rucervus duvaucelii
Rucervus schomburgki
Muntiacus muntjak
Muntiacus reevesi
Elaphodus cephalophus
Capreolus capreolus
Capreolus pygargus
Hydropotes interimus
Alces alces
Rangifer tarandus
Blastocerus dichotomus
Mazama americana
Odocoileus hemionus
Odocoileus virginianus
Out group
T: Trez tine
Cervus nippon
Cervus canadensis
Cervus elaphus
Rusa unicolor
Rusa timorensis
Rusa mariaana
Panolia eldii
Elaphurus davidianus
Dama dama
Axis axis
Axis porcinus
Rucervus duvaucelii
Rucervus schomburgki
Muntiacus muntjak
Muntiacus reevesi
Elaphodus cephalophus
Capreolus capreolus
Capreolus pygargus
Hydropotes interimus
Alces alces
Rangifer tarandus
Blastocerus dichotomus
Mazama americana
Odocoileus hemionus
Odocoileus virginianus
Out group
TBP: Trez-beam process
Cervus nippon
Cervus canadensis
Cervus elaphus
Rusa unicolor
Rusa timorensis
Rusa mariaana
Panolia eldii
Elaphurus davidianus
Dama dama
Axis axis
Axis porcinus
Rucervus duvaucelii
Rucervus schomburgki
Muntiacus muntjak
Muntiacus reevesi
Elaphodus cephalophus
Capreolus capreolus
Capreolus pygargus
Hydropotes interimus
Alces alces
Rangifer tarandus
Blastocerus dichotomus
Mazama americana
Odocoileus hemionus
Odocoileus virginianus
Out group
H: Higher beam

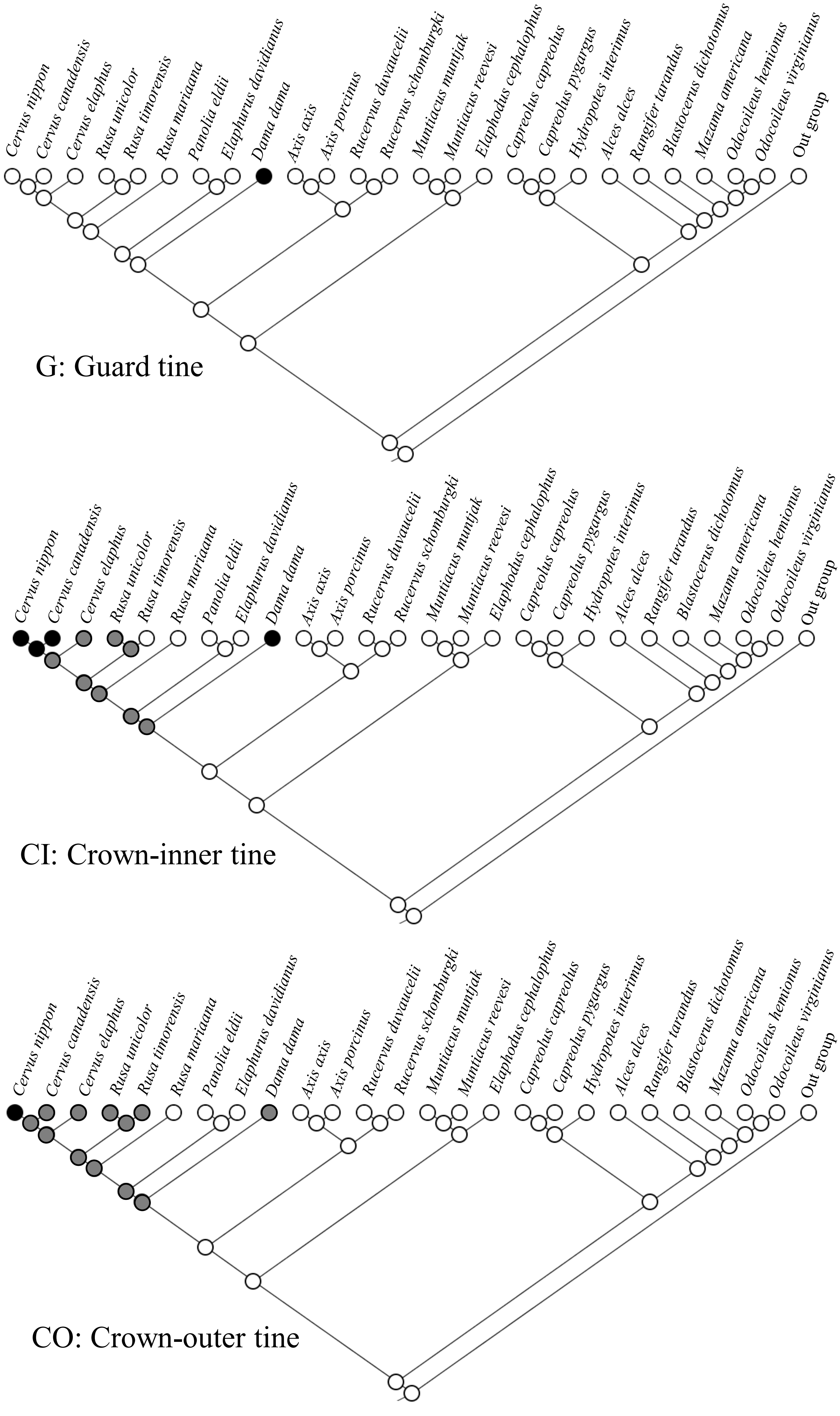
Cervus nippon
Cervus canadensis
Cervus elaphus
Rusa unicolor
Rusa timorensis
Rusa mariaana
Panolia eldii
Elaphurus davidianus
Dama dama
Axis axis
Axis porcinus
Rucervus duvaucelii
Rucervus schomburgki
Muntiacus muntjak
Muntiacus reevesi
Elaphodus cephalophus
Capreolus capreolus
Capreolus pygargus
Hydropotes interimus
Alces alces
Rangifer tarandus
Blastocerus dichotomus
Mazama americana
Odocoileus hemionus
Odocoileus virginianus
Out group
G: Guard tine
Cervus nippon
Cervus canadensis
Cervus elaphus
Rusa unicolor
Rusa timorensis
Rusa mariaana
Panolia eldii
Elaphurus davidianus
Dama dama
Axis axis
Axis porcinus
Rucervus duvaucelii
Rucervus schomburgki
Muntiacus muntjak
Muntiacus reevesi
Elaphodus cephalophus
Capreolus capreolus
Capreolus pygargus
Hydropotes interimus
Alces alces
Rangifer tarandus
Blastocerus dichotomus
Mazama americana
Odocoileus hemionus
Odocoileus virginianus
Out group
CI: Crown-inner tine
Cervus nippon
Cervus canadensis
Cervus elaphus
Rusa unicolor
Rusa timorensis
Rusa mariaana
Panolia eldii
Elaphurus davidianus
Dama dama
Axis axis
Axis porcinus
Rucervus duvaucelii
Rucervus schomburgki
Muntiacus muntjak
Muntiacus reevesi
Elaphodus cephalophus
Capreolus capreolus
Capreolus pygargus
Hydropotes interimus
Alces alces
Rangifer tarandus
Blastocerus dichotomus
Mazama americana
Odocoileus hemionus
Odocoileus virginianus
Out group
CO: Crown-outer tine

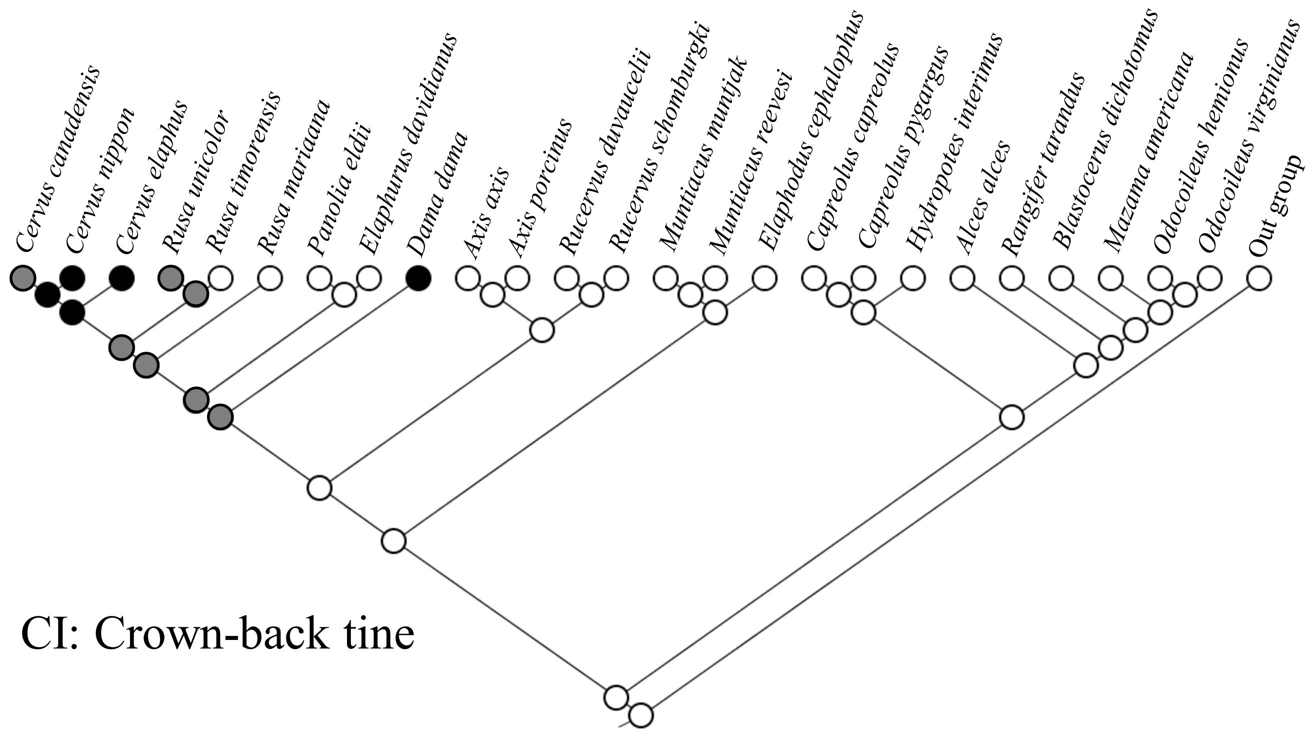
Cervus canadensis
Cervus nippon
Cervus elaphus
Rusa unicolor
Rusa timorensis
Rusa mariaana
Panolia eldii
Elaphurus davidianus
Dama dama
Axis axis
Axis porcinus
Rucervus duvaucelii
Rucervus schomburgki
Muntiacus muntjak
Muntiacus reevesi
Elaphodus cephalophus
Capreolus capreolus
Capreolus pygargus
Hydropotes interimus
Alces alces
Rangifer tarandus
Blastocerus dichotomus
Mazama americana
Odocoileus hemionus
Odocoileus virginianus
Out group
CI: Crown-back tine

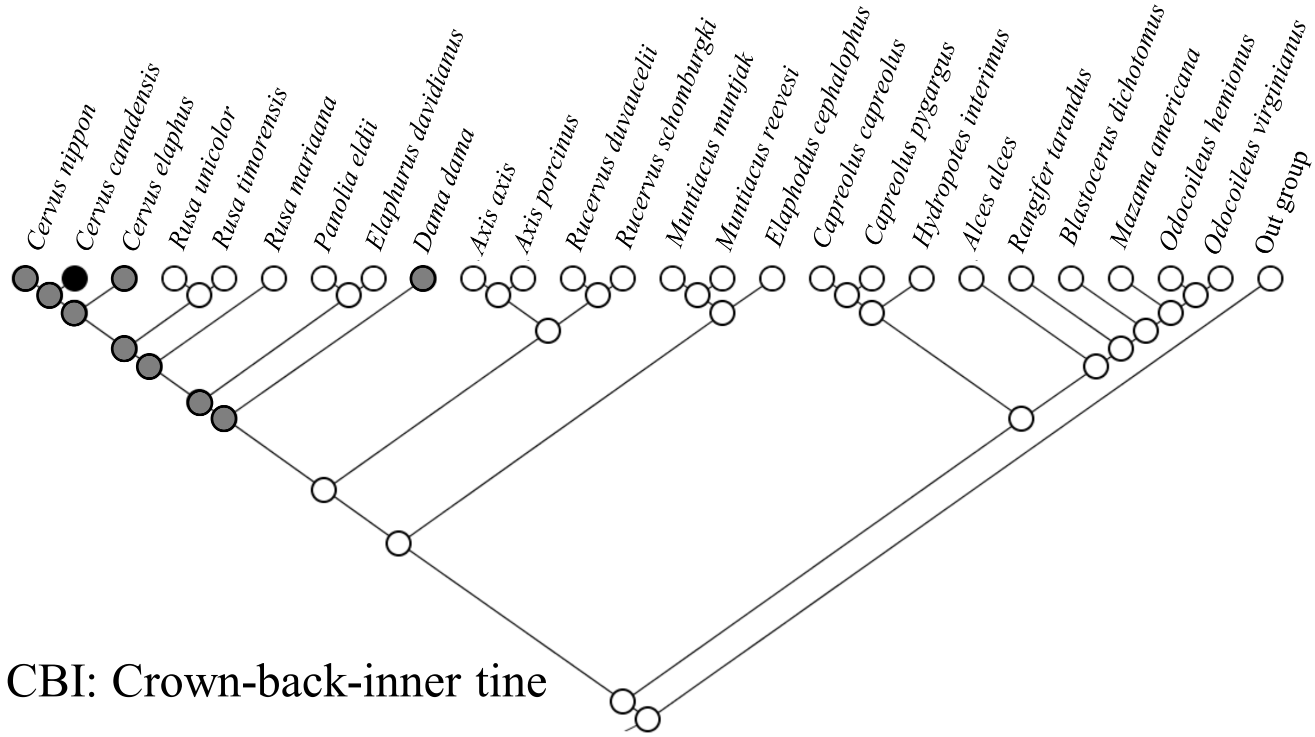
Cervus nippon
Cervus canadensis
Cervus elaphus
Rusa unicolor
Rusa timorensis
Rusa mariaana
Panolia eldii
Elaphurus davidianus
Dama dama
Axis axis
Axis porcinus
Rucervus duvaucelii
Rucervus schomburgki
Muntiacus muntjak
Muntiacus reevesi
Elaphodus cephalophus
Capreolus capreolus
Capreolus pygargus
Hydropotes interimus
Alces alces
Rangifer tarandus
Blastocerus dichotomus
Mazama americana
Odocoileus hemionus
Odocoileus virginianus
Out group
CBI: Crown-back-inner tine

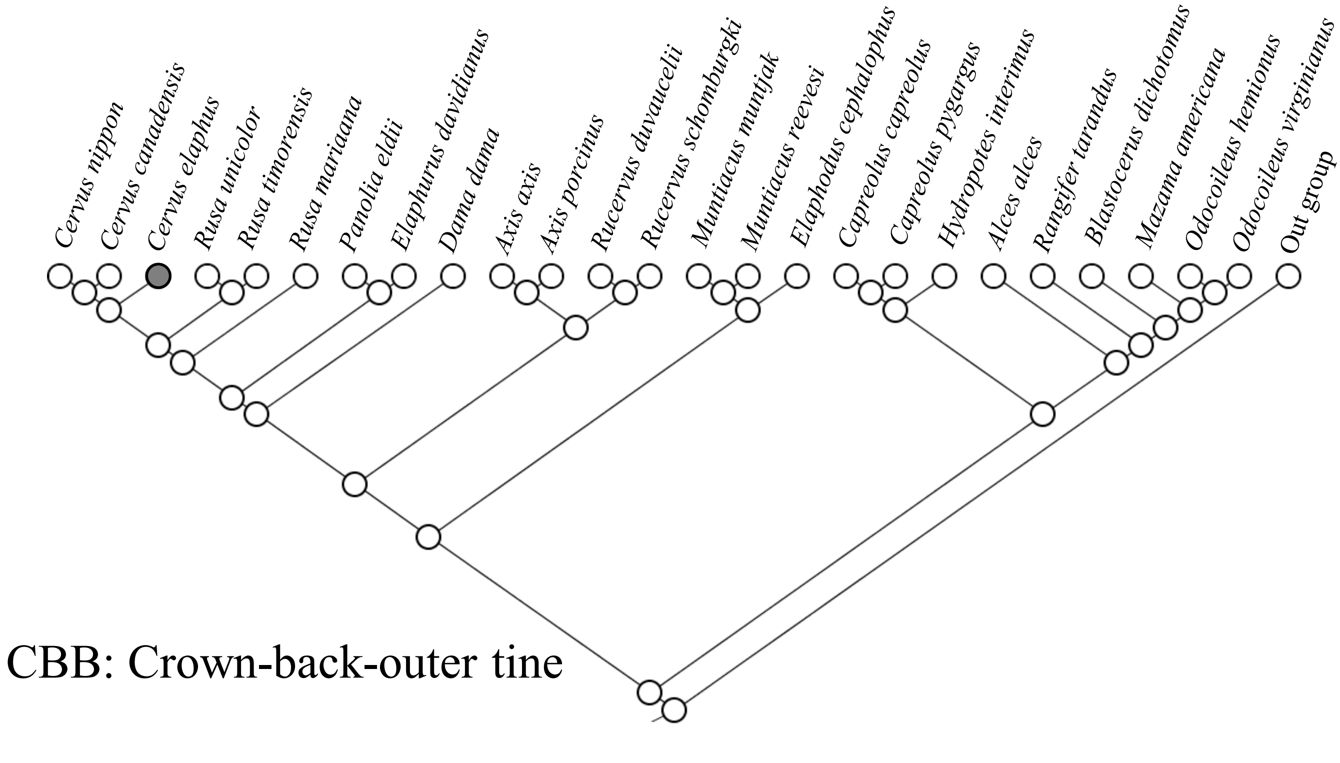
Cervus nippon
Cervus canadensis
Cervus elaphus
Rusa unicolor
Rusa timorensis
Rusa mariaana
Panolia eldii
Elaphurus davidianus
Dama dama
Axis axis
Axis porcinus
Rucervus duvaucelii
Rucervus schomburgki
Muntiacus muntjak
Muntiacus reevesi
Elaphodus cephalophus
Capreolus capreolus
Capreolus pygargus
Hydropotes interimus
Alces alces
Rangifer tarandus
Blastocerus dichotomus
Mazama americana
Odocoileus hemionus
Odocoileus virginianus
Out group
CBB: Crown-back-outer tine

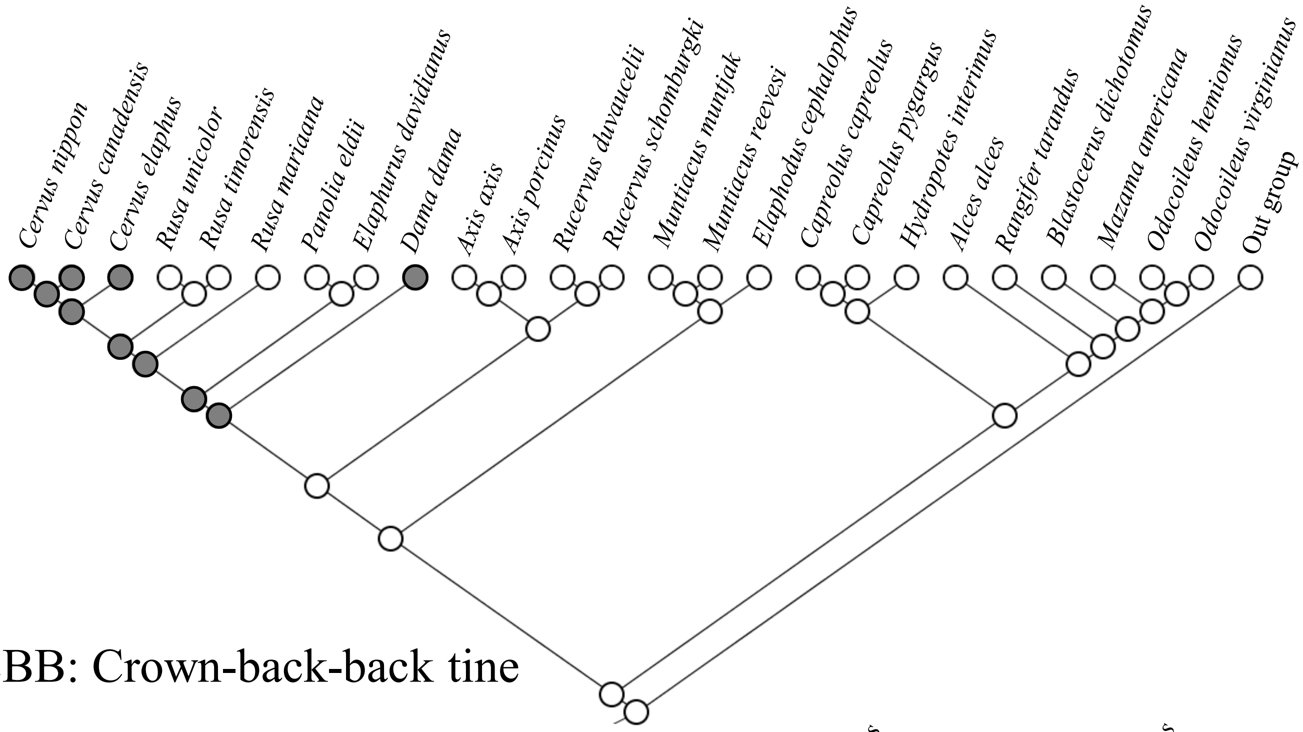

CBB: Crown-back-back tine

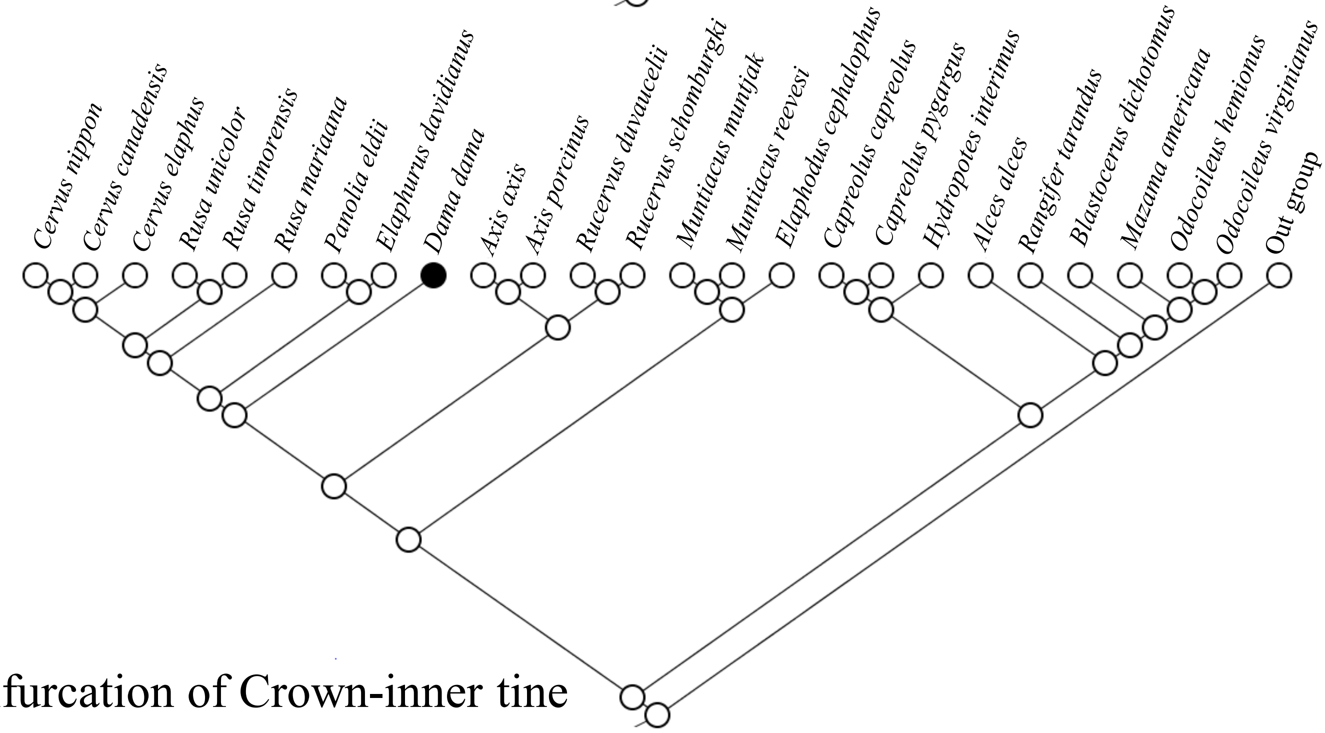

Bifurcation of Crown-inner tine

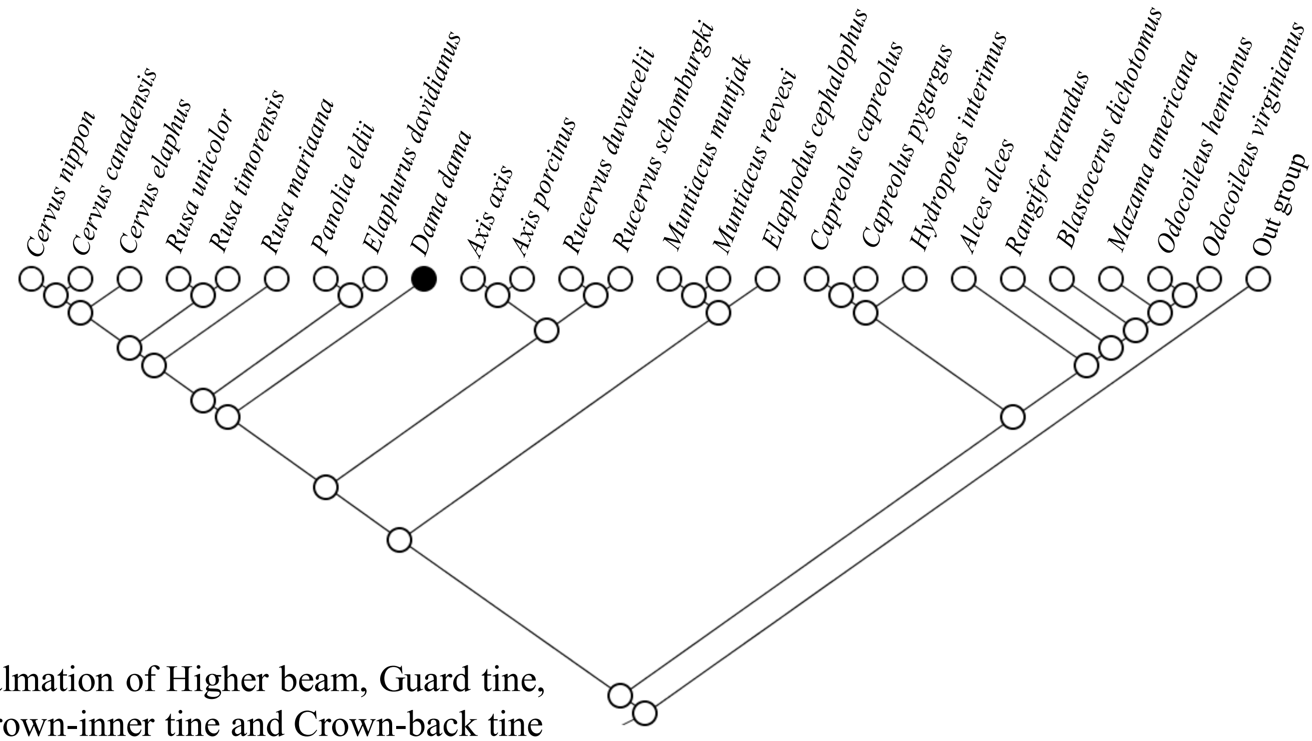

Palmation of Higher beam, Guard tine,
Crown-inner tine and Crown-back tine

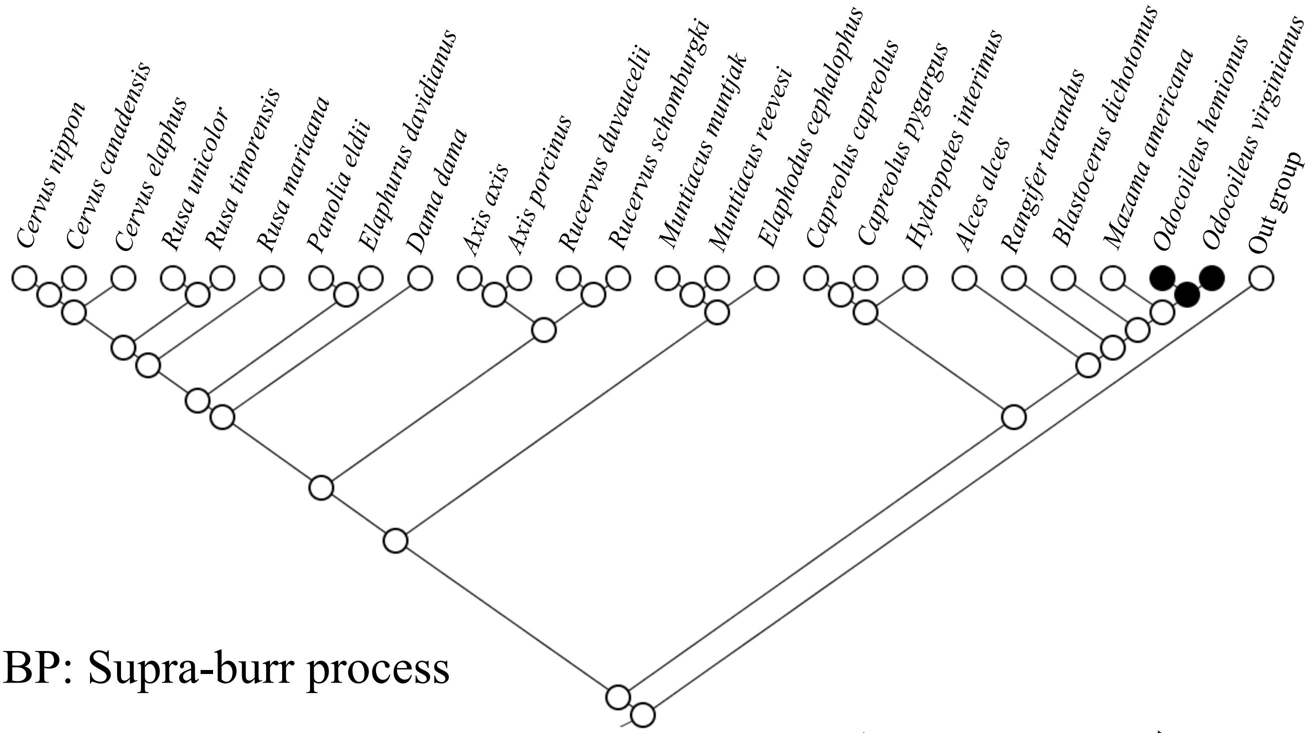

SBP: Supra-burr process

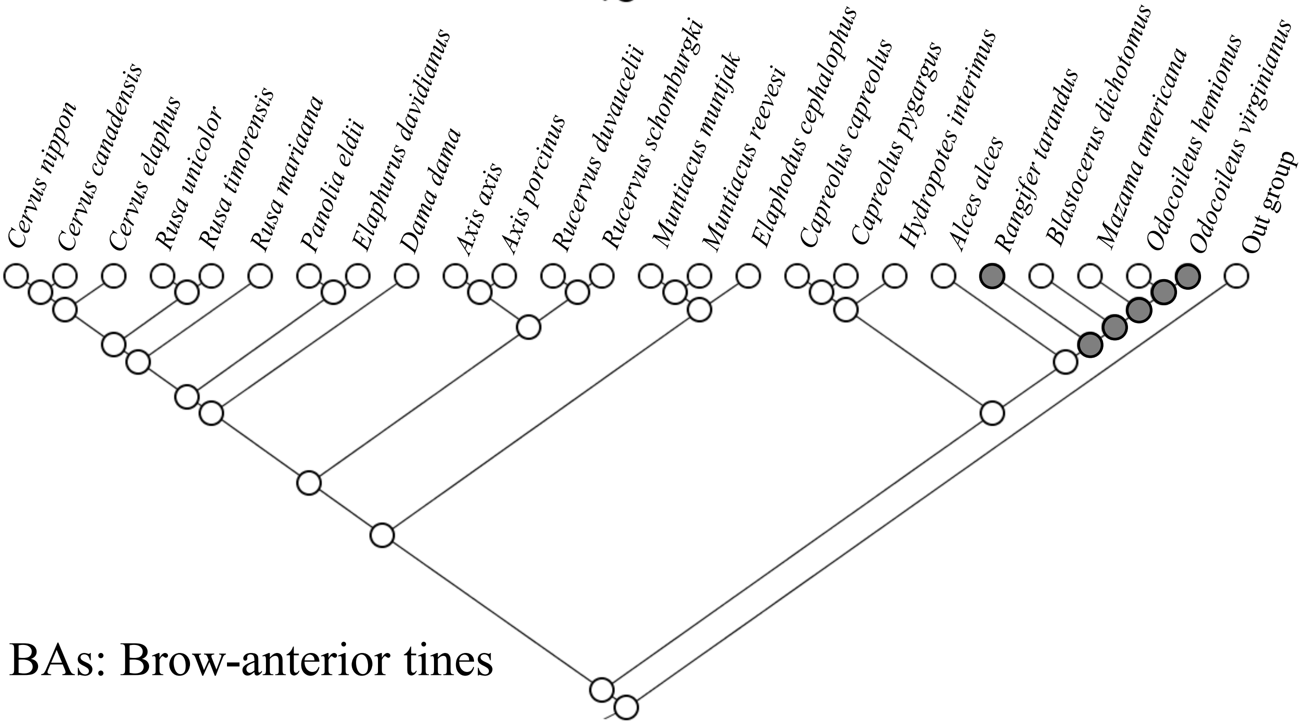

BAs: Brow-anterior tines

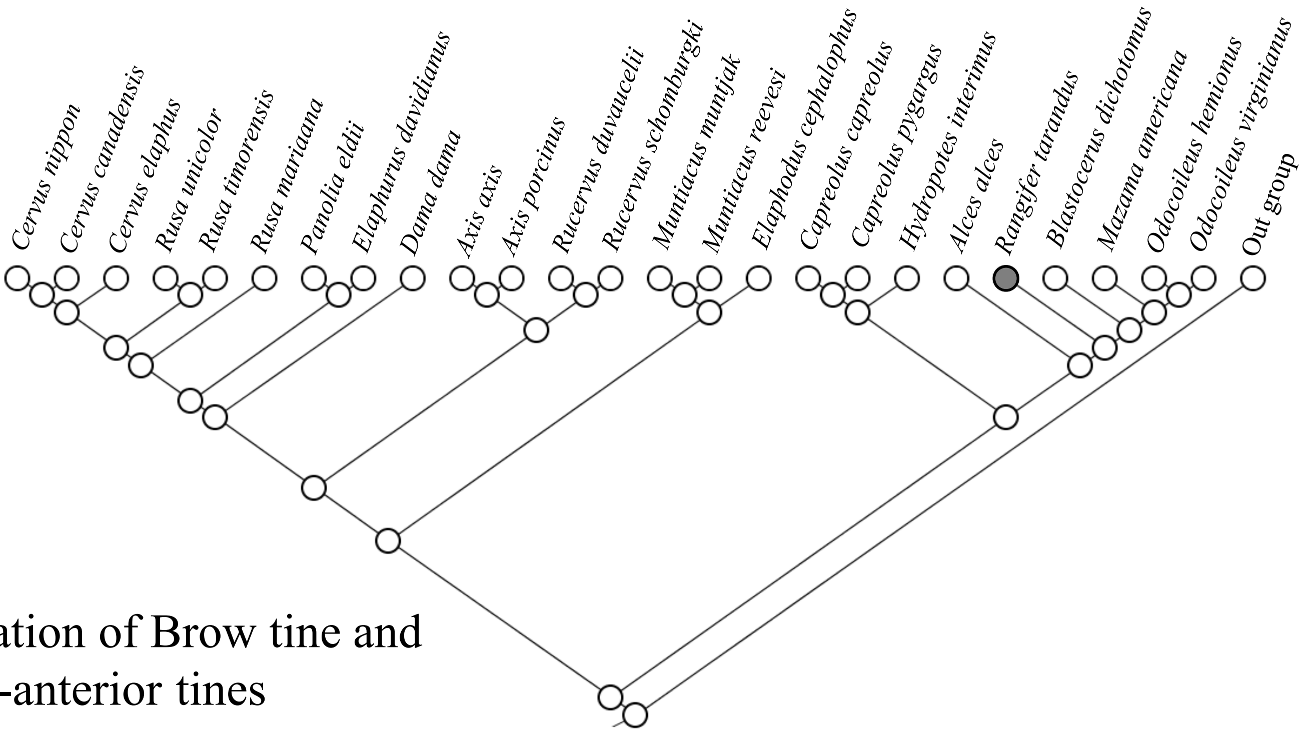

Palmation of Brow tine and
Brow-anterior tines

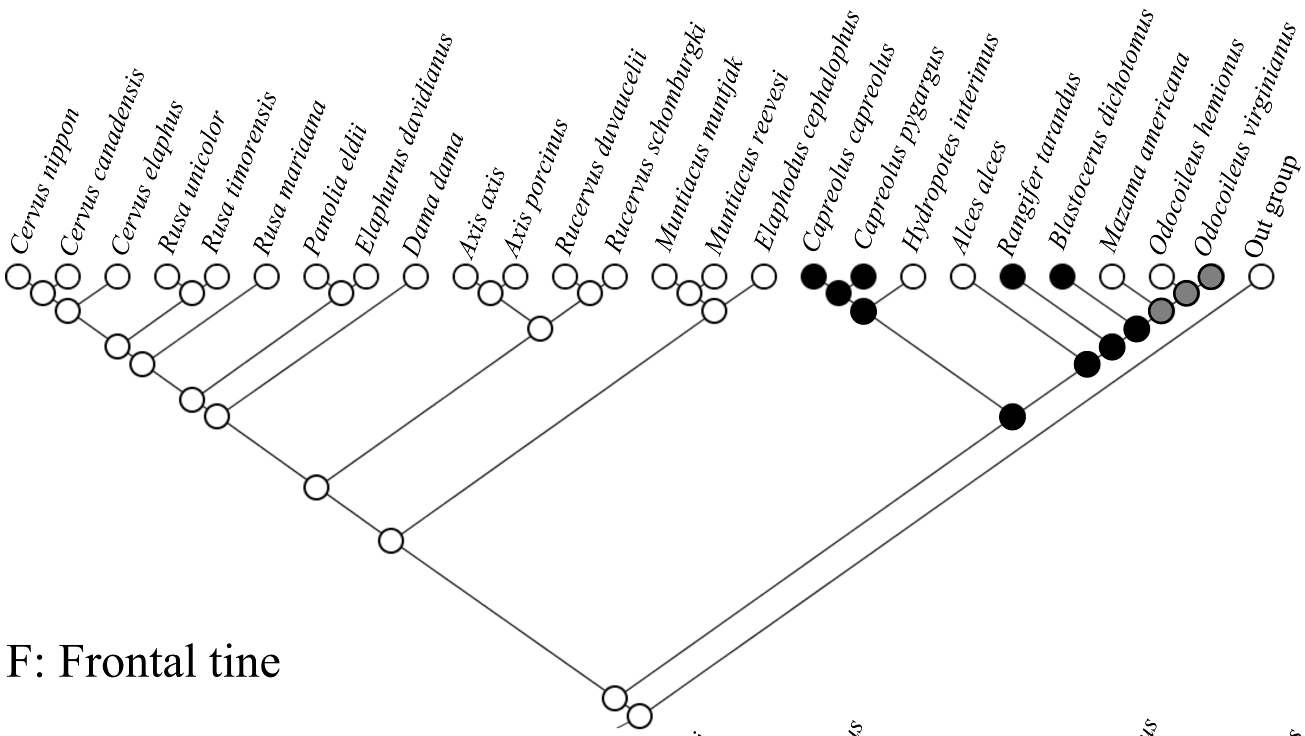

F: Frontal tine

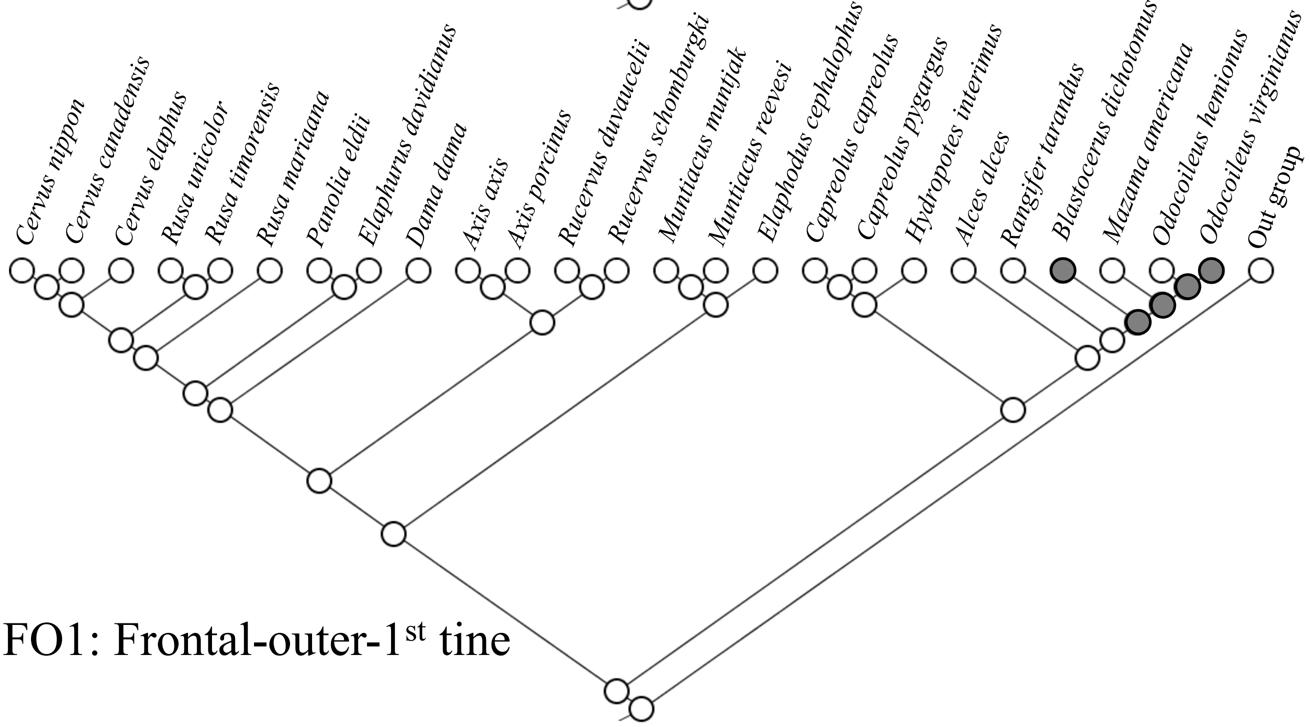

FO1: Frontal-outer-1st tine

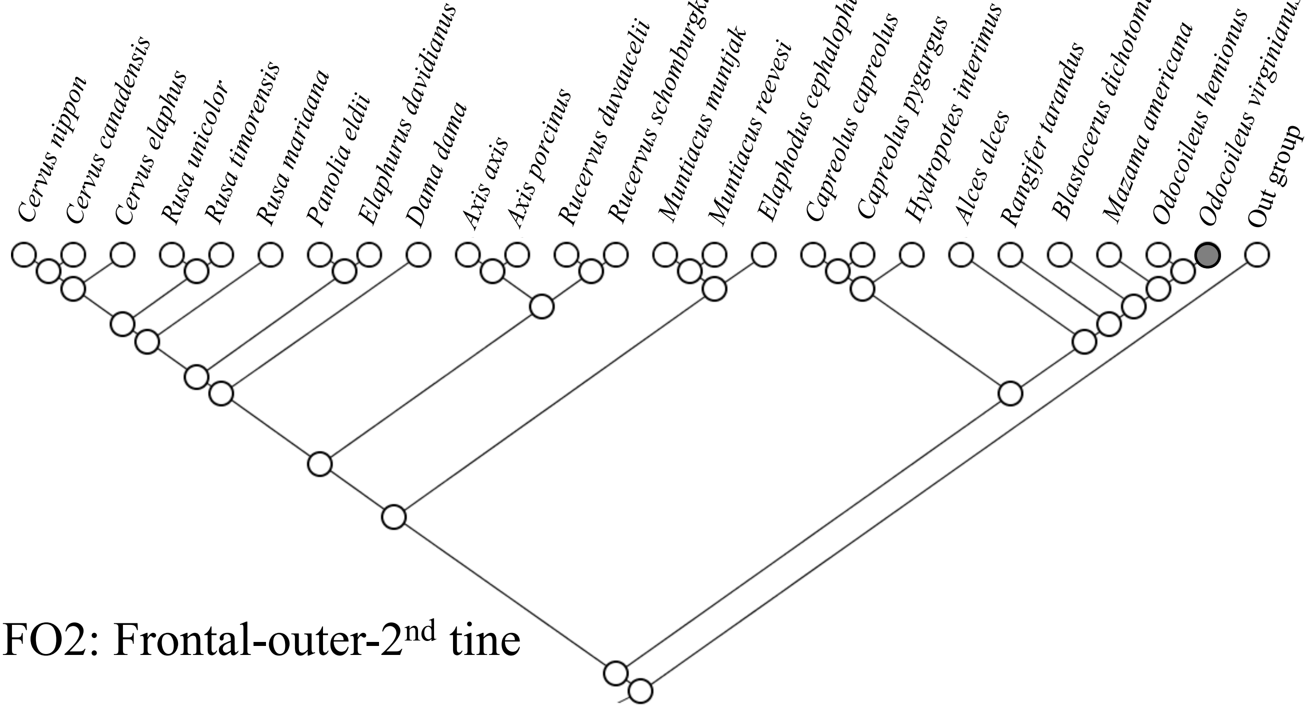

FO2: Frontal-outer-2nd tine

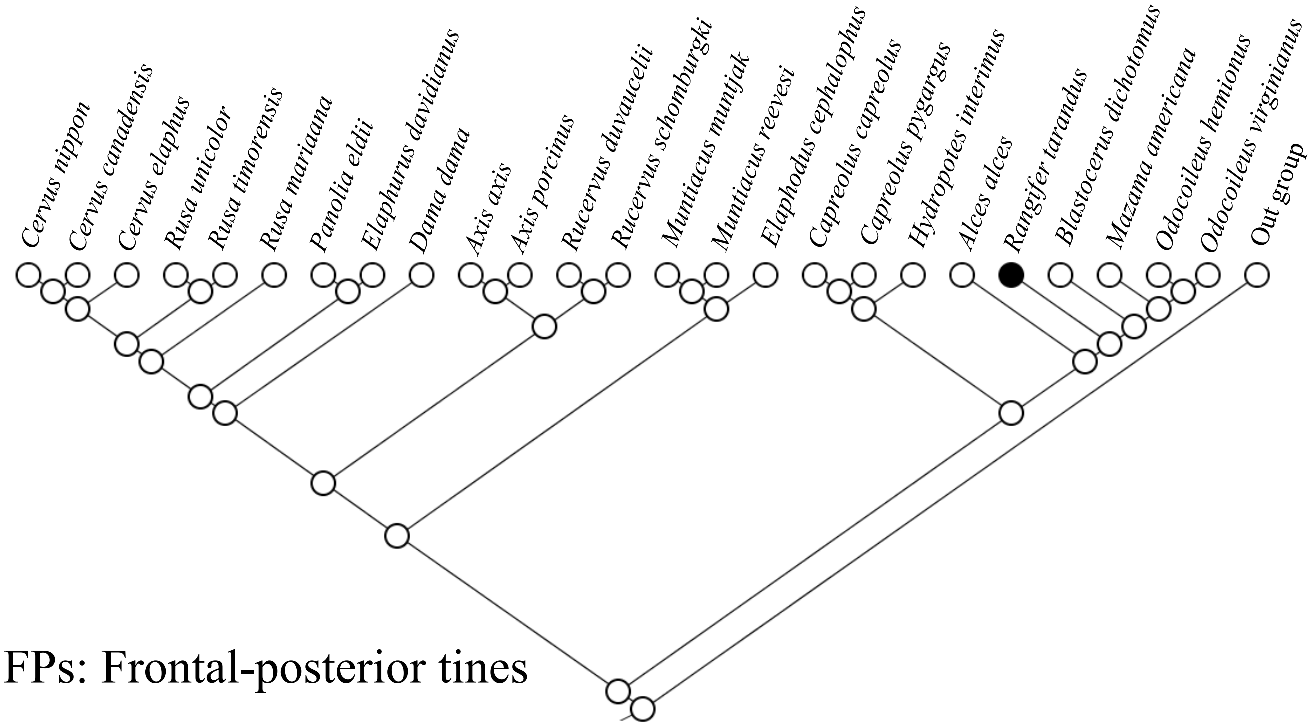

FPs: Frontal-posterior tines

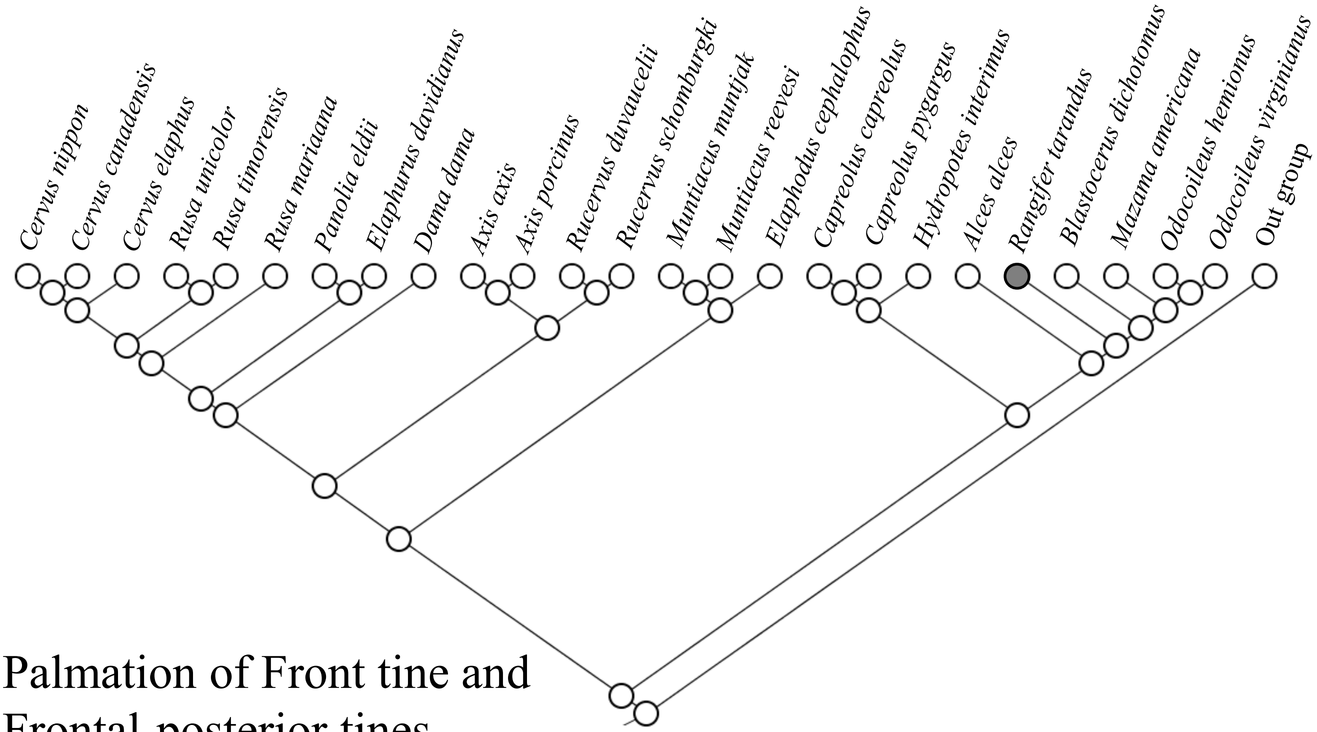

Palmation of Front tine and Frontal-posterior tines

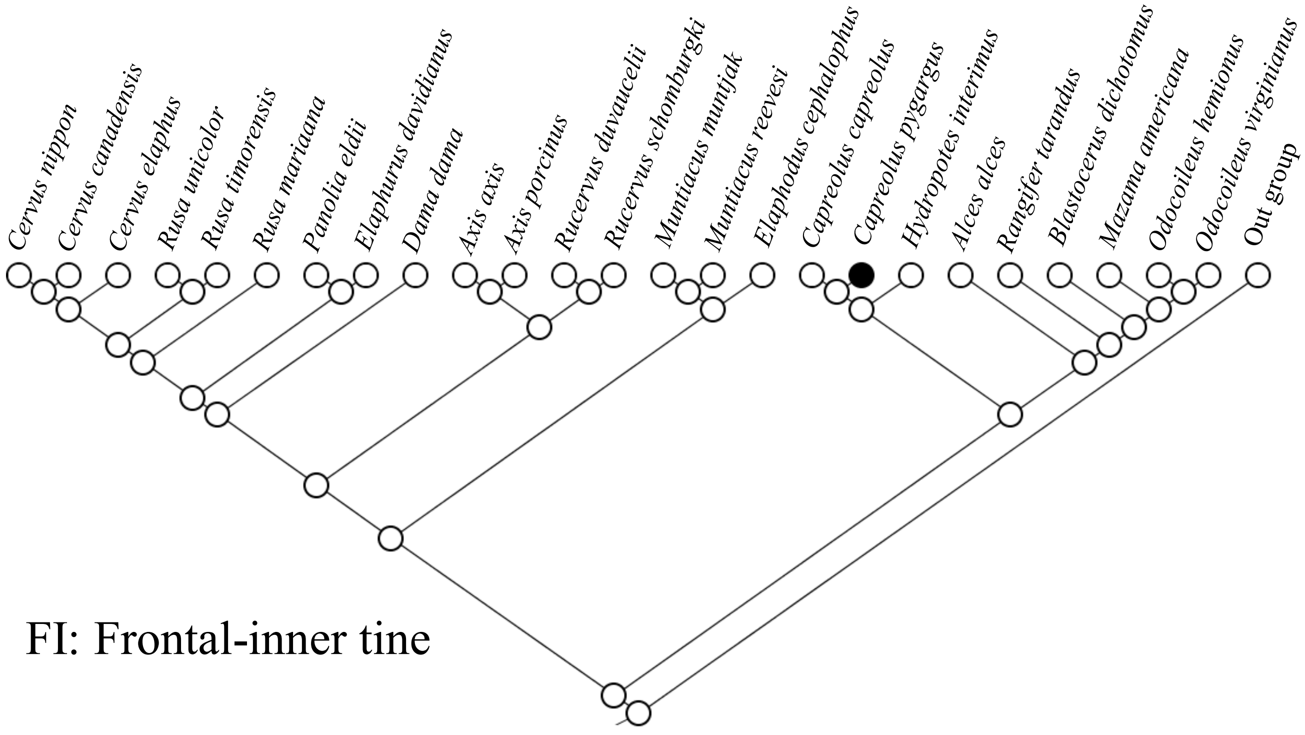

FI: Frontal-inner tine

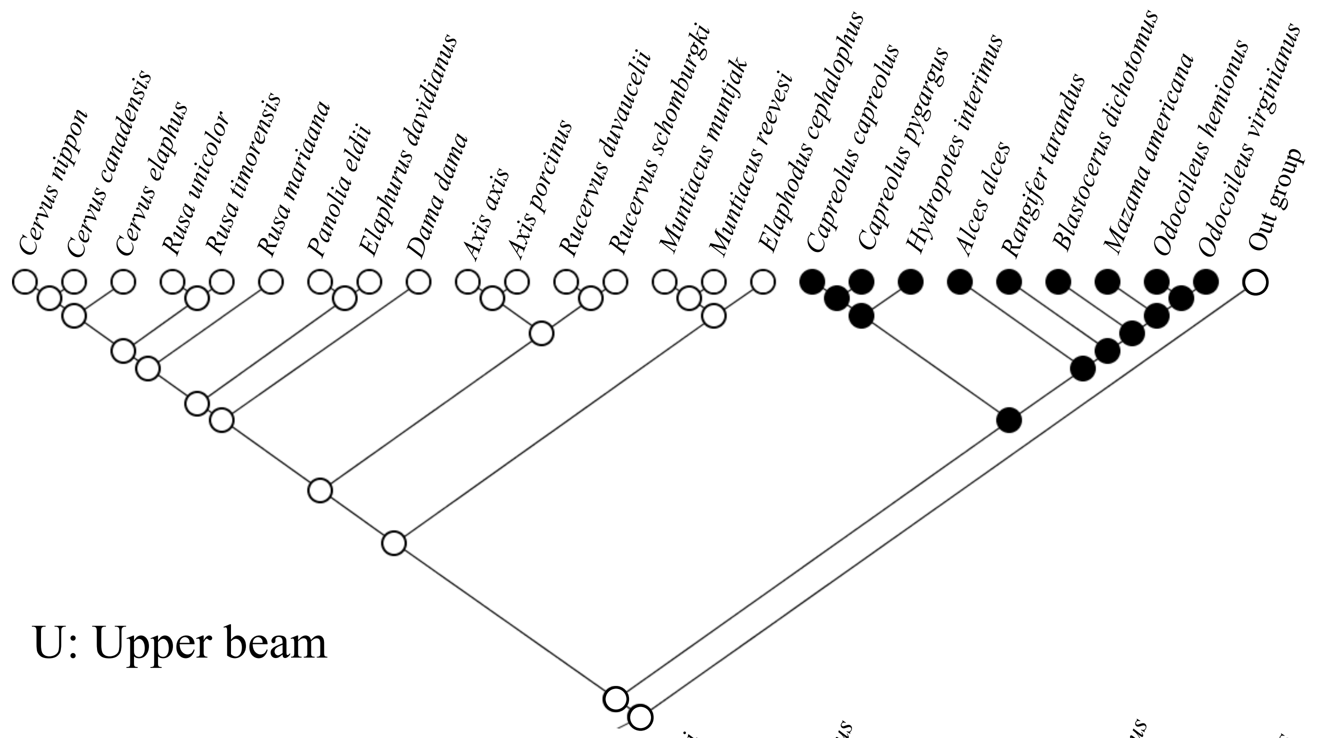
Cervus nippon
Cervus canadensis
Cervus elaphus
Rusa unicolor
Rusa timorensis
Rusa mariaana
Panolia eldii
Elaphurus davidianus
Dama dama
Axis axis
Axis porcinus
Rucervus duvaucelii
Rucervus schomburgki
Muntiacus muntjak
Muntiacus reevesi
Elaphodus cephalophus
Capreolus capreolus
Capreolus pygargus
Hydropotes interimus
Alces alces
Rangifer tarandus
Blastocerus dichotomus
Mazama americana
Odocoileus hemionus
Odocoileus virginianus
Out group
U: Upper beam

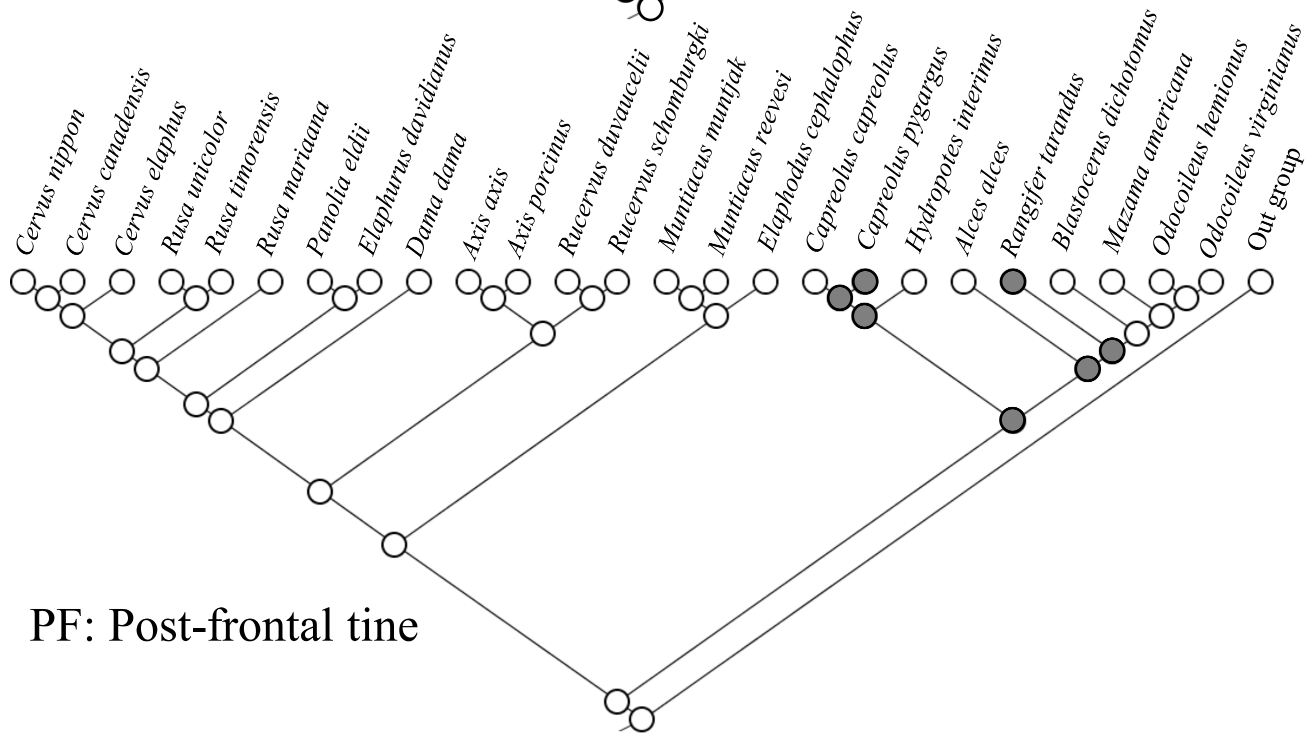
Cervus nippon
Cervus canadensis
Cervus elaphus
Rusa unicolor
Rusa timorensis
Rusa mariaana
Panolia eldii
Elaphurus davidianus
Dama dama
Axis axis
Axis porcinus
Rucervus duvaucelii
Rucervus schomburgki
Muntiacus muntjak
Muntiacus reevesi
Elaphodus cephalophus
Capreolus capreolus
Capreolus pygargus
Hydropotes interimus
Alces alces
Rangifer tarandus
Blastocerus dichotomus
Mazama americana
Odocoileus hemionus
Odocoileus virginianus
Out group
PF: Post-frontal tine

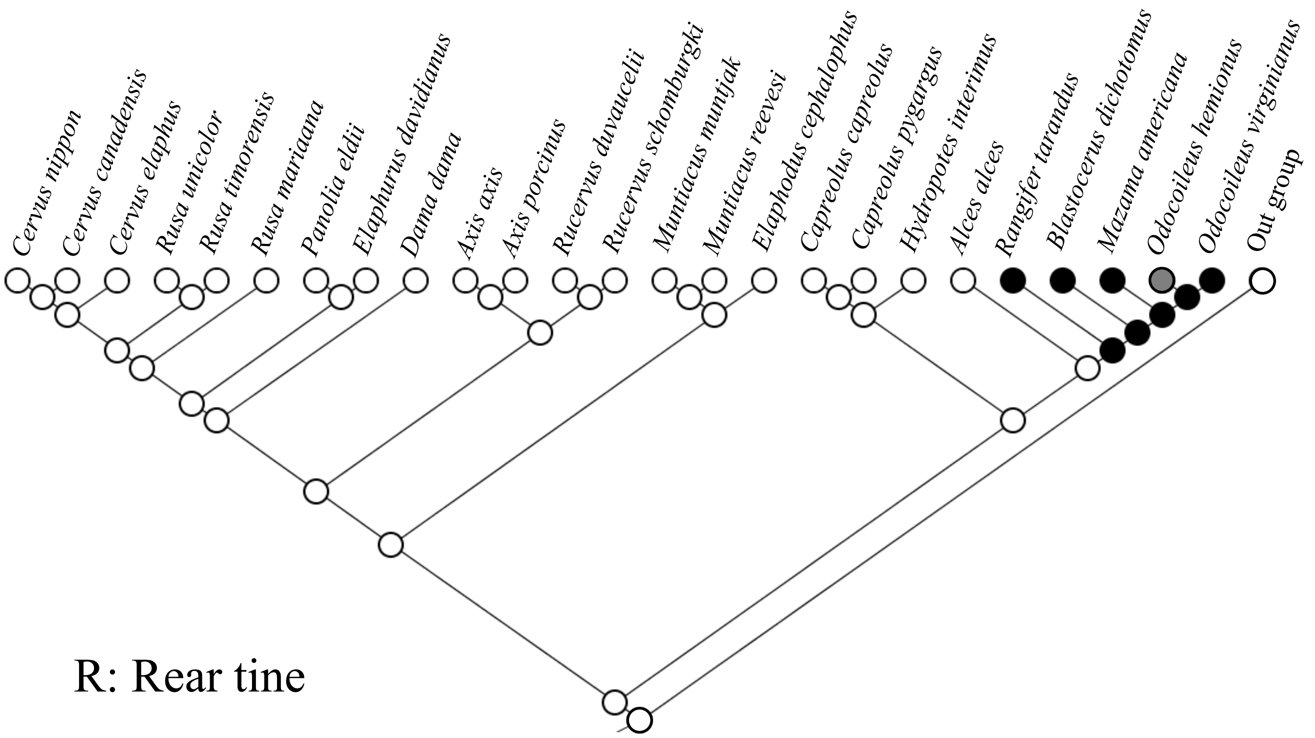
Cervus nippon
Cervus canadensis
Cervus elaphus
Rusa unicolor
Rusa timorensis
Rusa mariaana
Panolia eldii
Elaphurus davidianus
Dama dama
Axis axis
Axis porcinus
Rucervus duvaucelii
Rucervus schomburgki
Muntiacus muntjak
Muntiacus reevesi
Elaphodus cephalophus
Capreolus capreolus
Capreolus pygargus
Hydropotes interimus
Alces alces
Rangifer tarandus
Blastocerus dichotomus
Mazama americana
Odocoileus hemionus
Odocoileus virginianus
Out group
R: Rear tine

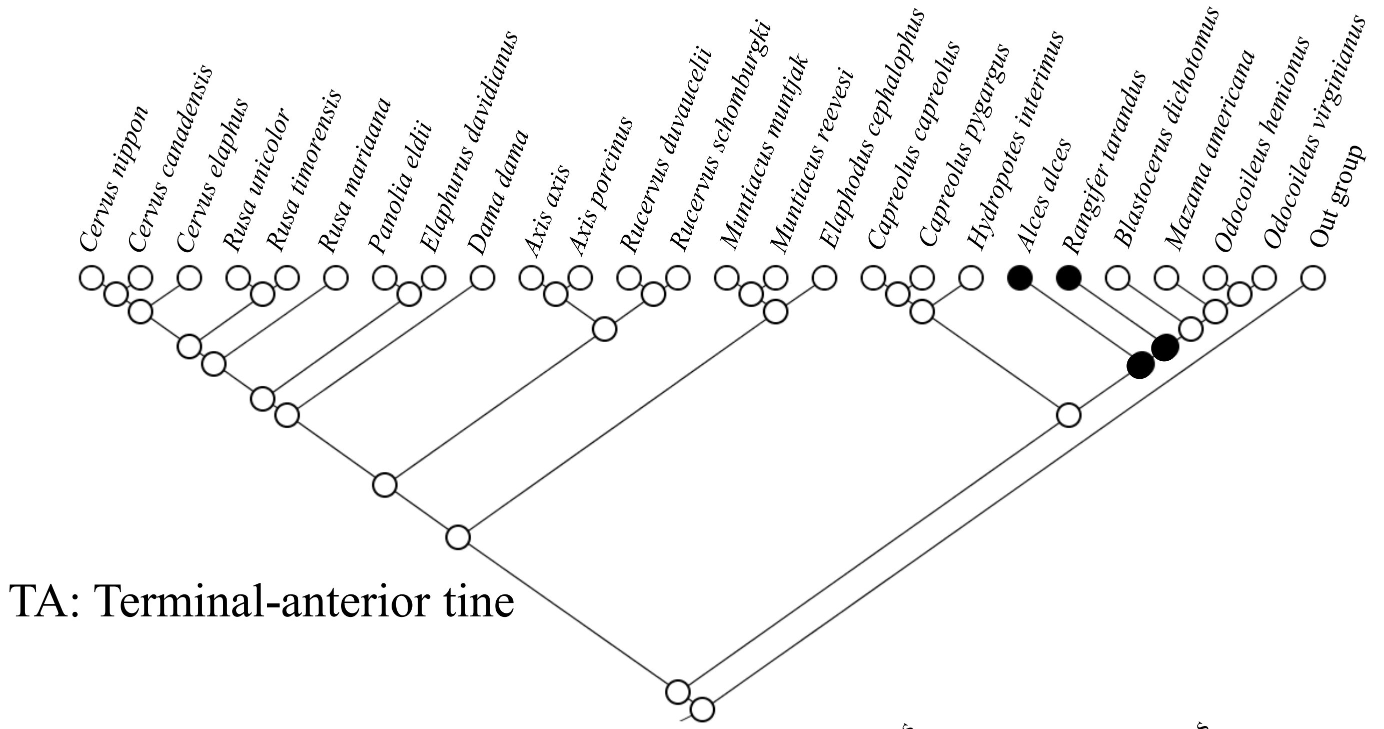
Cervus nippon
Cervus canadensis
Cervus elaphus
Rusa unicolor
Rusa timorensis
Rusa mariaana
Panolia eldii
Elaphurus davidianus
Dama dama
Axis axis
Axis porcinus
Rucervus duvaucelii
Rucervus schomburgki
Muntiacus muntjak
Muntiacus reevesi
Elaphodus cephalophus
Capreolus capreolus
Capreolus pygargus
Hydropotes interimus
Alces alces
Rangifer tarandus
Blastocerus dichotomus
Mazama americana
Odocoileus hemionus
Odocoileus virginianus
Out group
TA: Terminal-anterior tine

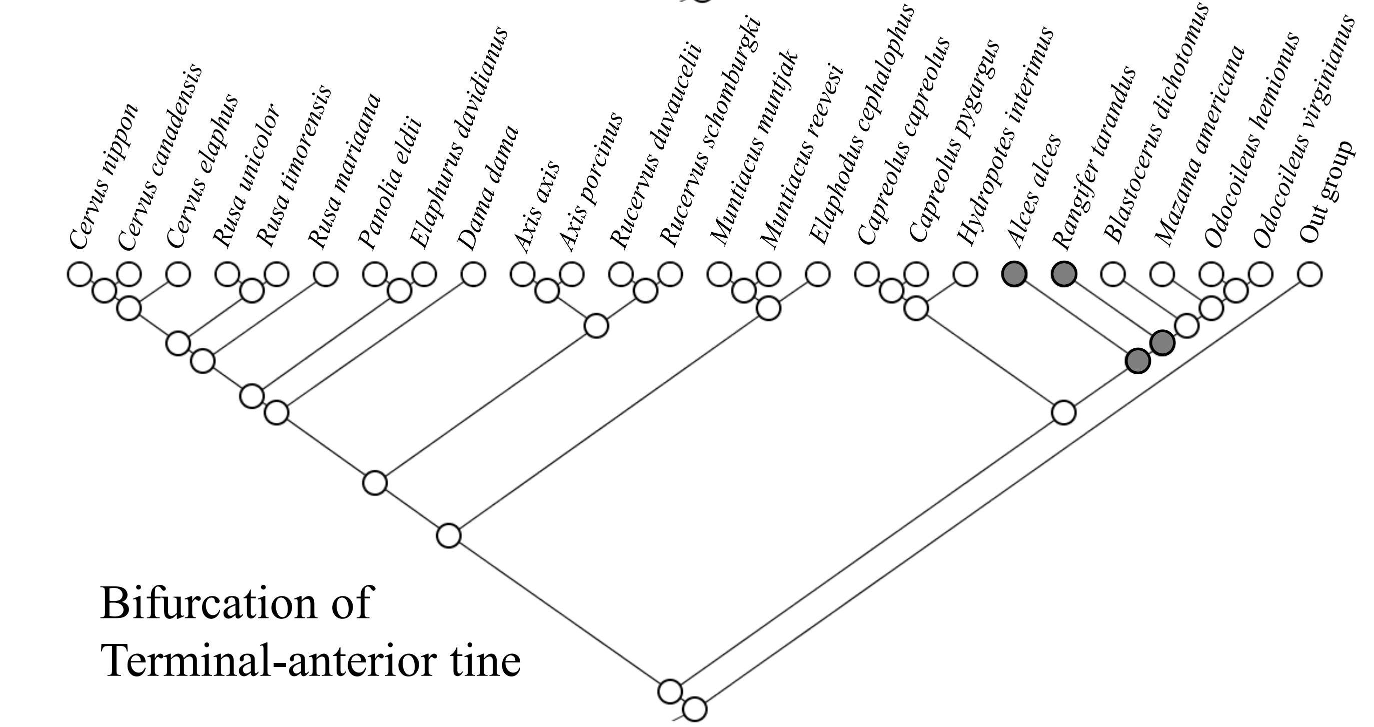
Cervus nippon
Cervus canadensis
Cervus elaphus
Rusa unicolor
Rusa timorensis
Rusa mariaana
Panolia eldii
Elaphurus davidianus
Dama dama
Axis axis
Axis porcinus
Rucervus duvaucelii
Rucervus schomburgki
Muntiacus muntjak
Muntiacus reevesi
Elaphodus cephalophus
Capreolus capreolus
Capreolus pygargus
Hydropotes interimus
Alces alces
Rangifer tarandus
Blastocerus dichotomus
Mazama americana
Odocoileus hemionus
Odocoileus virginianus
Out group
Bifurcation of
Terminal-anterior tine

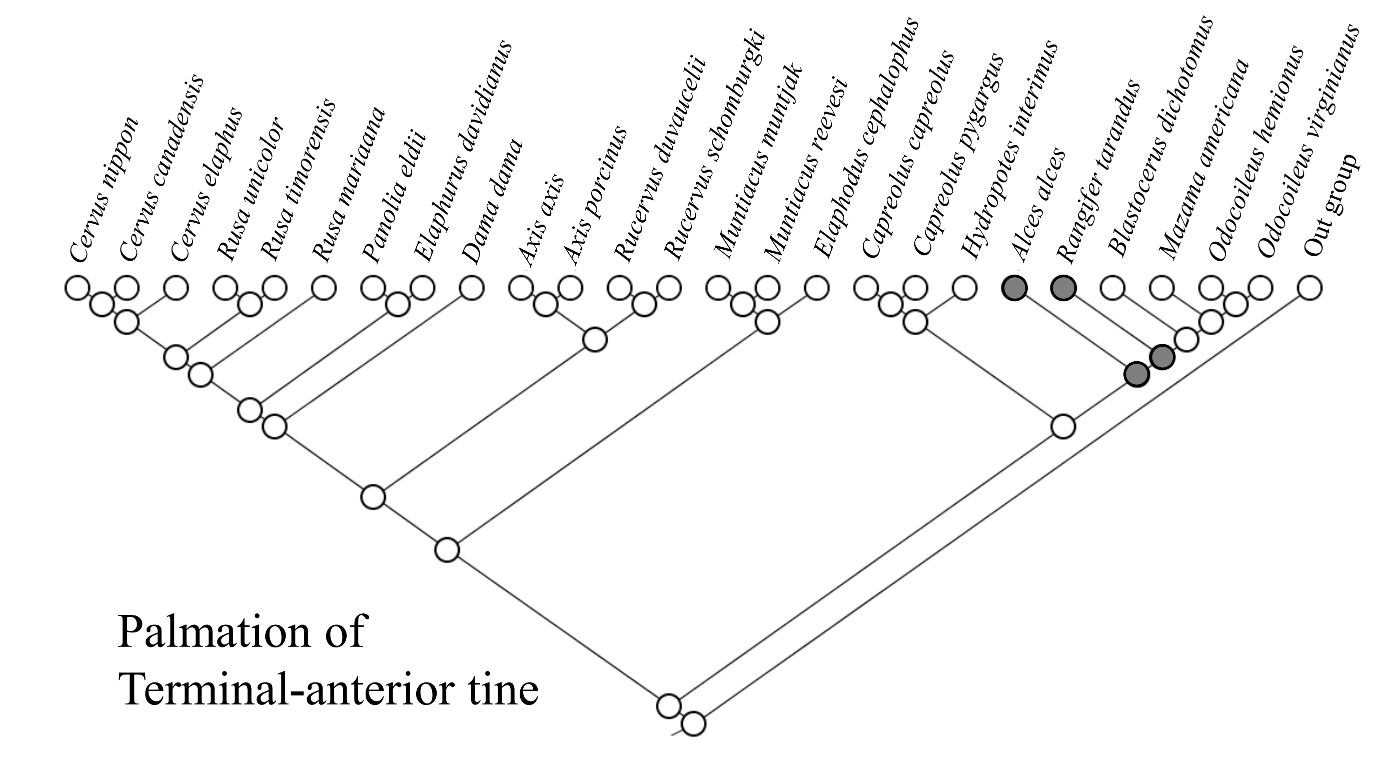
Cervus nippon
Cervus canadensis
Cervus elaphus
Rusa unicolor
Rusa timorensis
Rusa mariaana
Panolia eldii
Elaphurus davidianus
Dama dama
Axis axis
Axis porcinus
Rucervus duvaucelii
Rucervus schomburgki
Muntiacus muntjak
Muntiacus reevesi
Elaphodus cephalophus
Capreolus capreolus
Capreolus pygargus
Hydropotes interimus
Alces alces
Rangifer tarandus
Blastocerus dichotomus
Mazama americana
Odocoileus hemionus
Odocoileus virginianus
Out group
Palmation of
Terminal-anterior tine

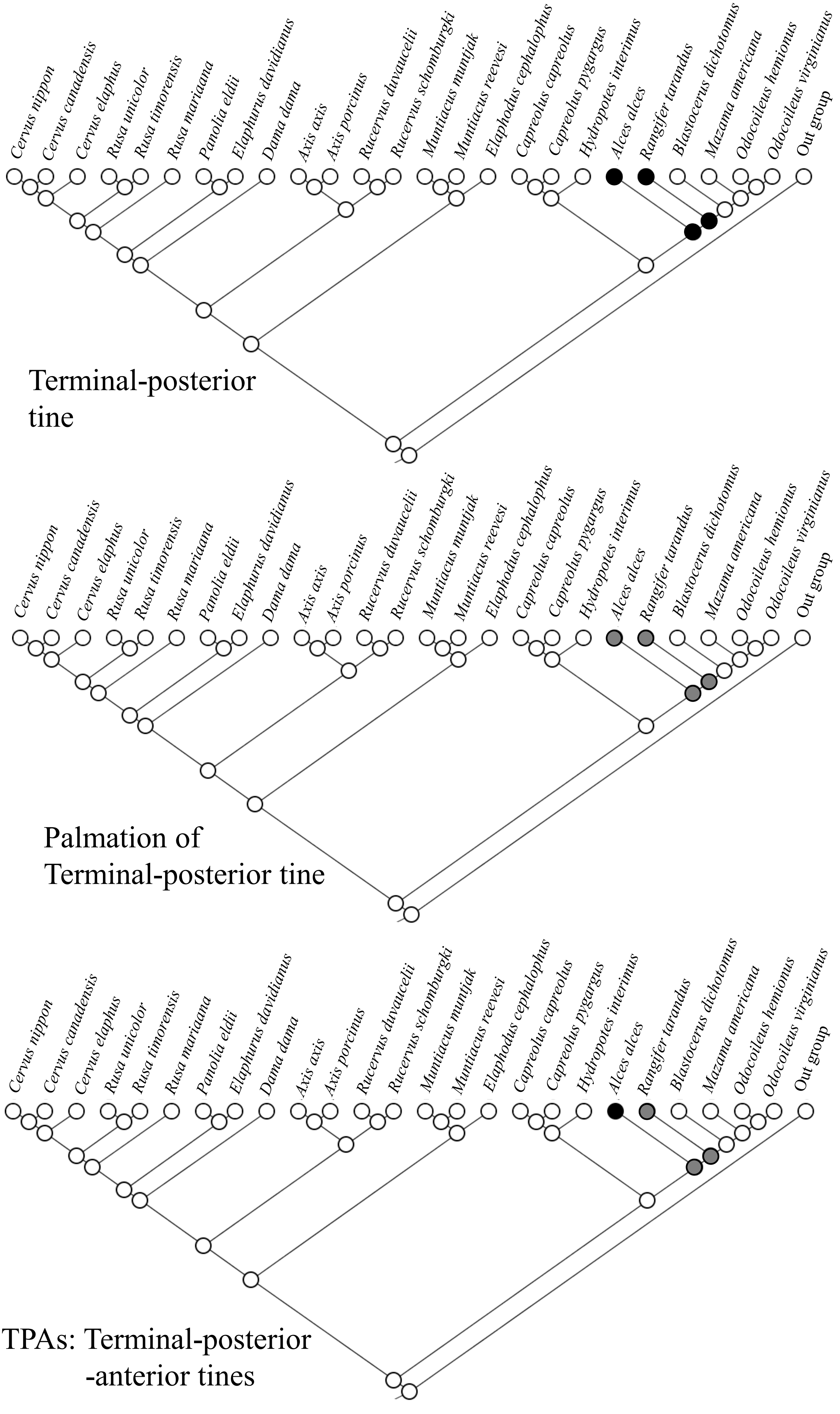
Cervus nippon
Cervus canadensis
Cervus elaphus
Rusa unicolor
Rusa timorensis
Rusa mariaana
Panolia eldii
Elaphurus davidianus
Dama dama
Axis axis
Axis porcinus
Rucervus duvaucelii
Rucervus schomburgki
Muntiacus muntjak
Muntiacus reevesi
Elaphodus cephalophus
Capreolus capreolus
Capreolus pygargus
Hydropotes interimus
Alces alces
Rangifer tarandus
Blastocerus dichotomus
Mazama americana
Odocoileus hemionus
Odocoileus virginianus
Out group
Terminal-posterior tine
Cervus nippon
Cervus canadensis
Cervus elaphus
Rusa unicolor
Rusa timorensis
Rusa mariaana
Panolia eldii
Elaphurus davidianus
Dama dama
Axis axis
Axis porcinus
Rucervus duvaucelii
Rucervus schomburgki
Muntiacus muntjak
Muntiacus reevesi
Elaphodus cephalophus
Capreolus capreolus
Capreolus pygargus
Hydropotes interimus
Alces alces
Rangifer tarandus
Blastocerus dichotomus
Mazama americana
Odocoileus hemionus
Odocoileus virginianus
Out group
Palmation of Terminal-posterior tine
Cervus nippon
Cervus canadensis
Cervus elaphus
Rusa unicolor
Rusa timorensis
Rusa mariaana
Panolia eldii
Elaphurus davidianus
Dama dama
Axis axis
Axis porcinus
Rucervus duvaucelii
Rucervus schomburgki
Muntiacus muntjak
Muntiacus reevesi
Elaphodus cephalophus
Capreolus capreolus
Capreolus pygargus
Hydropotes interimus
Alces alces
Rangifer tarandus
Blastocerus dichotomus
Mazama americana
Odocoileus hemionus
Odocoileus virginianus
Out group
TPAs: Terminal-posterior -anterior tines

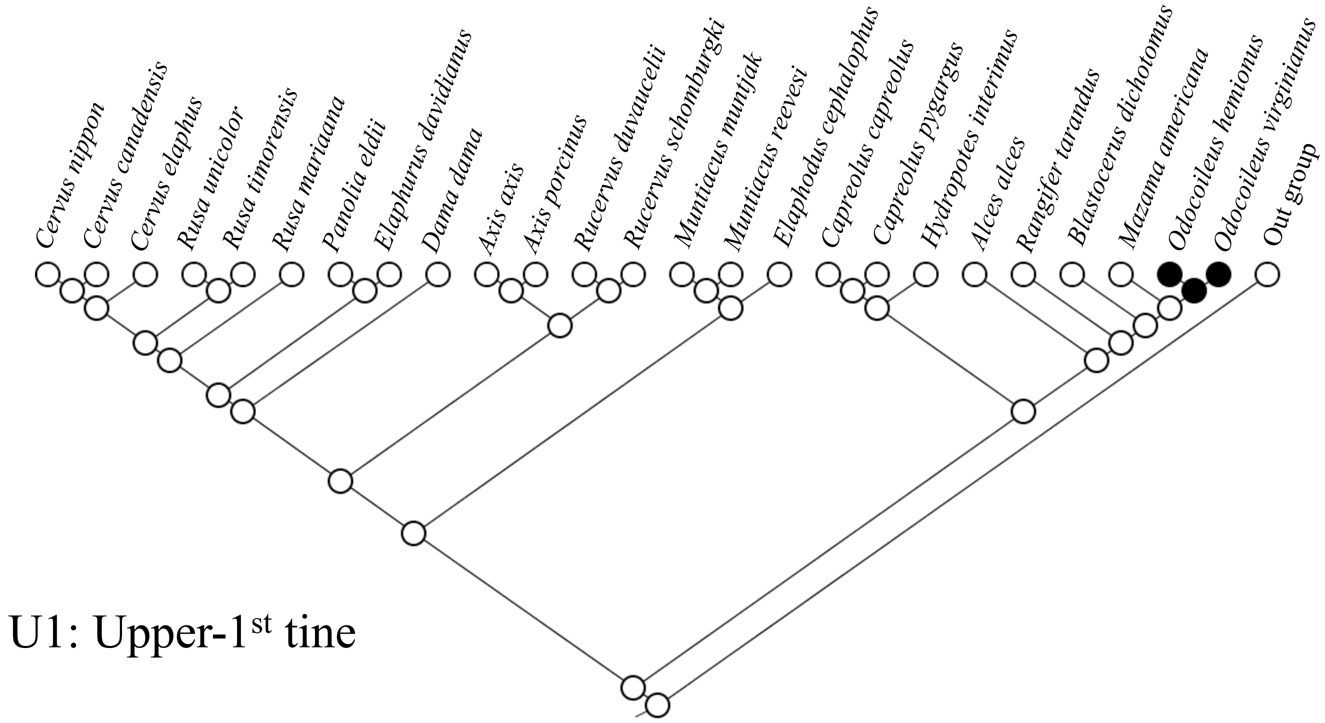
Cervus nippon
Cervus canadensis
Cervus elaphus
Rusa unicolor
Rusa timorensis
Rusa mariaana
Panolia eldii
Elaphurus davidianus
Dama dama
Axis axis
Axis porcinus
Rucervus duvaucelii
Rucervus schomburgki
Muntiacus muntjak
Muntiacus reevesi
Elaphodus cephalophus
Capreolus capreolus
Capreolus pygargus
Hydropotes interimus
Alces alces
Rangifer tarandus
Blastocerus dichotomus
Mazama americana
Odocoileus hemionus
Odocoileus virginianus
Out group
U1: Upper-1st tine

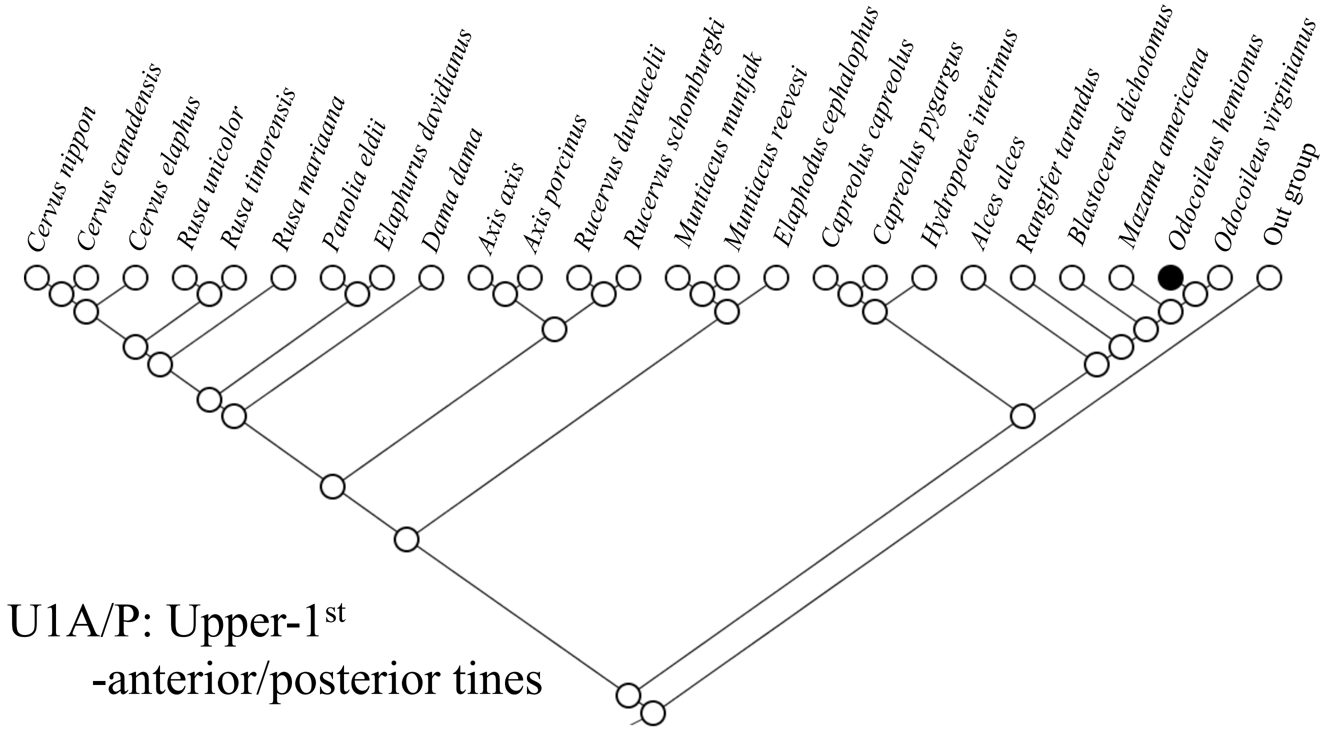
Cervus nippon
Cervus canadensis
Cervus elaphus
Rusa unicolor
Rusa timorensis
Rusa mariaana
Panolia eldii
Elaphurus davidianus
Dama dama
Axis axis
Axis porcinus
Rucervus duvaucelii
Rucervus schomburgki
Muntiacus muntjak
Muntiacus reevesi
Elaphodus cephalophus
Capreolus capreolus
Capreolus pygargus
Hydropotes interimus
Alces alces
Rangifer tarandus
Blastocerus dichotomus
Mazama americana
Odocoileus hemionus
Odocoileus virginianus
Out group
U1A/P: Upper-1st
-anterior/posterior tines

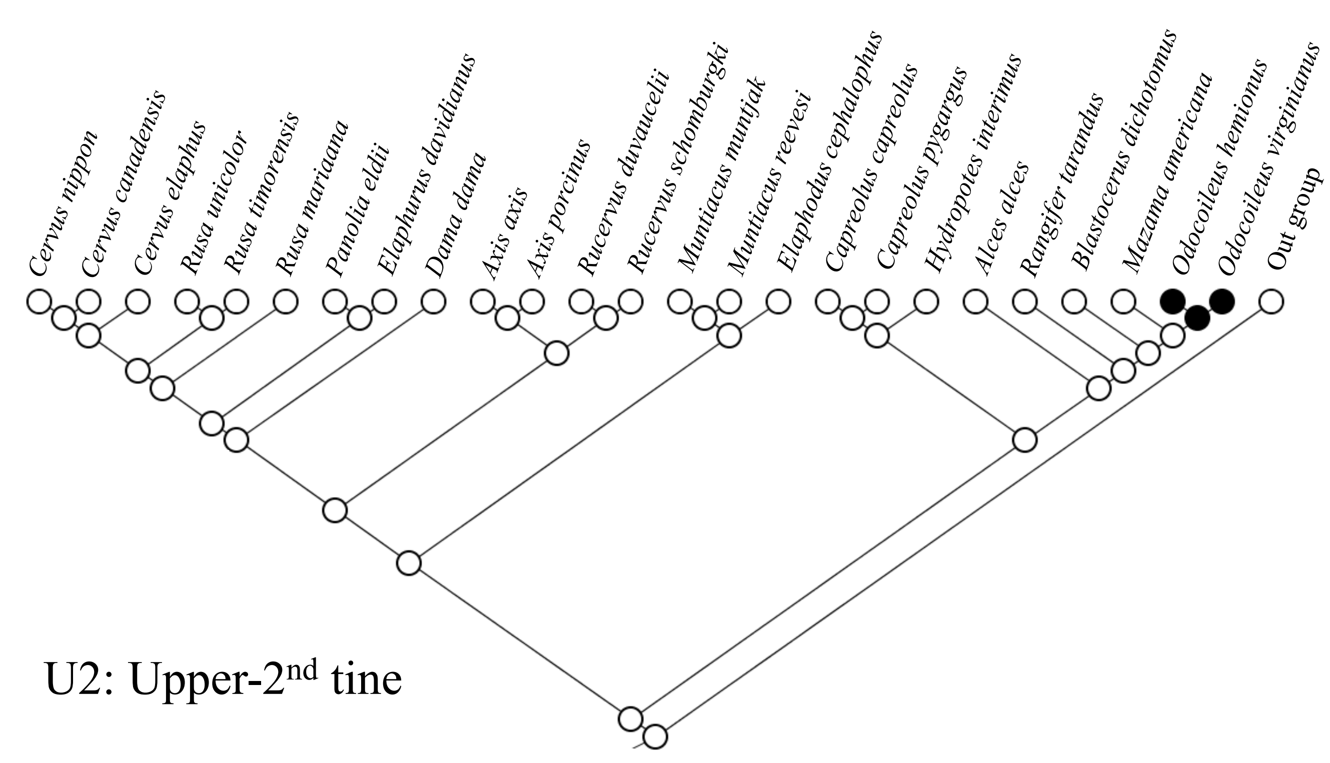
Cervus nippon
Cervus canadensis
Cervus elaphus
Rusa unicolor
Rusa timorensis
Rusa mariaana
Panolia eldii
Elaphurus davidianus
Dama dama
Axis axis
Axis porcinus
Rucervus duvaucelii
Rucervus schomburgki
Muntiacus muntjak
Muntiacus reevesi
Elaphodus cephalophus
Capreolus capreolus
Capreolus pygargus
Hydropotes interimus
Alces alces
Rangifer tarandus
Blastocerus dichotomus
Mazama americana
Odocoileus hemionus
Odocoileus virginianus
Out group
U2: Upper-2nd tine

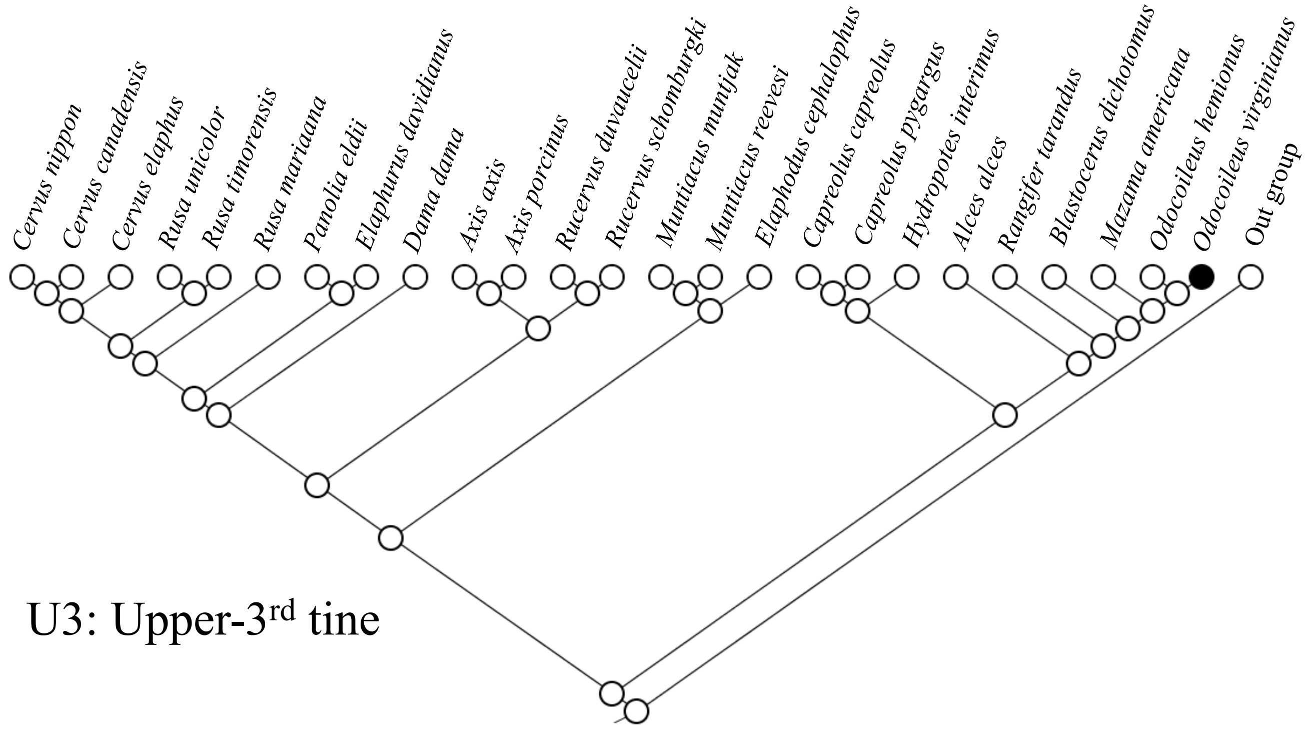
Cervus nippon
Cervus canadensis
Cervus elaphus
Rusa unicolor
Rusa timorensis
Rusa mariaana
Panolia eldii
Elaphurus davidianus
Dama dama
Axis axis
Axis porcinus
Rucervus duvaucelii
Rucervus schomburgki
Muntiacus muntjak
Muntiacus reevesi
Elaphodus cephalophus
Capreolus capreolus
Capreolus pygargus
Hydropotes interimus
Alces alces
Rangifer tarandus
Blastocerus dichotomus
Mazama americana
Odocoileus hemionus
Odocoileus virginianus
Out group
U3: Upper-3rd tine
