# Supplementary material for: A new viewpoint on antlers reveals the evolutionary history of deer (Cervidae, Mammalia)
Source: Sci Rep. 2020 Jun 2;10:8910. doi: 10.1038/s41598-020-64555-7 (PMC7265483; doi:10.1038/s41598-020-64555-7)
Supplement: Supplementary file 7 — Supplementary information 7 - Percentages of the elements by species. [file 41598_2020_64555_MOESM7_ESM.pdf]

Yuusuke Samejima & Hiroshige Matsuoka

Supplementary Information 8

List of the specimens observed

Abbreviations of the names of museums and institutes in which observed specimens are reposited are as follows;

CBM: Natural History Museum and Institute, Chiba
HUBG: Botanic Garden, Hokkaido University
KPM: Kanagawa Prefecture Museum of Natural History
KUGM: Department of Geology and Mineralogy, Facility of Science, Kyoto University
KUZ: The Kyoto University Museum
LBM: Lake Biwa Museum
NSMT: National Museum of Nature and Science
OMNH: Osaka Museum of Natural History
SMZ: Sapporo Maruyama Zoo
TG: Taga Town Museum
TUM: The Tohoku University Museum

Specimen numbers

Alces alces: **KUGM-**RM029, RM049, RM152, RM151, RM150, RM148, RM146, RM147; **NSMT-**M43310, M56471, M01172, M01095, M32304, M32040, M32374, M39880, M39881, M34879, M56391, M56393, M32305, M01052, M32305

Axis axis: **KUGM-**RM014, RM046, RM047; **NSMT-**M01145, M32346, M32342, M32131, M32345, M32132, M00131, M42835

Axis porcinus: **NSMT-**M19849

Blastocerus dichotomus: **KUGM**-RM134

Capreolus capreolus: **KUGM-**RM027, RM028, RM102, RM101, RM097, RM100; **NSMT-**M43323, M911, M32377, M32247, M56410, M32183

Capreolus pygargus: **KUGM**-RM131, RM080, RM081, RM082, RM083, RM084, RM085, RM086, RM087, RM130; **NSMT-**M23806, M01169 ×3, M1170, M56419

Cervus canadensis: **KUGM-**RM109; **NSMT-**M43324, M03810, M07538, M01164, M01167, M01165, M42710, M32034, M01094, M39878, M39877, M39876, M32353, M56402, M56403; **TUM**-No number

Cervus elaphus: **KUGM**-RM019, RM048, RM132; **LBM**-1900000213; **NSMT-**M43318, M01162, M43325, M32195, M56408, M56401, M32006, No number, M01163, M32194; **TUM**-No number ×2

Cervus nippon (mature individuals): **CBM**-ZZ0001202, ZZ0001182, No number (Displayed), ZZ000828, ZZ000618, ZZ000238, ZZ0001116, ZZ0005971; **HUBG**-10278, 13037, 10279; **KUGM**-RM135, RM124, RM127, RM125, RM126, RM001, RM005, RM003, RM007, RM091, RM004, RM017, RM104, RM018, RM016, RM145, RM213; **NSMT-**M43312, M43307, M43313, M21192, M01158, M01156, M43306, M43317, M43321, M43322, M43316, M43314, M47189, M14306, M13693, No number ×2, M42803, M37524, M37523, M50096, M49973; **SMZ**-Displayed 4th and 5th from the left on 1F in Wolf and Deer house, Displayed on 2F in Wolf and Deer house ×4; **TZP-**Displayed in *C.nippon* area ×3

Cervus nippon (juvenile individuals): **CBM**-ZZ0002037, ZZ0001967, ZZ0001776, ZZ0001570, ZZ0001775, ZZ0001679, ZZ0001516, ZZ0001553, ZZ0001313, ZZ0001774; **KUGM**-RM008, RM009, RM006; **NSMT-**M23151, No number; **SMZ**-Displayed 1st, 2nd and 3rd from the left on 1F in Wolf and Deer house

Dama dama (mature individuals): **KUGM**-RM010, RM011, RM045, RM133, RM143, RM144, RM107; **LBM-**1900000787; **NSMT-**M32070, M32192, M56407, No number (lamp) ×2

Dama dama (juvenile individuals): **KUGM**-RM056, RM200, RM201, RM202, RM203, RM204, RM205

Elaphurus davidianus: **KUGM-**RM022, RM041, RM042, RM043, RM044, RM153, RM154, RM155, **NSMT-**M00425, M00464, M01168, M38630

Elaphodus cephalophus: **KPM-**No number

Hydropotes intermis: **KUGM**-RM031, RM215

Mazama americana: **KUGM**-RM035

Muntiacus muntijak: **KUGM-**RM023; **NSMT-**M01144

Muntiacus reevesi: **CBM**-ZZ0004650, ZZ0004982, ZZ0005697, ZZ0003058, ZZ0005820, ZZ0005700, ZZ0005689, ZZ0004663, ZZ0004638, ZZ0004636, ZZ0004679, ZZ0004669, ZZ0003059, ZZ0006103, ZZ0006096, IM9, ZZ0006097, ZZ0006130, ZZ0004648, ZZ0004606; **KUGM-**RM024, RM026; **NSMT-**M43315×2

Odocoileus hemionus: **CBM**-ZZ0006163; **KUGM-**RM054, RM053, RM055, RM032, RM076; **NSMT-**M32364, M32409, M32355, M56412, M42837, M56386

Odocoileus virginianus: **KUGM**-RM033, RM208, RM074, RM077, RM078, RM117, RM118, RM116, RM112, RM115, RM058, RM054, RM071, RM088, RM072, RM090, RM068, RM089, RM075, RM073, RM057, RM064, RM059, RM066, RM067, RM062, RM141, RM142, RM034, RM092, RM136, RM079, RM069, RM113, RM061, RM060, RM070, RM065, RM144, RM119, RM063, RM111, RM123, RM114, RM120, RM121, RM122, RM110; **NSMT-**M00282, M32322, M39872, M00189, M39866, M32362, M39836, M39839, M39838, M39837, M39844

Panolia eldii: **HUBG**-10286; **LBM**-1990000009(=1990000010), **NSMT-**M01153, M01154, M01155; **ONHM**-M2034

Rangifer tarandus: **KUGM**-RM052, RM051, RM050; **KUZ**-M00205, No number; **NSMT-**M56438, M31343, M01248, M01096, M01173, M01174, M54712, M01023, M10205, M32042, M32032, M32044, M32318, M32307, M32317, M32043, M42840, M39883, M39882, M39884, M56387, M43364

Rucervus duvaucelii: **NSMT-**M01152; **TUM**-No number

Rucervus schomburgki: **NSMT-**M43311, M01151

Rusa mariaana: **HUBG**-10291; **NSMT-**M26698, M08423

Rusa timorensis: **KUGM**-RM021; **TG-**TK-0152

***Rusa* sp.**: **KUGM**-RM210, RM211, RM212

Rusa unicolor: **HUBG**-10290, 10294, 32836, 10276; **KUGM**-RM020, RM039, RM137, RM139, RM140, RM138; **KULB**-No number; **LBM**-19900000008, 19900000003; **NSMT-**M01146, M01147, M08939, M08932, M08950, M34316, M09624, M36067, M01849, M08955, M08949, M43318, M43308, M32196, No number, M32138, M05986; **OMNH**-M0435
