# Supplementary material for: A new viewpoint on antlers reveals the evolutionary history of deer (Cervidae, Mammalia)
Source: Sci Rep. 2020 Jun 2;10:8910. doi: 10.1038/s41598-020-64555-7 (PMC7265483; doi:10.1038/s41598-020-64555-7)
Supplement: Supplementary file 8 — Supplementary information 8 - Ancestral state reconstruction of the homologous elements. [file 41598_2020_64555_MOESM8_ESM.pdf]

A new viewpoint on antlers reveals the evolutionary history of deer (Cervidae, Mammalia)

Yuusuke Samejima & Hiroshige Matsuoka

Supplementary Information 3

Various types of antlers observed in this study

Note about the diagrams

- The large circle represents the burr cross section.
- The dots are the branching direction of the tines.
- The short lines orthogonal to the circle are the positions of the forks.
- The painted areas are the zones of the tines.
- Inside the circle, the branching hierarchical order among the tines is represented.
- The central dot is the base of antler.
- Outside the circle, the positional relation with the skull is represented; thin lines are the ridges on the pedicle and the thin arrows are the position extended along the nerve impressions.
- Hatching in the circle means palmation.

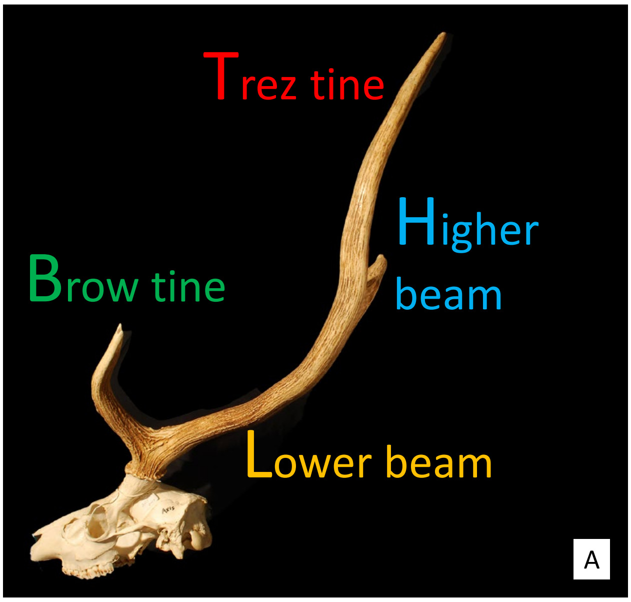

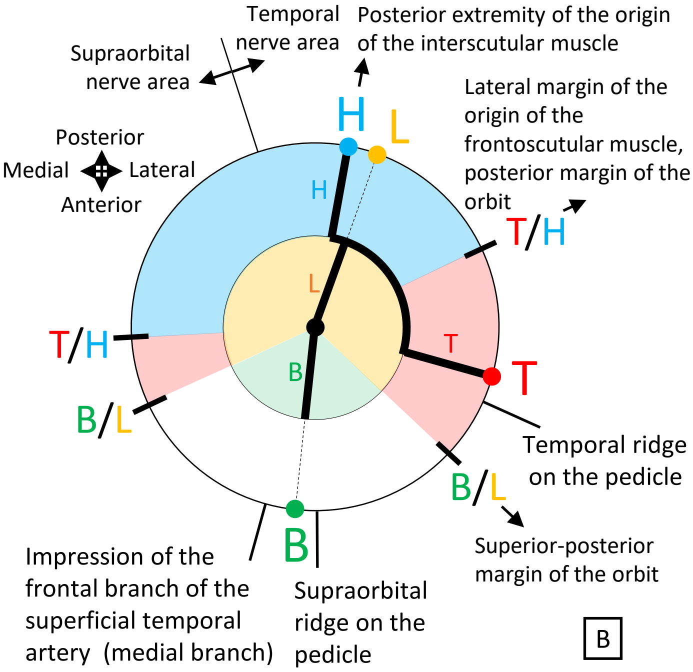

Figure 1. *Axis axis* (KUGM-RM014) A) Left antler, lateral view. B) Diagram of left antler.

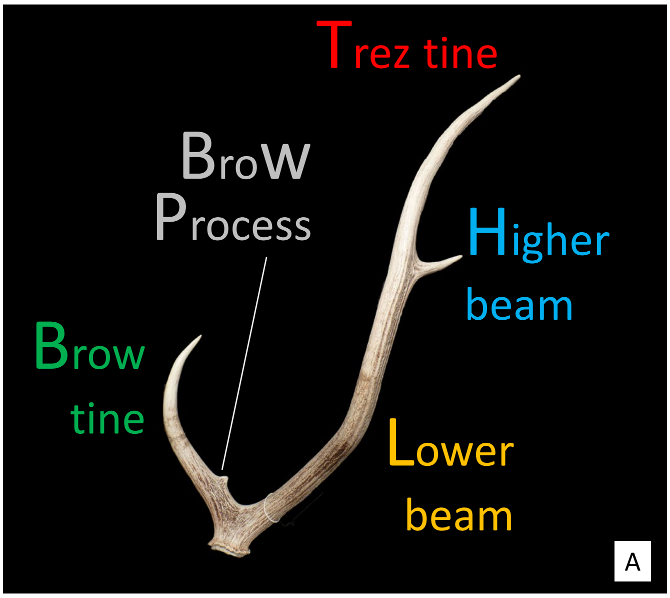

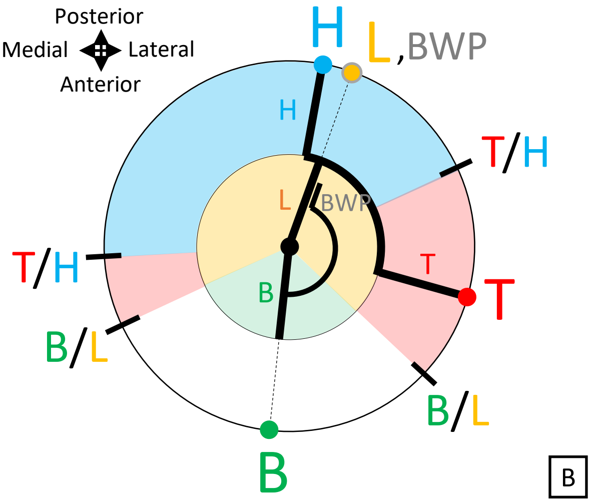

Figure 2. *Axis axis* (KUGM-RM046) A) Left antler, lateral view. B) Diagram of the left antler.

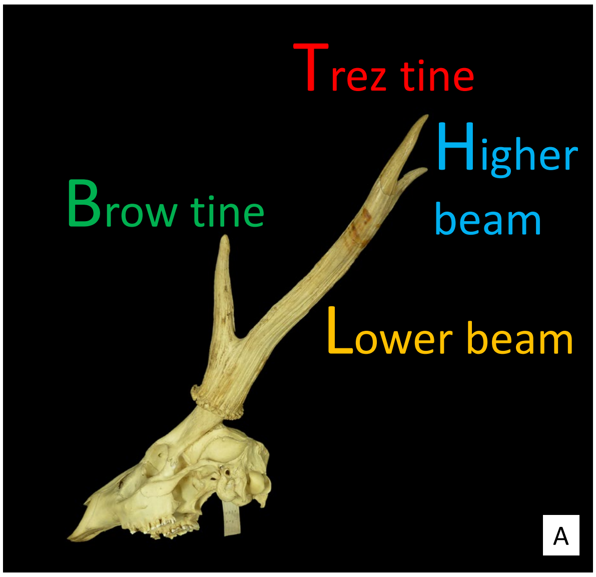

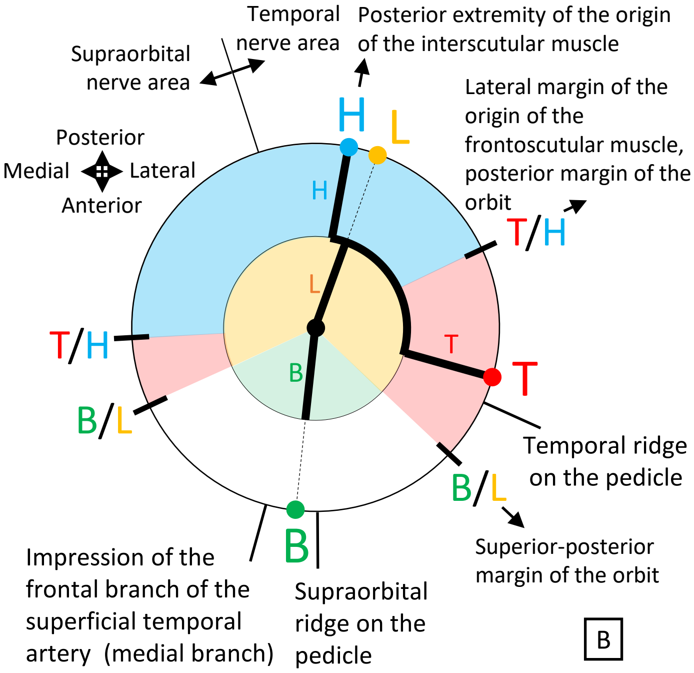

Figure 3. *Axis porcinus* (NSMT-M19849) A) Left antler, lateral view. B) Diagram of the left antler.

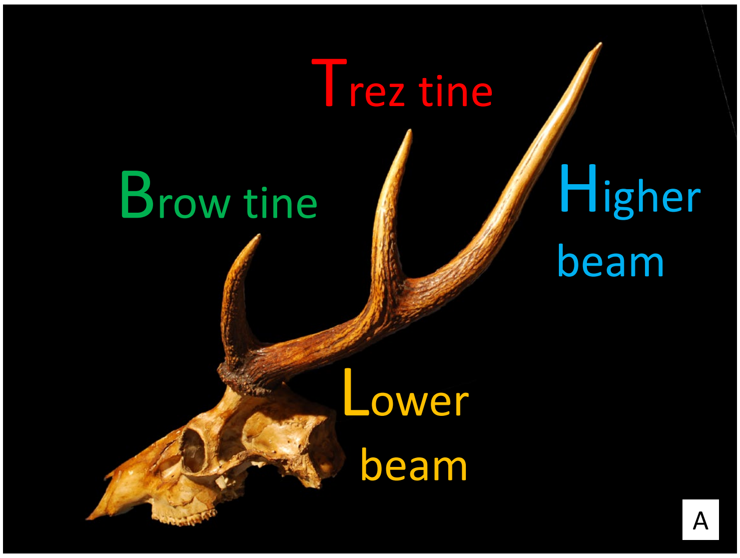

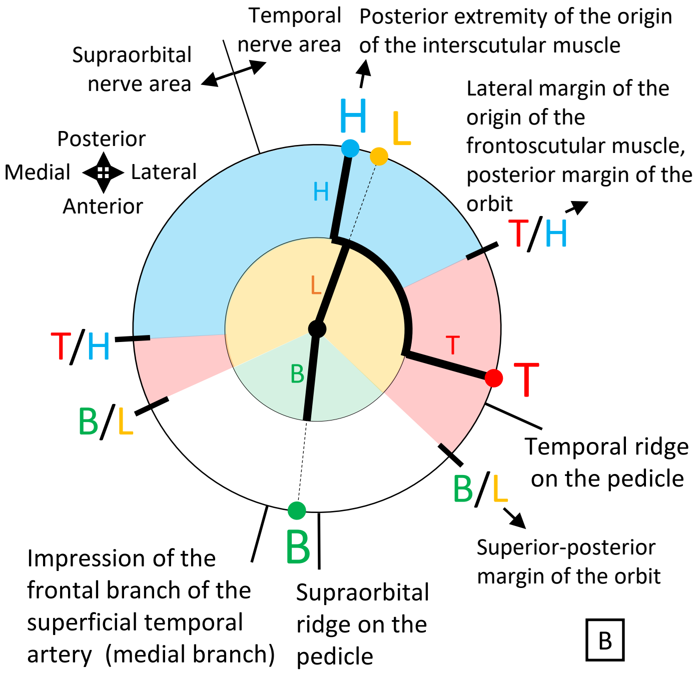

Figure 4. *Rusa timorensis* (KUGM-RM021) A) Left antler, lateral view. B) Diagram of the left antler.

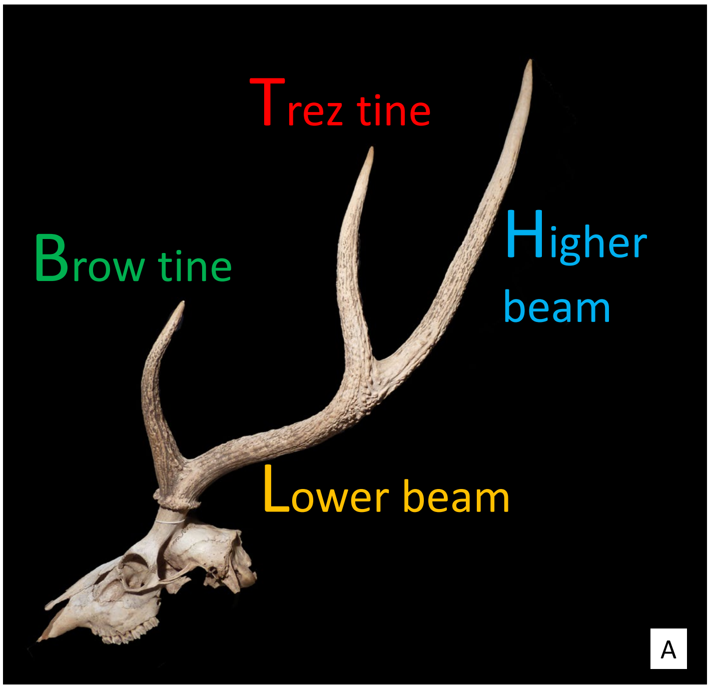

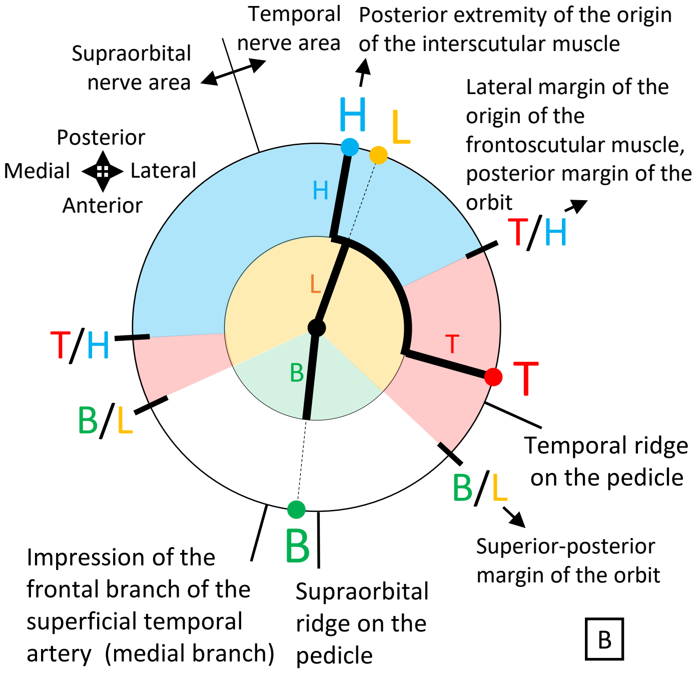

Figure 5. *Rusa unicolor* (KUGM-RM020) A) Left antler, lateral view. B) Diagram of the left antler.

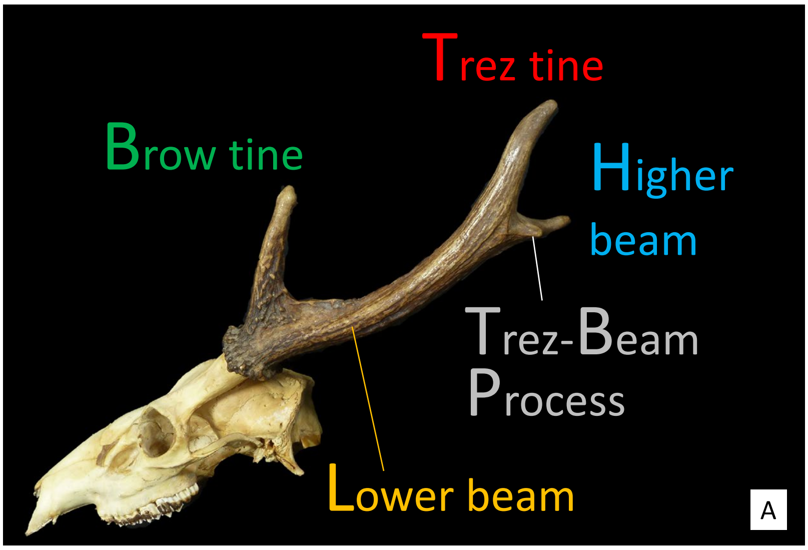

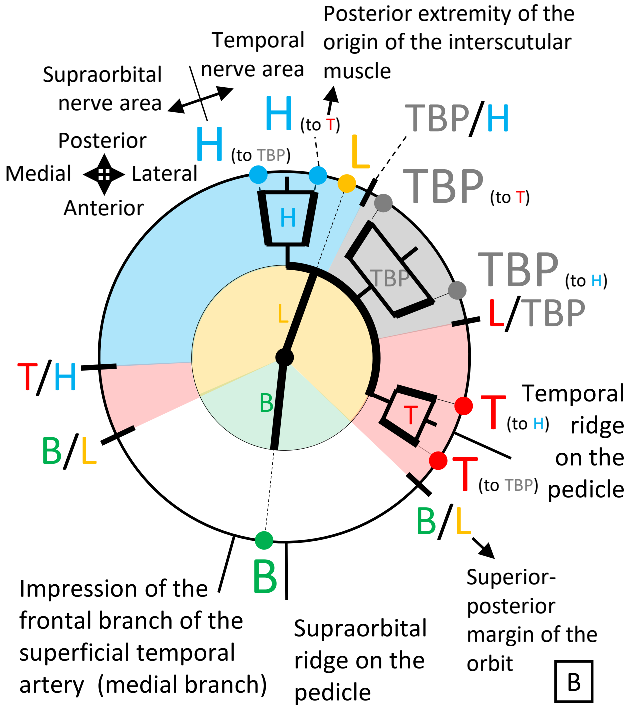

Figure 6. *Rusa marianna* (NSMT-M26698) A) Left antler, lateral view. B) Diagram of the left antler.

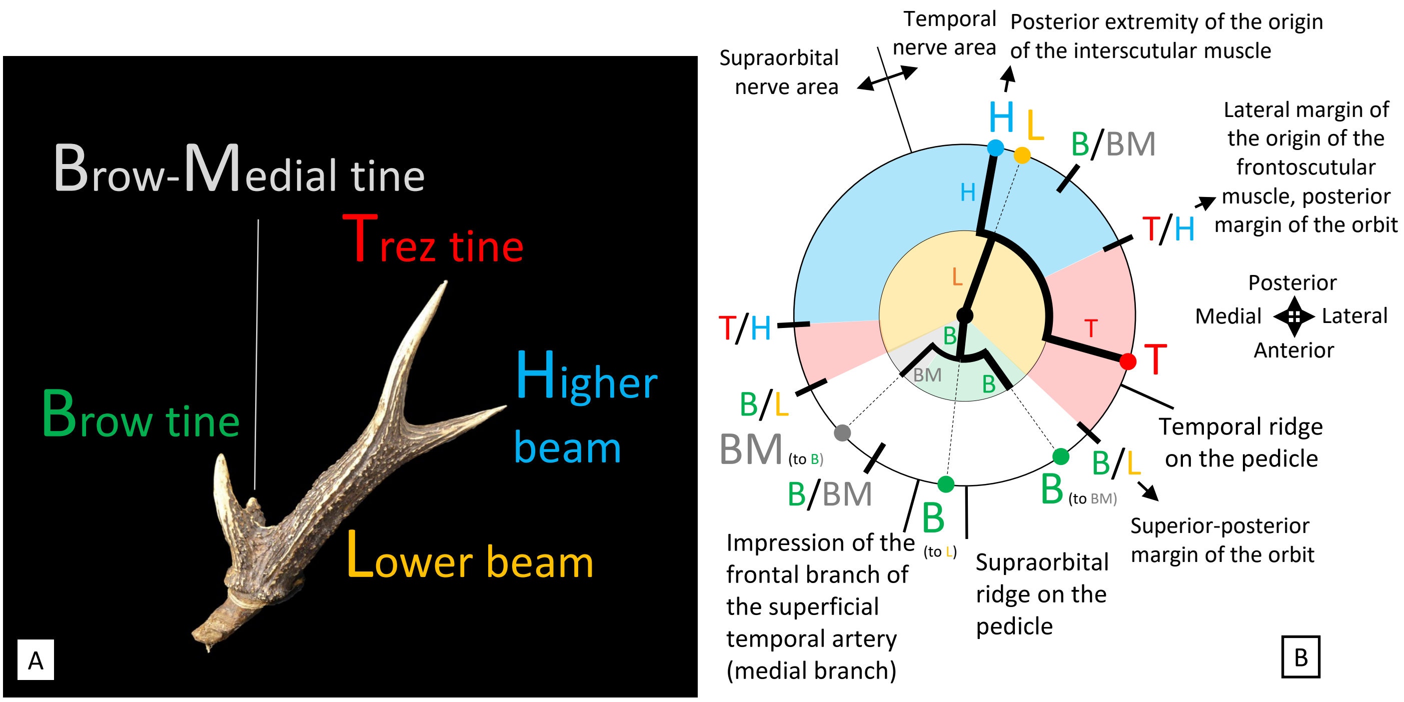

Figure 7. *Rusa mariaana* (NSMT-M08423) A) Left antler, lateral view. B) Diagram of the left antler.

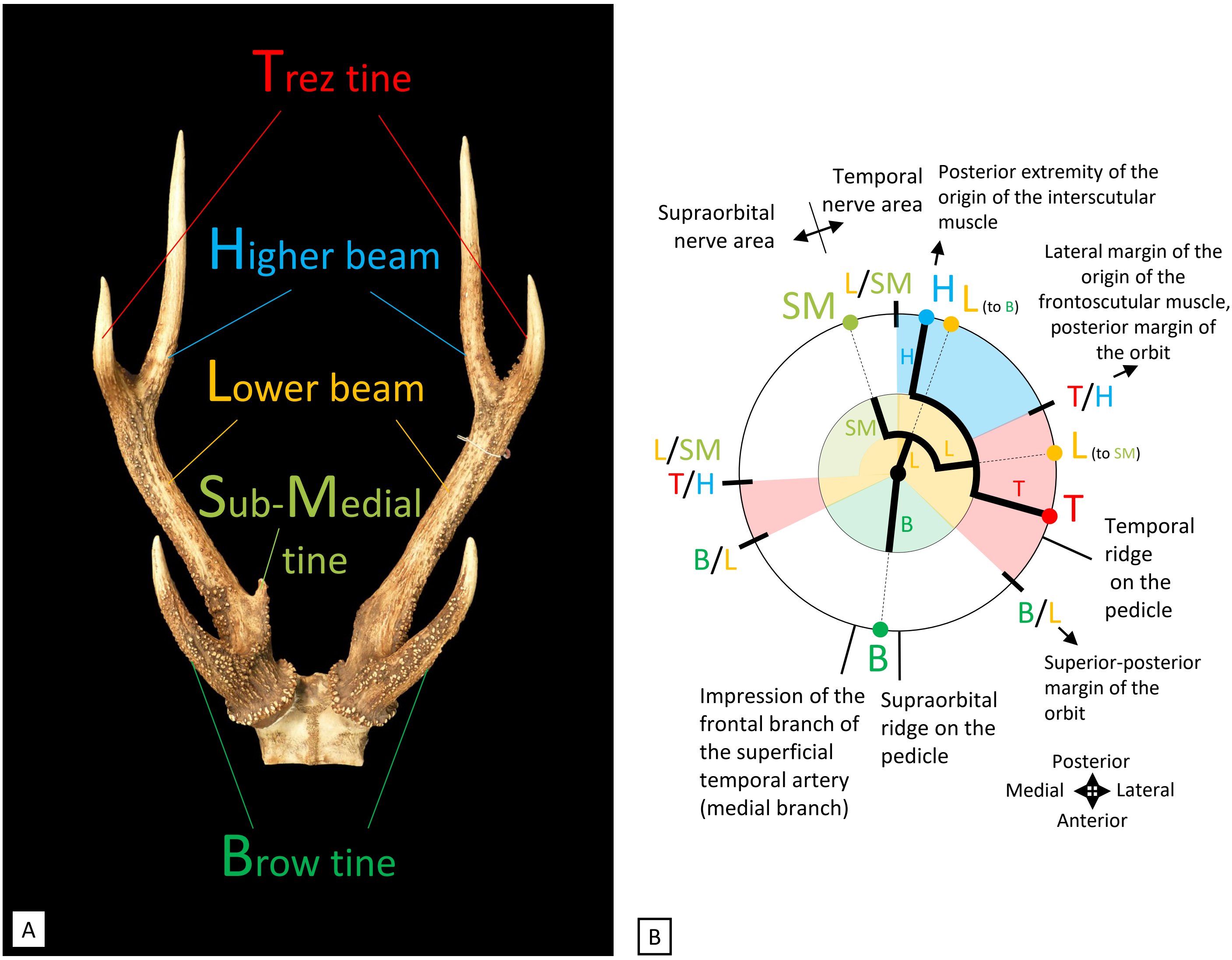

Figure 8. *Rusa* sp. (KUGM-RM212) A) Dorsal view. B) Diagram of the right antler (horizontally flipped) .

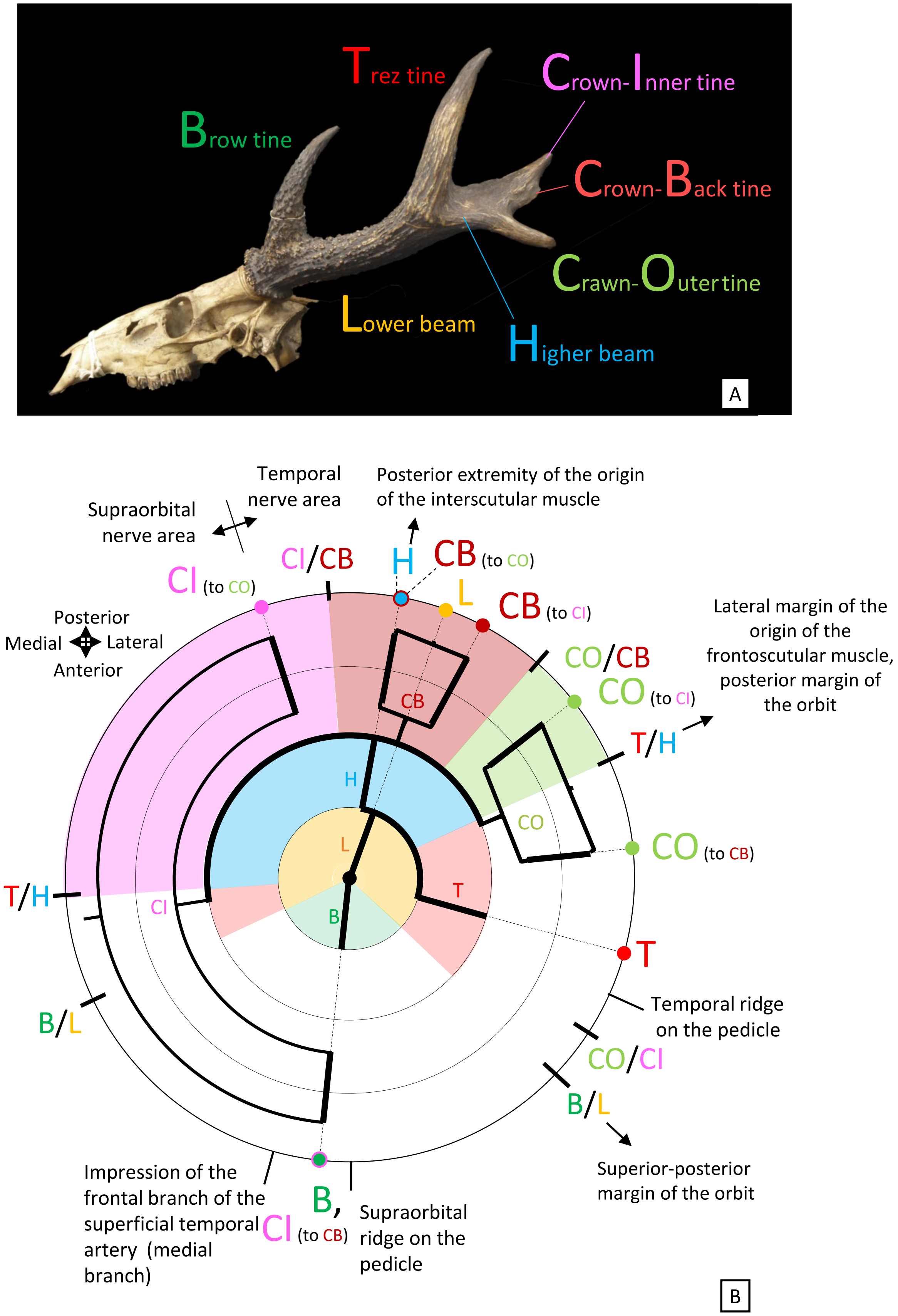

Figure 9. Non-typical antler of *Rusa unicolor* (NSMT-M08932) A) Left antler, lateral view. B) Diagram of the left antler.

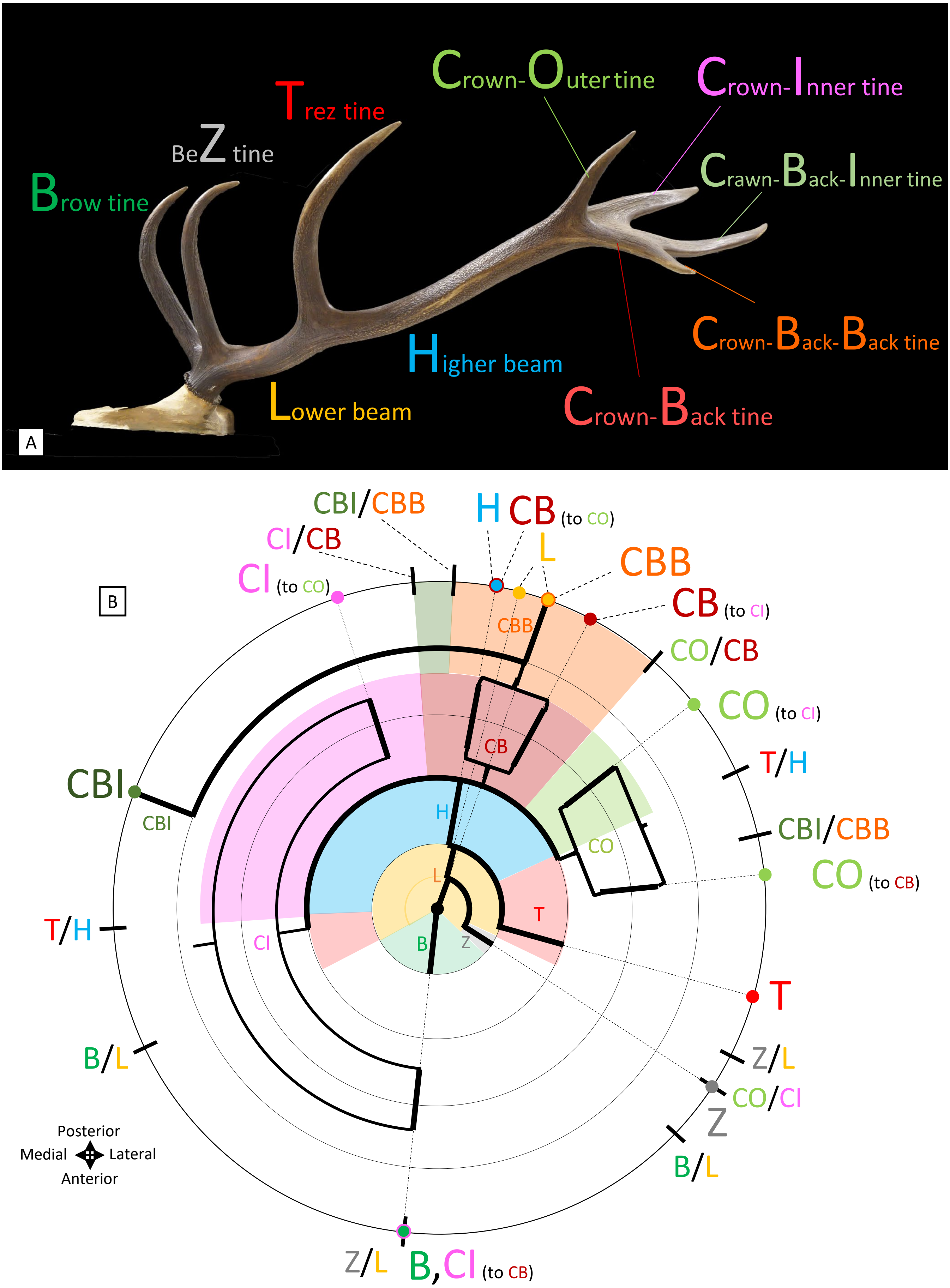

Figure 10. *Cervus elaphus* (NSMT-M01162) A) Left antler, lateral view. B) Diagram of the left antler.

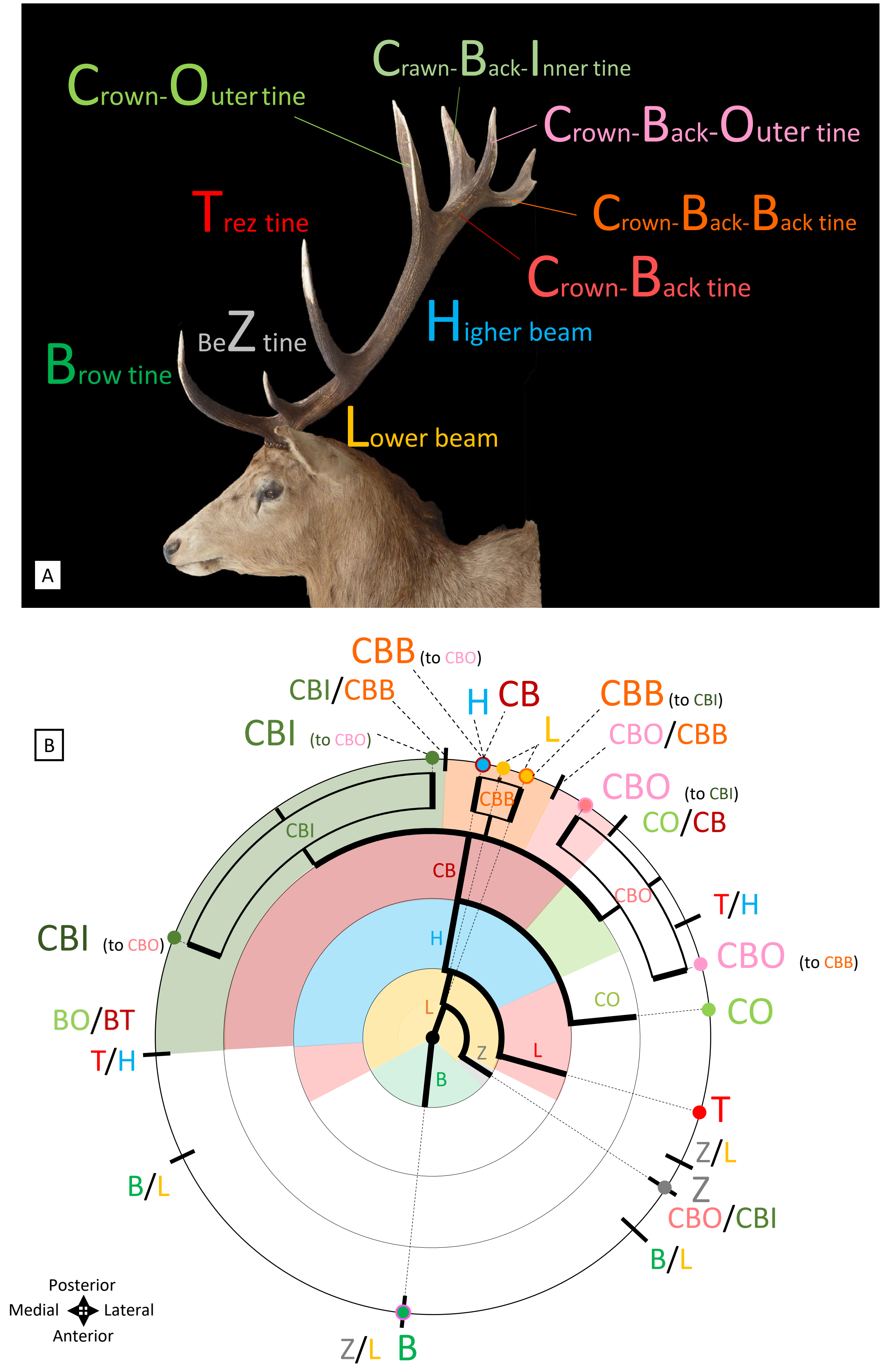

Figure 11. *Cervus elaphus* (NSMT-M56401) A) Left antler, lateral view. B) Diagram of the left antler.

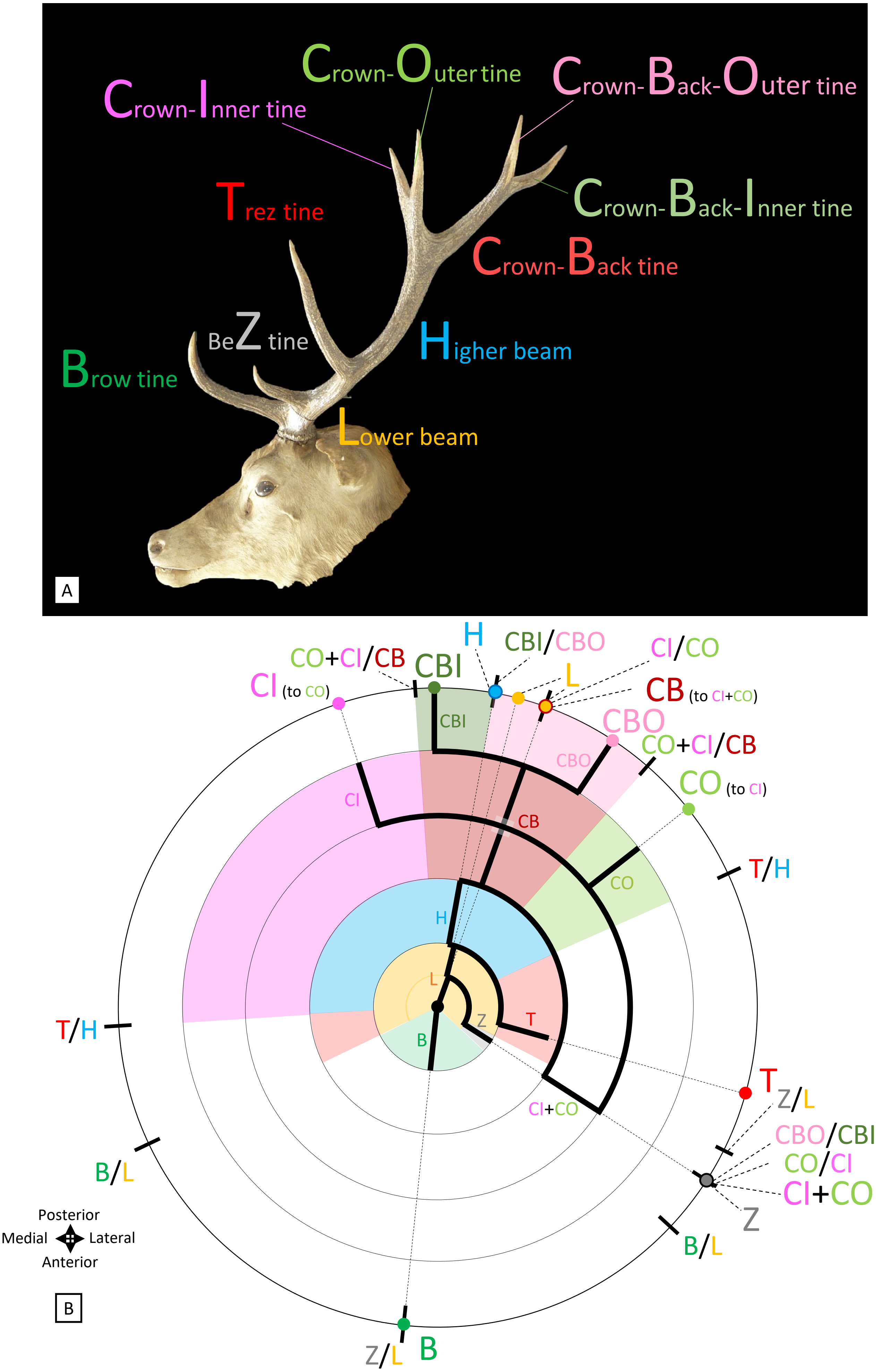

Figure 12. *Cervus elaphus* (NSMT-M43325) A) Left antler, lateral view. B) Diagram of the left antler.

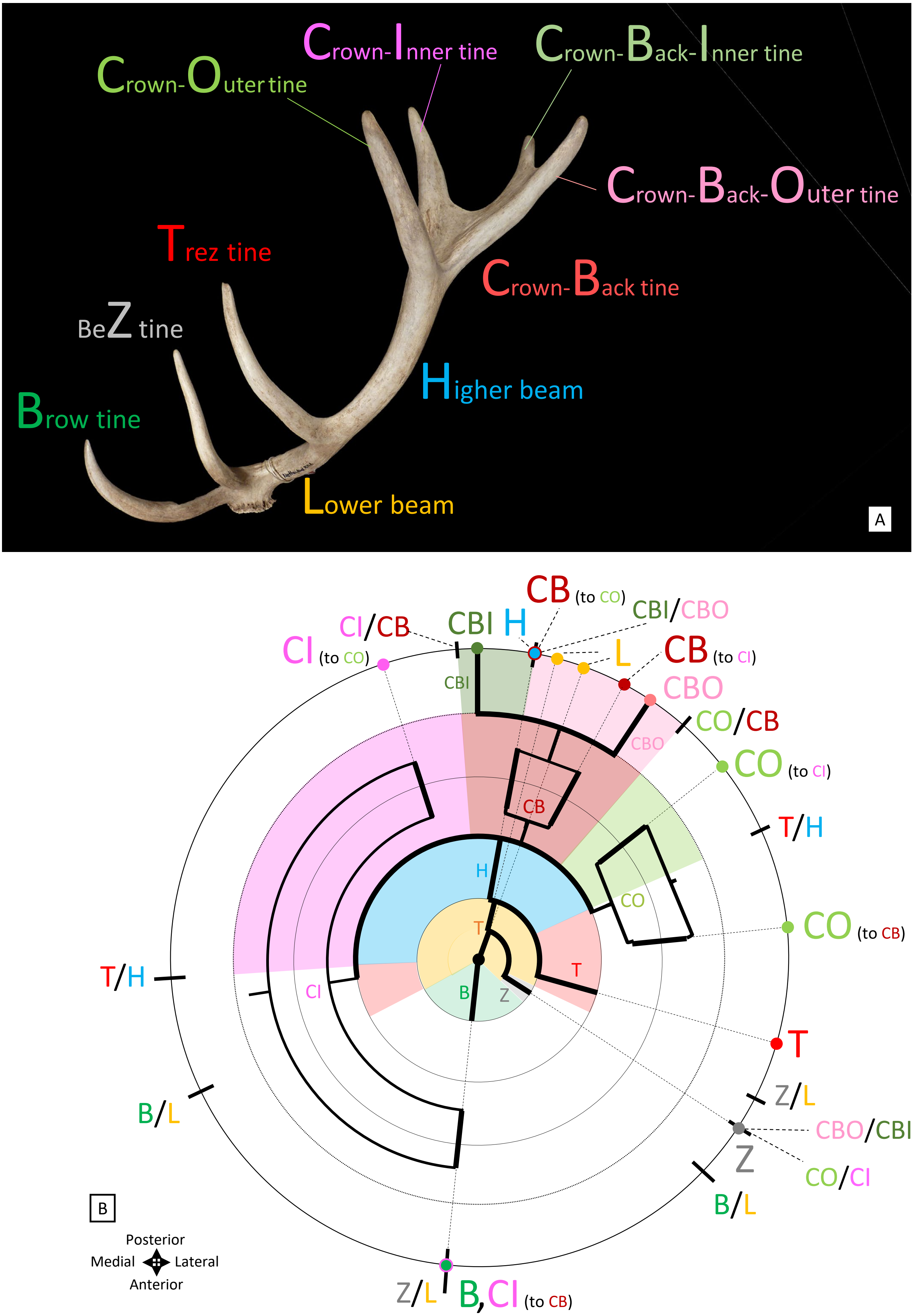

Figure 13 *Cervus elaphus* (KUGM-RM048) A) Left antler, lateral view. B) Diagram of the left antler.

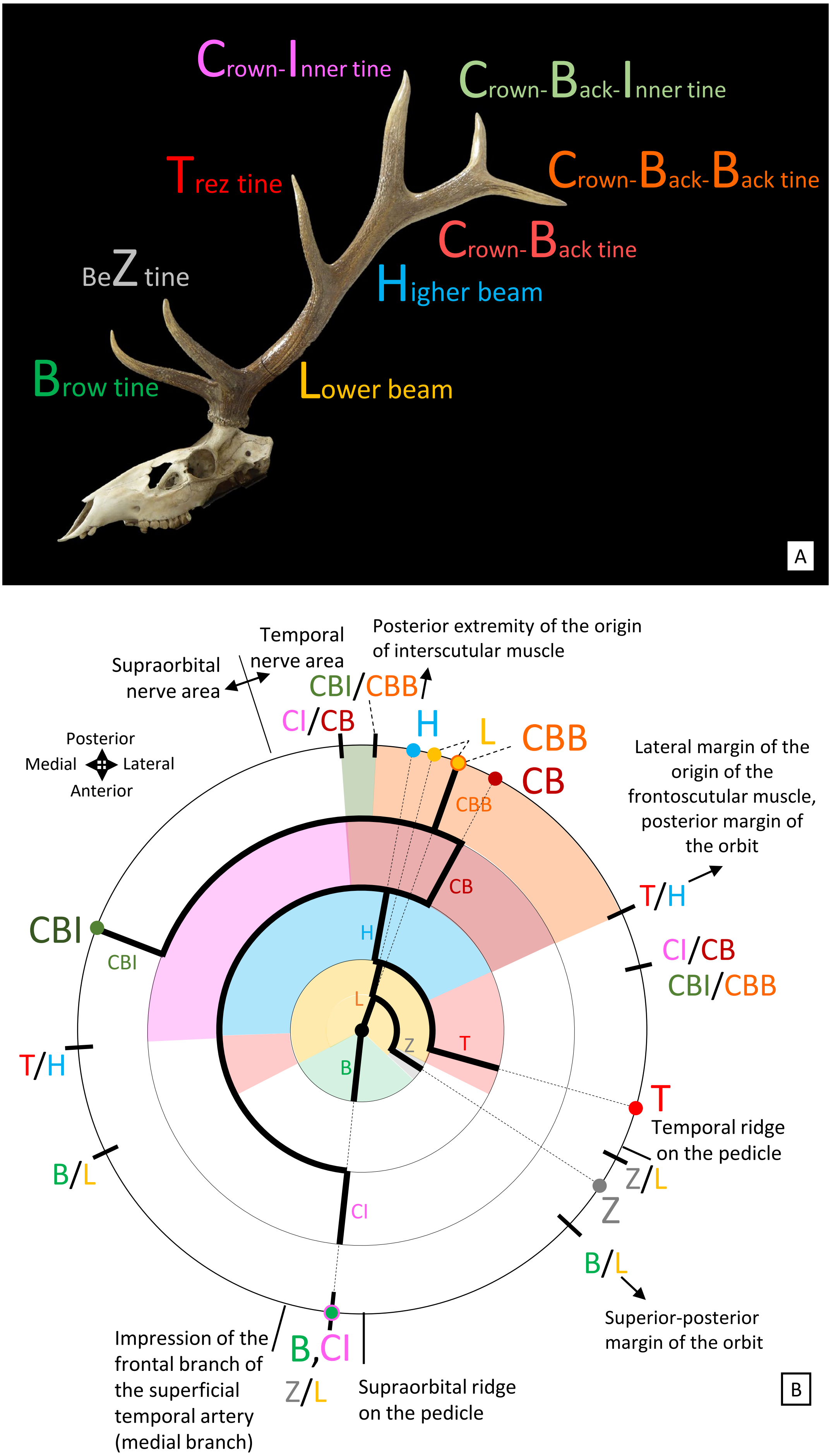

Figure 14. *Cervus canadensis* (NSMT-M43324) A) Left antler, lateral view. B) Diagram of the left antler.

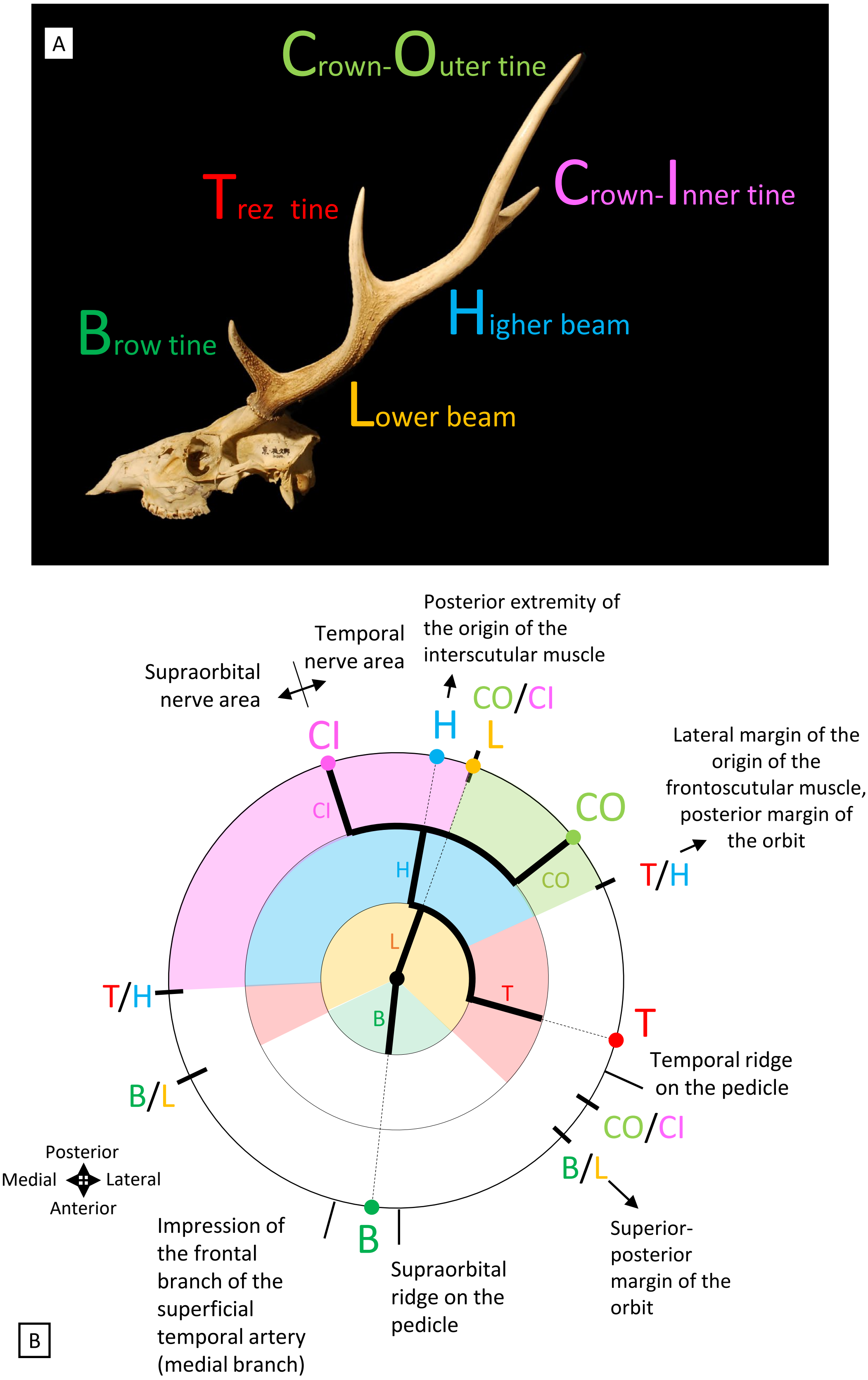

Figure 15. Typical antler of *Cervus nippon* (KUGM-RM003) A) Left antler, lateral view. B) Diagram of the left antler.

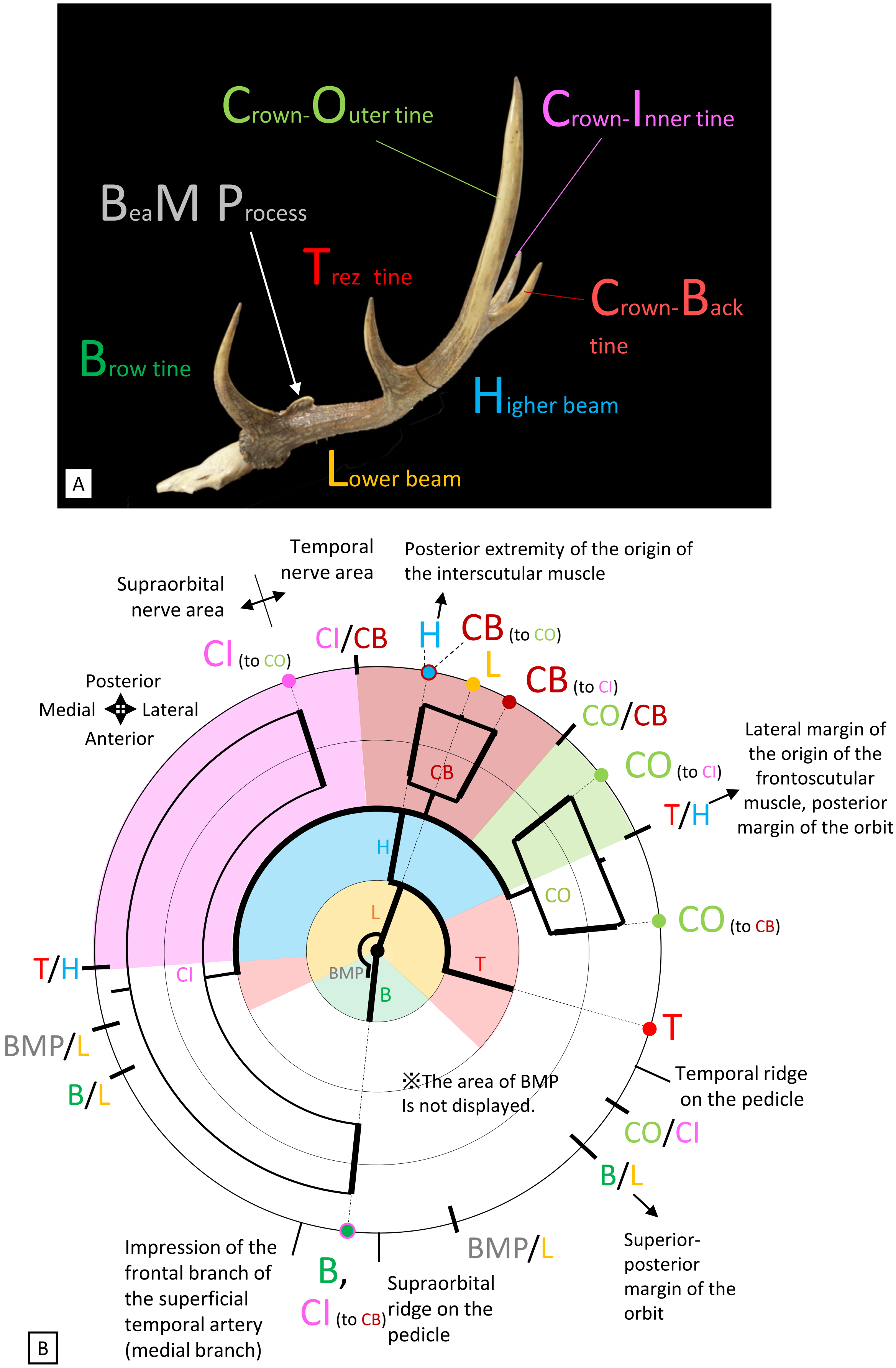

Figure 16. Non-typical antler of *Cervus nippon* (NSMT-M43307) A) Right antler, lateral view (horizontally flipped) . B) Diagram of the right antler (horizontally flipped) .

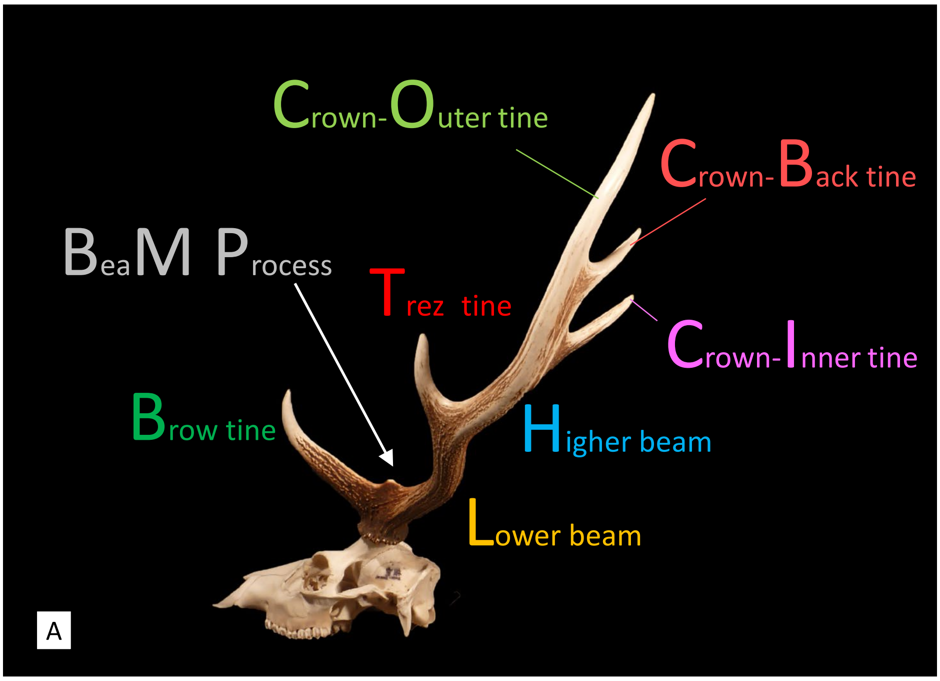

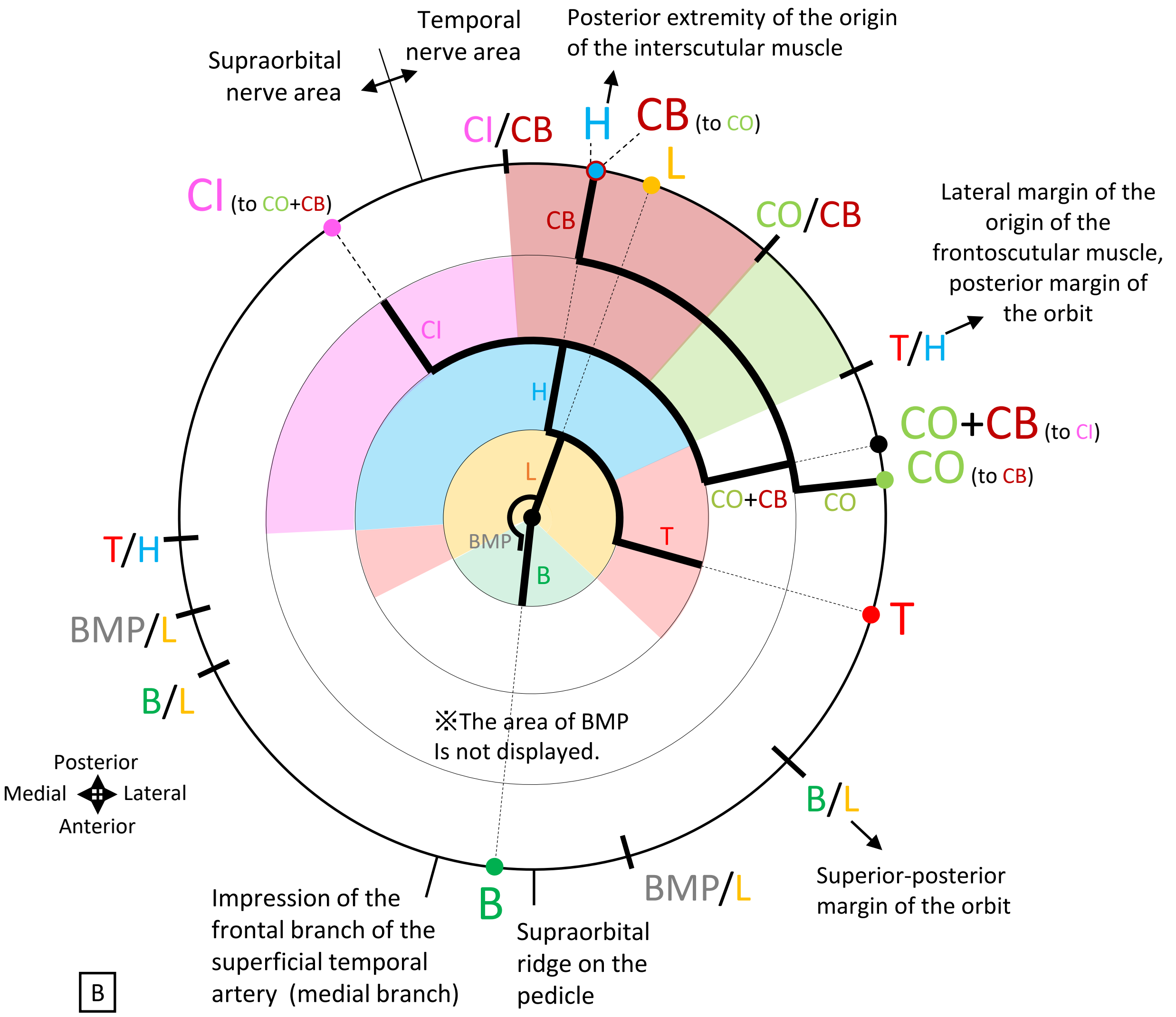

Figure 17. Non-typical antler of *Cervus nippon* (KUGM-RM016) A) Right antler, lateral view (horizontally flipped) . B) Diagram of the right antler (horizontally flipped) .

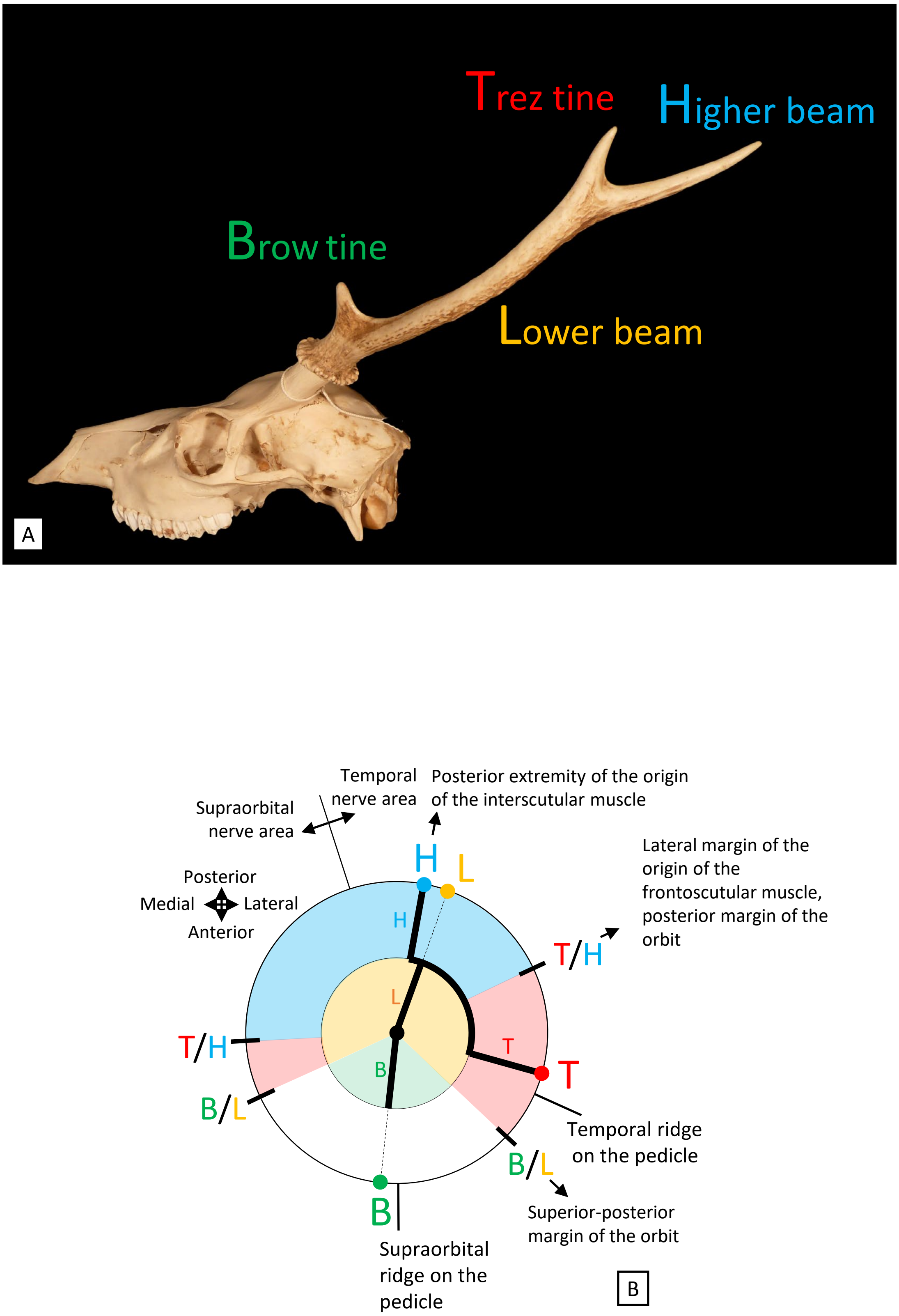

Figure 18. A) A juvenile *Cervus nippon* (KUGM-RM008) . Estimated 2 to 3 years old. Left antler, lateral view. **B)** Diagram of the left antler.

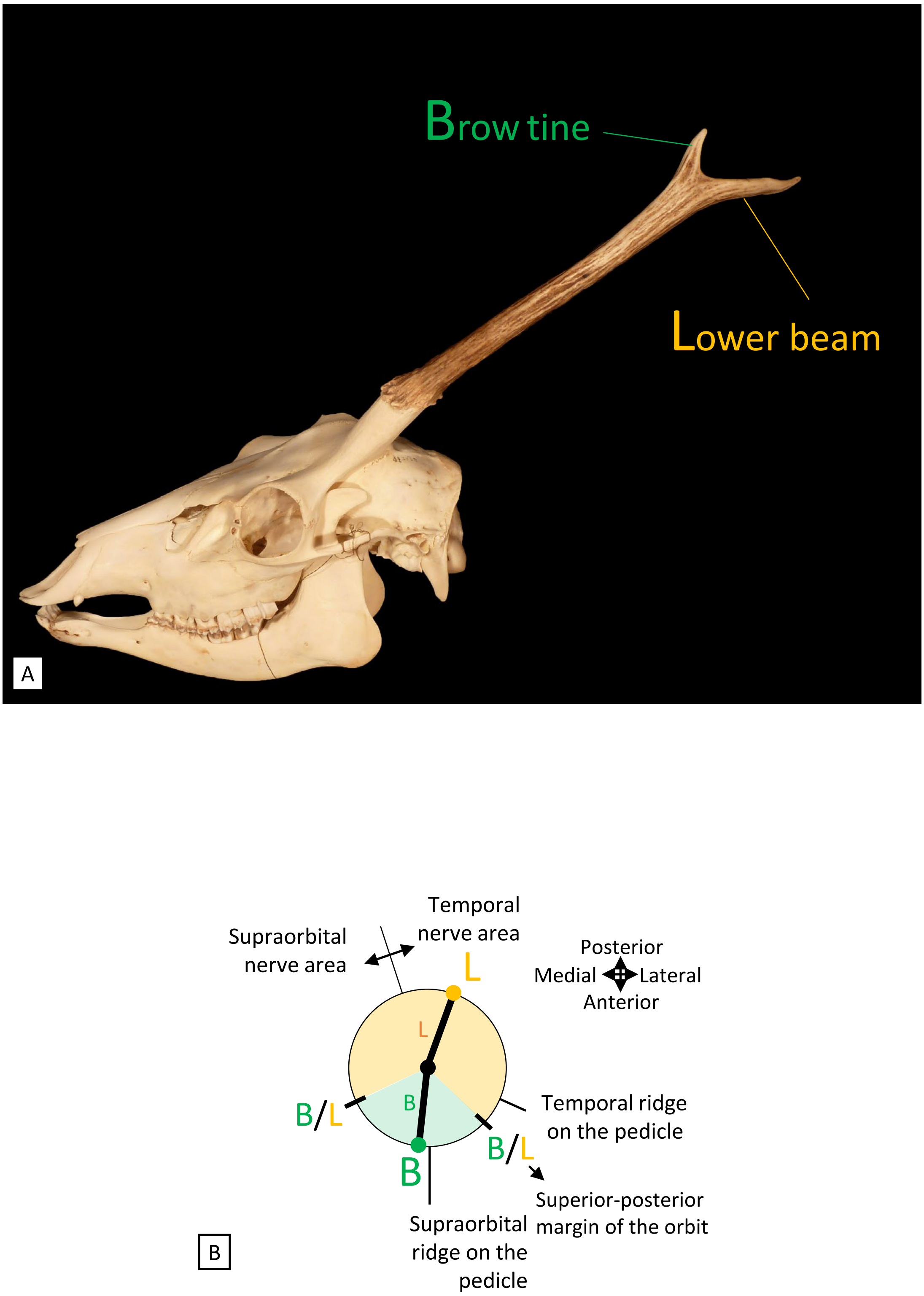

Figure 19. A) A juvenile *Cervus nippon* (KUGM-RM009) . Estimated 1 to 2 years old. Left antler, lateral view. **B)** Diagram of the left antler.

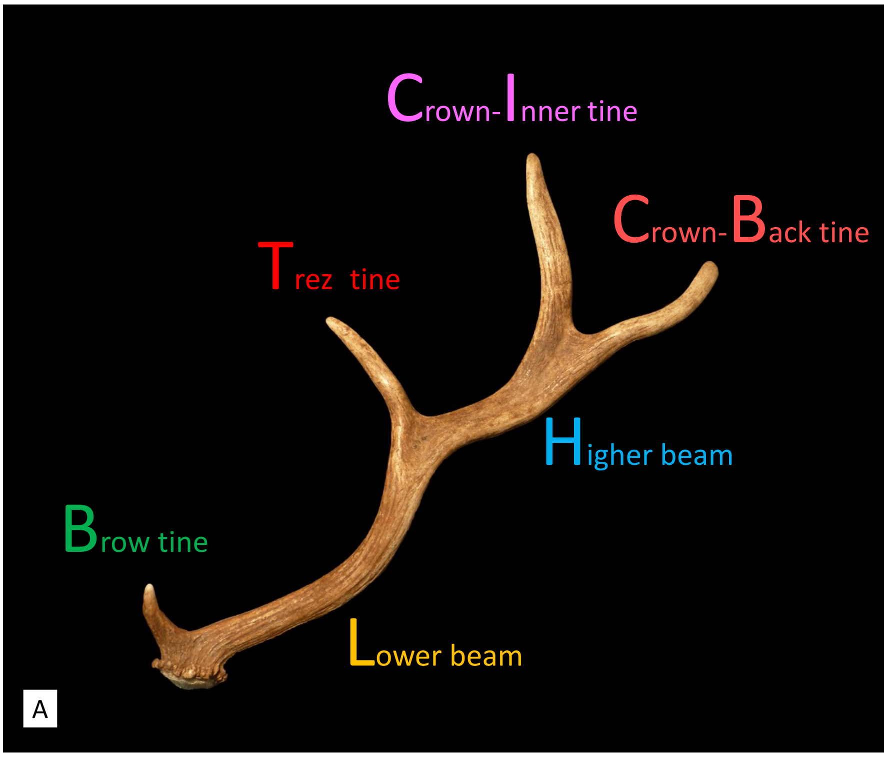

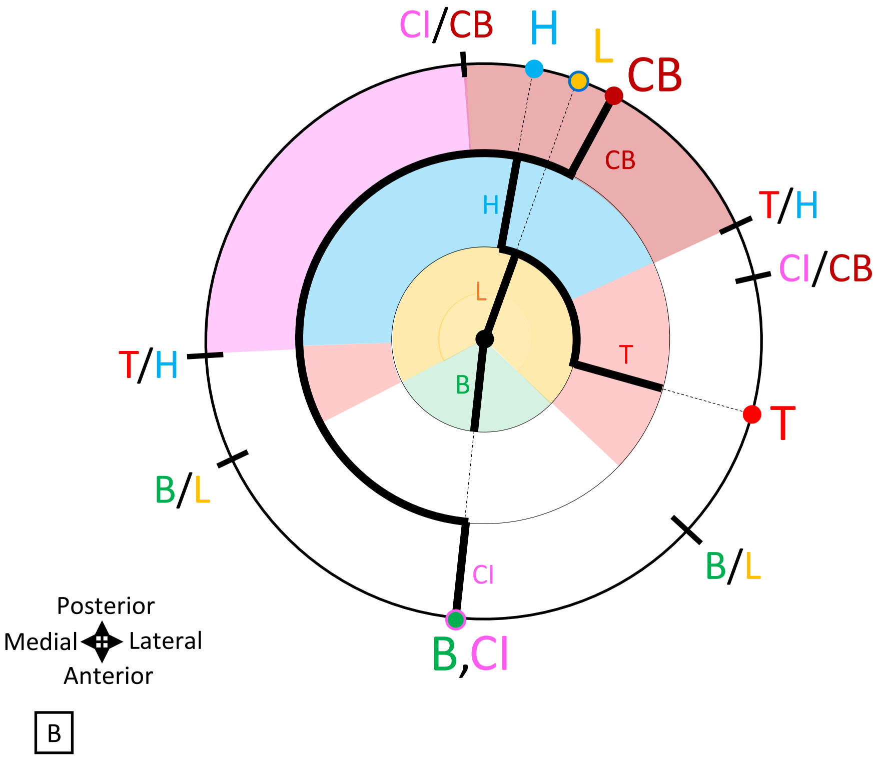

Figure 20. Juvenile antler of *Dama dama* (KUGM-RM204) A) Right antler, lateral view (horizontally flipped) . B) Diagram of the left antler (horizontally flipped) .

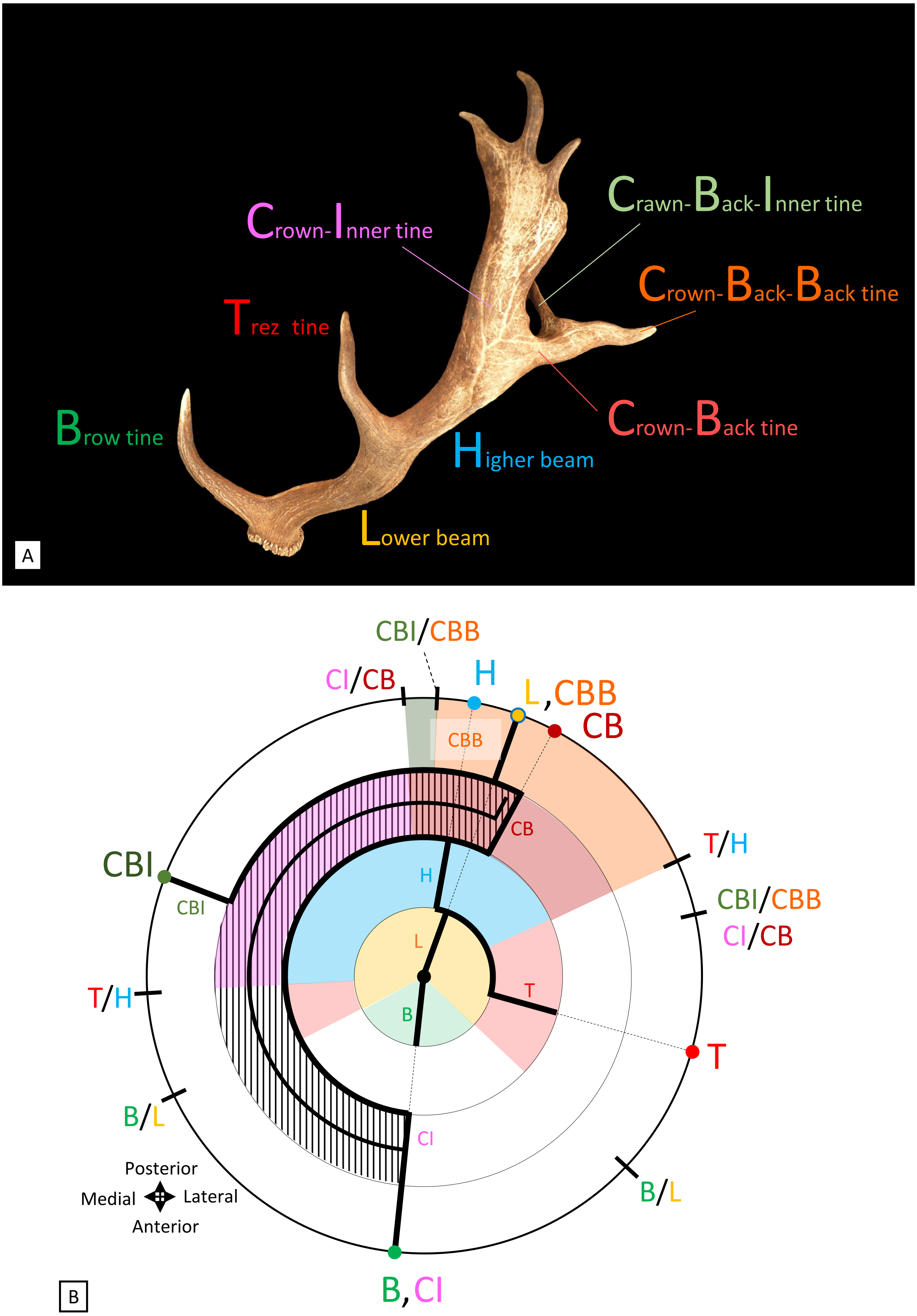

Figure 21. *Dama dama* (KUGM-RM107) A) Right antler, lateral view (horizontally flipped) . B) Diagram of the right antler (horizontally flipped) . Small tines in the palmate portion are omitted.

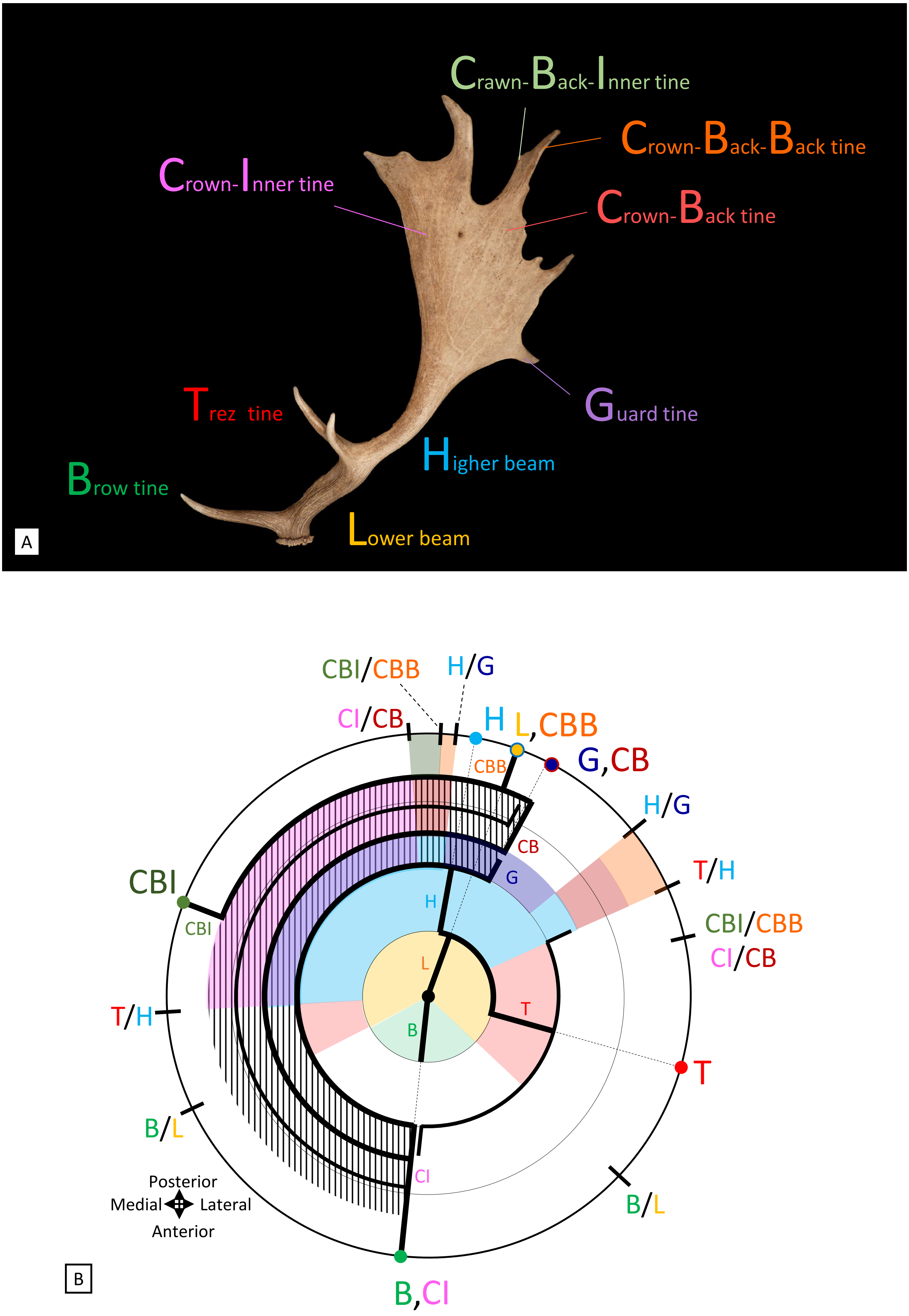

Figure 22. *Dama dama* (KUGM-RM144) A) Right antler, lateral view (horizontally flipped) . B) Diagram of the right antler (horizontally flipped) . Small tines in the palmate portion are omitted.

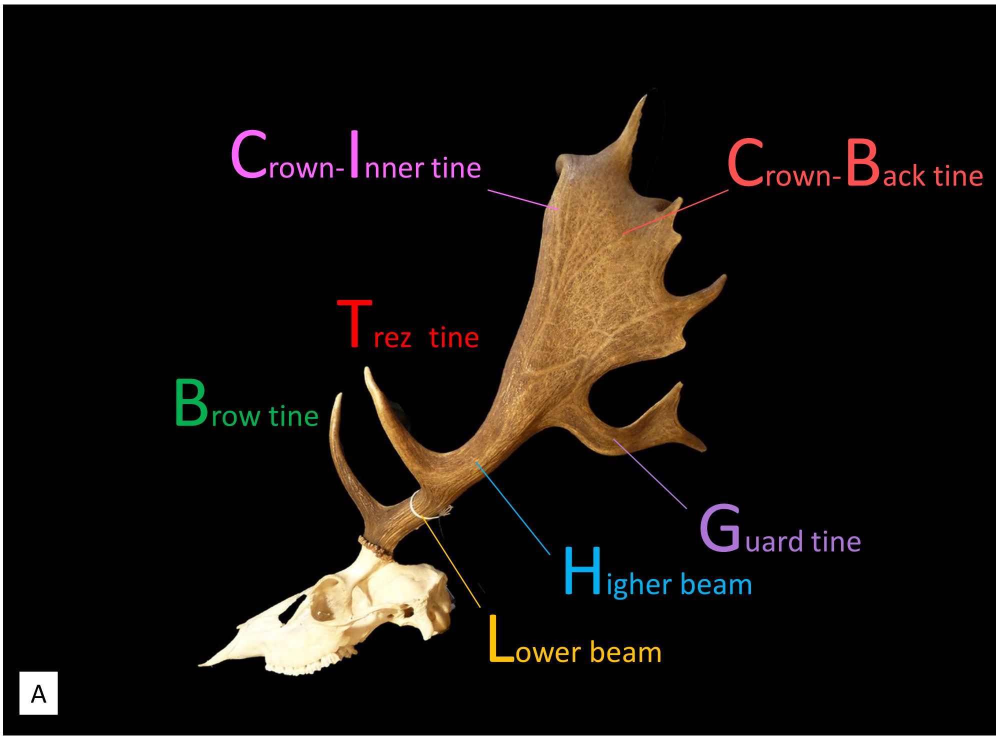

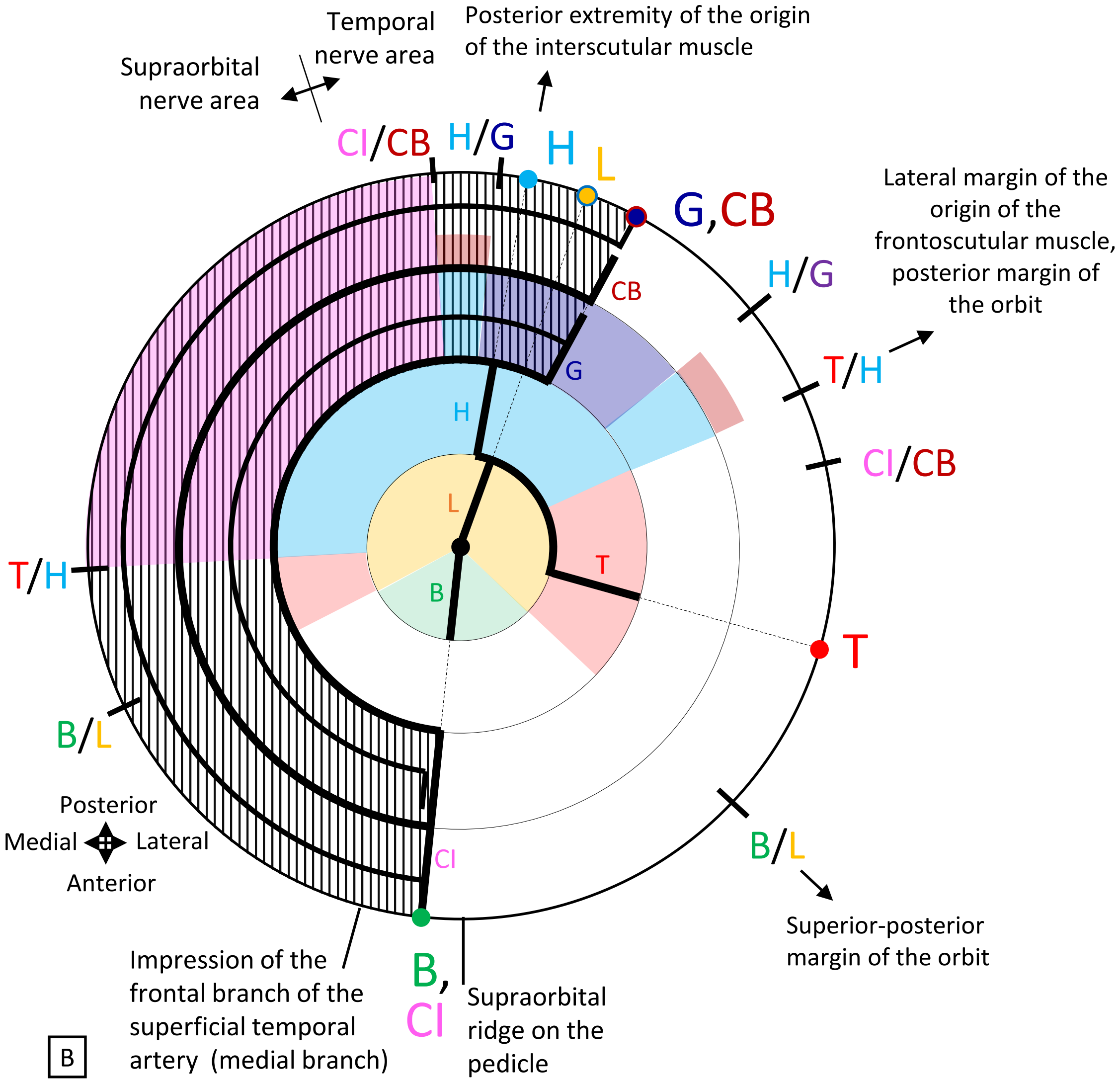

Figure 23. *Dama dama* (LBM-1900000787) A) Left antler, lateral view. B) The diagram of the left antler. Small tines in the palmate portion are omitted.

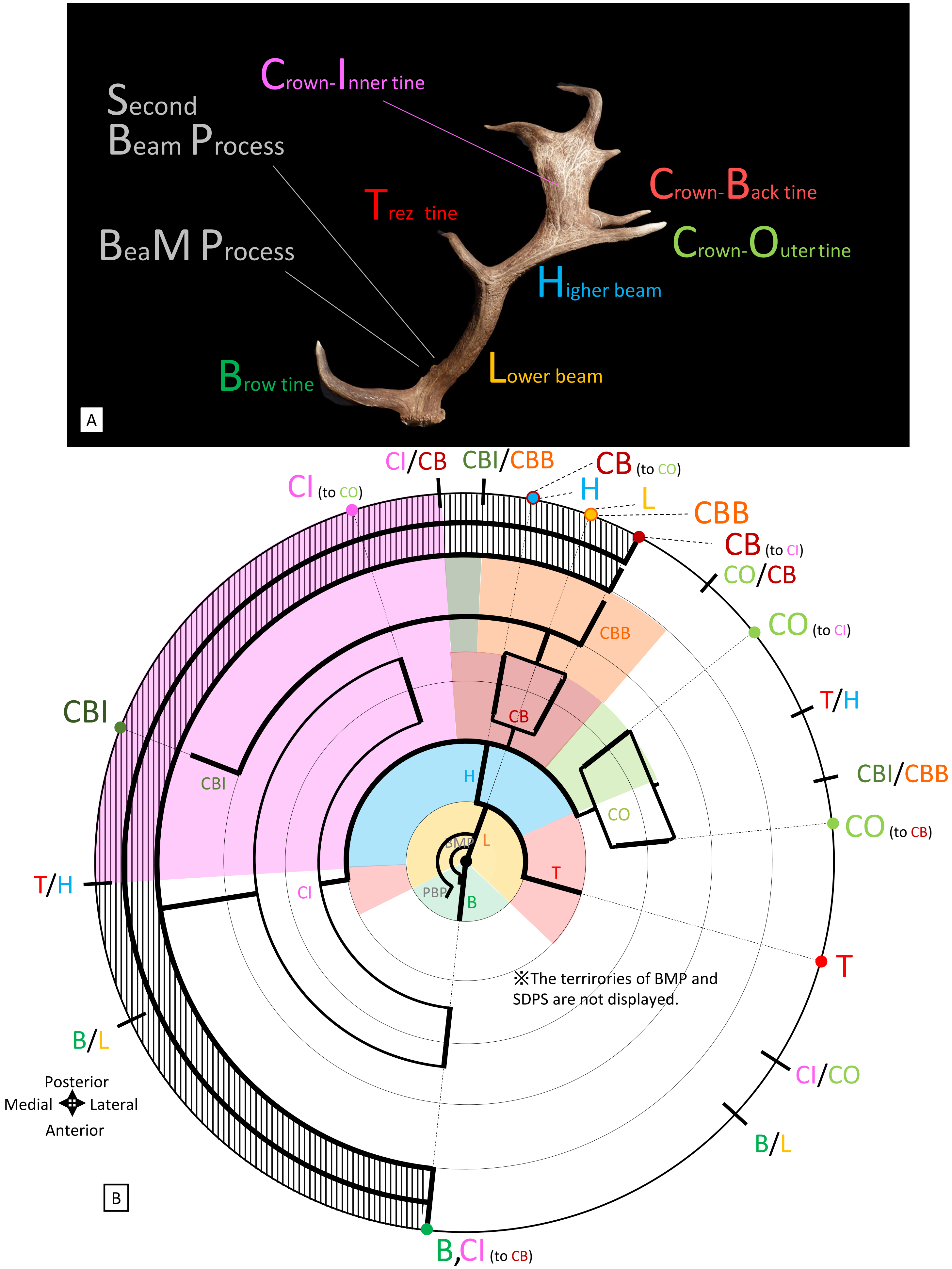

Figure 24. Non-typical antler of *Dama dama* (KUGM-RM107) A) Left antler, lateral view. B) The diagram of the left antler. Small tines in the palmate portion are omitted.

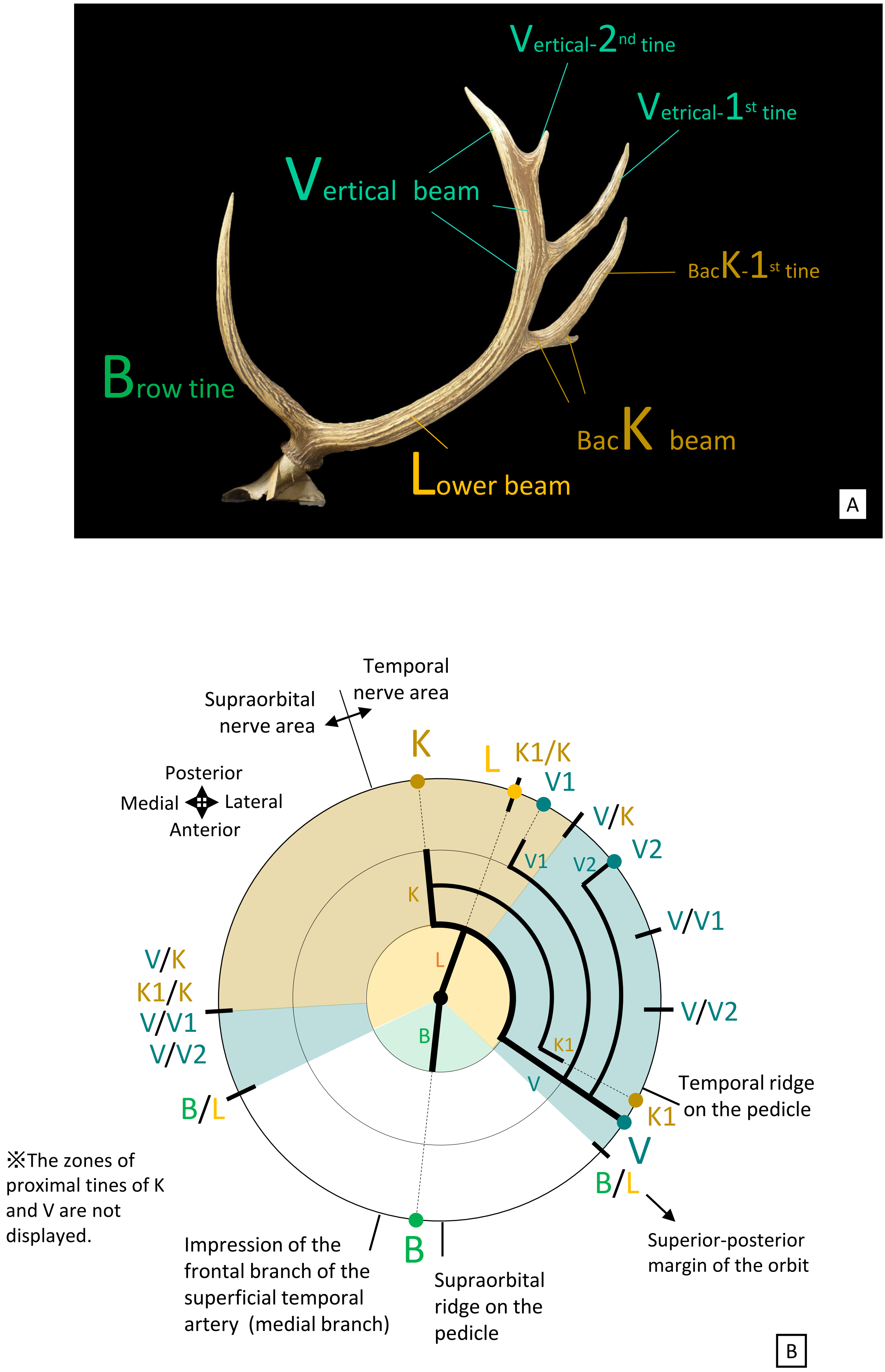

Figure 25. *Rucervus duvaucelii* (NSMT-M01152) A) Right antler, lateral view (horizontally flipped) . B) Diagram of the left antler (horizontally flipped) .

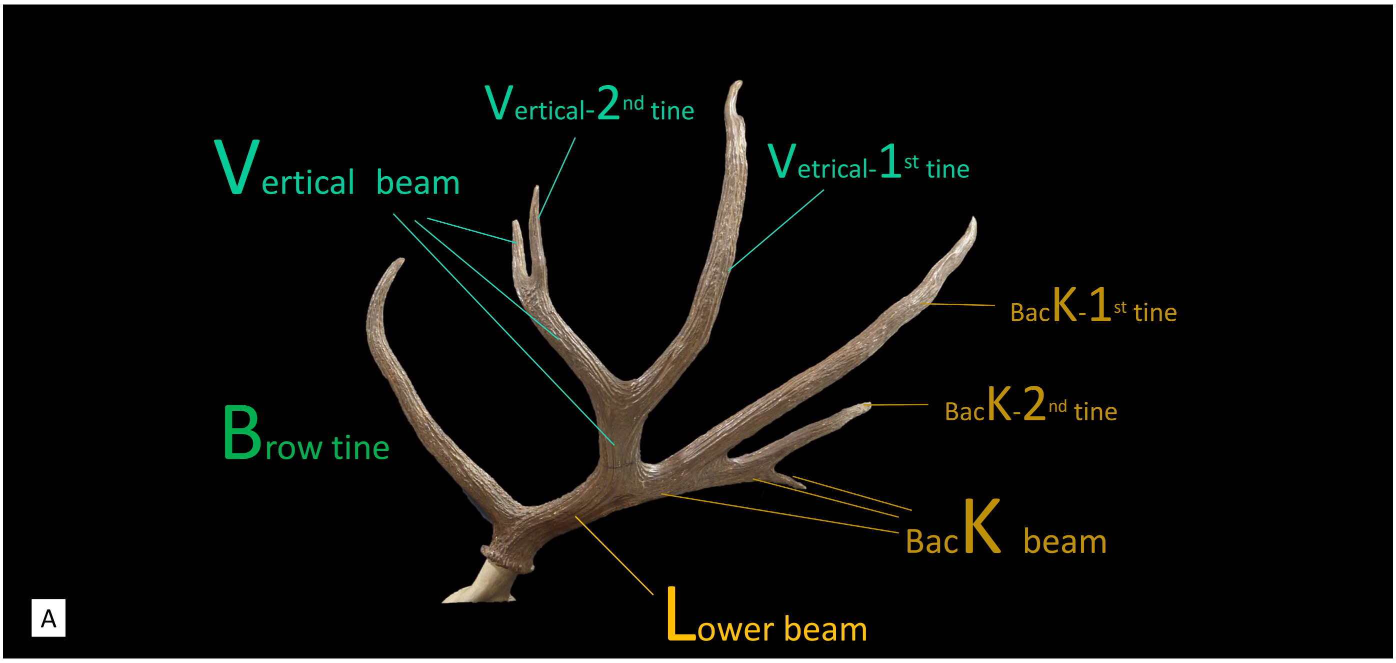

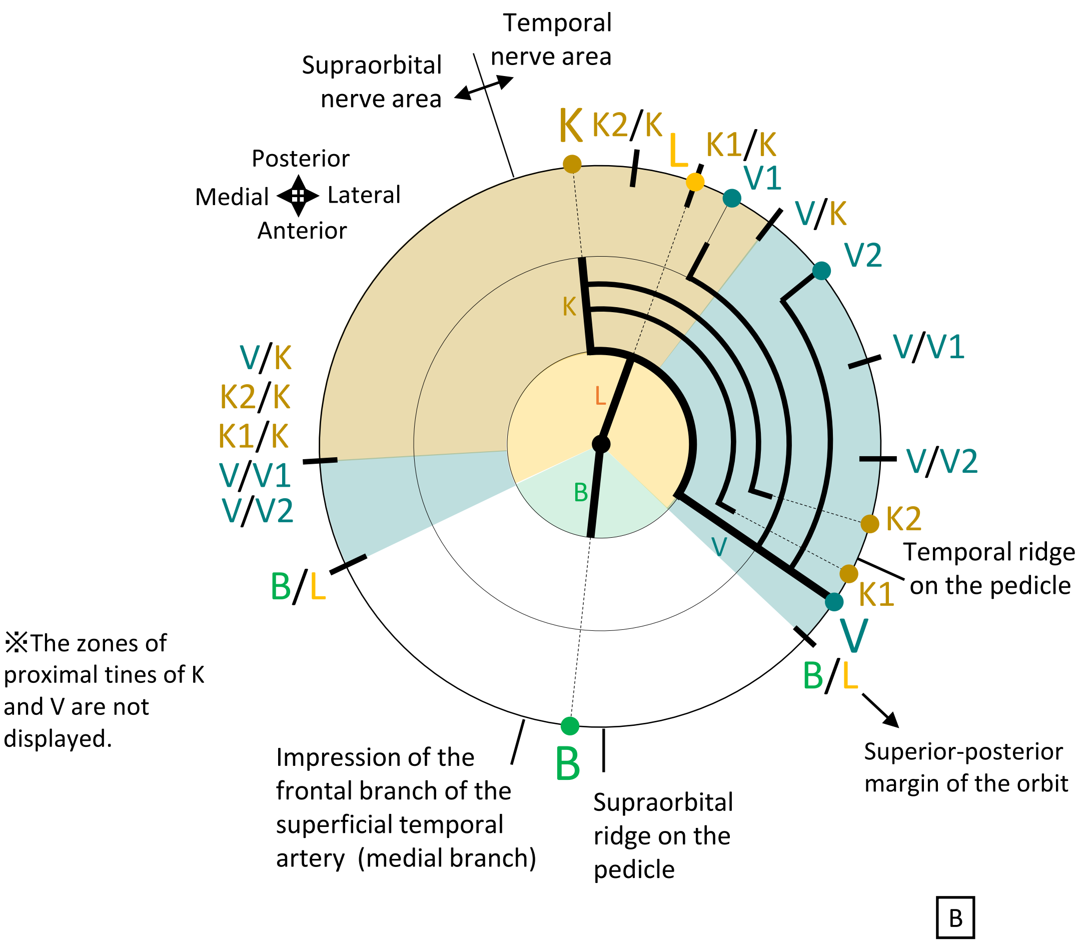

Figure 26. *Rucervus schomburgki* (NSMT-M01151) A) Right antler, lateral view (horizontaly flipped) . B) Diagram of the right antler (horizontaly flipped) .

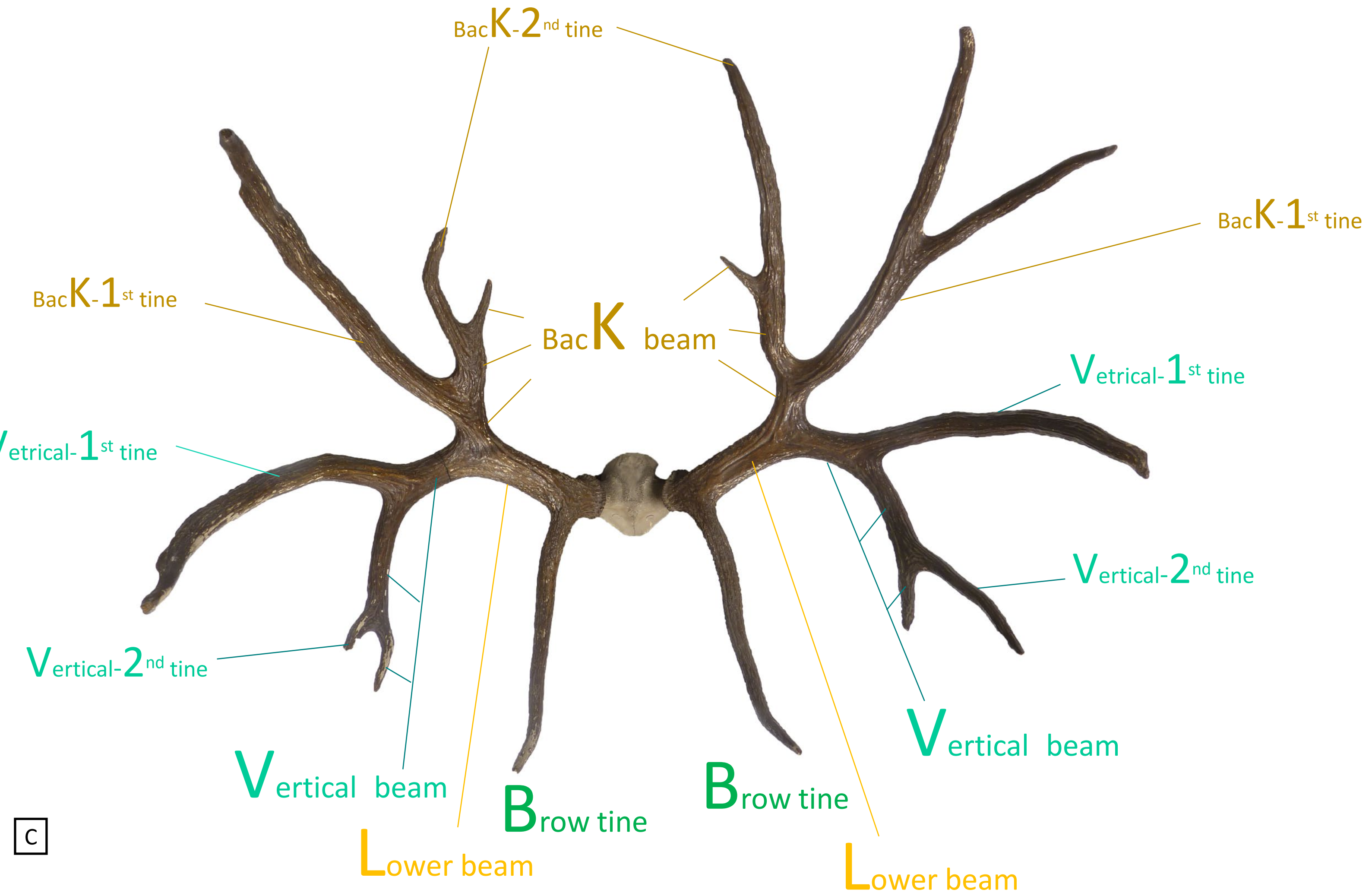

Figure 26. *Rucervus schomburgki* (NSMT-M01151) C) Dorsal view.

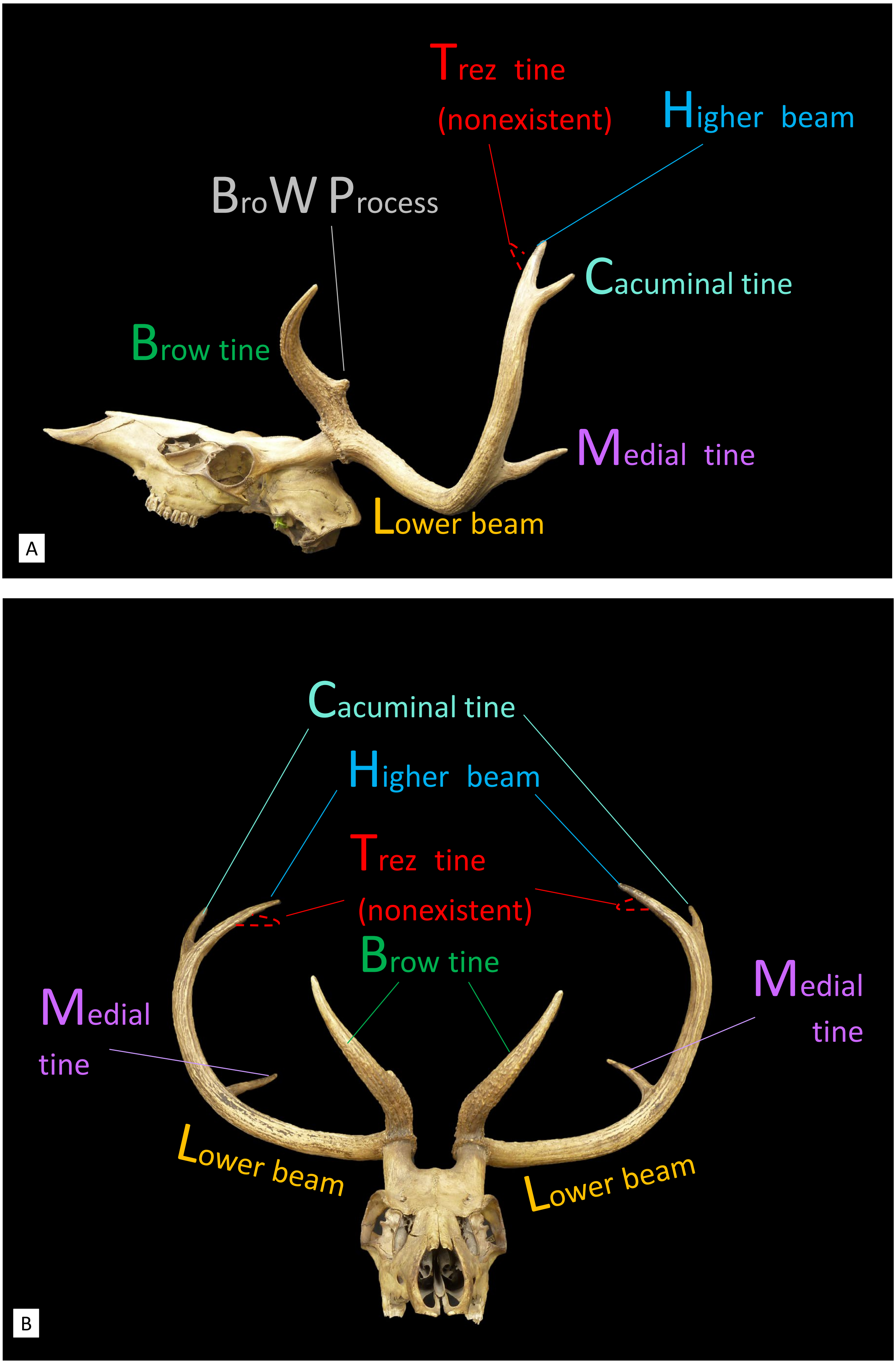

Figure 27. *Panolia eldii* (OMNH-M2034) A) Left antler, lateral view. B) Anterior view.

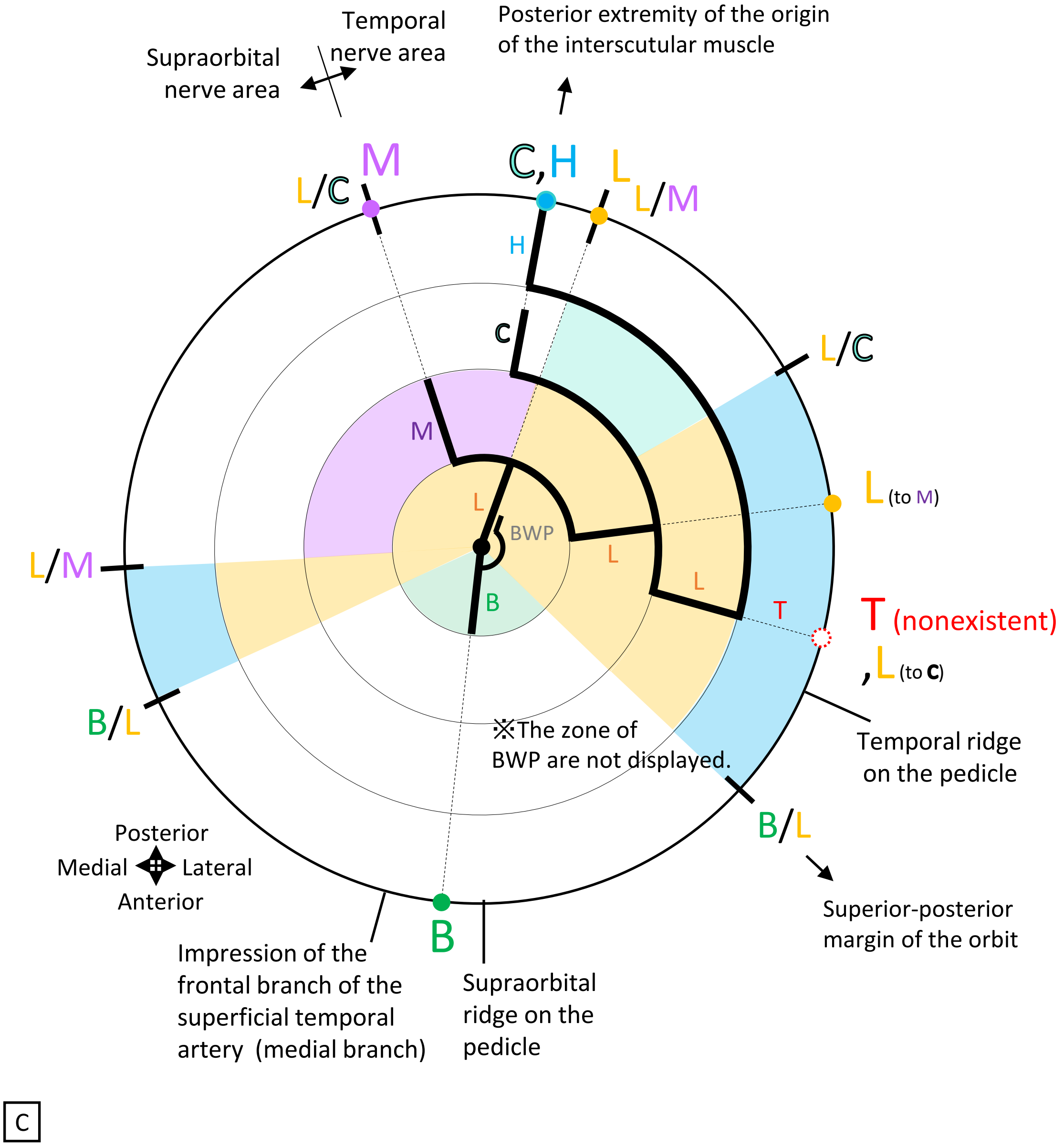

Figure 27. *Panolia eldii* (OMNH-M2034) C) Diagram of the left antler.

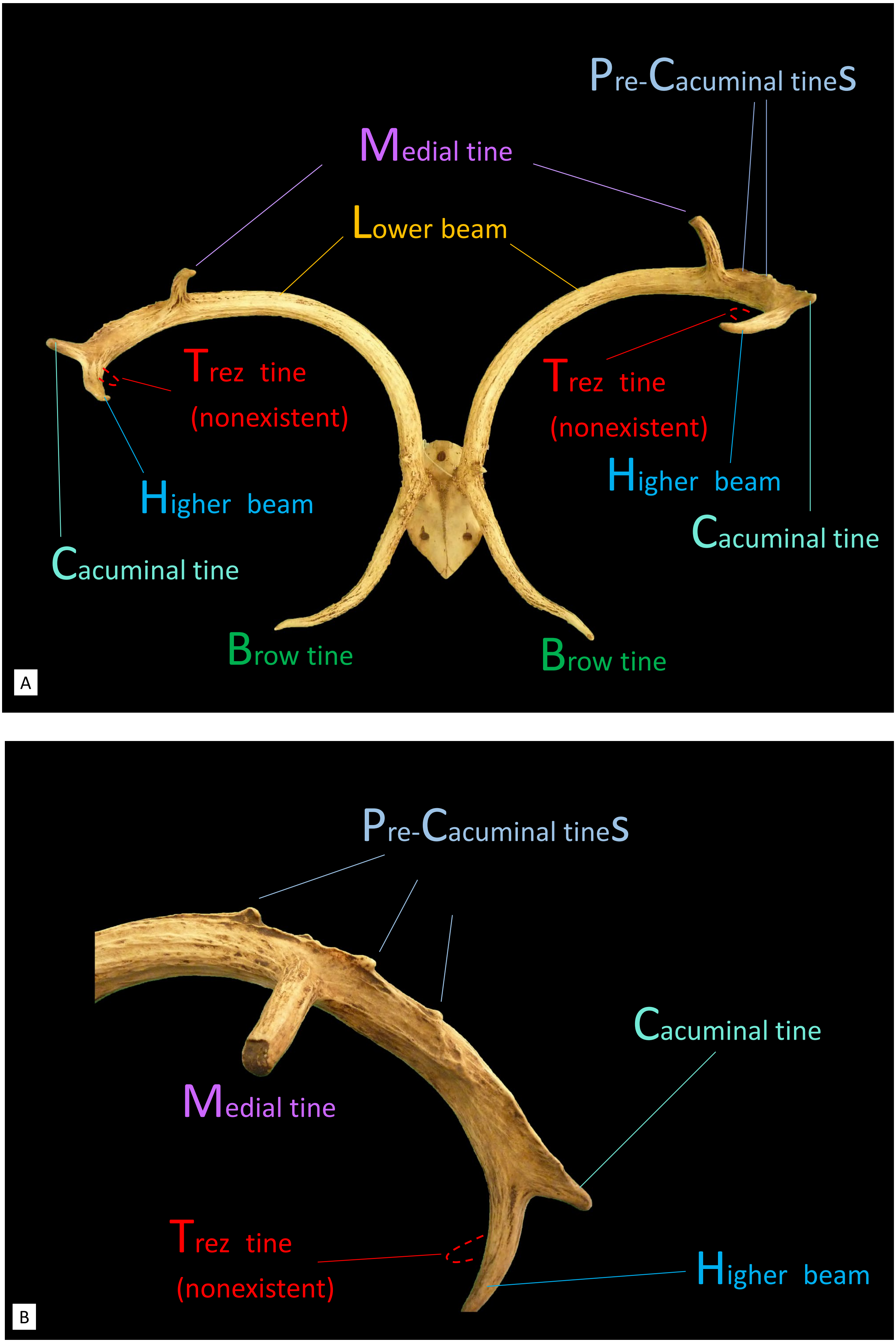

Figure 28. *Panolia eldii* (HUBG-10286) A) Dorsal view. B) Magnification of the distal part of the left antler.

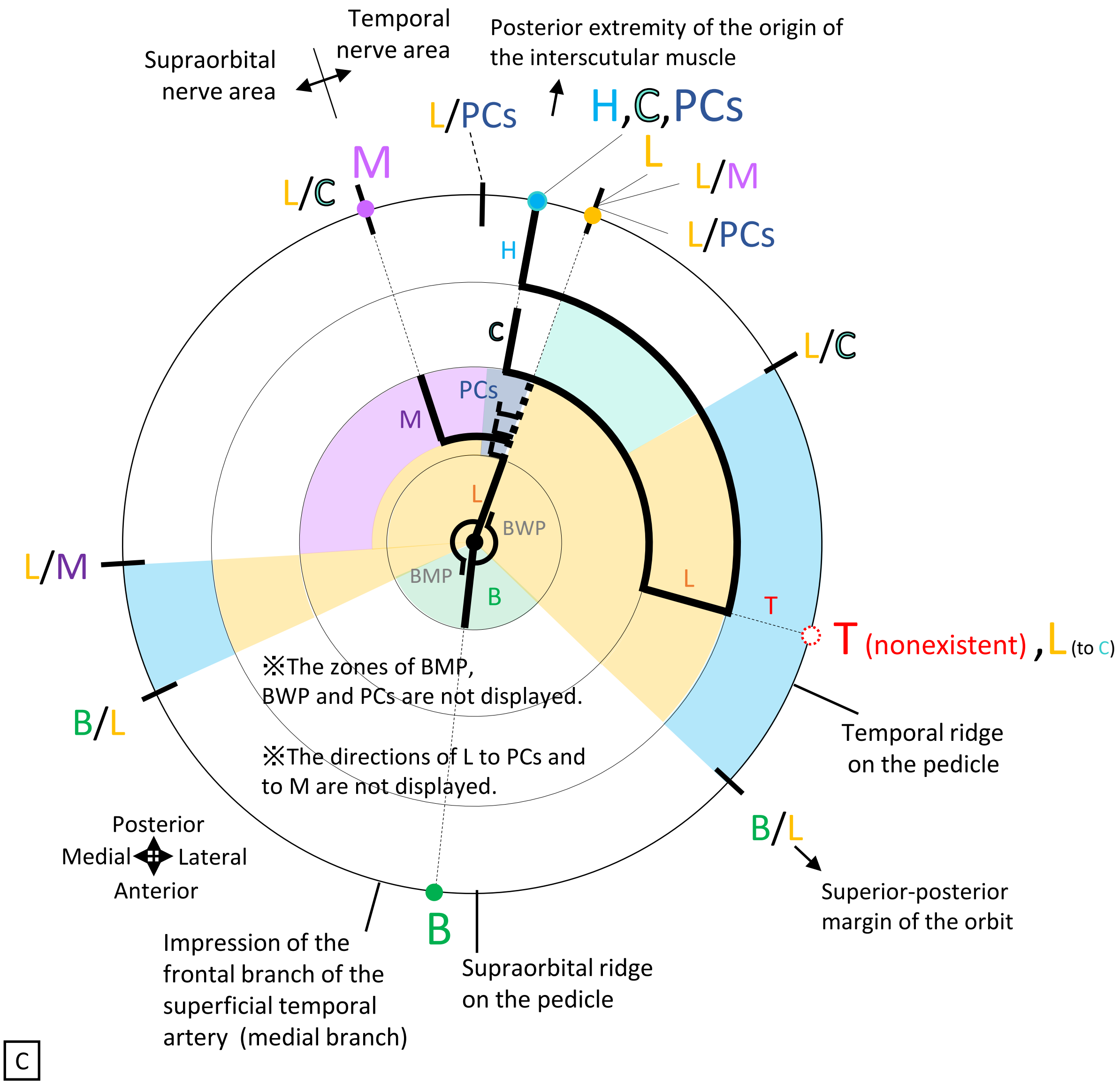

Figure 28. *Panolia eldii* (HUBG-10286) C) Diagram of the left antler.

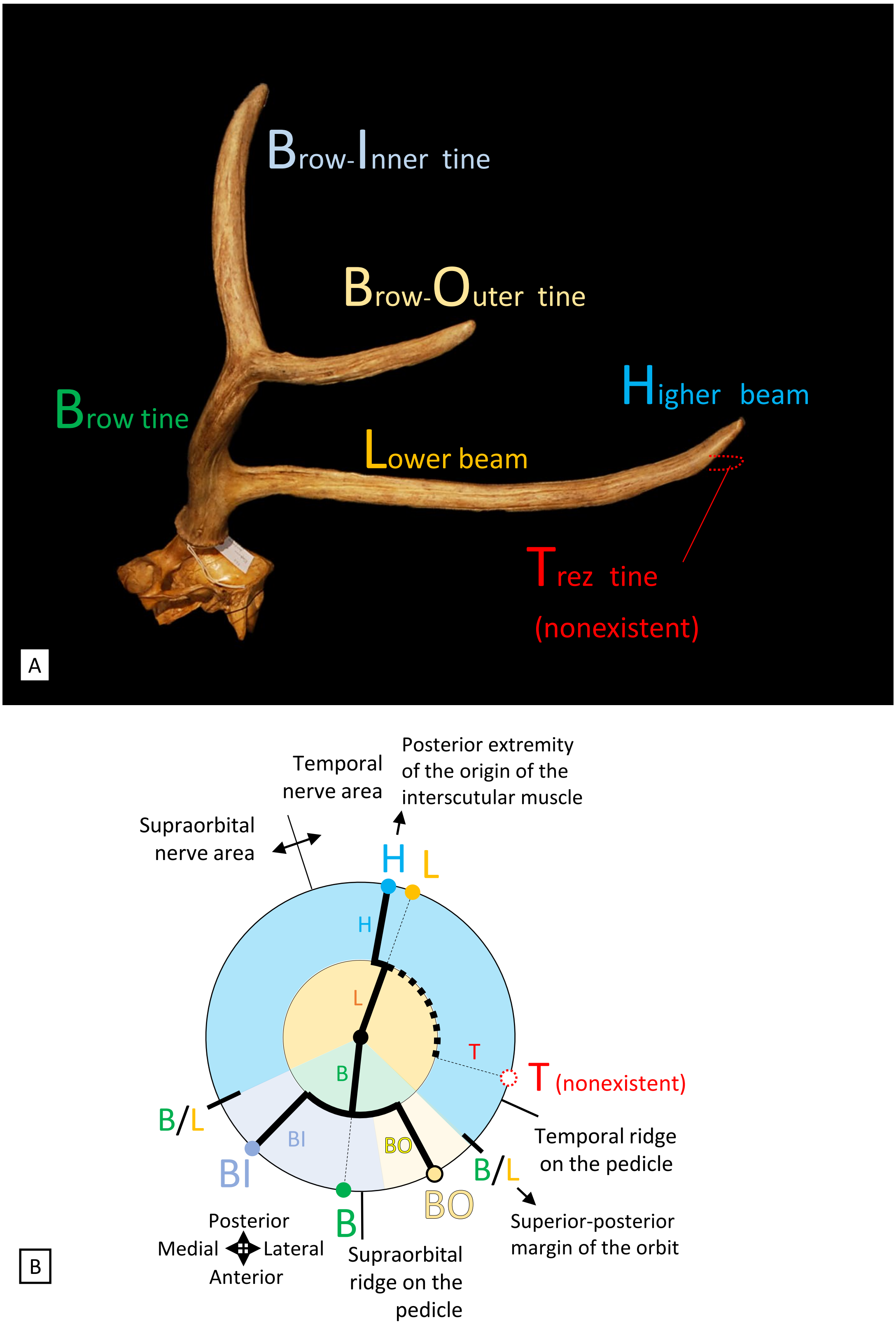

Figure 29. *Elaphurus davidianus* (KUGM-RM022) A) Left antler, lateral view. B) Diagram of the left antler.

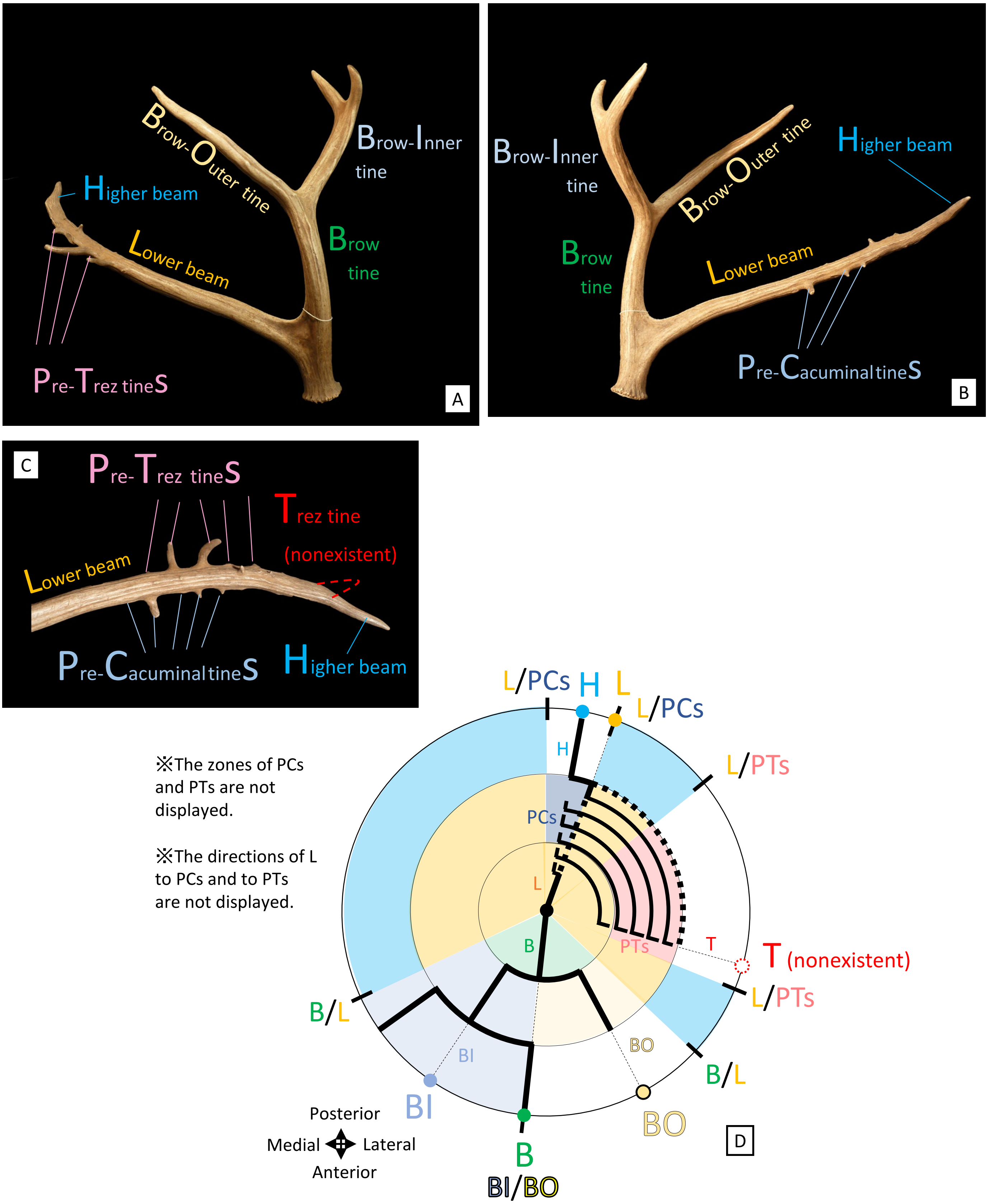

Figure 30. *Elaphurus davidianus* (KUGM-RM044) A) Right antler, lateral view. B) Right antler, medial view. C) Distal portion of the right antler, dorsal view. D) Diagram of the right antler (horizontally flipped) .

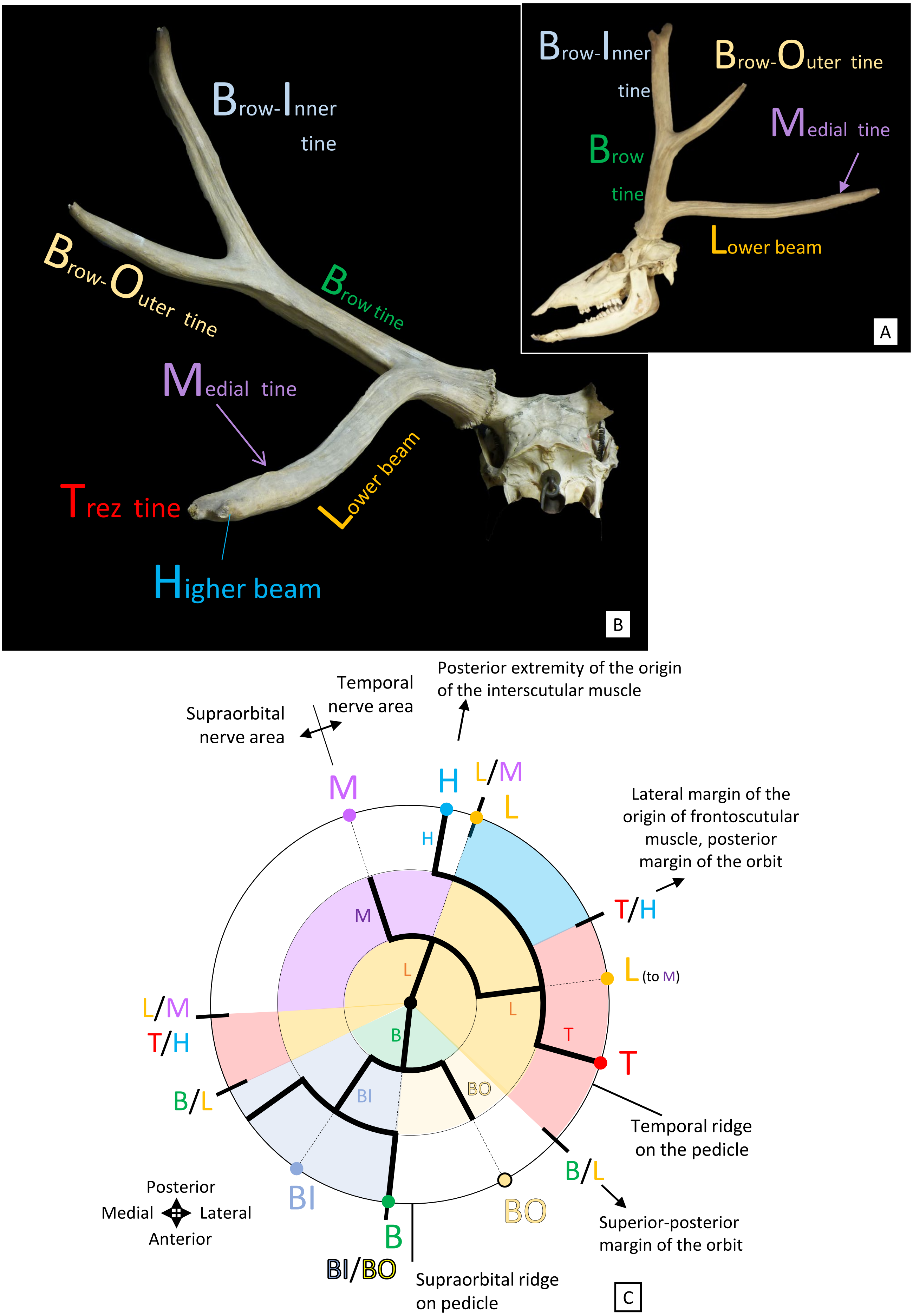

Figure 31. *Elaphurus davidianus* (NSMT-M00464) A) Left antler, lateral view. B) Left antler, posterior view. C) Diagram of the left antler.

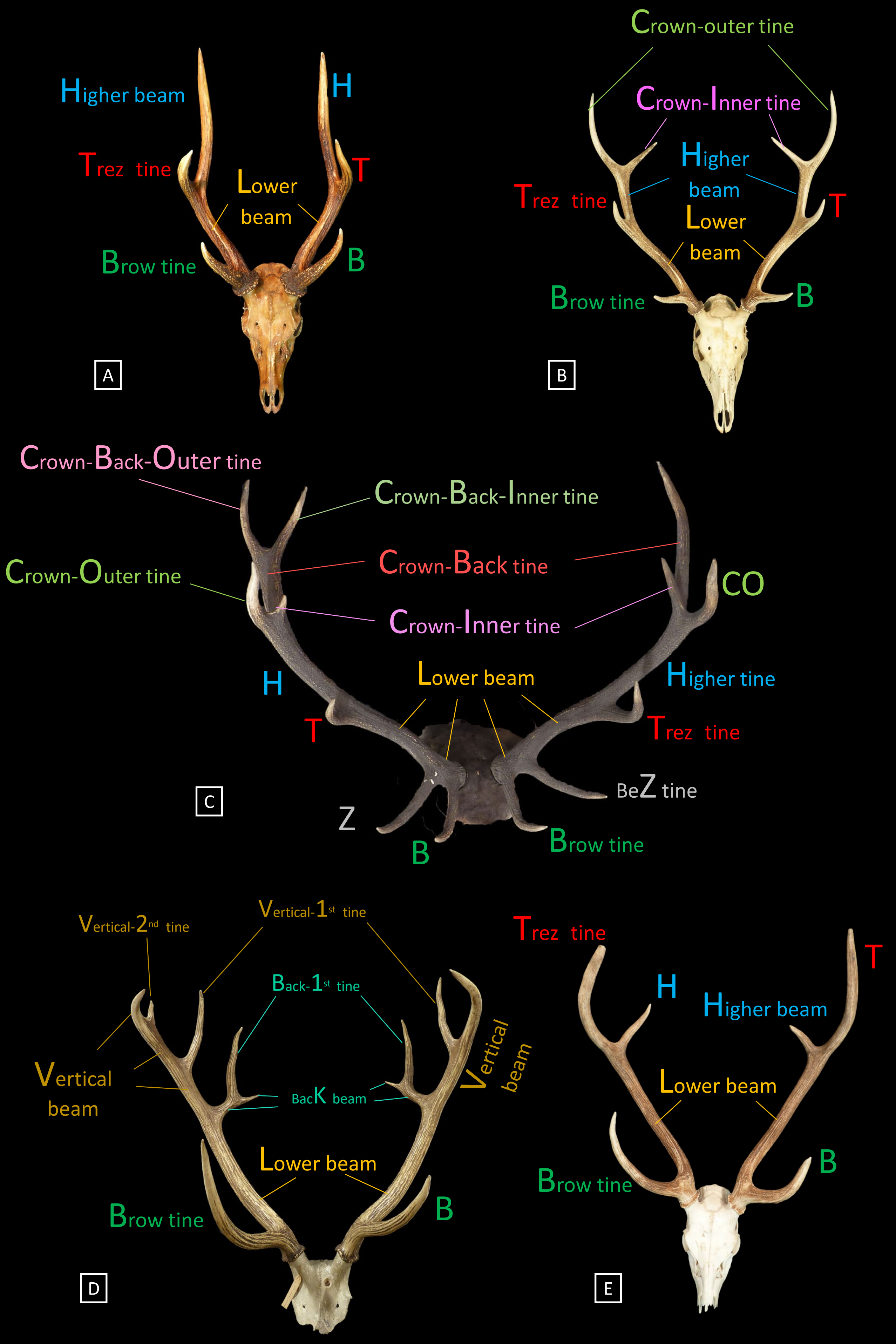

Figure 32. Comparison of A) *Rusa timorensis* (KUGM-RM021) , B) *Cervus nippon* (KUGM-RM003) , C) *Cervus elaphus* (NSMT-No number) , D) *Rucervus duvaucelii* (NSMT-M01152) and E) *Axis axis* (KUGM-RM014) in the dorsal view.

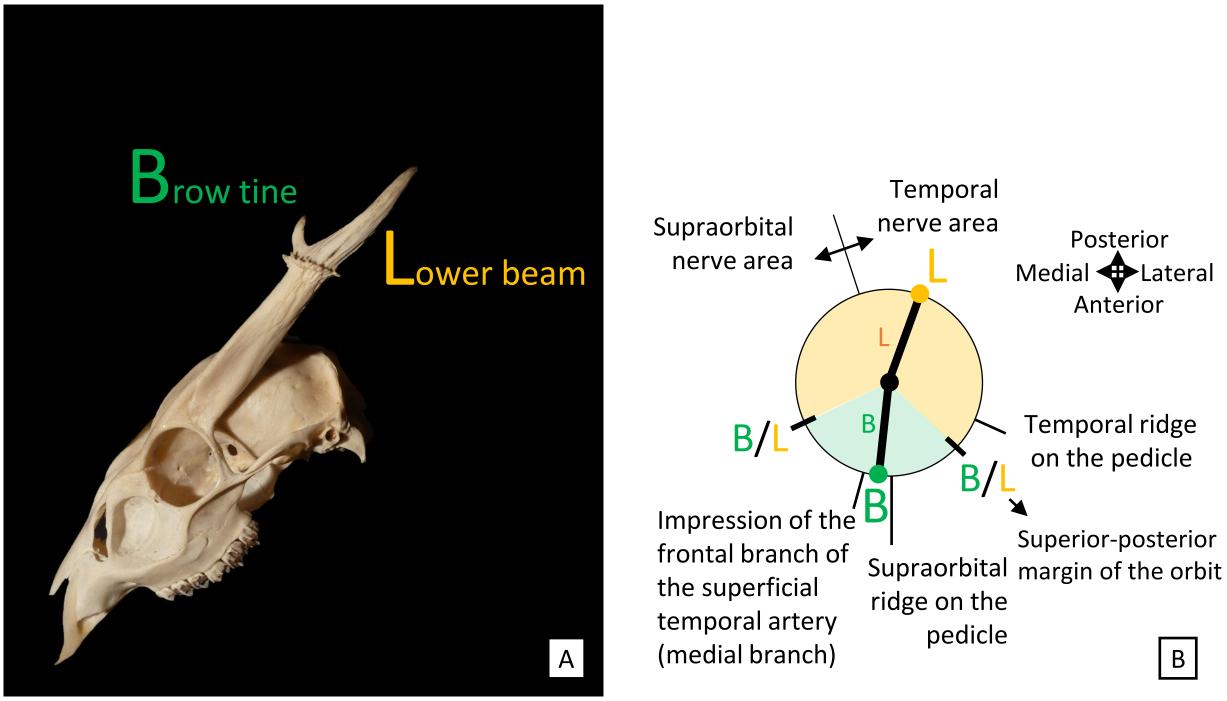

Figure 33. *Muntiacus reevisi* (KUGM-RM026) A) Left antler, lateral view. B) Diagram of the left antler.

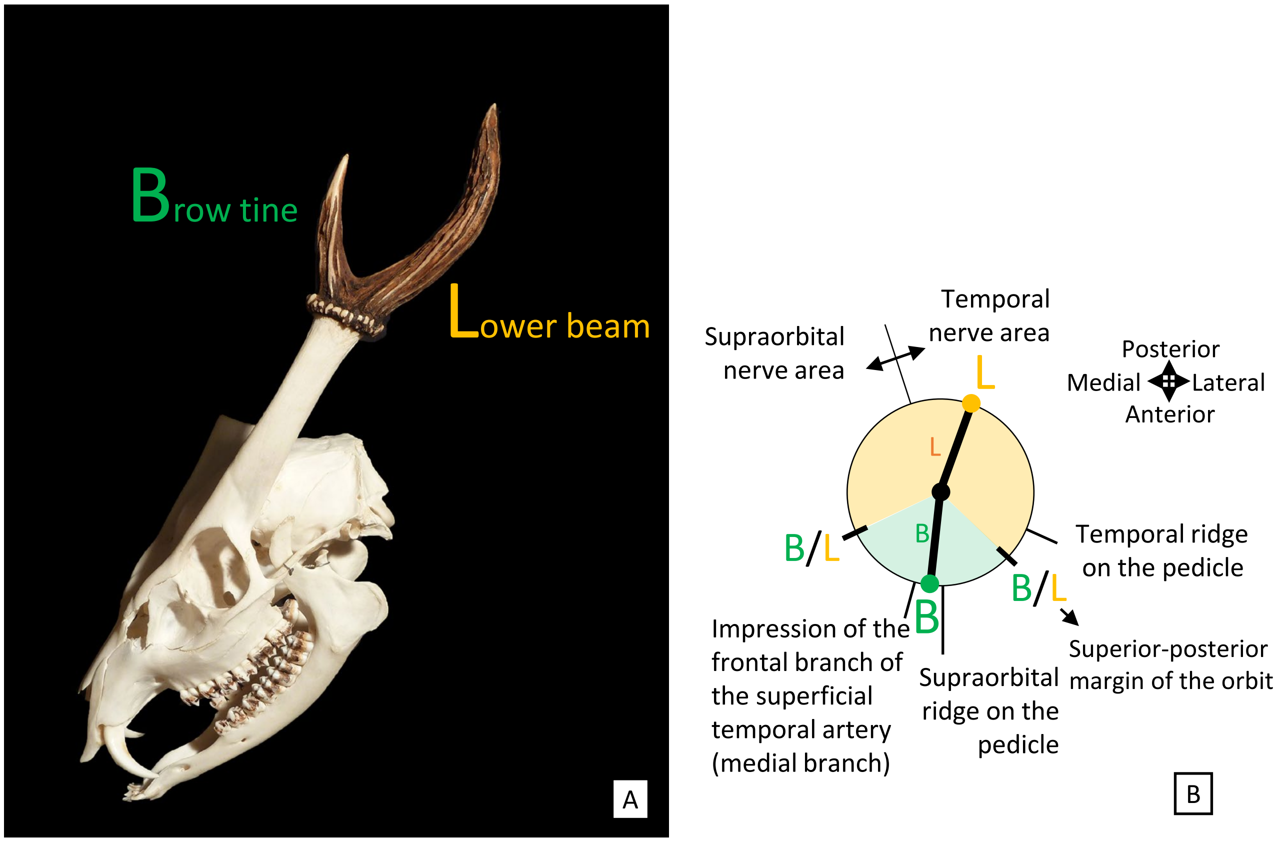

Figure 34. *Muntiacus muntjak* (KUGM-RM023) A) Left antler, lateral view. B) Diagram of the left antler.

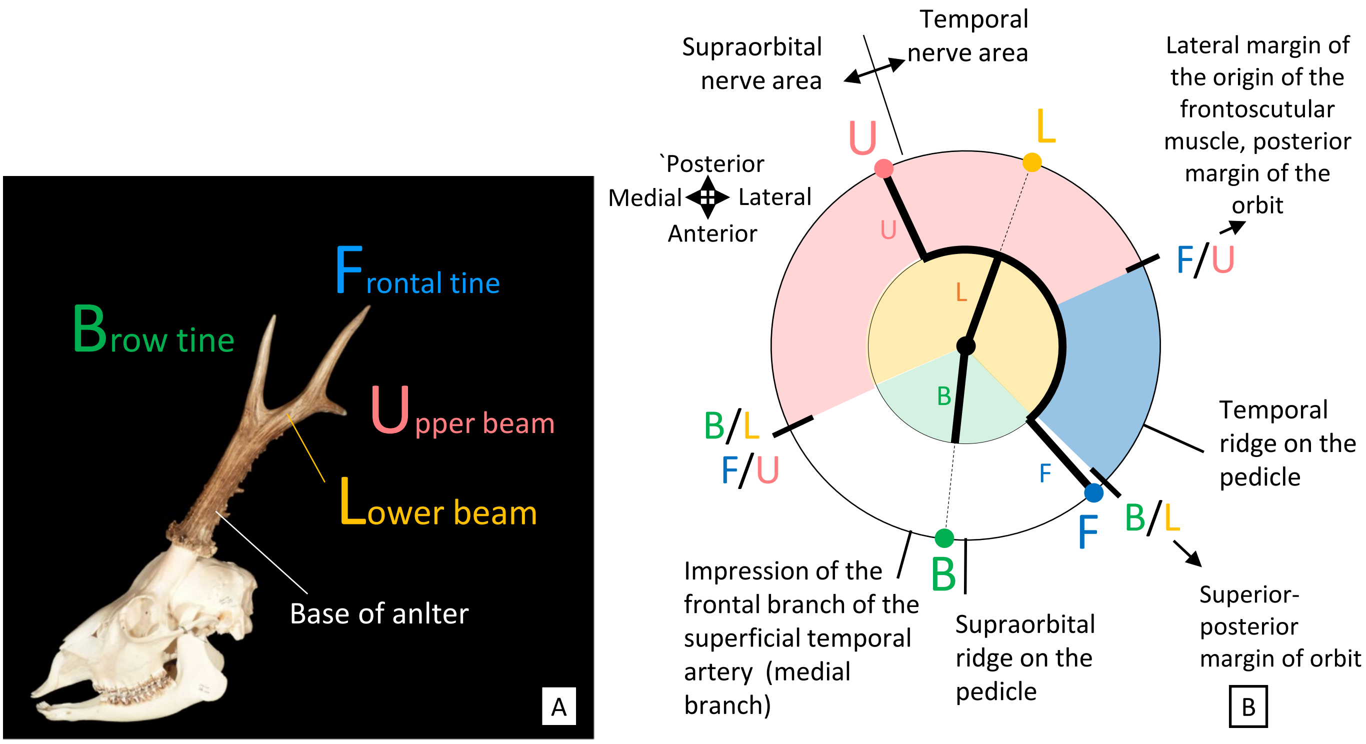

Figure 35. *Capreolus capreolus* (KUGM-RM027) A) Left antler, lateral view. B) Diagram of the left antler.

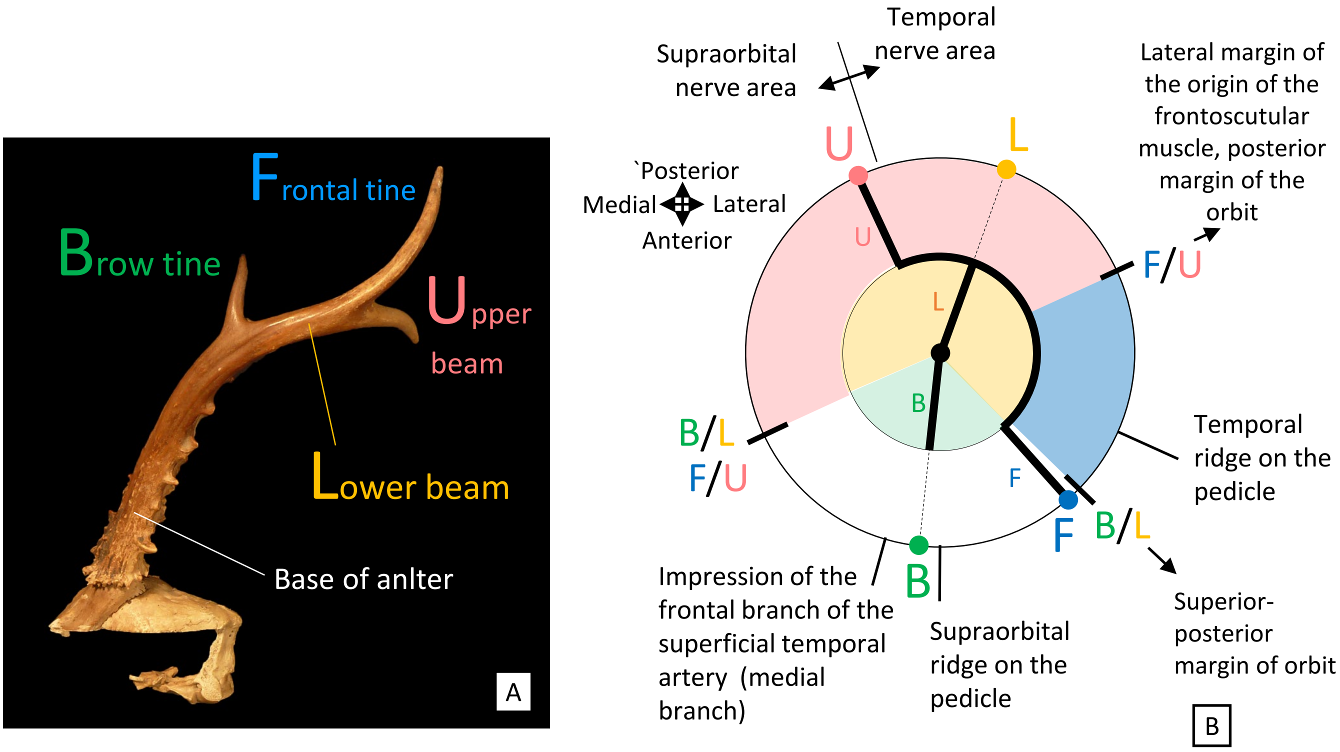

Figure 36. *Capreolus pygargus* (KUGM-RM081) A) Left antler, lateral view. B) Diagram of the left antler.

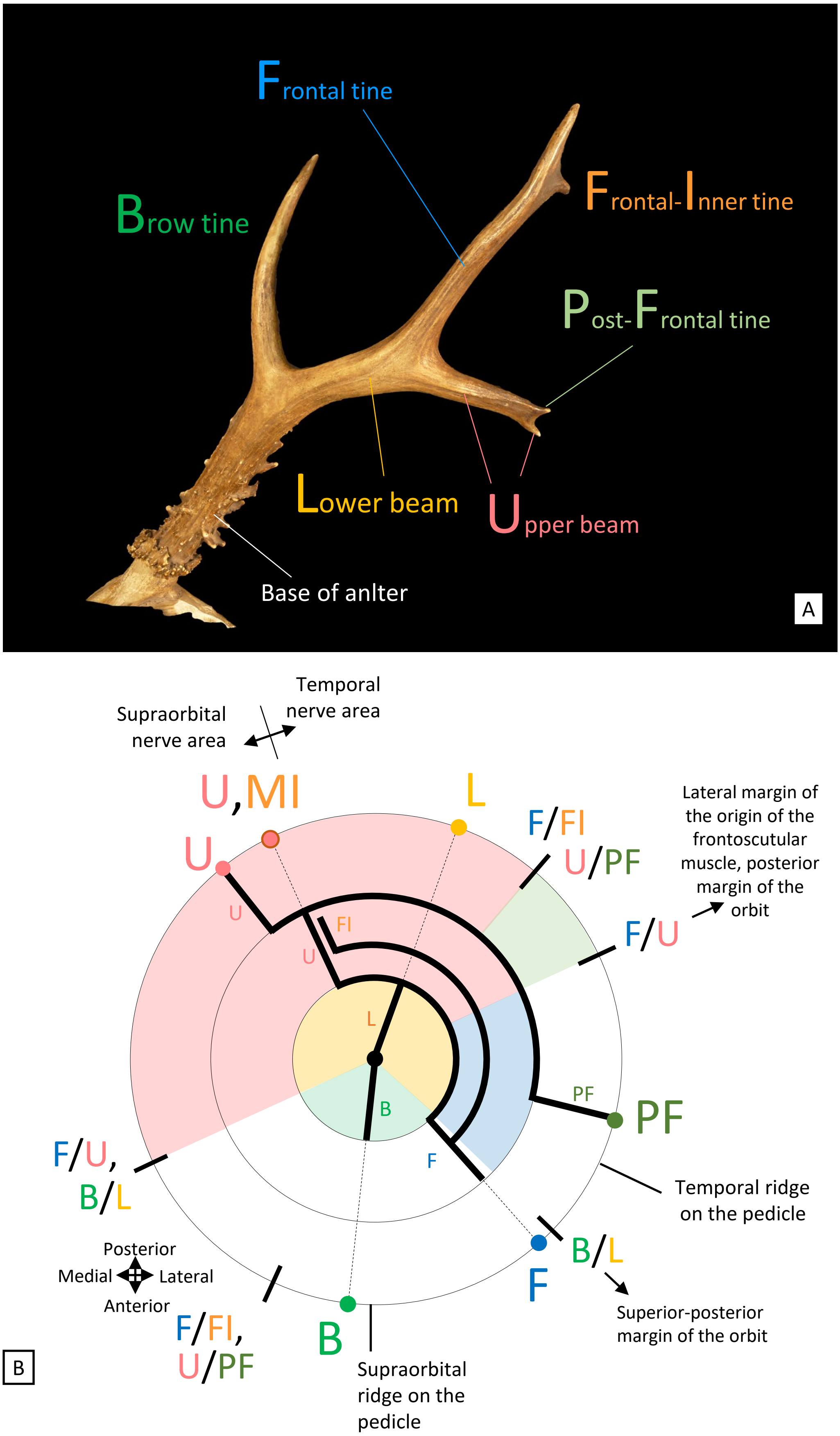

Figure 37. *Capreolus pygargus* (KUGM-RM131) A) Left antler, lateral view. B) Diagram of the left antler.

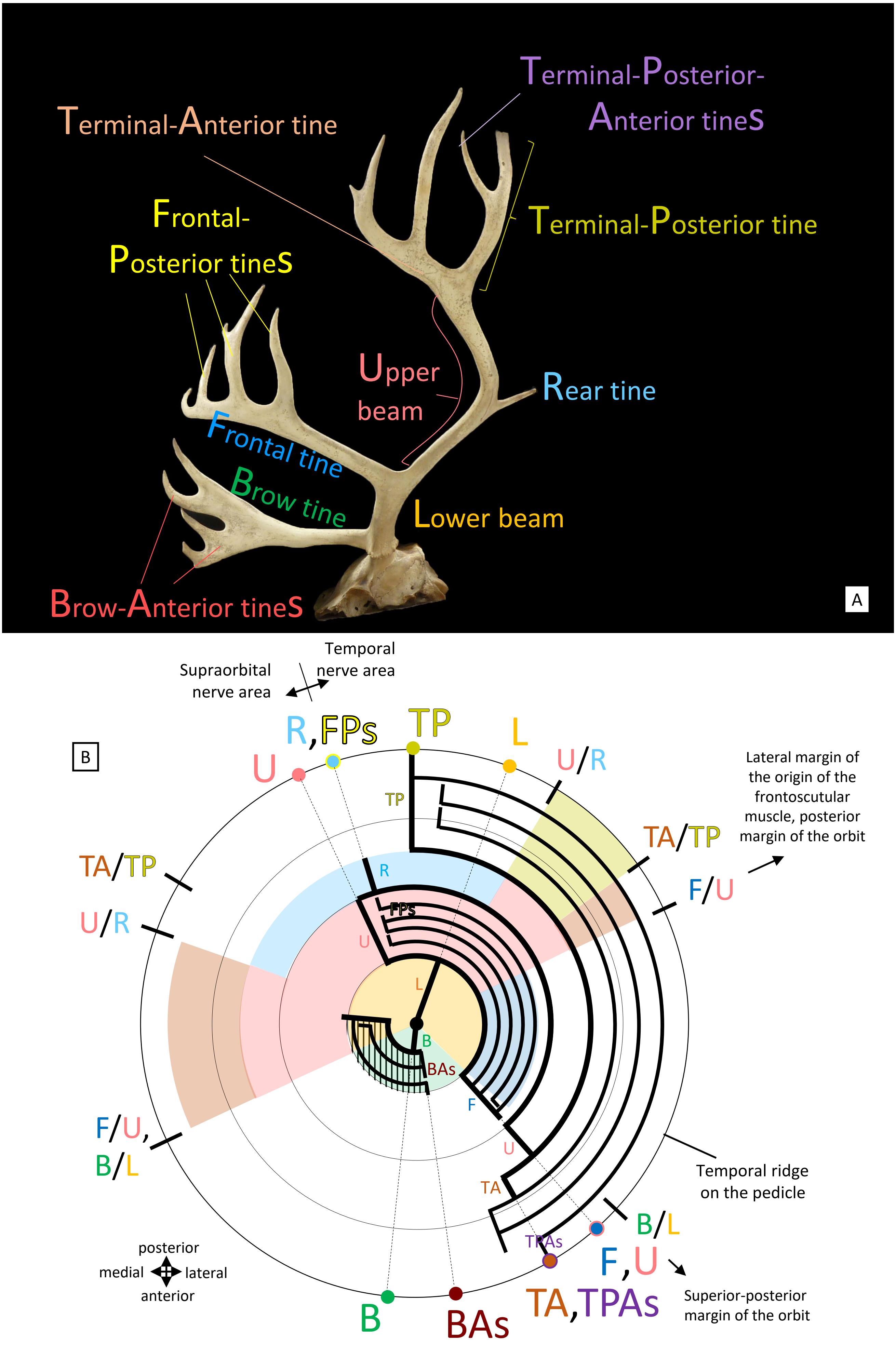

Figure 38. *Rangifer tarandus* (NSMT-M56438) A) Left antler, lateral view. B) Diagram of the left antler.

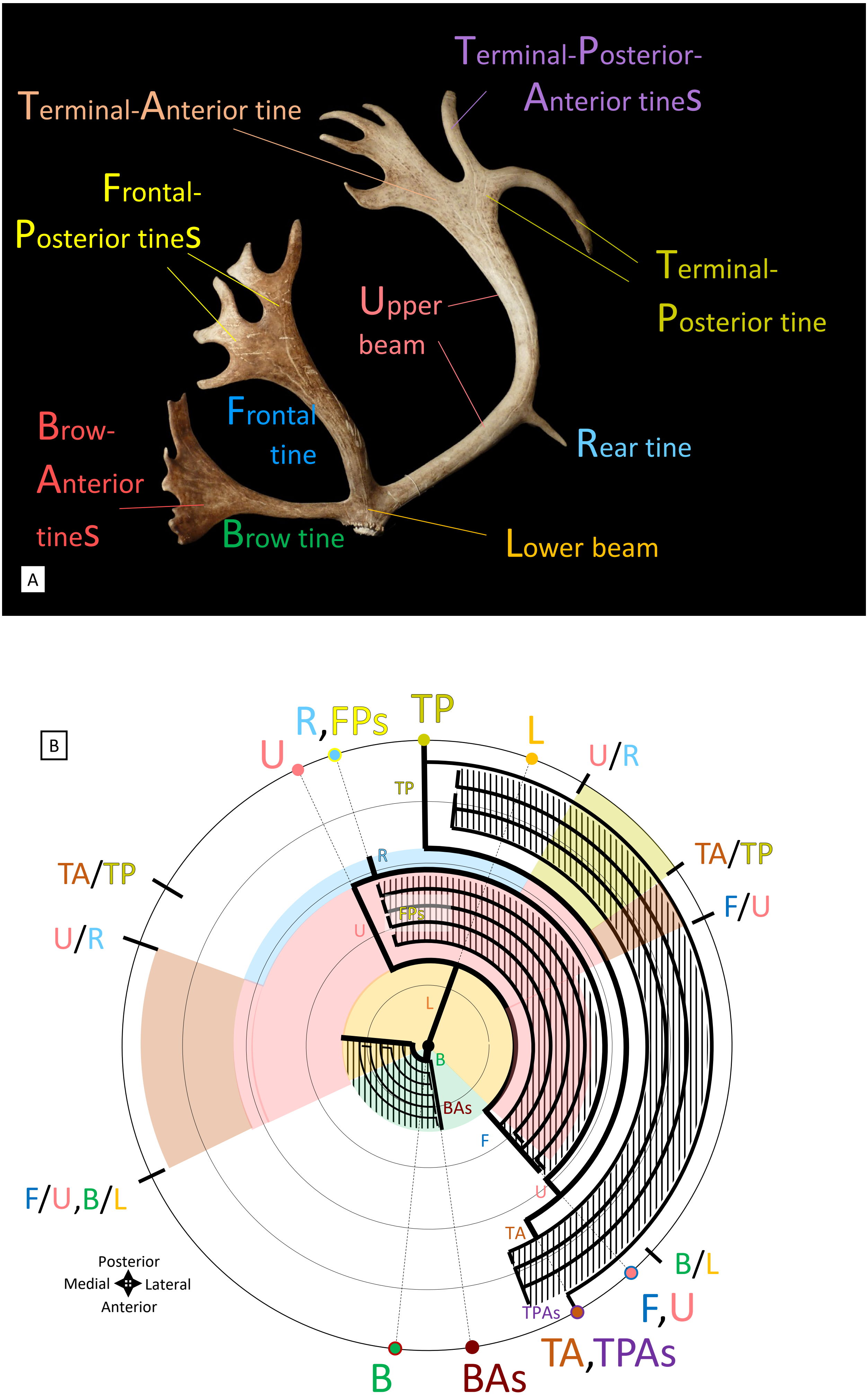

Figure 39. *Rangifer tarandus* (KUGM-RM052) A) Right antler, lateral view (horizontally flipped) . B) Diagram of the right antler (horizontally flipped) .

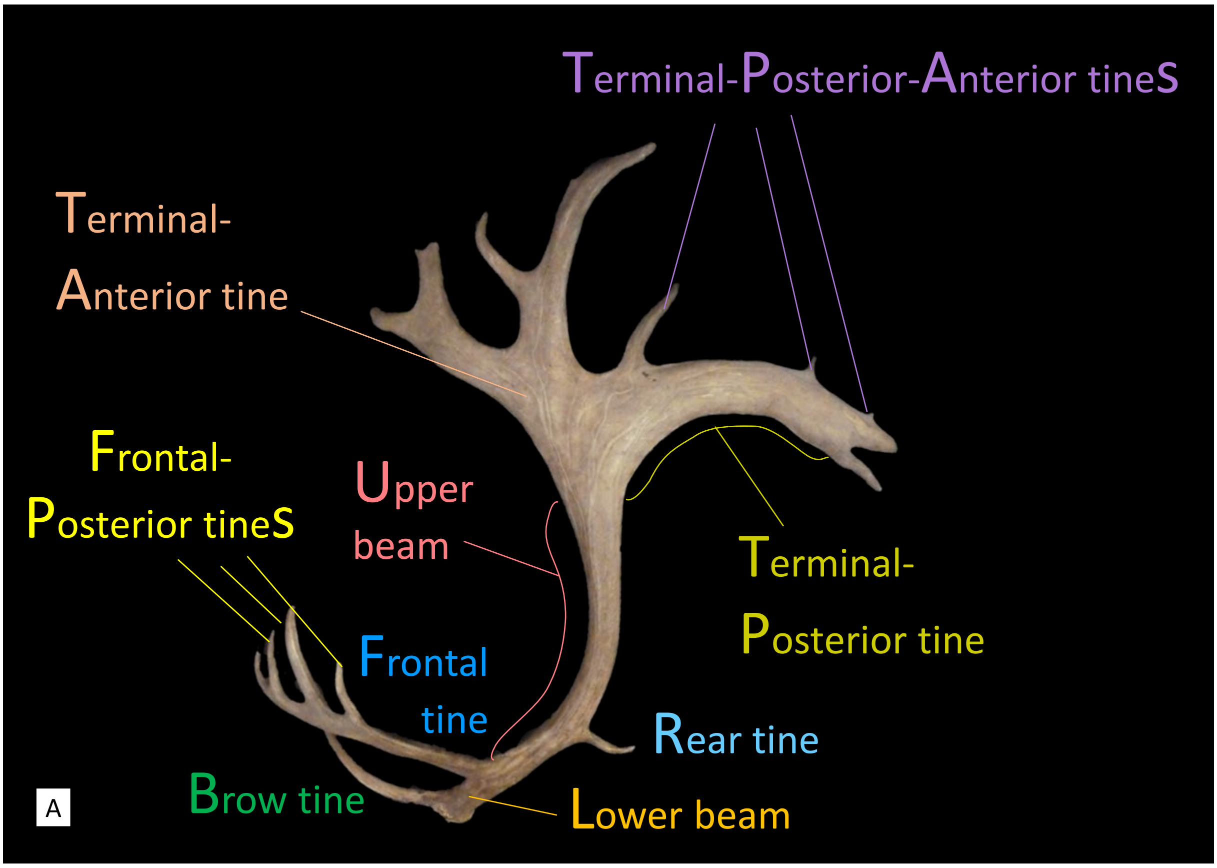

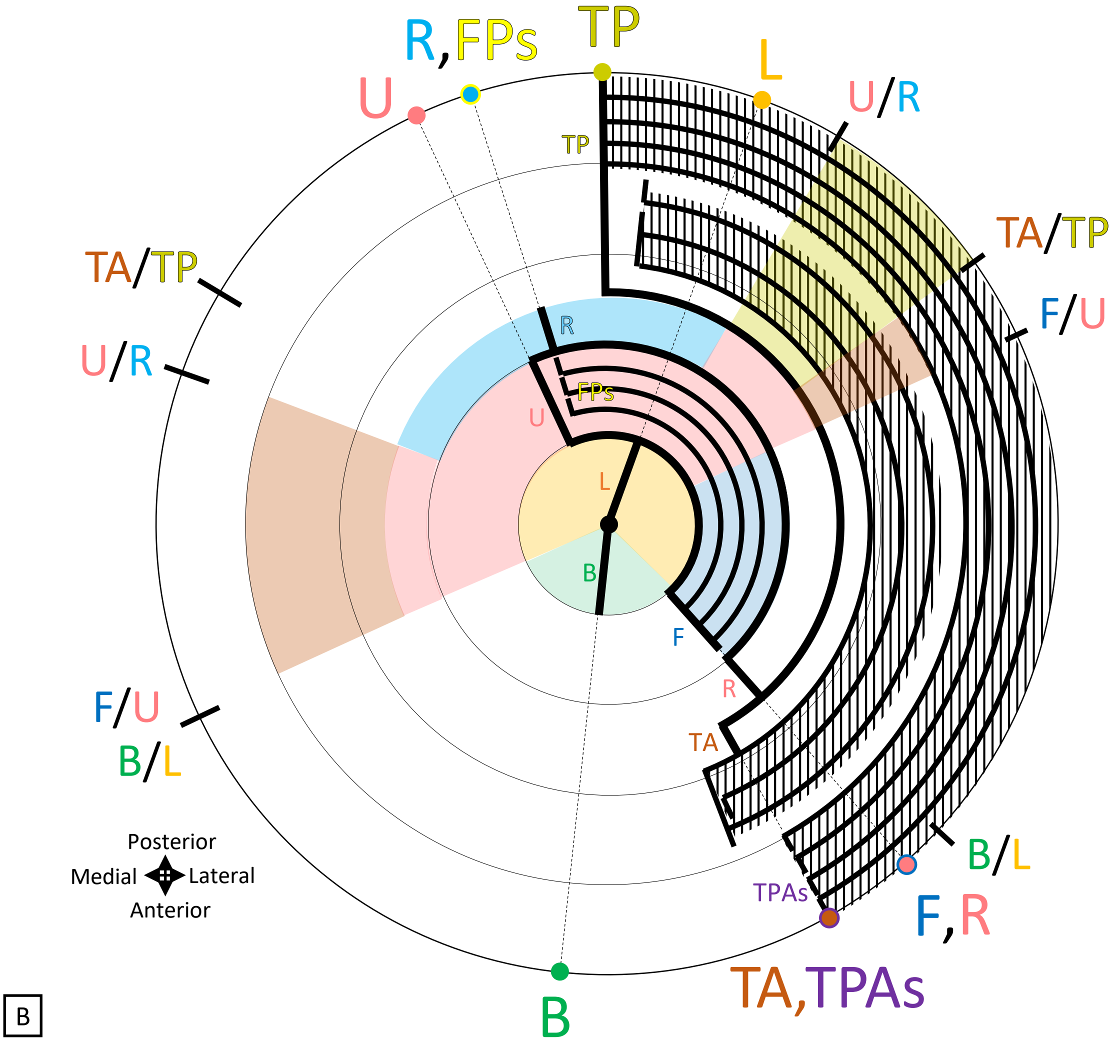

Figure 40. *Rangifer tarandus* (NSMT-M43364) A) Right antler, lateral view (horizontally flipped) . B) Diagram of the right antler (horizontally flipped) .

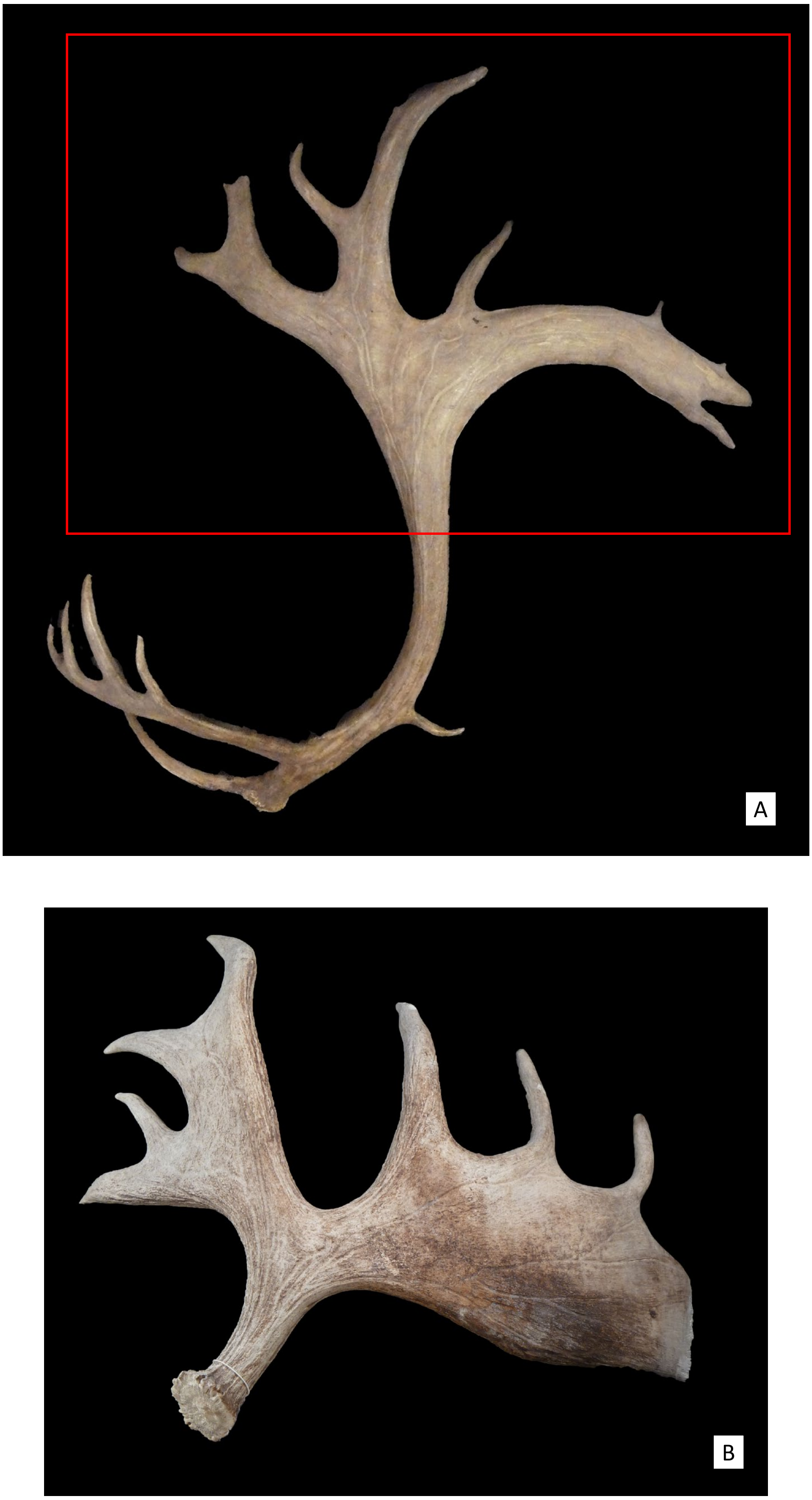

Figure 41. Comparison of A) *Rangifer tarandus* (NSMT-M43364) (horizontally flipped) and B) *Alces alces* (KUGM-RM049) . The palmate part of *R.tarandus* surrounded by the red square is resembles *A.alces.*

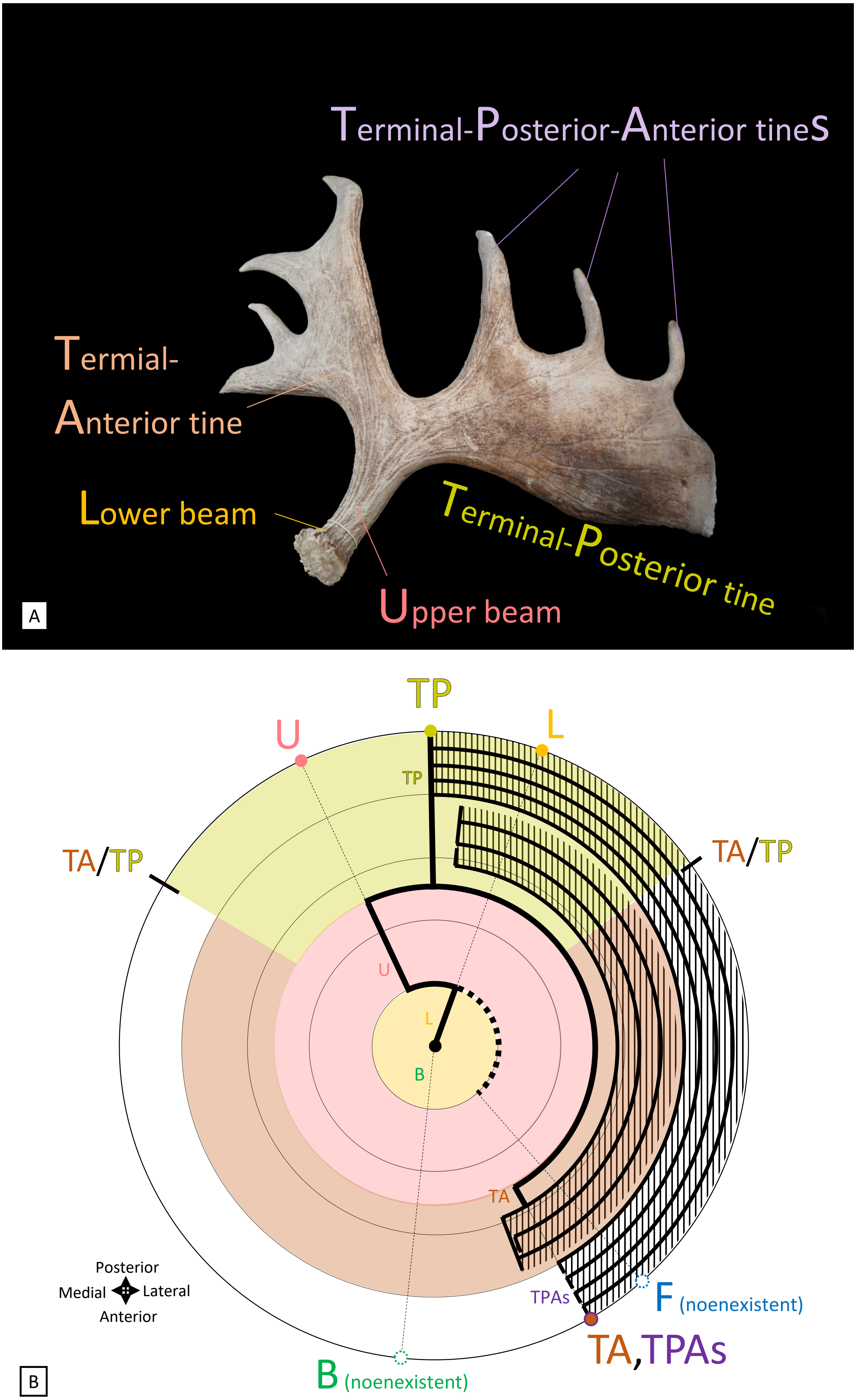

Figure 42. Typical antler of *Alces alces* (KUGM-RM049) . A) Left antler, ventral-lateral view . B) Diagram of the left antler.

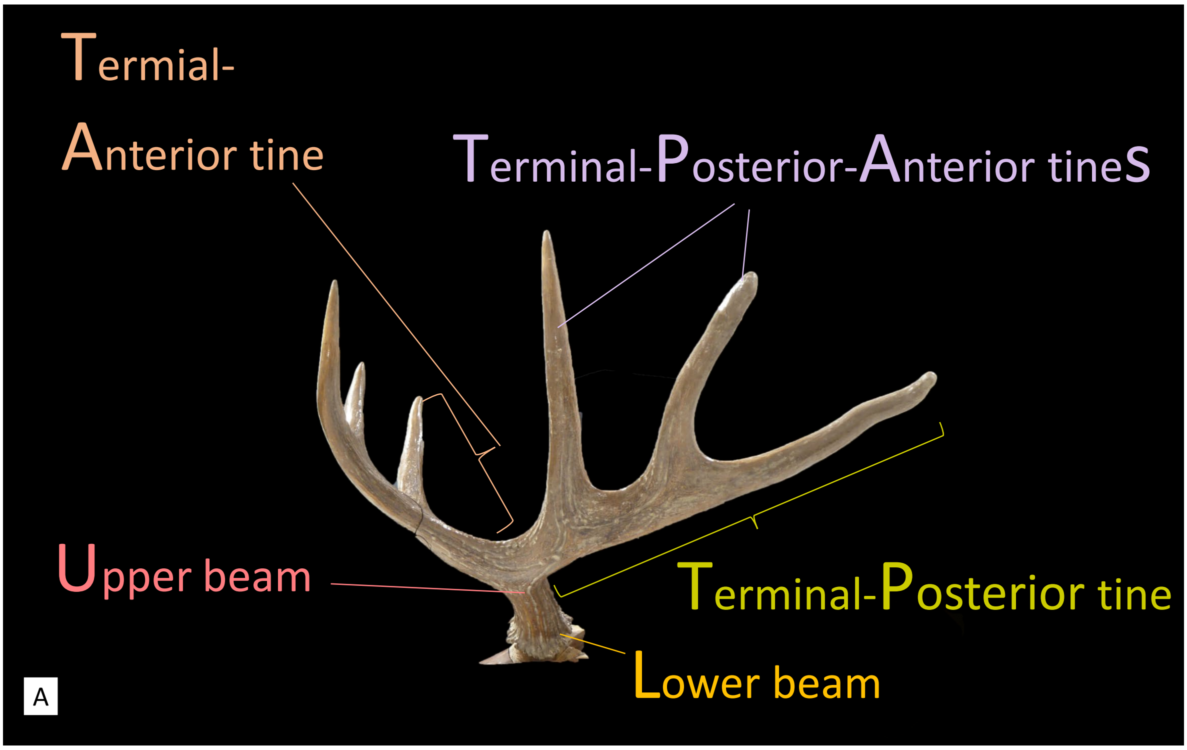

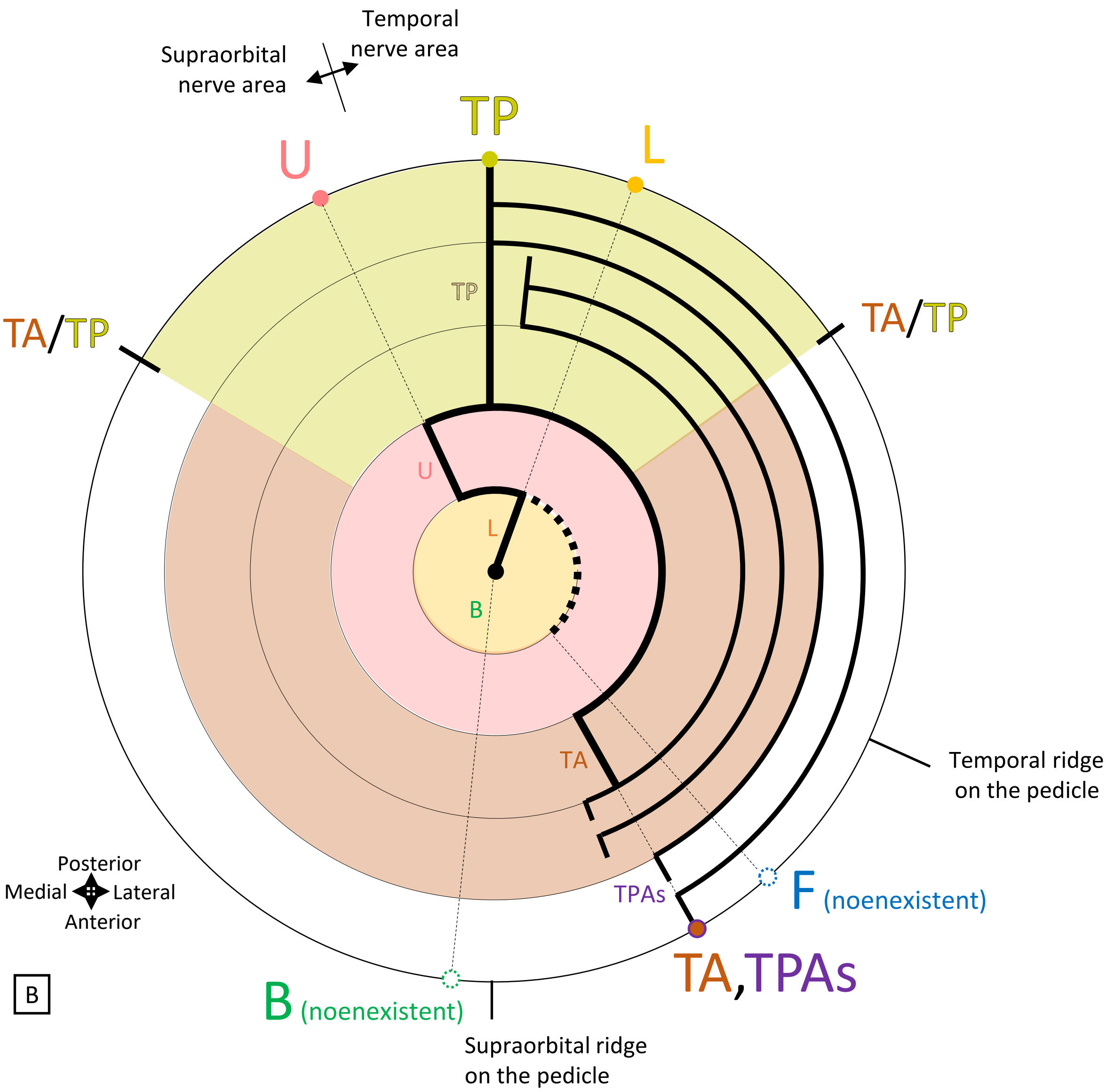

Figure 43. Non-palmating antler of *Alces alces* (NSMT-M43310) . A) Left antler, lateral view . B) Diagram of the left antler.

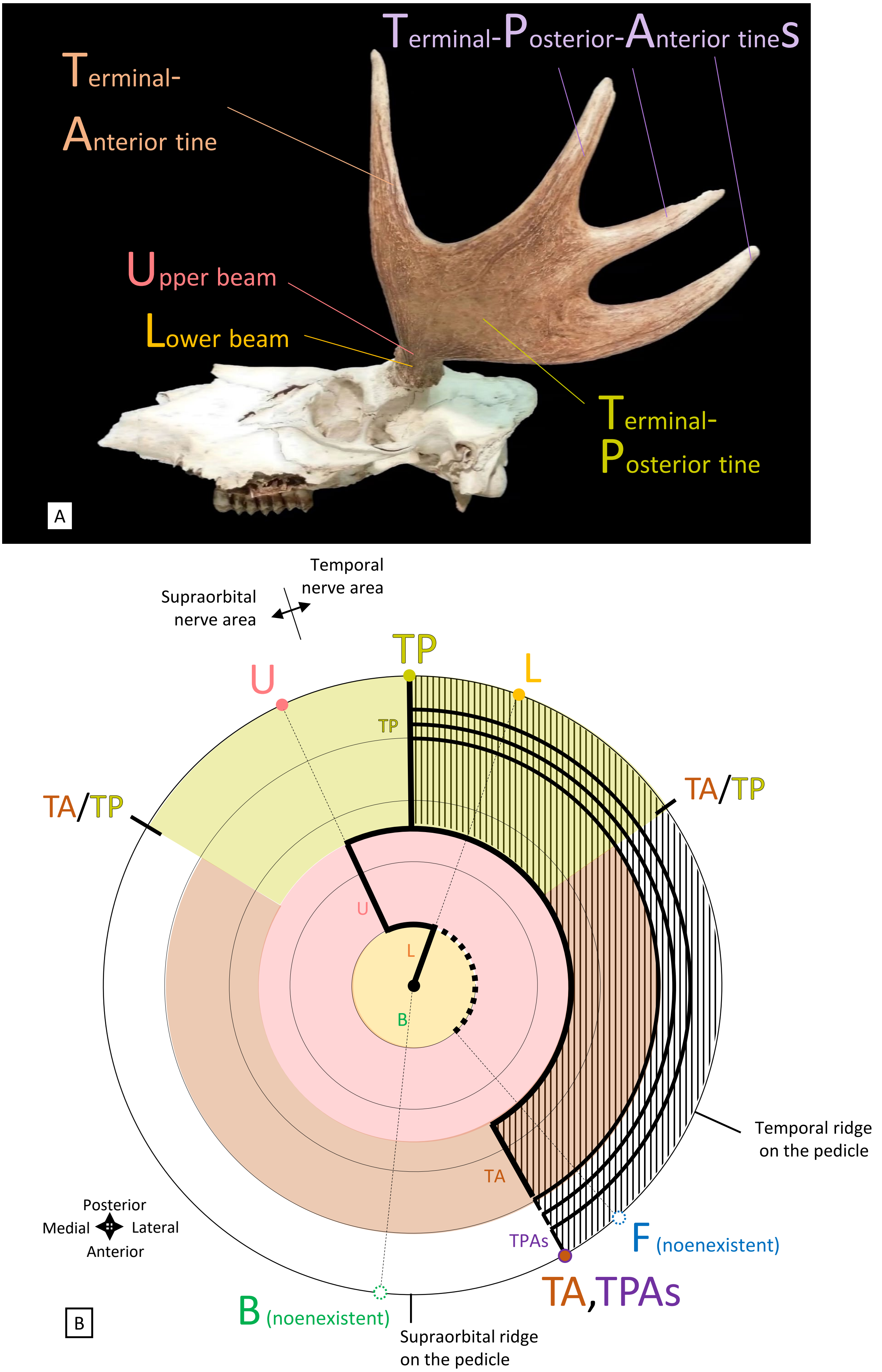

Figure 44. *Alces alces* (KUGM-RM029) . A) Left antler, lateral view . B) Diagram of the left antler.

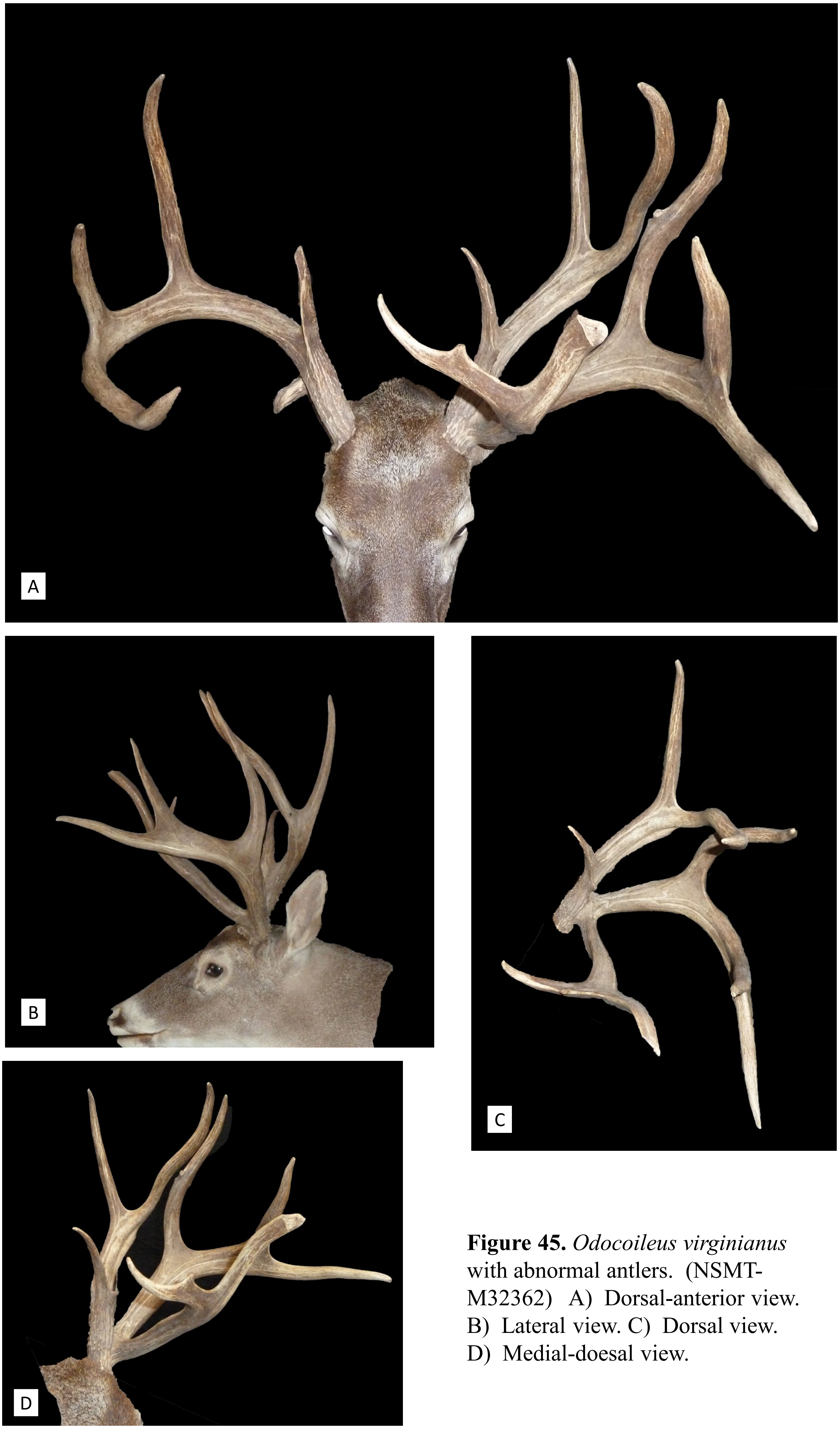

Figure 45. *Odocoileus virginianus* with abnormal antlers. (NSMT-M32362) A) Dorsal-anterior view. B) Lateral view. C) Dorsal view. D) Medial-doesal view.

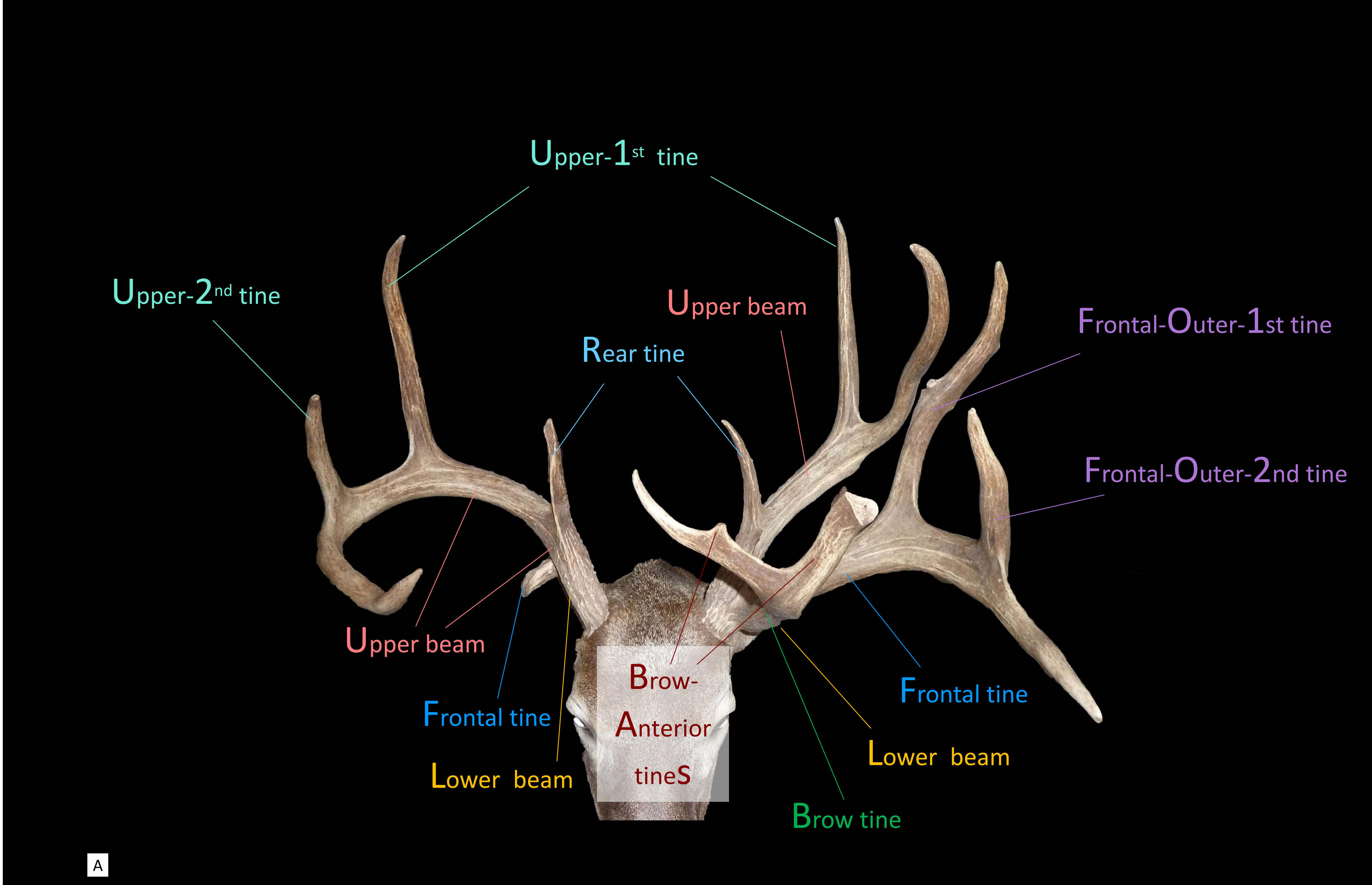

Figure 46. *Odocoileus virginianus* (NSMT-M32362) A) Dorsal-anterior view, with terminology of the tines.

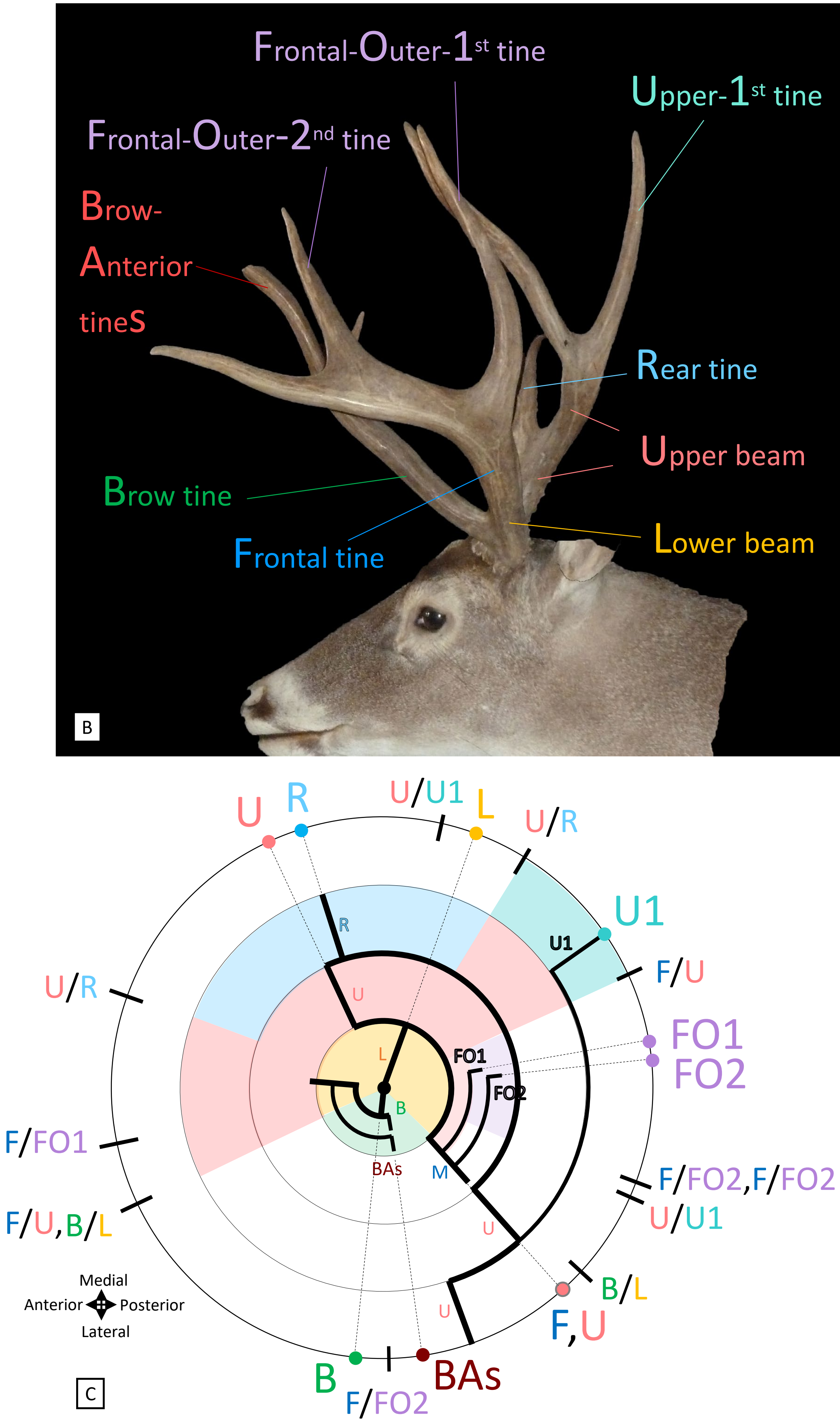

Figure 47. Abnormal antler of *Odocoileus virginianus* (NSMT-M32362) A) Left antler, lateral view. B) Diagram of the left antler.

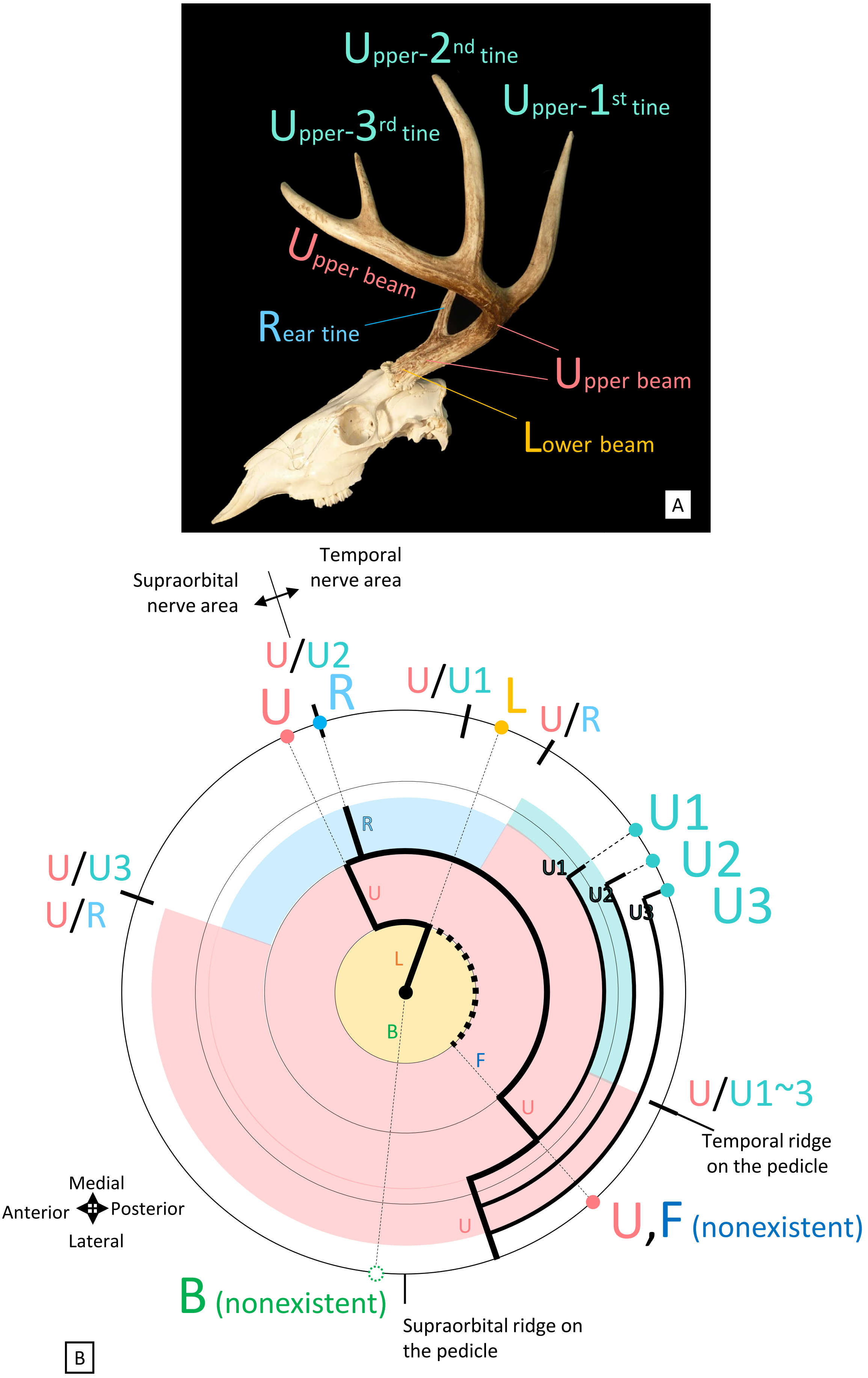

Figure 48. Typical antler of *Odocoileus virginianus*, with normal antlers. (KUGM-RM033) A) Right antler, lateral view. (horizontally flipped) , B) Diagram of the right antler. (horizontally flipped)

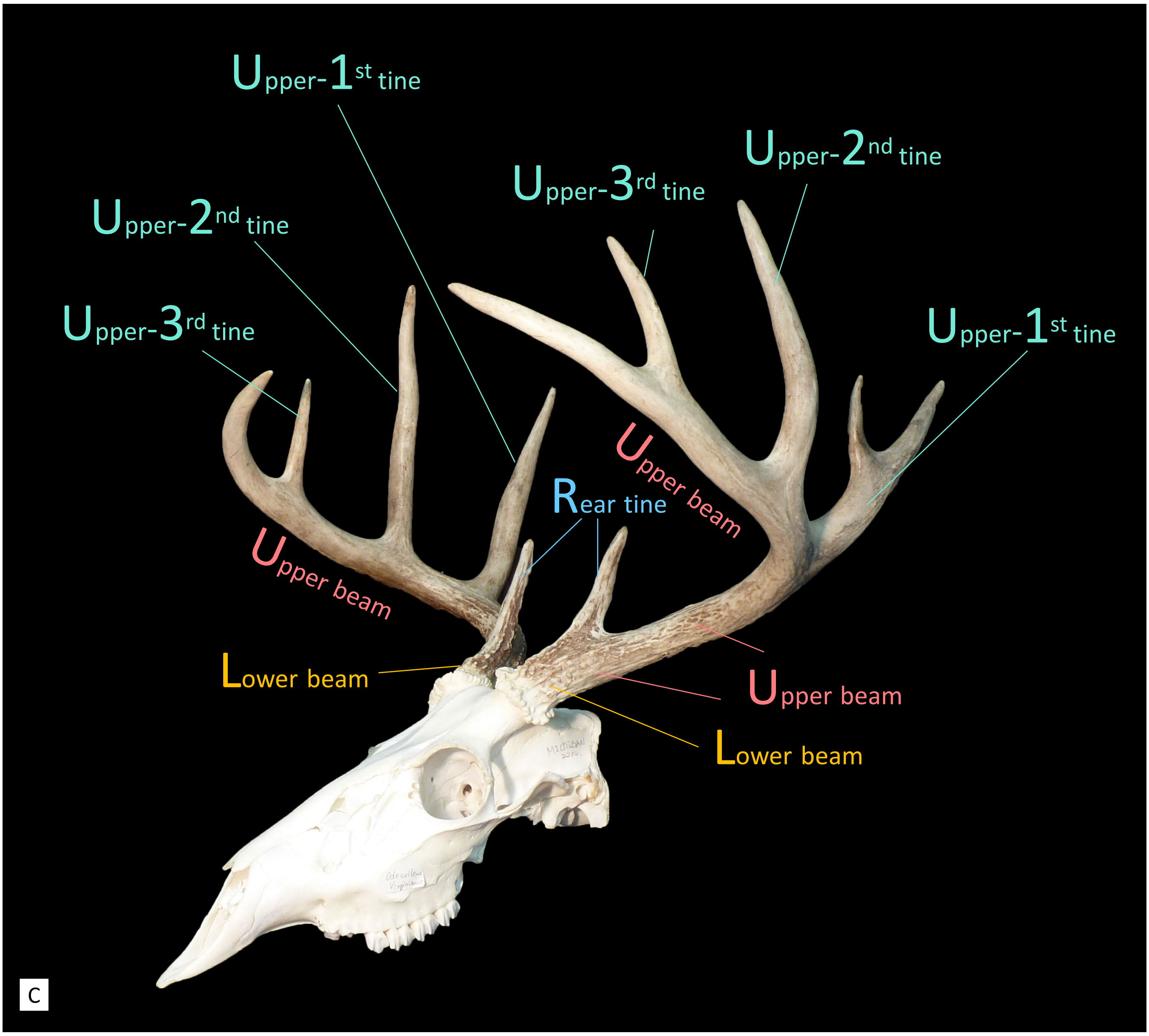

Figure 49. *Odocoileus virginianus*, with normal antlers. (KUGM-RM033) C) Lateral-anterior view.

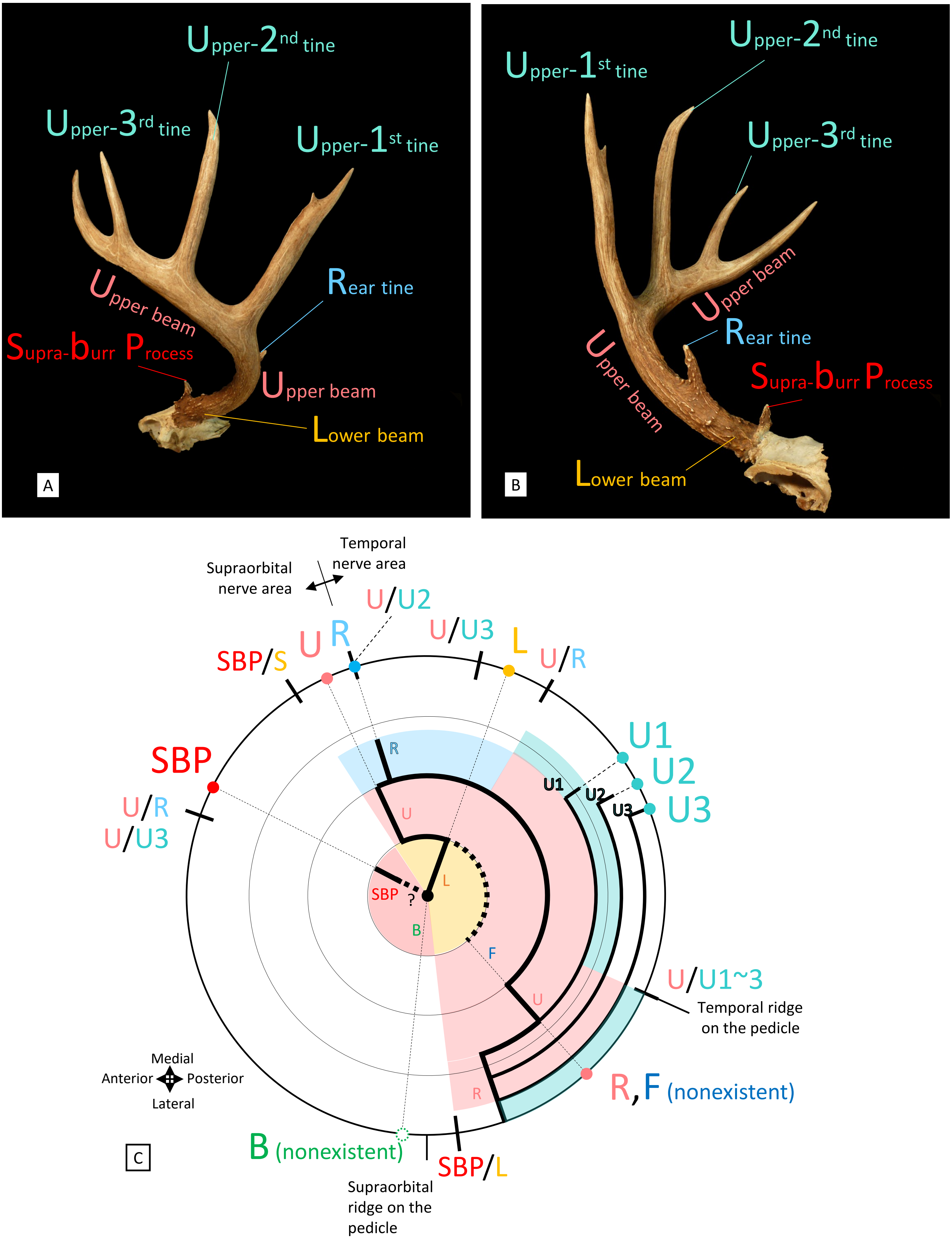

Figure 50. *Odocoileus virginianus*. (KUGM-RM208) A) Left antler, lateral view. B) Left antler, posterior-medial view. C) Diagram of the left antler.

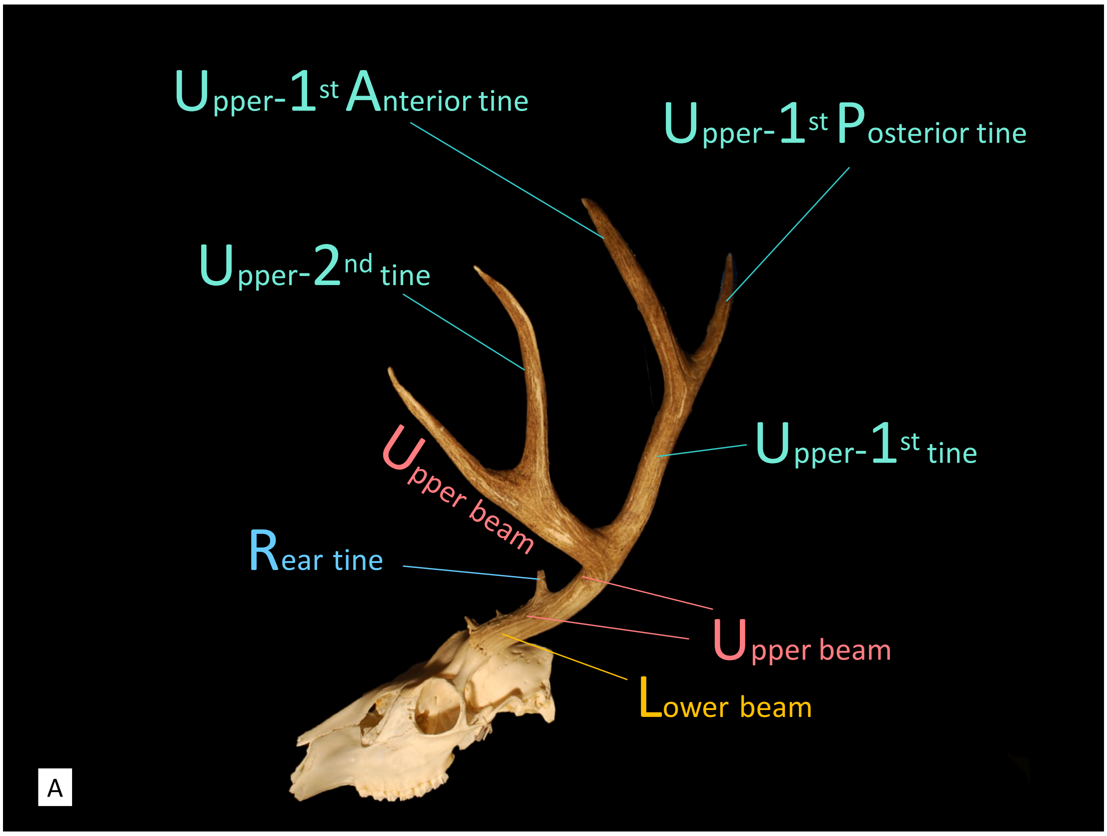

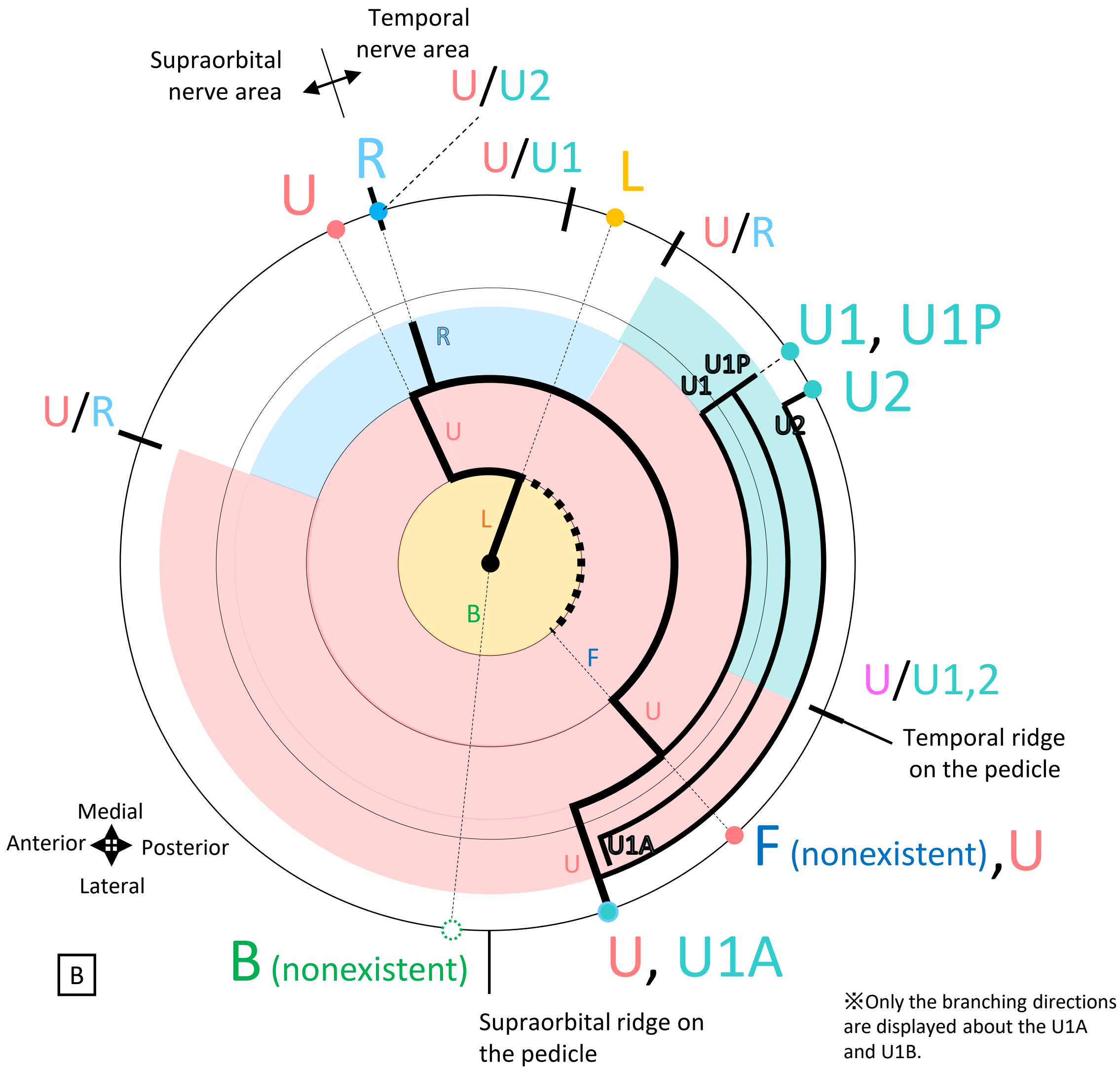

Figure 51. *Odocoileus hemionus*. (KUGM-RM032) A) Left antler, lateral view. B) Diagram of the left antler.

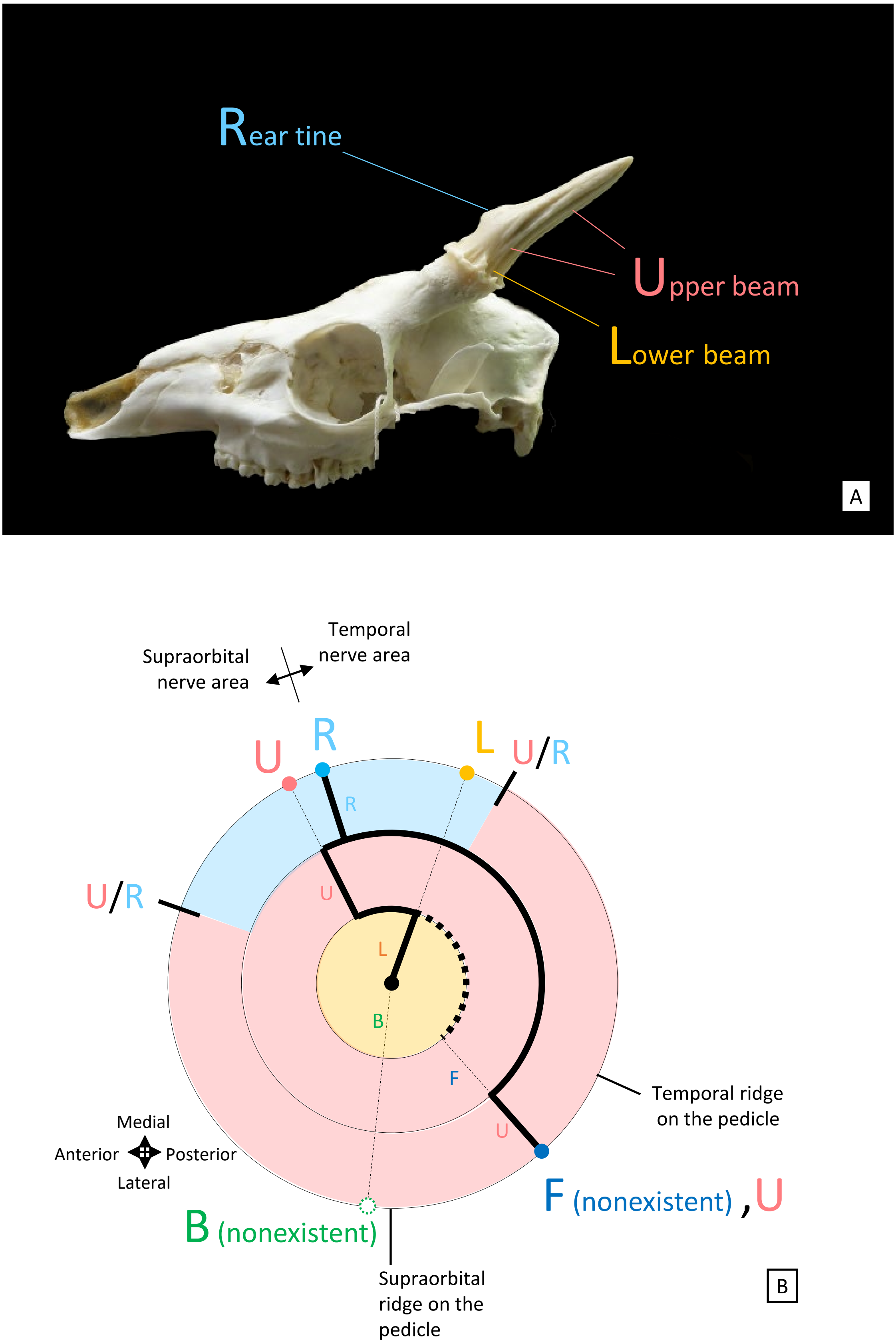

Figure 52. *Mazama americana*. (KUGM-RM035) A) Right antler, lateral view (horizontally flipped) . B) Diagram of the right antler (horizontally flipped) .

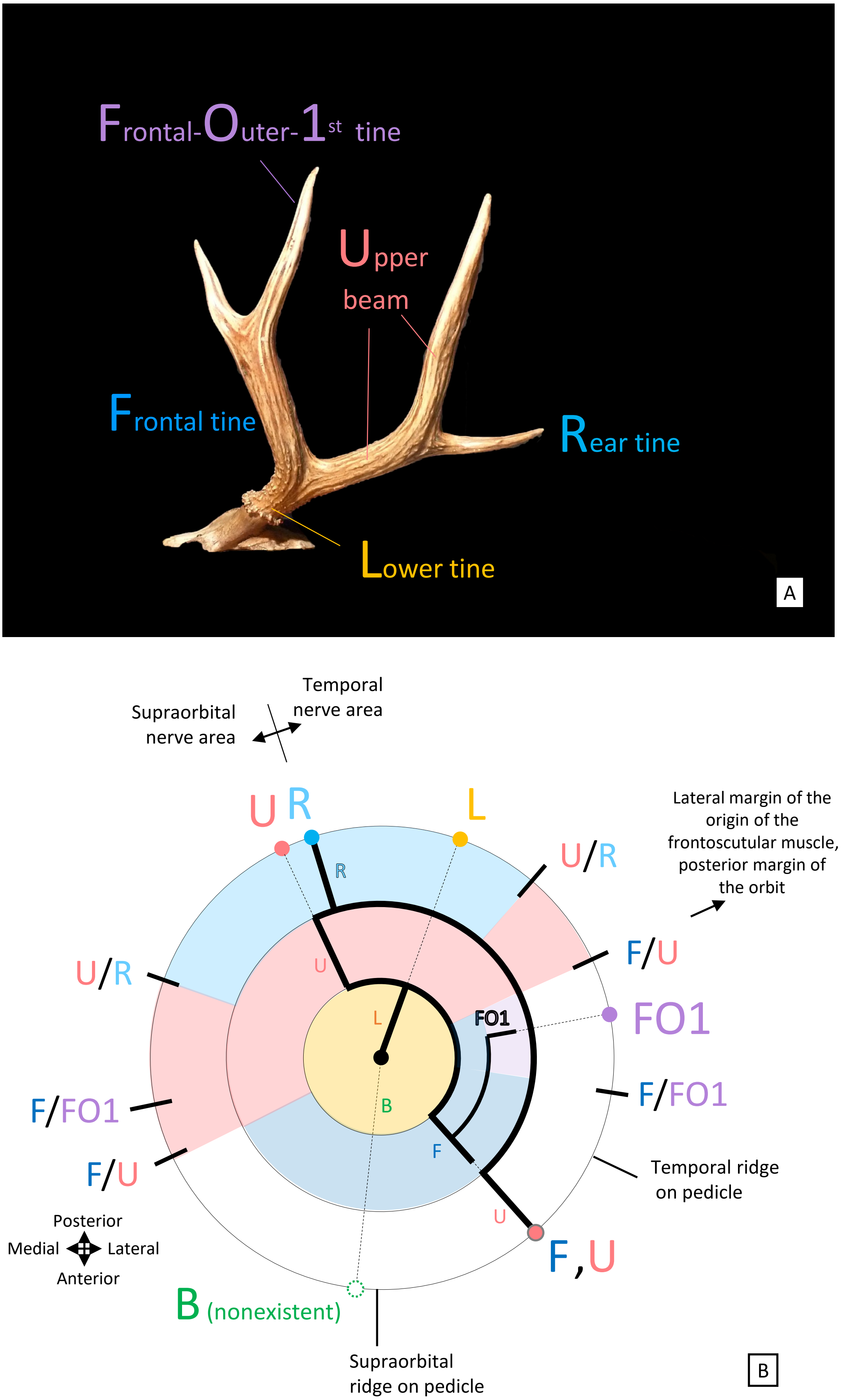

Figure 53. *Blastocerus dichotomus*. (KUGM-RM134) A) Left antler, lateral view . B) Diagram of the left antler.
